# Randomized Phase III Study of EGFR Tyrosine Kinase Inhibitor and Intercalated Platinum-Doublet Chemotherapy for Non–Small Cell Lung Cancer Harboring *EGFR* Mutation

**DOI:** 10.1158/1078-0432.CCR-24-3532

**Published:** 2025-03-31

**Authors:** Shintaro Kanda, Seiji Niho, Takayasu Kurata, Shogo Nomura, Yosuke Kawashima, Eiji Iwama, Toshihide Yokoyama, Yasutaka Watanabe, Hiroshi Tanaka, Yutaka Fujiwara, Yoshitaka Zenke, Koichi Azuma, Hirokazu Taniguchi, Ryo Toyozawa, Yukio Hosomi, Haruyasu Murakami, Satoshi Hara, Akihiro Bessho, Nobuyuki Yamamoto, Yuichiro Ohe

**Affiliations:** 1 Shinshu Cancer Center, Shinshu University Hospital, Matsumoto, Japan.; 2 Department of Pulmonary Medicine and Clinical Immunology, Dokkyo Medical University, Mibu, Japan.; 3 Department of Thoracic Oncology, Kansai Medical University Hospital, Osaka, Japan.; 4 Department of Biostatistics and Bioinformatics, Graduate School of Medicine, The University of Tokyo, Tokyo, Japan.; 5 Department of Pulmonary Medicine, Sendai Kousei Hospital, Sendai, Japan.; 6 Department of Respiratory Medicine, Graduate School of Medical Sciences, Kyushu University, Fukuoka, Japan.; 7 Department of Respiratory Medicine, Kurashiki Central Hospital, Kurashiki, Japan.; 8 Department of Thoracic Oncology, Saitama Cancer Center, Saitama, Japan.; 9 Department of Internal Medicine, Niigata Cancer Center Hospital, Niigata, Japan.; 10 Department of Thoracic Oncology, Aichi Cancer Center, Nagoya, Japan.; 11 Department of Thoracic Oncology, National Cancer Center Hospital East, Kashiwa, Japan.; 12 Division of Respirology, Neurology, and Rheumatology, Department of Internal Medicine, Kurume University School of Medicine, Kurume-Shi, Japan.; 13 Department of Respiratory Medicine, Nagasaki University Graduate School of Biomedical Sciences, Nagasaki, Japan.; 14 Department of Thoracic Oncology, National Hospital Organization Kyushu Cancer Center, Fukuoka, Japan.; 15 Department of Thoracic Oncology and Respiratory Medicine, Tokyo Metropolitan Cancer and Infectious Diseases Center Komagome Hospital, Tokyo, Japan.; 16 Division of Thoracic Oncology, Shizuoka Cancer Center, Shizuoka, Japan.; 17 Department of Respiratory Medicine, Itami City Hospital, Itami, Japan.; 18 Department of Respiratory Medicine, Japanese Red Cross Okayama Hospital, Okayama, Japan.; 19 Internal Medicine III, Wakayama Medical University, Wakayama, Japan.; 20 Department of Thoracic Oncology, National Cancer Center Hospital, Tokyo, Japan.

## Abstract

**Purpose:**

This study was performed to confirm the superiority in overall survival (OS) of EGFR tyrosine kinase inhibitor (TKI gefitinib or osimertinib) monotherapy versus EGFR TKI with intercalation of cisplatin plus pemetrexed as the first-line treatment for patients with advanced non-squamous non–small cell lung cancer (NSqNSCLC) harboring *EGFR* mutation.

**Patients and Methods:**

This was an open-label, multicenter, randomized phase III study. Patients with chemotherapy-naïve advanced or recurrent NSqNSCLC harboring *EGFR* mutation (exon 19 deletion or exon 21 L858R point mutation) were randomly assigned (1:1) to EGFR-TKI monotherapy or the EGFR TKI plus intercalated chemotherapy group. The primary endpoint was OS, and the secondary endpoints included progression-free survival (PFS).

**Results:**

From December 2015 to October 2020, 501 patients were randomized. The EGFR TKI was changed from gefitinib to osimertinib in October 2018 (gefitinib cohort: *n* = 308 and osimertinib cohort: *n* = 193). There was no survival advantage in the EGFR TKI plus intercalated chemotherapy group; the median survival time of both groups was 48.0 months (HR, 0.985; 91.4% confidence interval, 0.796–1.219; one-sided *P* = 0.4496). The median PFS time was 12.0 months in the EGFR-TKI monotherapy group and 18.0 months in the EGFR TKI plus intercalated chemotherapy group (HR, 0.762; 95% confidence interval, 0.628–0.925; one-sided *P* = 0.003). The OS and PFS trends in both gefitinib and osimertinib cohorts were identical to those in the entire population.

**Conclusions:**

The intercalation of cisplatin plus pemetrexed after the response to EGFR TKI improved PFS but not OS compared with EGFR TKI monotherapy as the first-line treatment for patients with advanced NSqNSCLC harboring *EGFR* mutation.

**Translational Relevance**

Preventing and overcoming acquired resistance to EGFR tyrosine kinase inhibitors (TKI) are important issues for improving the prognosis of patients with advanced non-squamous non–small cell lung cancer harboring *EGFR* mutation. We hypothesized that the intercalation of platinum-doublet chemotherapy during the initial response to EGFR TKI might prevent the emergence of acquired resistance to the EGFR TKI, and prolong patient survival, and performed this study to confirm the superiority in overall survival of EGFR TKI (gefitinib or osimertinib) monotherapy versus EGFR TKI with intercalation of cisplatin plus pemetrexed as the first-line treatment for patients with advanced non-squamous non–small cell lung cancer harboring *EGFR* mutation. This study demonstrated that the intercalation of platinum-doublet chemotherapy after the response to EGFR TKI improved progression-free survival but not overall survival compared with EGFR-TKI monotherapy and showed that EGFR TKI plus intercalating chemotherapy could not prevent the acquired resistance enough and might be less effective than the concurrent combination of EGFR TKI and chemotherapy.

## Introduction

The core drug for the first-line treatment of patients with advanced non-squamous non–small cell lung cancer (NSqNSCLC) harboring *EGFR*-activating mutation is an EGFR tyrosine kinase inhibitor (TKI). Osimertinib is a selective irreversible inhibitor of *EGFR*-activating mutation and *EGFR*^T790M^ point mutation, of which the latter causes resistance to previous-generation EGFR TKIs. Osimertinib is considered the first-choice EGFR TKI, considering the findings of the FLAURA study, which compared osimertinib with previous-generation EGFR TKIs (1, 2). However, acquired resistance to osimertinib occurred in almost all patients, limiting the duration of response and patient survival. The mechanism of resistance to EGFR TKIs, especially to osimertinib (3), was not fully understood. Preventing and overcoming the acquired resistances to EGFR TKIs are important issues for improving survival of these patients.

In the first-line setting, the combination of platinum-doublet chemotherapy with an EGFR TKI may prolong the survival of patients with advanced NSqNSCLC harboring *EGFR* mutation. A Japanese multicenter phase III study (the NEJ009 study) showed the superiority of concurrent combination therapy of gefitinib and platinum-doublet chemotherapy over gefitinib monotherapy in terms of progression-free survival (PFS) without a statistically significant gain in overall survival (OS; refs. 4, 5). By contrast, a single-center phase III study in India assessed the same regimens and reported the superiority of the combination therapy with respect to both PFS and OS (6). Recently, the FLAURA2 study demonstrated the superiority of concurrent combination therapy of osimertinib and platinum-doublet chemotherapy over osimertinib monotherapy in terms of PFS (7), but the OS data are still immature to evaluate.

Although the above several studies indicated the benefits of concurrent combination therapy of EGFR TKI and platinum-doublet chemotherapy, there is not enough evidence to suggest that the concurrent combination is the most promising in the combination methods. Several preclinical studies using wild-type *EGFR* cancer cells suggested that in the setting of concurrent combination therapy, EGFR TKI might attenuate the effect of cytotoxic agents, arresting the cell cycle at the G_1_ phase (8, 9) and inhibiting caspase-independent cell death by cisplatin (10). Additionally, in two phase III studies—INTACT1 (11) and INTACT2 (12)—comparing platinum-doublet chemotherapy and concurrent combination therapy comprising gefitinib and platinum-doublet chemotherapy, the concurrent combination therapy not only failed to demonstrate superiority over platinum-doublet chemotherapy but was also associated with slightly lower survival than chemotherapy. These findings may indicate the disadvantage of concurrent combination of EGFR TKI and platinum-doublet chemotherapy.

We hypothesized that the intercalation of platinum-doublet chemotherapy during the initial response to EGFR TKI might prevent the emergence of acquired resistance to the EGFR TKI and prolong patient survival. In addition, platinum-doublet chemotherapy itself might be more efficient after the initial tumor response to the EGFR TKI has been established because the tumor burden is reduced by the EGFR TKI (Supplementary Fig. S1). We previously conducted a single-group, single-center phase II study of gefitinib with intercalation of cisplatin plus docetaxel as first-line treatment in patients with advanced NSqNSCLC harboring *EGFR* mutation. The experimental treatment resulted in good PFS (median of 19.5 months) and good OS (median of 48.0 months; ref. 13). Considering the results of this phase II study, we conducted a phase III study to confirm our hypothesis.

## Patients and Methods

### Study design and treatment

This JCOG1404/WJOG8214L study (UMIN000020242) was an open-label, multicenter, randomized phase III study comparing two groups of treatment-naïve patients with advanced or recurrent NSqNSCLC harboring *EGFR* activation mutation at 75 institutions belonging to the Japan Clinical Oncology Group (JCOG) and West Japan Oncology Group in Japan. The patients were randomly assigned to receive EGFR-TKI monotherapy or EGFR TKI plus intercalated chemotherapy in a 1:1 ratio. In the EGFR-TKI monotherapy group, an EGFR TKI (gefitinib 250 mg once daily or osimertinib 80 mg once daily) was administered until disease progression occurred. In the EGFR TKI plus intercalated chemotherapy group, an EGFR TKI (gefitinib 250 mg once daily or osimertinib 80 mg once daily) was administered on days 1 to 56. Then, after a 2-week drug-free period, three cycles of cisplatin (75 mg/m^2^) and pemetrexed (500 mg/m^2^) were administered on days 71, 92, and 113. The EGFR TKI was thereafter reinitiated on day 134 and continued until disease progression occurred (Supplementary Fig. S2). We established a 2-week drug-free period after the first 8 weeks of administration of EGFR TKI to eliminate the negative effects expected from the concurrent use of EGFR TKI and platinum-doublet chemotherapy as shown in the preclinical studies described above. In addition, we chose three cycles of platinum-doublet chemotherapy given a clinical study showing the equivalence of the OS and the milder toxicities of three or four cycles compared with six cycles of platinum-doublet chemotherapy in patients with advanced NSCLC (14).

This study was performed in accordance with the Declaration of Helsinki and Japanese Ethical Guidelines for Medical and Health Research Involving Human Subjects. The study protocol (Supplementary Data S1) and amendments were approved by the institutional review board at each site. All the patients provided written informed consent.

### Participants

This study enrolled patients with NSqNSCLC confirmed histologically or cytologically; stage IIIB/IV or postoperative recurrence; *EGFR*-activating mutation (exon 19 deletion or exon 21 L858R point mutation); Eastern Clinical Oncology Group performance status of 0 or 1; age of 20 to 74 years; and no prior systemic treatment. Patients with stable central nervous system metastasis were included. All patients provided written informed consent before receiving the study treatment. All *EGFR* mutations were proven by a single-plex PCR assay [such as cobas EGFR Mutation Test (Roche) or therascreen EGFR RGQ PCR Kit (Qiagen)] or a multiplex next-generation sequencing assay [such as Oncomine Dx Target Test (Thermo Fisher Scientific)]. Data about PD-L1 status were not collected in this study.

The enrolled patients were randomly assigned in a 1:1 ratio to the EGFR-TKI monotherapy group or the EGFR TKI plus intercalated chemotherapy group and adjusted by institution, stage IIIB/IV or recurrence, sex, and *EGFR* exon 19 deletion or exon 21 L858R point mutation.

### Study endpoints

The primary endpoint was OS, defined as the duration of time from randomization to death due to any cause. The purpose of this study was to confirm the superiority of EGFR TKI plus intercalated chemotherapy over EGFR-TKI monotherapy with respect to OS. The secondary endpoints were PFS (duration of time from randomization to disease progression or death due to any cause), objective response rate (ORR; proportion of patients with a *partial* or *complete* response to the study treatment), adverse events (AE), severe AEs, and proportion of patients with *EGFR*^T790M^ positivity upon the development of progressive disease. PFS and tumor responses were assessed by investigators.

Tumor responses were assessed according to RECIST version 1.1. AEs were graded according to the NCI Common Terminology Criteria for Adverse Events version 4.0.

### Statistical analysis

The backbone EGFR TKI in both groups was gefitinib only in the original protocol (version 1.0, finalized in October 2015) because the label of osimertinib in Japan was revised to include first-line usage in August 2018, driven by the results of the FLAURA study. The original protocol required a total of 500 patients (297 deaths) to detect an HR for mortality of 0.749 with a one-sided α level of 5% and power of 80%. Assuming a 45% 3-year survival rate for the EGFR-TKI monotherapy group and a 10% increase for the EGFR TKI plus intercalated chemotherapy group, the HR would be 0.749. The amended protocol (version 2.0) was adopted in October 2018 to change the backbone EGFR TKI in both groups from gefitinib to osimertinib. The same HR of 0.749 was expected, corresponding to a 10.0% or 6.7% gain in 3-year survival of the gefitinib or osimertinib cohort (45.0% and 68.9%). Assuming a total of 326 patients would be enrolled in the gefitinib cohort until Japanese public insurance reimbursed osimertinib as first-line treatment, the amended protocol required 257 deaths (500 patients) to detect an HR of 0.749 with a one-sided α level of 5% and power of 75% in an intention-to-treat (ITT) population.

We performed two interim analyses and the final analysis. The first interim analysis was based on 13 deaths only; thus, only a futility termination was considered. The second interim analysis and the final analysis were based on 192 and 261 deaths, respectively. The Lan–DeMets O’Brien–Fleming alpha spending function was used to adjust multiplicity because of repeated testing. The multiplicity-adjusted one-sided α level for OS in the final analysis was 4.32%.

Efficacy endpoints (OS, PFS, and ORR) were analyzed for all randomized patients on an ITT basis. The primary analysis for OS was performed by a stratified log-rank test. For all stratified analyses, adjustment factors included sex, *EGFR* mutational status at randomization, and the use of EGFR TKIs (gefitinib or osimertinib). Cox proportional hazards models were used to estimate the HR. The ORR and proportion of *EGFR*^T790M^ positivity were compared using the Fisher exact test. All *P* values except those in the primary analysis were reported as two-sided. All collected data were analyzed using SAS version 9.4 software (SAS Institute, RRID: SCR_008567). Further details of the statistical analyses are provided in the study protocol.

### Data availability

Individual participant data that underlie the results reported in this article, after deidentification, will be shared if investigators whose proposed use of the data has been approved by the investigators from the Lung Cancer Study Group of JCOG identified for this purpose. Proposals should be directed to skanda@shinshu-u.ac.jp. The data will be made available to achieve the aims specified in the approved proposal.

## Results

### Participants

**Figure 1.**
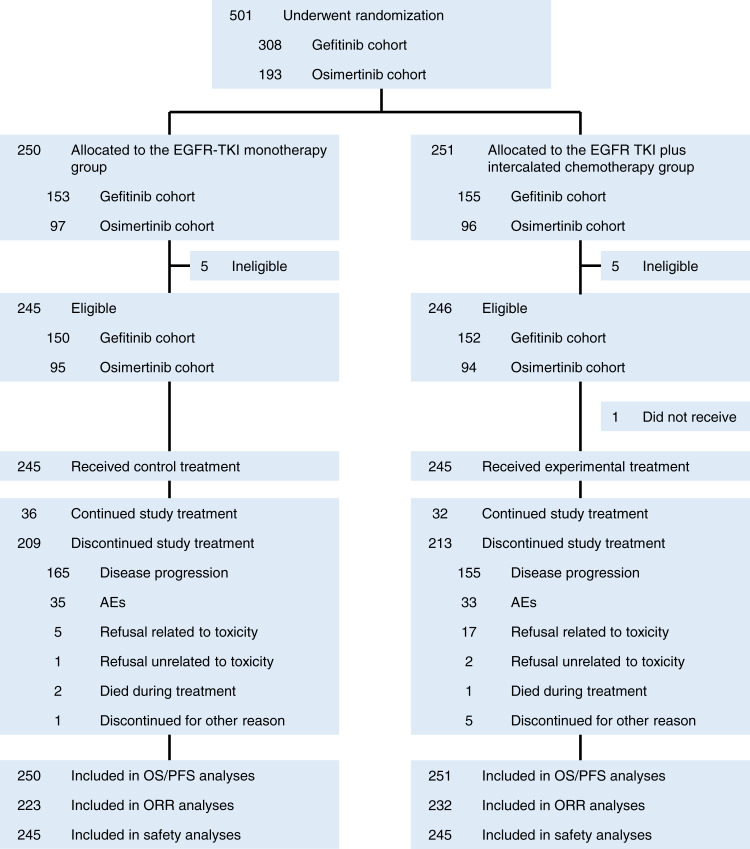
CONSORT diagram. Flowchart of patient disposition throughout the study, including randomization, eligibility, treatment, and follow-up.

From December 2015 to October 2020, 501 patients (gefitinib cohort: 308 patients and osimertinib cohort: 193 patients) were randomized. The patients’ median age was 65 years. A total of 53% had an Eastern Clinical Oncology Group performance status of 1, 59% were male, 86% had advanced-stage cancer, and 56% had exon 19 deletion. Additionally, 28% of the patients had stable central nervous system metastasis (Table 1; Supplementary Table S1). The data cutoff date was November 2022. The median follow-up time for all enrolled patients was 36.0 months. Of all 501 enrolled patients, 491 were eligible and 490 received the study treatment. Treatment discontinuation is summarized in the CONSORT diagram (Fig. 1).

**Table 1.** Patient characteristics. Data are presented as median (range) or *n* (%).

Characteristic		EGFR-TKI monotherapy (*n* = 250)	EGFR TKI plus intercalated chemotherapy (*n* = 251)	Total (*n* = 501)
Age, years		65 (38–74)	65 (26–74)	65 (26–74)
ECOG PS	0	126 (50.4%)	110 (43.8%)	236 (47.1%)
	1	124 (49.6%)	141 (56.2%)	265 (52.9%)
Sex	Male	101 (40.4%)	104 (41.4%)	205 (40.9%)
	Female	149 (59.6%)	147 (58.6%)	296 (59.1%)
Clinical stage	IIIB/IV	213 (85.2%)	216 (86.1%)	429 (85.6%)
	Recurrence	37 (14.8%)	35 (13.9%)	72 (14.4%)
*EGFR* mutation	Exon 19 DEL	138 (55.2%)	141 (56.2%)	279 (55.7%)
	Exon 21 L858R	112 (44.8%)	110 (43.8%)	222 (44.4%)
CNS metastasis		74 (29.6%)	68 (27.1%)	142 (28.3%)
EGFR TKI	Gefitinib	153 (61.2%)	155 (61.8%)	308 (61.5%)
	Osimertinib	97 (38.8%)	96 (38.2%)	193 (38.5%)

Abbreviations: CNS, central nervous system; ECOG, Eastern Clinical Oncology Group; Exon 19 DEL, EGFR exon 19 deletion; Exon 21 L858R, EGFR exon 21 L858 point mutation; PS, performance status.

### OS

**Figure 2.**
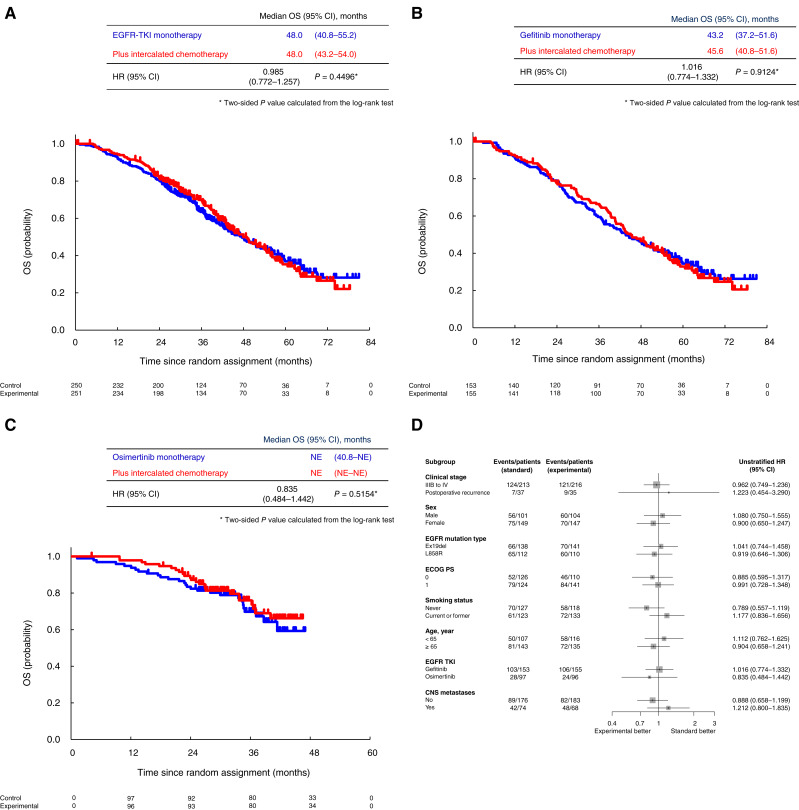
Kaplan–Meier analysis of OS. **A,** ITT population. The 1-, 2-, 3-, 4-, and 5-year survival rates were 92.8%, 80.4%, 63.5%, 49.6%, and 36.9% in the control group and 94.4%, 81.7%, 69.9%, 49.9%, and 35.3% in the experimental group, respectively. **B, **Gefitinib cohort. The 1-, 2-, 3-, 4-, and 5-year survival rates were 91.5%, 78.4%, 59.5%, 46.3%, and 34.4% in the control group and 92.2%, 77.7%, 66.4%, 46.5%, and 32.9% in the experimental group, respectively. **C, **Osimertinib cohort. The 1-, 2-, and 3-year survival rates were 94.8%, 83.4%, and 69.8% in the control group and 97.9%, 88.3%, and 76.0% in the experimental group, respectively. **D, **Subgroup analysis of OS. ECOG, Eastern Clinical Oncology Group; Ex19del, EGFR exon 19 deletion; L858R, EGFR exon 21 L858 point mutation; NE; not estimable; PS, performance status.

The superiority of OS was not demonstrated in the ITT population. The median OS was 48.0 months [95% confidence interval (CI), 40.8–55.2] in the EGFR-TKI monotherapy group and 48.0 months (95% CI, 43.2–54.0) in the EGFR TKI plus intercalated chemotherapy group (HR = 0.976; 91.4% CI, 0.796–1.219; one-sided *P* = 0.4496; Fig. 2A). In the gefitinib cohort, the median survival time was 43.2 months (95% CI, 37.2–51.6) in the EGFR-TKI monotherapy group and 45.6 months (95% CI, 40.8–51.6) in the EGFR TKI plus intercalated chemotherapy group (HR = 1.016; 95% CI, 0.774–1.332; *P* = 0.9124; Fig. 2B). In the osimertinib cohort, the median survival time could not be estimated in either the EGFR-TKI monotherapy or the EGFR TKI plus intercalated chemotherapy group (HR = 0.835; 95% CI, 0.484–1.442; *P* = 0.5154; Fig. 2C). The subgroup analysis showed no favorable subgroup for the EGFR TKI plus intercalated chemotherapy group (Fig. 2D; Supplementary Fig. S3).

### PFS and the other secondary endpoints

**Figure 3.**
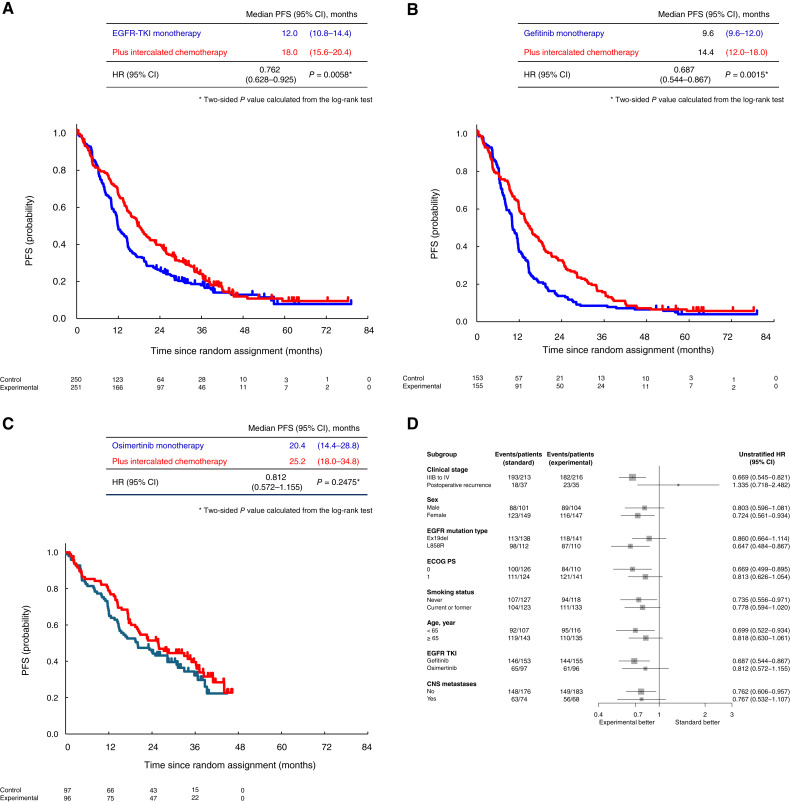
Kaplan–Meier analysis of PFS. **A,** ITT population. The 1-, 2-, 3-, 4-, and 5-year PFS rates were 49.2%, 26.0%, 18.0%, 12.9%, and 7.8% in the control group and 67.0%, 39.8%, 24.1%, 11.9%, and 9.5% in the experimental group, respectively. **B,** Gefitinib cohort. The 1-, 2-, 3-, 4-, and 5-year PFS rates were 37.3%, 13.7%, 8.5%, 6.5%, and 4.0% in the control group and 59.5%, 32.7%, 15.7%, 7.2%, and 5.7% in the experimental group, respectively. **C,** Osimertinib cohort. The 1-, 2-, and 3-year PFS rates were 68.0%, 45.3%, and 32.2% in the control group and 79.0%, 51.4%, and 39.6% in the experimental group, respectively. **D, **Subgroup analysis of PFS. ECOG, Eastern Clinical Oncology Group; Ex19del, EGFR exon 19 deletion; L858R, EGFR exon 21 L858 point mutation; PS, performance status.

In the ITT population, the median PFS was 12.0 months (95% CI, 10.8–14.4) in the EGFR-TKI monotherapy group and 18.0 months (95% CI, 15.6–20.4) in the EGFR TKI plus intercalated chemotherapy group (HR = 0.762; 95% CI, 0.628–0.925; *P* = 0.0003; Fig. 3A). In the gefitinib cohort, the median PFS was 9.6 months (95% CI, 9.6–12.0) in the EGFR-TKI monotherapy group and 14.4 months (95% CI, 12.0–18.0) in the EGFR TKI plus intercalated chemotherapy group (HR = 0.687; 95% CI, 0.544–0.867; *P* = 0.0015; Fig. 3B). In the osimertinib cohort, the median PFS was 20.4 months (95% CI, 14.4–28.8) in the EGFR-TKI monotherapy group and 25.2 months (95% CI, 18.0–34.8) in the EGFR TKI plus intercalated chemotherapy group (HR = 0.812; 95% CI, 0.572–1.155; *P* = 0.2475; Fig. 3C). In the subgroup analysis, the EGFR TKI plus intercalated chemotherapy group showed a lower HR for PFS in the exon 21 L858R point mutation subgroup than in the exon 19 deletion subgroup (Fig. 3D). In the gefitinib cohort, the EGFR TKI plus intercalated chemotherapy group showed a similar HR for PFS in both the exon 21 L858R point mutation subgroup and exon 19 deletion subgroup (Supplementary Fig. S4A). In the osimertinib cohort, the EGFR TKI plus intercalated chemotherapy group showed a better PFS in the exon 21 L858R point mutation subgroup but not in the exon 19 deletion subgroup (Supplementary Fig. S4B).

The ORRs of the EGFR-TKI monotherapy group and the EGFR TKI plus intercalated chemotherapy group were 78.0% (95% CI, 72.0%–83.3%) and 71.6% (95% CI, 65.3%–77.3%), respectively, in the ITT population (*P* = 0.1309). In the gefitinib cohort, the ORRs were 76.4% and 69.0%. In the osimertinib cohort, the ORRs were 80.7% and 75.6%. In the subgroup harboring exon 19 deletion, the ORRs were 84.7% and 75.2%. In the subgroup harboring exon 21 L858R point mutation, the ORRs were 69.7% and 67.0%, respectively.

In the gefitinib cohort, biopsies at the time of progressive disease were performed in 152 patients. The proportion of *EGFR*^T790M^ point mutation positivity at progressive disease was 41% in the EGFR-TKI monotherapy group and 49% in the EGFR TKI plus intercalated chemotherapy group (*P* = 0.3284).

### Safety

Unexpected AEs were not observed although AEs caused by cisplatin plus pemetrexed occurred in the EGFR TKI plus intercalated chemotherapy group (Table 2). Pneumonitis was observed in 1.0% of the gefitinib cohort (1.3% of the EGFR-TKI monotherapy group and 0.7% of the EGFR TKI plus intercalated chemotherapy group) and 7.9% of the osimertinib cohort (10.5% of the EGFR-TKI monotherapy group and 5.3% of the EGFR TKI plus intercalated chemotherapy group). Severe pneumonitis (grade ≥3) was observed in only one patient in the EGFR-TKI monotherapy group of the osimertinib cohort. Treatment-related death occurred in one patient in the EGFR-TKI monotherapy group of the osimertinib cohort. This death was due to cerebral infarction and worsening respiratory failure triggered by *Mycoplasma* infection, and causality of the protocol treatment could not be denied.

**Table 2.** Subsequent treatment.

CTCAE ver4.0	EGFR-TKI monotherapy (*n* = 250)				EGFR TKI plus intercalated chemotherapy (*n* = 251)			
	Gefitinib (*n* = 153)		Osimertinib (*n* = 97)		Gefitinib (*n* = 155)		Osimertinib (*n* = 96)	
	Any (%)	G3 to 4 (%)	Any (%)	G3 to 4 (%)	Any (%)	G3 to 4 (%)	Any (%)	G3 to 4 (%)
Decreased neutrophil count	22.0	1.3	36.8	3.2	65.6	11.3	70.2	18.1
Decreased platelet count	22.7	0.0	49.5	1.1	47.0	0.7	71.3	2.1
Increased AST	82.0	15.3	43.2	3.2	80.1	15.2	69.1	3.2
Increased ALT	80.7	26.7	44.2	4.2	83.4	27.8	72.3	8.5
Increased creatitine	28.0	0.0	35.8	0.0	36.4	0.0	53.2	1.1
Nausea	8.7	0.0	9.5	0.0	50.3	2.6	45.7	2.1
Anorexia	13.3	2.0	9.5	0.0	51.7	6.0	45.7	2.1
Diarrhea	38.7	2.0	52.6	0.0	41.1	0.7	44.7	1.1
Acneiform rash	82.0	2.0	58.9	2.1	80.8	1.3	58.5	1.1
Paronychia	38.7	2.0	44.2	1.1	23.2	0.7	40.4	0.0
Prolonged QTc on ECG	—	—	6.3	0.0	—	—	10.6	1.1
Pneumonitis	1.3	0.0	10.5	1.1	0.7	0.7	5.3	0.0
AE leading to discontinuation (%)	14.4		11.3		8.4		14.6	
Treatment-related death (%)	0.0		1.0		0.0		0.0	

Abbreviations: ALT, alanine aminotransferase; AST, aspartate aminotransferase; G3 to 4, grade 3 to 4.

### Subsequent treatment

In total, 80% of the patients in both groups received subsequent treatment including EGFR TKI beyond progressive disease, and 65% of the EGFR-TKI monotherapy group and 56% of the EGFR TKI plus intercalated chemotherapy group received a second subsequent treatment. In the EGFR-TKI monotherapy group, platinum-doublet chemotherapies were used in approximately 60% of the gefitinib cohort patients and approximately 50% of the osimertinib cohort patients. In the gefitinib cohort, 43% of the EGFR-TKI monotherapy group and 41% of the EGFR TKI plus intercalated chemotherapy group were administered a third-generation EGFR TKI as the first or second subsequent treatment (Table 3).

**Table 3.** Safety summary.

	EGFR-TKI monotherapy						EGFR TKI plus intercalated chemotherapy						
	All *n* = 250 (%)		Gefitinib *n* = 153 (%)		Osimertinib *n* = 97 (%)		All *n* = 251 (%)		Gefitinib *n* = 155 (%)		Osimertinib *n* = 96 (%)		
Continuing study treatment	36	(14)	5	(3)	31	(32)	32	(13)	5	(3)	27	(28)	
Discontinued study treatment	214	(86)	148	(97)	66	(68)	219	(87)	150	(97)	69	(72)	
Received first subsequent treatment	200	(80)	139	(91)	61	(63)	204	(81)	139	(90)	65	(68)	
Received second subsequent treatment	161	(64)	117	(76)	44	(45)	142	(57)	102	(66)	40	(42)	
Received first subsequent treatment	200		139		61		204		139		65		
Third-generation EGFR TKI	61^a^	(30)	29	(21)	32^a^	(52)	68	(33)	25	(18)	43^a^	(66)	
The other EGFR TKI	85^a^	(43)	78^a^	(56)	7	(11)	102	(50)	97^a^	(70)	5	(8)	
Platinum-based therapy	50	(25)	29	(21)	21	(34)	23	(11)	11	(8)	12	(18)	
The other chemotherapy	4	(2)	3	(2)	1	(2)	11	(6)	6	(4)	5	(8)	
Received second subsequent treatment	161		117		44		142		102		40		
Third-generation EGFR TKI	41	(25)	37	(32)	4	(9)	40	(28)	39	(38)	1	(3)	
The other EGFR TKI	14	(9)	8	(7)	6	(14)	22	(16)	18	(17)	4	(10)	
Platinum-based therapy	84	(52)	60	(51)	24	(55)	44	(31)	22	(22)	22	(55)	
The other chemotherapy	22	(14)	12	(10)	10	(22)	36	(25)	23	(23)	13	(32)	

a Including gefitinib or osimertinib beyond progressive disease.

## Discussion

This JCOG1404/WJOG8214L study was one of the few phase III studies that attempted to confirm the efficacy of the combination of EGFR TKI including osimertinib and platinum-doublet chemotherapy in patients with advanced NSqNSCLC harboring *EGFR*-activating mutation in improving OS, although the efficacy in the improvement of OS was not confirmed. The intercalation of cisplatin plus pemetrexed after the initial response to EGFR TKI improved PFS but not OS compared with EGFR-TKI monotherapy. PFS was better in the EGFR TKI plus intercalated chemotherapy group than in the EGFR-TKI monotherapy group by a median of 5 to 6 months. The prolongation of PFS with intercalated chemotherapy was statistically significant in the gefitinib cohort but not in the osimertinib cohort. In the L858R point mutation subgroup, the trends of longer PFS with intercalated chemotherapy were observed across EGFR TKIs; in the exon 19 deletion subgroup, longer PFS with intercalated chemotherapy was observed only in the gefitinib cohort and not in the osimertinib cohort. In cancer with *EGFR* exon 19 deletion, the disadvantages of temporarily discontinuing osimertinib might be significant. The probability of longer PFS was not increased by the experimental treatment. It seemed that intercalation of platinum-doublet chemotherapy exhibited temporal disease control efficacy but not the expected long-term efficacy by overcoming the resistance mechanism. Although the OS was not prolonged in the EGFR TKI plus intercalated chemotherapy group compared with that in the EGFR-TKI monotherapy group, the median survival time in both groups was extremely favorable. We reasoned that the modest prolongation of PFS in the EGFR TKI plus intercalated chemotherapy group was not reflected in OS because of the effects of the intensive subsequent treatments.

An international open-label randomized phase III study (FLAURA2) demonstrated that concurrent combination treatment comprising osimertinib and platinum plus pemetrexed with pemetrexed maintenance therapy improved the PFS compared with osimertinib monotherapy in patients with advanced NSCLC harboring *EGFR* mutation (HR = 0.62; 95% CI, 0.49–0.79; *P* < 0.0001; median PFS was 16.7 months in the osimertinib monotherapy and 25.5 months in the concurrent combination), although the OS is still immature at the second interim analysis (HR = 0.75; 95% CI, 0.57–0.97; *P* = 0.0280, the significance level in the second interim analysis was set at *P* ≤ 0.000001; ref. 13). On the other hand, a single-center phase III study in India comparing gefitinib monotherapy and concurrent combination treatment comprising gefitinib and carboplatin plus pemetrexed confirmed the superiority of concurrent combination treatment with respect to not only PFS but also OS. In that study, the proportion of patients who received the subsequent treatment was low (platinum-doublet chemotherapy was administered to 3% of the gefitinib group and 9% of the gefitinib plus chemotherapy group). To determine the efficacy of the combination treatment of EGFR TKI and platinum-doublet chemotherapy as the first-line treatment, under the situation that adequate subsequent treatments are provided, the prolongation of OS should be evaluated. The OS result of the FLAURA2 study is awaited. On the other hand, an international open-label randomized phase III study (MARIPOSA) demonstrated the superiority of amivantamab plus lazertinib over osimertinib monotherapy in PFS. The OS results of the FLAURA2 study and MARIPOSA study are awaited to determine the optimal first-line treatment for patients with NSqNSCLC harboring *EGFR* mutation.

The current study might not have maximized the efficacy of the experimental therapy to prove the hypothesis for the following reasons. First, we configured a schedule consisting of 8 weeks of EGFR TKI followed by 2 weeks of rest and cisplatin plus pemetrexed chemotherapy with the expectation that chemotherapy would be administered at the time of maximum EGFR-TKI antitumor efficacy. However, this first 8-week administration of EGFR TKI may have been too short to achieve the maximum EGFR-TKI antitumor efficacy because the ORR tended to be lower in the EGFR TKI plus intercalated chemotherapy group than the EGFR-TKI monotherapy group. The ORR of the EGFR TKI plus intercalated chemotherapy group in the exon 19 deletion subgroup was approximately 10% lower than that of the EGFR-TKI monotherapy group, although the difference in the L858R point mutation subgroup was 2%. This difference in ORR between the groups may have influenced the worse PFS HR in the exon 19 deletion subgroup than in the exon 21 L858R point mutation subgroup. In other words, the first 8-week administration of EGFR TKI may have been too short to achieve the maximum EGFR-TKI efficacy, especially in the exon 19 deletion subgroup. Perhaps the platinum-doublet chemotherapy should have been given at the point of maximum response to the EGFR TKI instead of limiting it to 8 weeks. Additionally, in the exon 19 deletion subgroup (in which dependence on the EGFR pathway was considered stronger than that in the L858R point mutation subgroup), the antitumor efficacy might have been attenuated by the 2-week rest that followed the EGFR TKI and preceded the intercalation of cisplatin plus pemetrexed. Second, cisplatin plus pemetrexed might not be the most promising chemotherapy for NSqNSCLC harboring *EGFR* mutation. A phase III study comparing cisplatin plus pemetrexed and cisplatin plus vinorelbine for resected stage II to IIIA NSqNSCLC showed that relapse-free survival with cisplatin plus pemetrexed was worse than that with cisplatin plus vinorelbine in patients harboring *EGFR* mutation (15, 16).

### Conclusion

In patients with advanced or recurrent NSqNSCLC harboring *EGFR*-activating mutation, the intercalation of cisplatin plus pemetrexed after the initial response to an EGFR TKI (gefitinib or osimertinib) did not prolong OS compared with EGFR-TKI monotherapy (gefitinib or osimertinib), although it prolonged PFS. EGFR TKI plus intercalating platinum-doublet chemotherapy might be less effective than the concurrent combination therapy of EGFR TKI and platinum-doublet chemotherapy. The mechanisms underlying the acquired resistance to osimertinib are diverse and remain incompletely understood (17, 18). We are investigating the mechanisms of acquired resistance to osimertinib and the study treatment using tumor tissue and liquid biopsy samples.

## Supplementary Material

Supplementary Table S1 Supplementary Table S1. Representativeness of study participants.

Supplementary Figure S1 Supplementary Figure S1. Study hypothesis. Abbreviations: EGFR; epidermal growth factor receptor; EGFR-TKI, epidermal growth factor receptor tyrosine kinase inhibitor.

Supplementary Figure S2 Supplementary Figure S2. Study design of JCOG1404/WJOG8214L. Abbreviations: NSCLC, non-small cell lung cancer; EGFR; epidermal growth factor receptor; Exon 19 DEL, EGFR exon 19 deletion; Exon 21 L858R, EGFR exon 21 L858 point mutation; ECOG, Eastern Clinical Oncology Group; PS, performance status; CNS, central nervous system; EGFR-TKI, epidermal growth factor receptor tyrosine kinase inhibitor; PD, progressive disease.

Supplementary Figure S3 Supplementary Figure S3. Subgroup analyses of overall survival. (A) Gefitinib cohort. (B) Osimertinib cohort. Abbreviations: CI, confidence interval; EGFR, epidermal growth factor receptor; Ex19del, EGFR exon 19 deletion; L858R, EGFR exon 21 L858 point mutation; ECOG, Eastern Clinical Oncology group; PS, performance status; EGFR-TKI, epidermal growth factor receptor tyrosine kinase inhibitor; CNS, central nervous system.

Supplementary Figure S4 Supplementary Figure S4. Subgroup analyses of progression-free survival. (A) Gefitinib cohort. (B) Osimertinib cohort. Abbreviations: CI, confidence interval; EGFR, epidermal growth factor receptor; Ex19del, EGFR exon 19 deletion; L858R, EGFR exon 21 L858 point mutation; ECOG, Eastern Clinical Oncology group; PS, performance status; EGFR-TKI, epidermal growth factor receptor tyrosine kinase inhibitor; CNS, central nervous system.

Supplementary Data S1 Supplementary Data S1. Study Protocol of JCOG1404/WJGO8214L.
