# Supplementary material for: Randomized Phase III Study of EGFR Tyrosine Kinase Inhibitor and Intercalated Platinum-Doublet Chemotherapy for Non–Small Cell Lung Cancer Harboring EGFR Mutation
Source: Clin Cancer Res. 2025 Mar 31;31(12):2317–26. doi: 10.1158/1078-0432.CCR-24-3532 (PMC12163600; doi:10.1158/1078-0432.CCR-24-3532)
Supplement: Supplementary Table S1 — Representativeness of study participants. [file ccr-24-3532_supplementary_table_s1_suppst1.docx]

**Supplementary Table S1.** Representativeness of study participants.

| Cancer type(s)/subtype(s)/stage(s)/condition | Advanced or recurrent non-squamous non-small cell Lung Cancer harboring *EGFR* mutation |
| --- | --- |
| Considerations related to: |  |
| Sex | The prevalence of *EGFR* mutation is higher in females than males. A large systematic review and meta-analysis including 456 studies reported that the pooled prevalences (including data obtained from 322–331 studies) of *EGFR* mutation were 43.7% in females and 24.0% in males^1^. |
| Age | The median age of patients included in the systematic review and meta-analysis cited above was 63 years^1^. |
| Race/ethnicity | The systematic review and meta-analysis cited above reported that the prevalences of *EGFR* mutation were 38.8% in Asian  population, 17.4% in Caucasians, 17.2% in African Americans, and 27.0% in mixed populations^1^. |
| Geography | The systematic review and meta-analysis cited above reported that region the prevalences of *EGFR* mutation were 38.4% in Asia, 24.4% in North and South America and 14.1% in Europe^1^. The other study using the data of National Cancer Center Hospital, Japan (319 Japanese patients) and TCGA (The Cancer Genome Atlas) study (230 US patients) reported that the prevalences of *EGFR* mutation were 53.0% in Japan and 11.3% in US^2^. |
| Other considerations | *EGFR* mutations are more frequent in female, Asians, non-smoker and patient with adenocarcinoma^1^. |
| Overall representativeness of this study | The patients enrolled in this study were all Japanese. Median age of the patients was 65 years, and it was consistent with that of the systematic review and meta-analysis cited above. In this study, more female (60%) than male (40%) were enrolled, which was consistent with the reports above. |

1. Zhang Y-L, Yuan J-Q, Wang K-F, et al. *Oncotarget* 2016; 7: 78985–78993; 2. Saito M, Shiraishi K, Kunitoh H, et al. *Cancer Science* 2016; 107: 713-720.
