# Supplementary material for: Randomized Phase III Study of EGFR Tyrosine Kinase Inhibitor and Intercalated Platinum-Doublet Chemotherapy for Non–Small Cell Lung Cancer Harboring EGFR Mutation
Source: Clin Cancer Res. 2025 Mar 31;31(12):2317–26. doi: 10.1158/1078-0432.CCR-24-3532 (PMC12163600; doi:10.1158/1078-0432.CCR-24-3532)
Supplement: Supplementary Figure S1 — Study hypothesis. Abbreviations: EGFR; epidermal growth factor receptor; EGFR-TKI, epidermal growth factor receptor tyrosine kinase inhibitor. [file ccr-24-3532_supplementary_figure_s1_suppsf1.pptx]

## Slide 1
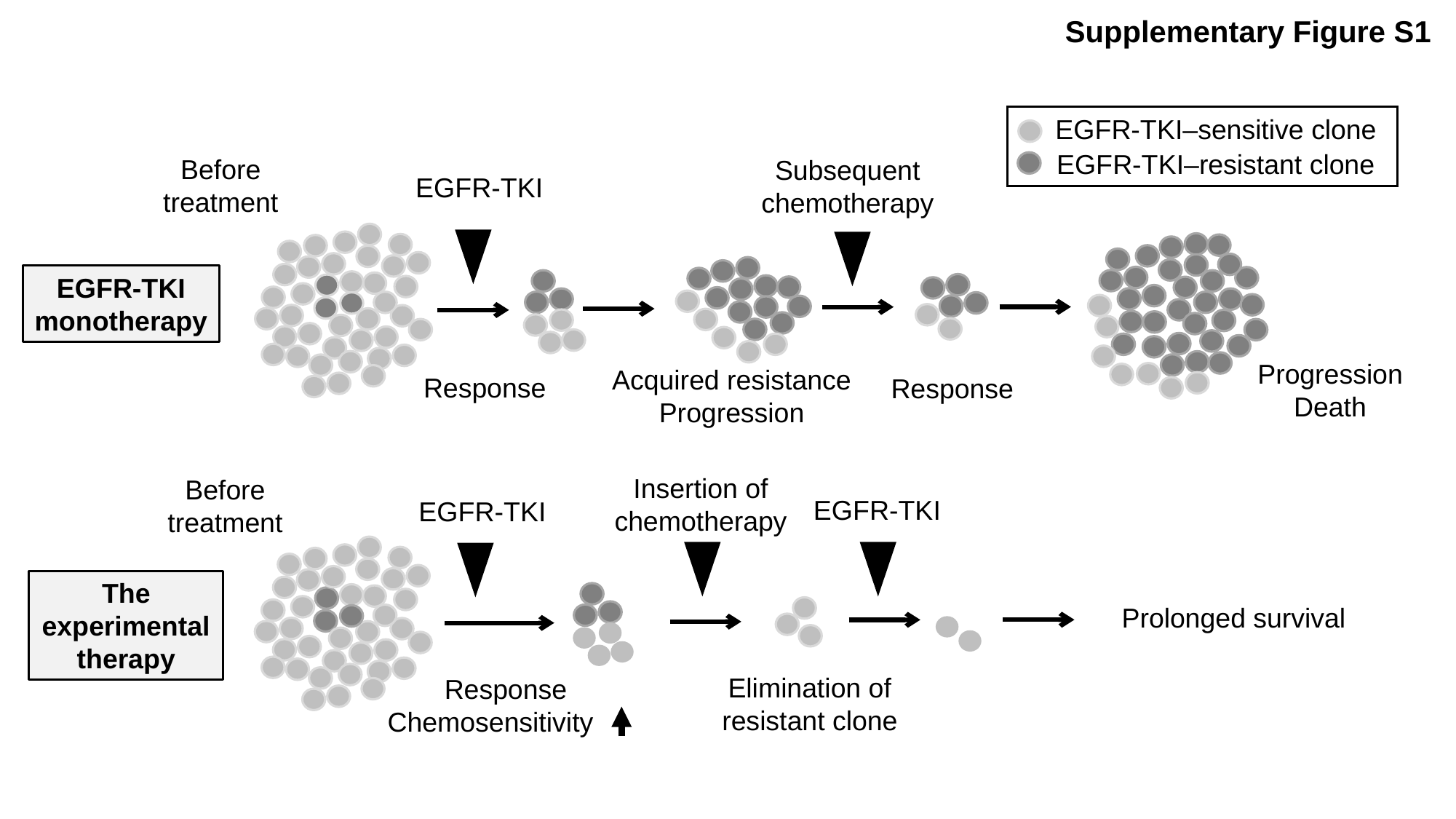

Supplementary Figure S1
EGFR-TKI–sensitive clone
EGFR-TKI–resistant clone
Before
treatment
Subsequent
chemotherapy
EGFR-TKI
EGFR-TKI
monotherapy
Progression
Death
Acquired resistance
Progression
Response
Response
Insertion of
chemotherapy
Before
treatment
EGFR-TKI
EGFR-TKI
The experimental therapy
Prolonged survival
Elimination of
resistant clone
Response
Chemosensitivity

## Slide 2
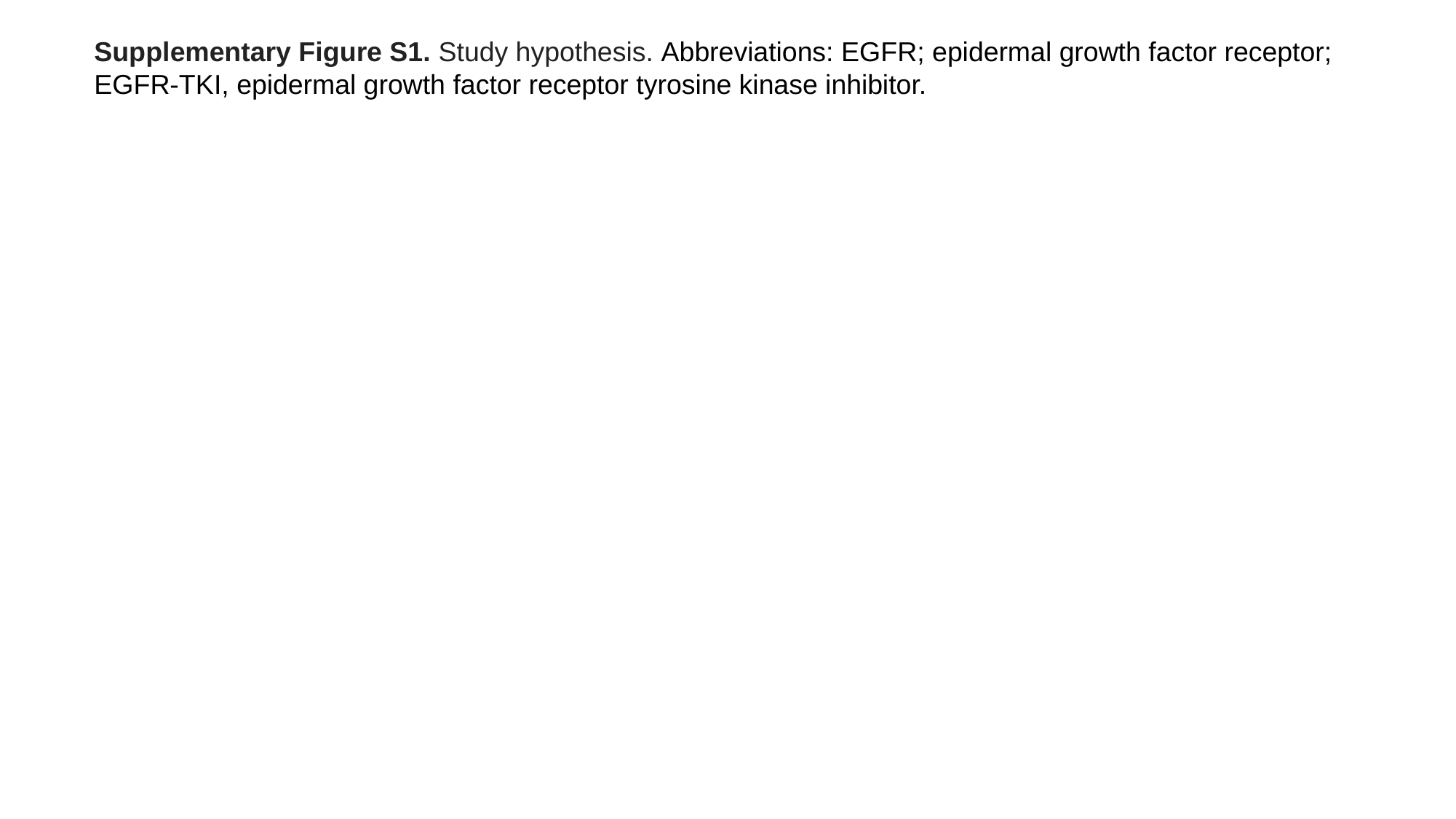

Supplementary Figure S1. Study hypothesis. Abbreviations: EGFR; epidermal growth factor receptor; EGFR-TKI, epidermal growth factor receptor tyrosine kinase inhibitor.
