# Supplementary material for: Randomized Phase III Study of EGFR Tyrosine Kinase Inhibitor and Intercalated Platinum-Doublet Chemotherapy for Non–Small Cell Lung Cancer Harboring EGFR Mutation
Source: Clin Cancer Res. 2025 Mar 31;31(12):2317–26. doi: 10.1158/1078-0432.CCR-24-3532 (PMC12163600; doi:10.1158/1078-0432.CCR-24-3532)
Supplement: Supplementary Figure S2 — Study design of JCOG1404/WJOG8214L. Abbreviations: NSCLC, non-small cell lung cancer; EGFR; epidermal growth factor receptor; Exon 19 DEL, EGFR exon 19 deletion; Exon 21 L858R, EGFR exon 21 L858 point mutation; ECOG, Eastern Clinical Oncology Group; PS, performance status; CNS, central nervous system; EGFR-TKI, epidermal growth factor receptor tyrosine kinase inhibitor; PD, progressive disease. [file ccr-24-3532_supplementary_figure_s2_suppsf2.pptx]

## Slide 1
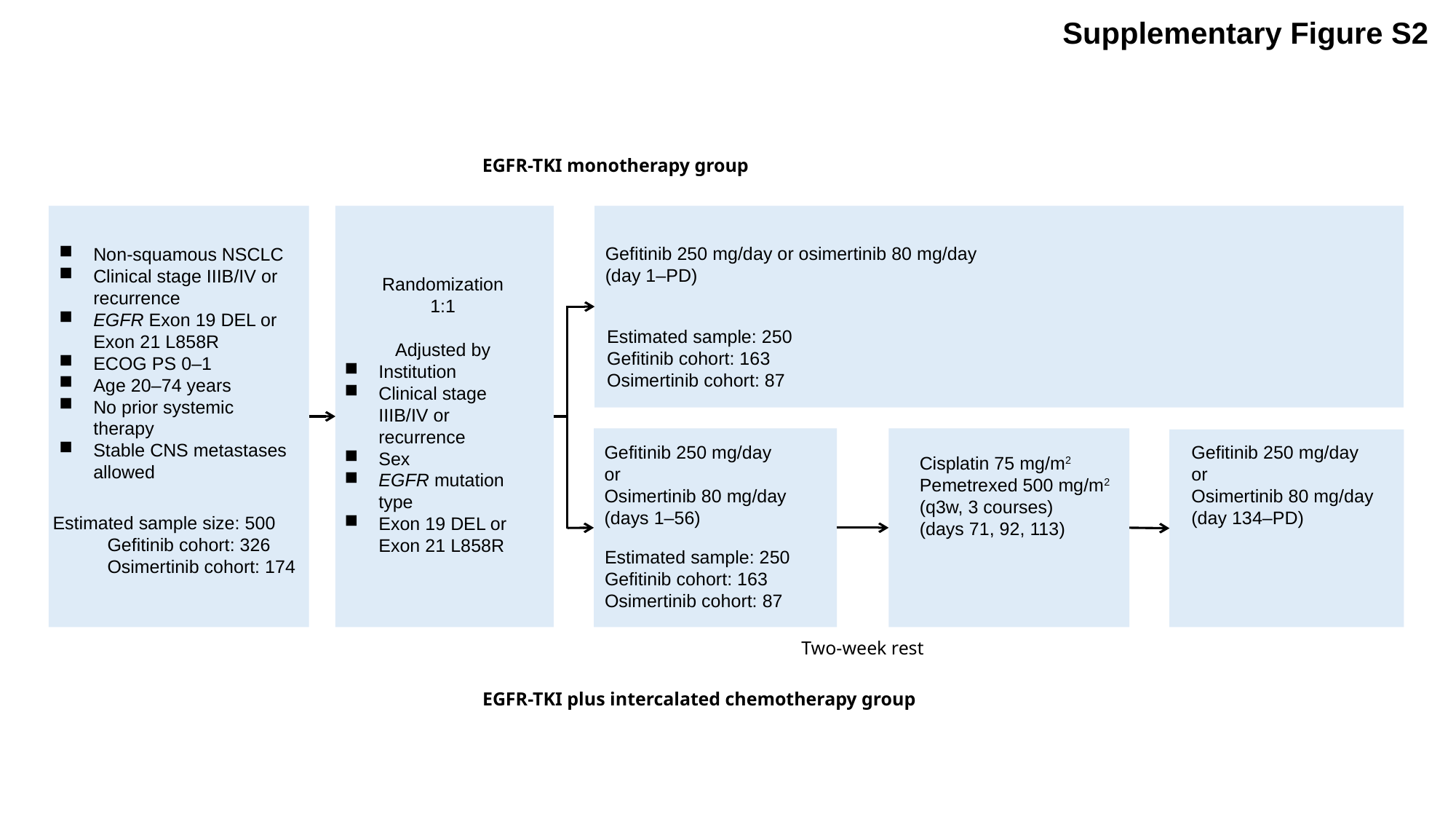

Supplementary Figure S2
EGFR-TKI monotherapy group
Gefitinib 250 mg/day or osimertinib 80 mg/day
(day 1–PD)
Non-squamous NSCLC
Clinical stage IIIB/IV or recurrence
EGFR Exon 19 DEL or Exon 21 L858R
ECOG PS 0–1
Age 20–74 years
No prior systemic therapy
Stable CNS metastases allowed
Randomization
1:1
Adjusted by
Institution
Clinical stage IIIB/IV or recurrence
Sex
EGFR mutation type
Exon 19 DEL or Exon 21 L858R
Estimated sample: 250
Gefitinib cohort: 163
Osimertinib cohort: 87
Gefitinib 250 mg/day
or
Osimertinib 80 mg/day
(days 1–56)
Gefitinib 250 mg/day
or
Osimertinib 80 mg/day
(day 134–PD)
Cisplatin 75 mg/m2
Pemetrexed 500 mg/m2
(q3w, 3 courses)
(days 71, 92, 113)
Estimated sample size: 500
Gefitinib cohort: 326
Osimertinib cohort: 174
Estimated sample: 250
Gefitinib cohort: 163
Osimertinib cohort: 87
Two-week rest
EGFR-TKI plus intercalated chemotherapy group

## Slide 2
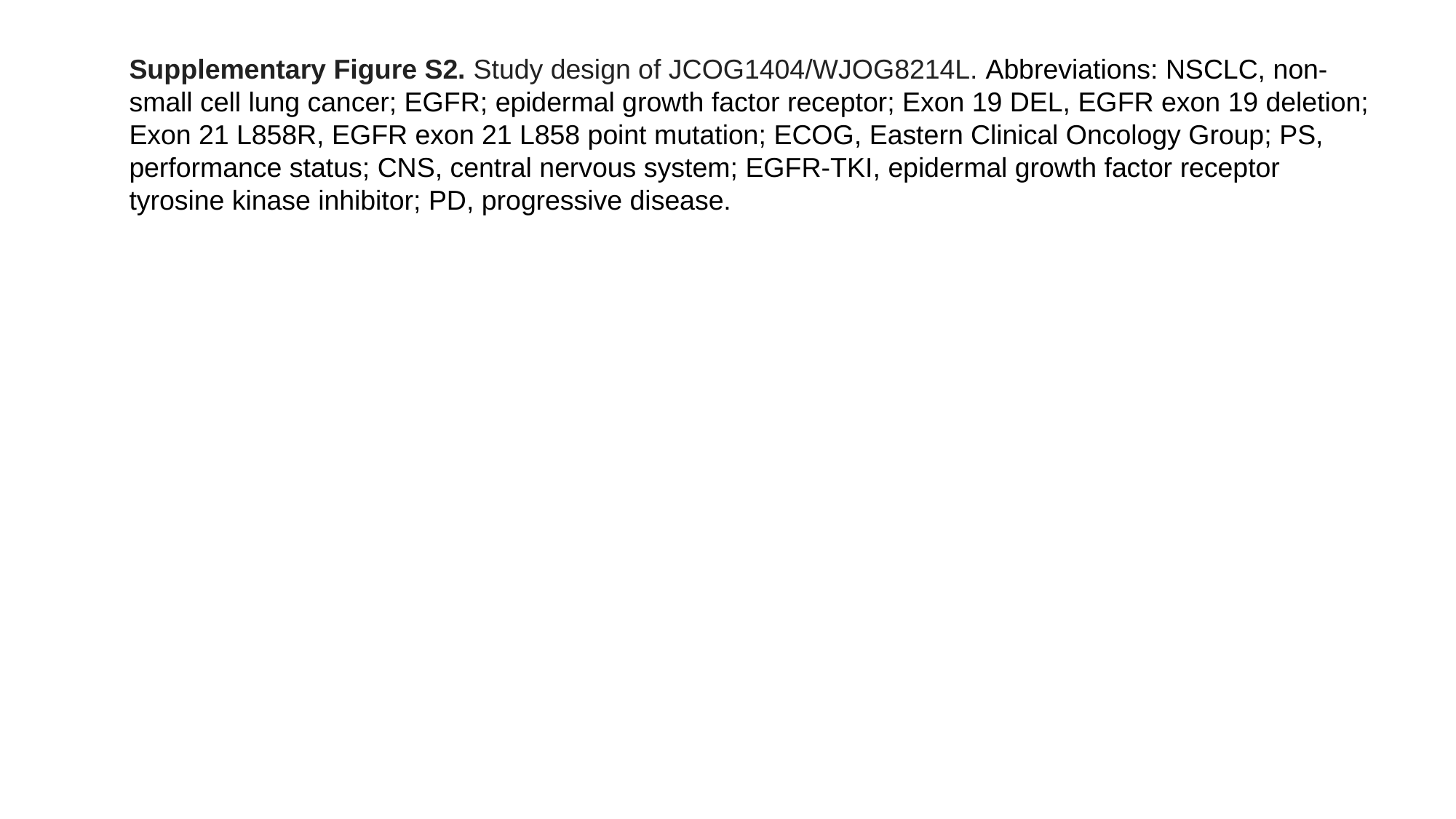

Supplementary Figure S2. Study design of JCOG1404/WJOG8214L. Abbreviations: NSCLC, non-small cell lung cancer; EGFR; epidermal growth factor receptor; Exon 19 DEL, EGFR exon 19 deletion; Exon 21 L858R, EGFR exon 21 L858 point mutation; ECOG, Eastern Clinical Oncology Group; PS, performance status; CNS, central nervous system; EGFR-TKI, epidermal growth factor receptor tyrosine kinase inhibitor; PD, progressive disease.
