# Supplementary material for: Randomized Phase III Study of EGFR Tyrosine Kinase Inhibitor and Intercalated Platinum-Doublet Chemotherapy for Non–Small Cell Lung Cancer Harboring EGFR Mutation
Source: Clin Cancer Res. 2025 Mar 31;31(12):2317–26. doi: 10.1158/1078-0432.CCR-24-3532 (PMC12163600; doi:10.1158/1078-0432.CCR-24-3532)
Supplement: Supplementary Figure S4 — Subgroup analyses of progression-free survival. (A) Gefitinib cohort. (B) Osimertinib cohort. Abbreviations: CI, confidence interval; EGFR, epidermal growth factor receptor; Ex19del, EGFR exon 19 deletion; L858R, EGFR exon 21 L858 point mutation; ECOG, Eastern Clinical Oncology group; PS, performance status; EGFR-TKI, epidermal growth factor receptor tyrosine kinase inhibitor; CNS, central nervous system. [file ccr-24-3532_supplementary_figure_s4_suppsf4.pptx]

## Slide 1
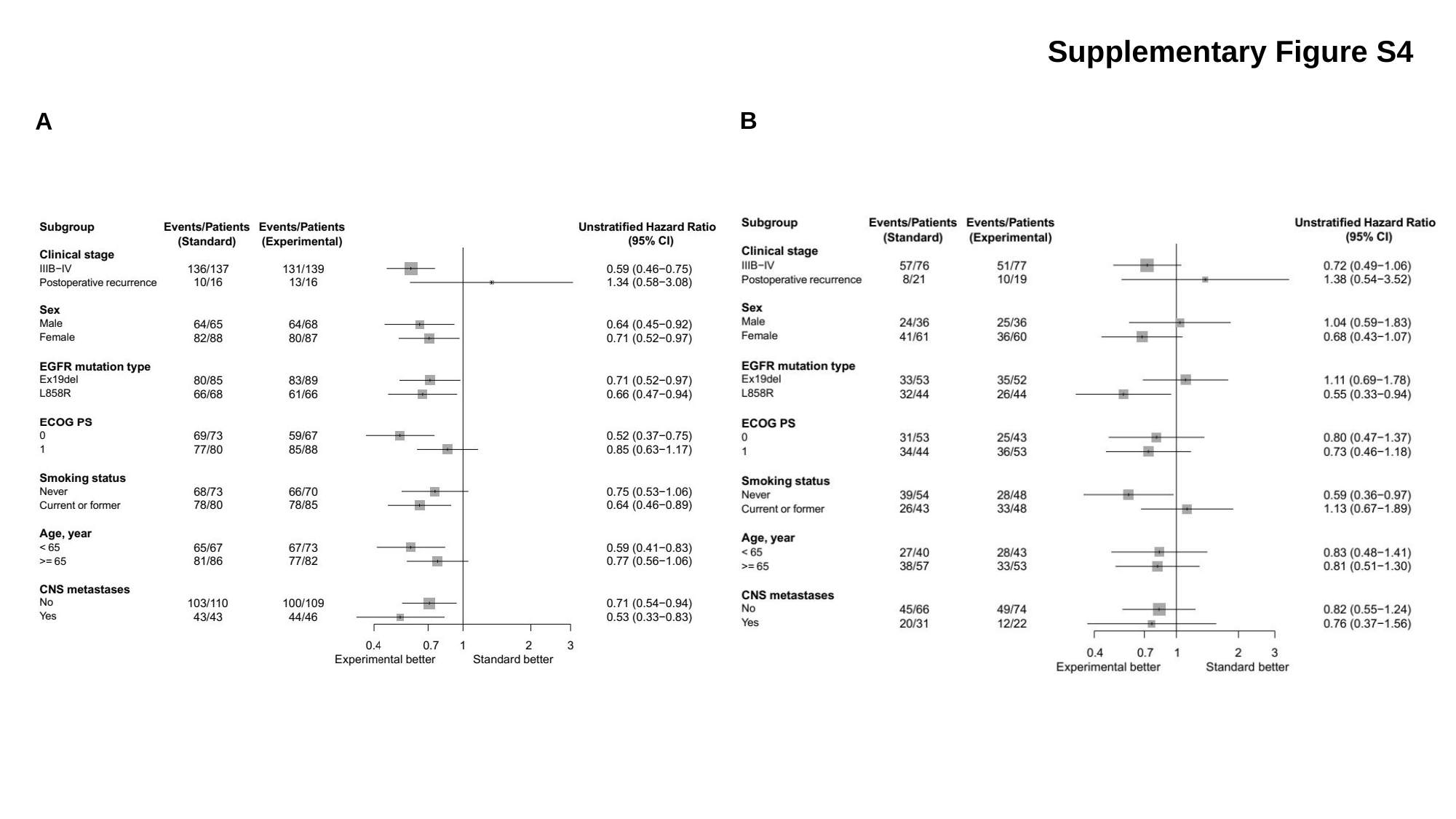

# Supplementary Figure S4
B
A

## Slide 2
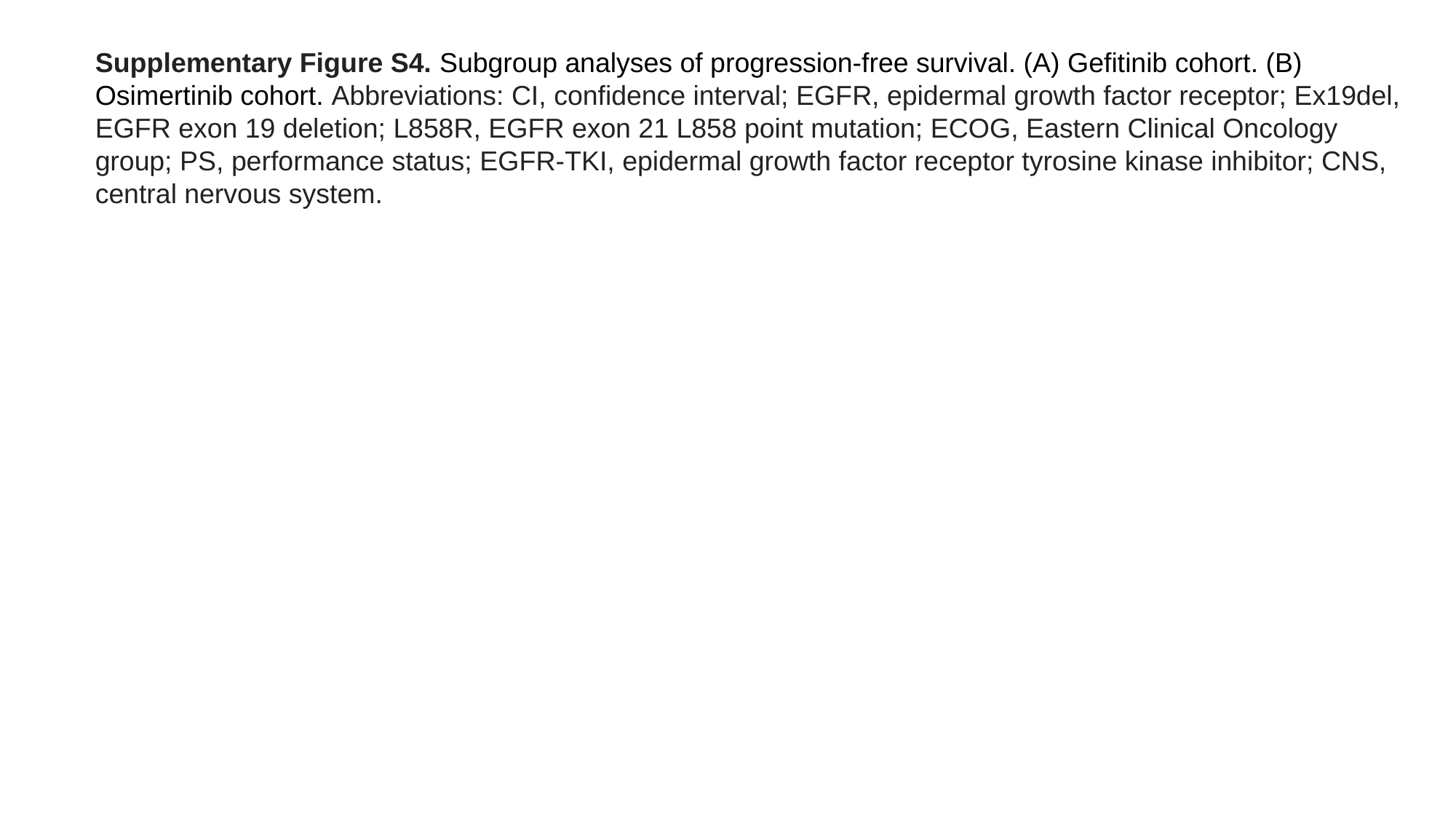

Supplementary Figure S4. Subgroup analyses of progression-free survival. (A) Gefitinib cohort. (B) Osimertinib cohort. Abbreviations: CI, confidence interval; EGFR, epidermal growth factor receptor; Ex19del, EGFR exon 19 deletion; L858R, EGFR exon 21 L858 point mutation; ECOG, Eastern Clinical Oncology group; PS, performance status; EGFR-TKI, epidermal growth factor receptor tyrosine kinase inhibitor; CNS, central nervous system.
