# Supplementary material for: Randomized Phase III Study of EGFR Tyrosine Kinase Inhibitor and Intercalated Platinum-Doublet Chemotherapy for Non–Small Cell Lung Cancer Harboring EGFR Mutation
Source: Clin Cancer Res. 2025 Mar 31;31(12):2317–26. doi: 10.1158/1078-0432.CCR-24-3532 (PMC12163600; doi:10.1158/1078-0432.CCR-24-3532)
Supplement: Supplementary Data S1 — Study Protocol of JCOG1404/WJGO8214L. [file ccr-24-3532_supplementary_data_s1_suppsd1.docx]

**Supplementary Data S1**

**
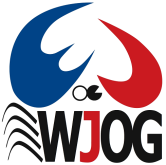
**
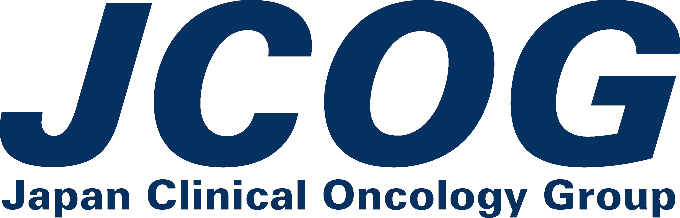


National Cancer Center Research and Development Fund 2020-J-3

“Basic Research for Establishing Standard Treatments for Adult Solid Cancers” Group

Japan Organization for Medical Research and Development commissioned R&D expenditure Innovative oncology practical application research project

"Randomised controlled trials of gefitinib or osimertinib monotherapy with intermediate cisplatin plus pemetrexed in gefitinib or osimertinib for EGFR mutation-positive advanced non-squamous non-small cell lung cancer"

**Japan Clinical Oncology Group (JCOG) West Japan Oncology Group (WJOG)**

**JCOG1404/WJOG8214L**

**A phase III study comparing gefitinib or osimertinib and inserted cisplatin and pemetrexed with gefitinib or osimertinib as a first-line treatment for patients with advanced non-squamous non-small-cell lung cancer harboring *EGFR* activating mutation**

**(AGAIN study)**

**JCOG Group Chair**

**JCOG Study Chair**

Yuchiro Ohe

Department of Respiratory Medicine, Central Hospital, National Cancer Research Center

〒104-0045 Tsukiji 5-1-1, Chyuo-ku, Tokyo

TEL: +81-3-3542-2511

FAX: +81-3-3545-5370

E-mail: yohe@ncc.go.jp

**JCOG Study Coodinator**

Shintaro Kanda

Shinshu Cancer Center, Shinshu University Hospital

〒390-8621 3-1-1 Asahi, Matsumoto City, Nagano Prefecture

TEL: +81-263-37-2554

FAX: +81-263-37-3302

E-mail: [skanda@shinshu-u.ac.jp](mailto:skanda@shinshu-u.ac.jp)

Seiji Niho

Department of Pulmonary Medicine and Clinical Immunology, Dokkyo Medical University

〒321-0293 880 Oaza Kitakobayashi, Mibu-cho, Shimotsuga-gun, Tochigi Prefecture

TEL: +81-282-86-1111

FAX: +81-282-86-7780

E-mail: [siniho@dokkyomed.ac.jp](mailto:siniho@dokkyomed.ac.jp)

**WJOG Respiratory Group Chair**

**WJOG Study Chair**

Nobuyuki Yamamoto

Internal Medicine III, Wakayama Medical University

〒641-8509 811-1 Ki-Misaidera, Wakayama City, Wakayama Prefecture,

TEL:073-447-2300

TEL: +81-73-447-2300

FAX: +81-73-446-2877

E-mail: nbyamamo@wakayama-med.ac.jp

**WJOG Study Coordinator**

Takayasu Kurata

Department of Thoracic Oncology, Kansai Medical University Hospital

〒573-1191 2-3-1 Hirakata Municipality, Osaka

TEL: 072-804-0101

FAX: 072-804-0131

E-mail: kuratat@hirakata.kmu.ac.jp

September 20, 2014 Protocol concept approved by JCOG Executive Committee (PC1404)

October 3, 2015 Protocol approved by JCOG Protocol Review Committee

October 23, 2015 Protocol concept approved by WJOG Respiratory Committee

May 23, 2016 Revision ver. 1.1 approved by JCOG Data and Safety Monitoring Committee

June 11, 2016 Revision ver. 1.1 approved by WJOG Respiratory Committee

June 20, 2016 Ver. 1.1 enacted

Septembeer 19, 2018 Amendment ver. 2.0 approved by JCOG Data and Safety Monitoring Committee

October 6, 2018 Amendment ver. 2.0 approved by WJOG Respiratory Committee

October 10, 2018 Ver. 2.0 enacted

August 26, 2019 Revision ver. 2.1.0 approved by JCOG Data and Safety Monitoring Committee

September 23, 2019 Revision ver. 2.1.0 approved by WJOG Respiratory Committee

September 30, 2019` Ver. 2.1.0 enacted

May 22, 2020 Revision ver. 2.2.0 approved by JCOG Data and Safety Monitoring Committee

June 20, 2020 Revision ver. 2.2.0 approved by WJOG Respiratory Committee

June 29, 2020 Ver. 2.2.0 enacted

# Summary

## Schema

- Patients enrolled before or in ver. 1.1 are administered gefitinib, and those enrolled in or after ver. 2.0 are administered osimertinib.

## Objectives

To confirm the usefulness of the regimen of administering gefitinib or osimertinib* alone following 3 courses of cisplatin+pemetrexed combination therapy after first administering gefitinib or osimertinib as the first-line treatment for patients with advanced non-squamous non-small-cell lung cancer harboring EGFR activating mutation in comparison with gefitinib or osimertinib* alone by a randomized controlled trial.

- Patients enrolled before or in ver. 1.1 are administered gefitinib, and those enrolled in or after ver. 2.0 are administered osimertinib.

Primary endpoint : Overall survival

Secondary endpoints : Progression-free survival, response rate, proportion of patients with adverse events, proportion of patients with serious adverse events, proportion of patients with exon 20 T790M point mutation of the EGFR gene in tumor during a period of progression.

## Subjects

* Refer to “4.2. Exclusion criteria” in patient registration.

1. Patients definitively diagnosed histologically or cytologically to have non-squamous non-small-cell lung cancer (adenocarcinoma, large cell lung cancer (except large cell neuroendocrine cancer), or non-small cell lung cancer with unidentified histological type) (See “3.2. Histological classification”).
2. Patients with stage IIIB/IV disease untreatable by radical radiation therapy or postoperative recurrence.
3. Patients with a genetic mutation in the tissue or cell sample that fulfills all of the following criteria.
   1. Presence of partial deletion of exon 19 or point mutation of exon 21 L858R in the EGFR gene*
   2. Absence of point mutation of exon 20 T790M in the EGFR gene* (However, those with point mutation of exon 20 T790M in the EGFR gene are also accepted if osimertinib is used.)
   3. Absence of KRAS gene mutation if the KRAS gene is identified before enrollment (However, identification of the KRAS gene mutation is not essential.)

- Mutation testing of the EGFR gene must be performed by one of the following methods (However, testing by liquid biopsy is unacceptable.)

1. Real-time PCR test using COBAS®EGFR mutation detection kit
2. Real-time PCR test using therascreen® EGFR mutation detection kit
3. PNA-LNA clamp method
4. PCR Invader method
5. Cycleave method
6. PCR-RFLP method
7. Loop-Hybrid method
8. Oncomine Dx Target Test Multi CDx System
9. Age at registration is between 20 and 74 years old.
10. ECOG performance status (PS) is 0 or 1 (PS must be recorded in the clinical record).
11. The presence or absence of a measurable lesion does not matter.
12. There is no symptomatic brain metastasis, meningeal carcinomatosis, or spinal metastasis needing radiation therapy or surgery.
13. There is no Grade 3 or severer superior vena cava syndrome, pericardial effusion, pleural effusion, or ascites.
    Regarding pleural effusion, patients may be registered unless Grade 3 or severer pleural effusion is observed 14 days after pleurodesis following drainage.
14. The patient has not undergone surgery involving organ resection within 28 days before registration. However, patients 14 or more days after surgery for cytology or biopsy or exploratory thoracotomy are considered eligible.
15. The patient has not undergone palliative radiation therapy for metastases of lung cancer within 14 days before registration (However, patients are considered eligible if palliative radiation therapy has ended 15 or more days before registration.).
16. The patient has not undergone systemic chemotherapy or radical chest radiation therapy including treatment for other cancers (However, patients can be registered if the washout period after postoperative chemotherapy by oral administration of UFT or S-1 following surgery for lung cancer until registration is 4 weeks or longer. Those who have undergone cisplatin combination chemotherapy after surgery for lung cancer can be registered if 48 weeks (336 days) or more have passed after the last day of chemotherapy (patients who have recurrence on the same day of the week 48 weeks after the last day of treatment can be registered.). Patients who have undergone surgery or hormone therapy for other cancers are also considered eligible.).
17. The most recent laboratory test within 14 days prior to registration (the same day of the week 2 weeks prior to the enrollment date is acceptable) meets all of the following.
18. Neutrophil count ≥1,500/mm^3^
19. Platelet count ≥10×10^4^/mm^3^
20. Total bilirubin ≤1.5 mg/dL
21. AST (GOT) ≤100 U/L
22. ALT (GPT) ≤100 U/L
23. Serum creatinine ≤1.2 mg/dL
24. Creatinine clearance ≥60 mL/min

If the estimated creatinine clearance is <60 mL/min, the patient is considered eligible if the measured value in 24-hour pooled urine is confirmed to be ≥60 mL/min.

Cockcroft-Gault formula

Male: Ccr = {(140 – age) x body weight (kg)} / {72 x serum creatinine (mg/dL)}

Female: Ccr = 0.85 x {(140 – age) x body weight (kg)} / {72 x serum creatinine (mg/dL)}

SpO_2_ ≥92% (room air)
However, if SpO_2_ is <92%, the patient is considered eligible if room air PaO_2_ is ≥60 Torr.

1. There is no finding of interstitial pneumonia or pulmonary fibrosis on CT examination of the chest.
2. Written consent to participation in the study has been obtained from the patient in person.

## Treatments

Patients registered before or in Ver. 1.1 are administered gefitinib, and those registered in or after ver. 2.0 are administered osimertinib.

**<Patients registered before or in ver. 1.1>**

**Arm A (standard regimen): Gefitinib alone**

Gefitinib: 250 mg/day, orally, once a day (continued until “6.2.2. Criteria for termination of protocol treatment” are met)

**Arm B (trial regimen)**

1. Gefitinib: 250 mg/day, orally, once a day, day 1-56
2. Two-week washout period
3. Cisplatin+pemetrexed combination therapy (three 3-week courses)

Cisplatin: 75 mg/m^2^ day 71, 92, 113

Pemetrexed: 500 mg/m^2^ day 71, 92, 113

1. Gefitinib 250 mg/day, orally, once a day, day 134- after cisplatin+pemetrexed combination therapy

(continued until “6.2.2. Criteria for termination of protocol treatment” are met)

**<Patients registered in or after ver. 2.0>**

**Arm A (standard regimen): Osimertinib alone**

Osimertinib: 80 mg/day, orally, once a day (continued until “6.2.2. Criteria for termination of protocol treatment” are met)

**Arm B (trial regimen)**

1. Osimertinib: 80 mg/day, orally, once a day, day 1-56
2. Two-week washout period
3. Cisplatin+pemetrexed combination therapy (three 3-week courses)

Cisplatin: 75 mg/m^2^ day 71, 92, 113

Pemetrexed: 500 mg/m^2^ day 71, 92, 113

1. Osimertinib 80 mg/day, orally, once a day, day 134- after cisplatin+pemetrexed combination therapy

(continued until “6.2.2. Criteria for termination of protocol treatment” are met)

## Planned sample size and study period

Planned number of patients: 500 patients

Registration period: 3 years. Follow-up period: 3 years after the end of registration. Analysis period: 1 year. Total study duration: 7 years

<Items added in ver. 2.0>

Planned number of patients: 500 patients

Registration period: 5 years. Follow-up period: 2 years after the end of registration. Analysis period: 1 year. Total study duration: 8 years

## Contact information

Eligibility criteria, treatment modification, and other issues requiring clinical decisions: Study Coordinator (front cover and 16.8.)

Enrollment procedure protocol, case report form (CRF) entries: JCOG Data Center (16.14.), WJCOG Data Center (16.15.)

Adverse event reporting: JCOG Data and Safety Monitoring Committee (16.11.)

**Table of contents**

0. Summary 3

0.1. Schema 3

0.2. Objectives 3

0.3. Subjects 3

0.4. Treatments 5

0.5. Planned sample size and study period 5

0.6. Contact information 5

1. Objectives 9

2. Background and the Rationale of the Trial 10

2.1. Subjects of the Study 10

2.2. Standard treatment for the target disease 17

2.3. Rationale for protocol treatment 19

2.4. Trial Design 30

2.5. Summary of advantages and disadvantages expected from the participation in the trial 35

2.6. Significance of this trial 36

2.7. Associated studies (including sample analysis studies) 36

2.8. JCOG biobank collaborating with BioBank Japan (BBJ) (JCOG-BBJ biobank) 36

3. Criteria and definitions used in this study 37

3.1. Stage classification （UICC-TNM 7^th^ edition） 37

3.2. Pasthological classification 38

4. Patients Selection Criteria 39

4.1. Exclusion criteria 40

5. Registration and randomization 41

5.1. Procedure of registration 41

5.2. Random allocation and adjustment factors 42

5.3. Procedures for accrual completion 42

6. Treatment Plan and Treatment Modification Criteria 44

6.1. Protocol treatment 44

6.2. Protocol Treatment Termination/Completion Criteria 48

6.3. Treatment modification criteria 50

6.4. Concomitant treatment and supportive care 56

6.5. Post-study Treatment 62

7. Expected Adverse Events 63

7.1. Expected Adverse Reactions 63

7.2. Evaluation of adverse events/reactions 64

8. Examination, evaluation and evaluation schedule 66

8.1. Examination and evaluation before registration 66

8.2. Definitions of evaluation periods 66

8.3. Examination and evaluation during treatment 67

8.4. Items of examinations and evaluations 53 or more weeks after the initiation of protocol treatment 72

8.5. Study calendar 73

9. Data Collection 75

9.1. Case Report Form (CRF）, Electronic Case Report Form (eCRF） 75

10. Reporting of Adverse Events 77

10.1. Serious Adverse Events and subjects of Expedited Reporting 77

10.2. Site Investigator/Representative's responsibility and the procedures of expedited reporting 79

10.3. Responsibilities of Principal Investigator/Study Coordinator 80

10.4. Responsibilities of the Site Investigator/Representative of participating institution (including reporting institution) 82

10.5. Responsibility of the administrator of the institution where the adverse event occurred 82

10.6. Evaluation by Data and Safety Monitoring Committee 82

11. Response Evaluation and Endpoint Definition 84

11.1. Response evaluation 84

11.2. Definitions of analyses sets 90

11.3. Definition of endpoints 91

12. Statistical Considerations 94

12.1. Main analysis and evaluation criteria 94

12.2. Target number of registrations, registration period, follow-up period 94

12.3. Interim analysis and early termination of trial 96

12.4. Analysis of secondary endpoints 98

12.5. Final analysis 99

12.6. Exploratory analyses 99

12.7. Early termination of the study 100

12.8. Procedures after early termination of the Study 101

13. ETHICAL CONSIDERATION 102

13.1. Human subject protection 102

13.2. Informed consent 102

13.3. Protection of personal information ans patient identification 103

13.4. Compliance with the protocol 105

13.5. Approval of the Ethics Review Committee of the medical institution 105

13.6. Protocol revision/amendment 106

13.7. Control of Conflicts of Interest (COIs) 107

13.8. Compensation 108

13.9. Intellectual Property 108

13.10. Disclosure of information on this study 108

14. MONItering and audit 109

14.1. Periodic monitoring 109

14.2. Site visit audits 110

15. Special Instructions 112

15.1. JCOG BioBank Japan (BBJ) Biorepository 112

15.2. Multiple Study Enrollment 112

16. Organization 113

16.1. Main study fund (funding source) of this study 113

16.2. JCOG（Japan Clinical Oncology Group） 113

16.3. WJOG（West Japan Oncology Group） 113

16.4. JCOG Chair 114

16.5. WJOG President 114

16.6. Study group and group chair 114

16.7. Study chair 114

16.8. Study coordinator 115

16.9. Participating institutions 116

16.10. JCOG Protocol Review Committee 118

16.11. Data and Safety Monitoring Committee 118

16.12. Audit Committee 118

16.13. Conglict of Interests 120

16.14. JCOG data center/operation office 120

16.15. WJOG data center 121

16.16. Protocol development 121

17. Pubrication of the study results and completion of the study 122

17.1. Papers and conference presentations 122

17.2. Primary Endpoint Report and Clinical Summary Report 122

17.3. Compketion of the study 122

18. References 124

19. Appendix 129

# Objectives

To confirm the usefulness of the regimen of administering gefitinib or osimertinib* alone following 3 courses of cisplatin+pemetrexed combination therapy after first administering gefitinib or osimertinib as the first-line treatment for patients with advanced non-squamous non-small-cell lung cancer harboring EGFR activating mutation in comparison with gefitinib or osimertinib* alone by a randomized controlled trial.

- Patients enrolled before or in ver. 1.1 are administered gefitinib, and those enrolled in or after ver. 2.0 are administered osimertinib.

Primary endpoint : Overall survival

Secondary endpoints : Progression-free survival, response rate, proportion of patients with adverse events, proportion of patients with serious adverse events, proportion of patients with exon 20 T790M point mutation of the EGFR gene in tumor during a period of progression.

# Background and the Rationale of the Trial

## Subjects of the Study

### Epidemiology

The morbidity and mortality from lung cancer in Japan have increased since 1950. According to the National Cancer Center Institution for Cancer Control, the number of patients who suffered lung cancer (malignant neoplasm of the trachea, bronchus, and lung) was about 108,000 (74,000 males and 34,000 females) in 2010 and is expected to increase to 125,000 (91,000 males and 34,000 females) in 2020.^1)^ The number of deaths due to lung cancer was 71,500 (51,400 males and 20,100 females) in 2012, ranking first among cancers at various sites in males and second only to colon cancer in females.^2)^

### Clinical pathology

Lung cancer is classified into non-small-cell lung cancer and small-cell lung cancer, with the former accounting for 80-85% of all lung cancers.

Non-small-cell lung cancer is classified by the histological type into squamous cell carcinoma, adenocarcinoma, and large cell carcinoma. Non-squamous non-small-cell lung cancer (non-small-cell lung cancers other than squamous cell carcinoma) accounts for about 65% of all lung cancers in males and 85% in females.

Recently, in non-small-cell lung cancer, particularly, adenocarcinoma, a few somatic cell genetic aberrations bearing carcinogenicity (carcinogenic genetic aberrations) have been discovered, and molecularly targeted drugs targeting them have been shown to be effective (most carcinogenic genetic aberrations are mutually exclusive, and the presence of 2 or more carcinogenic genetic aberrations in one tumor is rare).

Epidermal growth factor receptor (EGFR) gene mutation is one of such genetic aberrations, and the presence of EGFR gene mutation is both a prognostic factor (favorable prognostic factor) of advanced non-small-cell lung cancer and a predictive factor of the response to EGFR tyrosine kinase inhibitor (EGFR-TKI). EGFR gene mutations are frequently observed in Asians, females, non-smokers, and adenocarcinoma patients. The proportion of individuals with EGFR gene mutation is about 10-20% in Western countries but high at 30-50% among Japanese. There are several types in EGFR gene mutations, among which partial deletion of exon 19 and point mutation of exon 21 L858R are dominant, together accounting for 90% of all mutations.^3)^ In addition to these major mutation types, there are also rare mutation types, such as point mutation G719X of exon 18 and partial insertion of exon 20, but they are poorly responsive or non-responsive to EGFR-TKI and different from exon 19 partial deletion or exon 21 L858R point mutation.^4)^


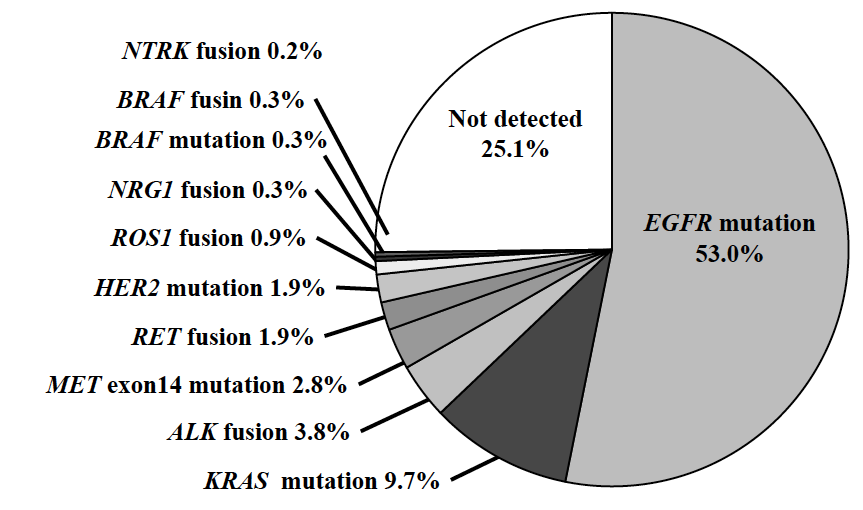


Figure 2.1.1. Carcinogenic genetic aberrations in Japanese with lung adenocarcinoma^5)^

### Staging

A staging system in which lesions are classified into limited disease (LD) and extensive disease (ED) is widely applied to small-cell lung cancer. On the other hand, non-small-cell lung cancer is staged from I to IV according to the degree of progression (UICC-TNM staging system, 7th edition). The T-factor is defined according to the maximum tumor diameter and degree of involvement of other organs, N-factor according to the extent of metastasis to regional lymph node, and M-factor according to the presence or absence of distant metastasis (See “3.1. Staging criteria (UICC-TNM staging system, 7th edition)).

### Overview of standard treatment and prognosis by stage

The standard treatment for stage IA disease with a maximum tumor diameter of ≤2 cm is radical surgery without postoperative chemotherapy, but there is no consensus about the effectiveness of surgery with postoperative chemotherapy for stage IA disease with a maximum diameter of 2-3 cm. Surgery with postoperative chemotherapy is recommended for stage IB, IIA, IIB, and radically operable IIIA disease. Also, the standard treatment for radically unresectable stage IIIA and IIIB disease is chemoradiotherapy. Drug therapy consisting primarily of anticancer agents is performed for stage IIIB and stage IV lesions, which are not indications for radical radiation therapy.

Table 2.1.4. Five-year survival rate of patients with non-small-cell lung cancer by the pathological stage^6)^

| Pathological staging | 5-year survival rate |
| --- | --- |
| IA | 86.8% |
| IB | 73.8% |
| IIA | 61.6% |
| IIB | 49.8% |
| IIIA | 40.9% |
| IIIB | 27.8% |
| IV | 27.9% |

Two-thirds of non-small-cell lung cancers are advanced cancers having distant metastases at the time of the diagnosis or untreatable by radical radiation therapy. Advanced non-small-cell lung cancer is a radically incurable systemic disease and is managed primarily by drug therapy aiming to palliate symptoms or prolong the survival.

For patients aged 74 years or less with a favorable PS, platinum-based combination chemotherapy is the standard initial chemotherapy, but the median survival time (MST) by this therapy is 8-15 months.^7)-9)^ For patients with EGFR gene mutation, EGFR-TKI (gefitinib, erlotinib, afatinib) are the first choices, and MST by this regimen is 22-36 months (gefitinib: 21.6-35.5 months, erlotinib: 19.3-27.7 months, afatinib: 23.1-28.2 months).^12)-23)^

<Items added in ver. 2.0>

As described in detail in 2.2.1.3), the use of osimertinib, a third-generation EGFR-TKI, was approved in Japan as the first-line treatment for EGFR-mutation-positive advanced/recurrent non-small-cell lung cancer in the third quarter of 2018 and became the first choice by replacing the previous first/second-generation EGFR-TKIs (gefitinib, erlotinib, afatinib). The FLAURA trial, which provided evidence for the approval, was a double-blind randomized controlled trial comparing osimertinib with a first-generation EGFR-TKI (gefitinib or erlotinib) conducted in PS 0-1 previously untreated EGFR-mutation-positive patients with advanced/recurrent non-small-cell lung cancer including those aged 75 years and older. Although the survival period was longer in those treated with osimertinib than in those treated with a first-generation EGFR-TKI (18-month survival rate: 83% vs. 71%), MST has not been obtained.^77)^

### Tumor-related complications

Symptoms associated with advanced lung cancer vary widely and are classified into 4 types according to the cause: ➀ symptoms due to extension/invasion to areas around the intrathoracic tumor, ➁ symptoms due to distant metastasis, ➂ non-specific systemic symptoms, and ➃ paraneoplastic syndrome.

➀As symptoms due to extension/invasion to areas around the intrathoracic tumor, respiratory symptoms, such as cough, bloody sputum/hemoptysis, and dyspnea, are frequently observed, but superior vena cava syndrome, dysphagia due to esophageal invasion, chest and back pain due to chest wall invasion, hiccups due to phrenic nerve invasion, hoarseness due to recurrent nerve invasion, and Horner syndrome due to upper sympathetic nerve infiltration may also be observed.

➁Symptoms due to distant metastasis may be focal symptoms due to brain metastasis, symptoms of intracranial hypertension, such as headache, nausea/vomiting, and disturbance of consciousness, localized pain and morbid fracture due to bone metastasis, and hypercalcemia due to bone lysis.

➂Non-specific systemic symptoms include anorexia, weight loss, emaciation, and fever. Also, in cancer patients, hyperactivation of the blood coagulation system is observed, occasionally causing venous thromboembolism such as deep vein thrombosis and pulmonary artery thromboembolism.

➃Paraneoplastic syndrome includes syndrome of inappropriate secretion of ADH causing hyponatremia.

Among complications, respiratory infections (observed in about 70% of all lung cancer patients), such as obstructive pneumonia, intratumoral abscess formation, and trachea (broncho)-esophageal fistula, are most important.

### Recurrence/progression patterns

The response rate (RR) of advanced non-small-cell lung cancer to first-line platinum-based chemotherapy is 30-40%, and median PFS is 4-6 months.^7)-9)^ RR of EGFR-mutation-positive advanced non-small-cell lung cancer to platinum-based chemotherapy is 30-47%, and median PFS is 4.6-6.9 months.^12)-23)^ RR of EGFR-mutation-positive non-small-cell lung cancer to EGFR-TKI is 60-70%, and median PFS is 9-14 months.^12)-23)^ By any treatment, nearly all patients experience re-progression. Non-small-cell lung cancer may metastasize to all organs, and the lung, pleura, pericardium, bone, brain, liver, and adrenal gland are common sites of metastasis.

### Prognostic/predictive factors

The presence of an EGFR gene mutation is a predictive as well as prognostic factor of the effectiveness of EGFR-TKI. According to a retrospective study that compared the prognosis of advanced non-small-cell lung cancer before the advent of EGFR-TKI between cancers with and without an EGFR gene mutation, the prognosis was more favorable in EGFR mutation-positive than mutation-negative non-small-cell lung cancer, with MST being 13.2 and 10.4 months, respectively.^24)^

Of the major EGFR mutations, the prognosis may be better in patients with exon 19 partial deletion than in those with exon 21 L858R point mutation. As for comparison between exon 19 partial deletion and exon 21 L858R point mutation, there have been reports of clinical studies using gefitinib against advanced lung adenocarcinoma that there was no difference in the effectiveness or prognosis between them.^14)16)^ On the other hand, there is also a report that RR, PFS, and overall survival (OS) by treatment with gefitinib or erlotinib were better in patients with exon 19 partial deletion than in those with exon 21 L858R point mutation.^25)^ In this study, 36 lung cancer patients with exon 19 partial deletion or with exon 21 L858R point mutation treated with gefitinib or erlotinib were retrospectively compared and showed that the treatment outcome was better in patients with exon 19 partial deletion than in those with exon 21 L858R point mutation with RR being 73% vs. 50%, PFS being 24 months vs. 10 months, and OS being 38 months and 17 months, respectively. Also, in an integrated analysis (631 patients) LUX-Lung3 study (307 patients) and LUX-Lung6 study (324 patients), in which afatinib and platinum-based combination chemotherapy were compared in patients with EGFR mutation-positive advanced lung adenocarcinoma, OS was better in those treated with afatinib (236 patients) than in those treated by chemotherapy (119 patients) in the group with exon 19 partial deletion (355 patients) (31.7 months vs. 20.7 months, HR=0.59, 95% CI 0.45-0.77, p=0.0001), but there was no difference in OS between those treated with afatinib (183 patients) and those treated with chemotherapy (93 patients) in the group with exon 21 L858R point mutation (22.1 months vs. 26.9 months, HR=1.25, 95% CI 0.92-1.71, p=0.1600).^23)^ In addition, by meta-analysis that integrated 7 phase III trials comparing EGFR-TKI and platinum-based combination chemotherapy as the first-line treatment, prolongation of PFS by the treatment with EGFR-TKI was more notable in lung cancer with exon 19 partial deletion than in lung cancer with exon 21 L858R point mutation (HR against chemotherapy for lung cancer with exon 19 partial deletion=0.24, HR against chemotherapy for lung cancer with exon 21 L858R point mutation=0.48, P<0.001).^26)^

Regarding the surgical history, there are many reports that the outcome is more favorable in patients with postoperative recurrence than in those with advanced lung cancer (stage III or IV untreatable by radical radiation therapy). According to retrospective analysis by National Cancer Center Hospital, MST was 13.3 months in stage IV patients who underwent chemotherapy but was 21.3 months in patients with postoperative recurrence.^26)^ In the phase III trial comparing gefitinib and CDDP+docetaxel (DTX) in patients with EGFR mutation-positive advanced or postoperative recurrent non-small-cell lung cancer patients (WJTOG3405 study) conducted in Japan, MST was 27.5 and 38.8 months in patients with advanced disease (phase III or IV cancer untreatable by radical radiation therapy) (101 patients) but was 42.7 and 47.6 months, respectively, in those with postoperative recurrence (71 patients).^15)^ Also, in the retrospective study in 496 patients with EGFR mutation-positive lung cancer carried out in the United States, MST was 30 months in stage IV patients (366 patients) and 32 months in patients with postoperative recurrence (130 patients) (HR=1.36, 95% CI 1.05-1.76, p=0.019).^28)^

Sex has long been recognized as a prognostic factor of advanced non-small-cell lung cancer, and many reports support a better prognosis in females than in males. For example, multivariate analysis in a retrospective study of 3,455 Japanese patients with advanced non-small-cell lung cancer showed that sex was a prognostic factor alone with PS, age, disease stage, and smoking history (females had a better prognosis with HR=0.747, 95% CI 0.677-0.825, p<0.0001).^29)^ When the subjects were restricted to patients with EGFR mutation-positive lung cancer, no sex difference was observed in the above retrospective study in the United States, but the prolongation of PFS by EGFR-TKI was more notable in females than in males in the above meta-analysis integrating 7 phase III trials comparing EGFR-TKI and platinum-based chemotherapy (HR=0.33 against chemotherapy in females, HR=0.45 against chemotherapy in males, P=0.02).^26)^ In this study, sex was included in adjustment factors in consideration of the previous reports and the results of meta-analyses about all non-small-cell lung cancers.

<Items added in ver. 2.0>

FLAURA study is the only clinical study of osimertinib in patients with untreated EGFR mutation-positive advanced/recurrent non-small-cell lung cancer, and data concerning whether there are predictive factors of the effectiveness of osimertinib other than EGFR gene mutations are insufficient.

### Rationale for the selection of patient population

#### 1) Reasons for the selection of EGFR mutation-positive (exon19 partial deletion or exon21 L858R point mutation) non-small-cell lung cancer as the target disease

➀ **EGFR mutation-positive patients**

The first choice as the first-line treatment for EGFR mutation-positive advanced non-small-cell lung cancer is EGFR-TKI alone based on the results of a few phase III trials comparing it with platinum-based combination chemotherapy in Japan.^12)-23)^

Although 70-80% of the EGFR mutation-positive non-small-cell lung cancers respond to this therapy, most of them resistance and are re-exacerbated after a progression-free survival period. Gatekeeper mutation (point mutation of exon 20 T790M),^30)^ activation of collateral pathways, such as HGF (hepatocyte growth factor)-Met pathway,^31)^ and transformation to small-cell lung cancer^32)^, are known as molecular biological mechanisms of such resistance to EGFR-TKI, but no practical treatment/strategy to prevent/overcome resistance has been established.^33)^ In addition, EGFR-TKI is presently administered alone continuously until acquisition of resistance or progression, after which it is usually switched to other drugs as practiced conventionally in chemotherapy for lung cancer, but the validation of the appropriateness of this administration method has been insufficient. The development of more effective therapeutic strategies using EGFR-TKI is considered important, because, as observed above, the incidence of EGFR mutation-positive lung cancer is higher in Japanese than in Western populations. Therefore, EGFR mutation-positive lung cancer were selected as the target disease.

As of 2015, in Japan, real time PCR method based on Scorpion-ARMS (therascreen®) and COBAS®EGFR mutation detection kit were approved as pharmaceuticals and used under health insurance as an extracorporeal diagnostic agents for EGFR gene mutations. In addition, in daily practice, techniques including PNA-LNA clamp method, PCR Invader method, Cycleave method, loop-hybrid method, PCR-RFLP method, which are considered equivalent to the two examinations above in sensitivity, are used. Allele-specific PCR including Scorpion-ARMS method can detect mutations if sample DNA contains tumor-derived DNA at 1%, the variation among assays is not considered large, with a high concordance rate (κ index) of 0.70-1.00.^34)^ Therefore, in this study, these 7 testing methods are permitted for the detection of EGFR susceptibility gene mutations and T790M mutations. While testing by next generation sequencing is highly sensitive, it is presently a research level testing method, and it is not used in this study as a method for detecting EGFR gene mutations until its usefulness is sufficiently validated.

<Items added in ver. 2.1.0>

Oncomine Dx Target Test Multi CDx System (Oncomine) was added to treatments covered by health insurance as a method for testing EGFR gene mutations on June 1, 2019. Because facilities that adopt this method at the time of selection of the first-line treatment of advanced non-small-cell lung cancer are expected to increase for the future due to the advantage of the ability to examine multiple genetic aberrations with a small amount of sample, Oncomine is accepted as a method for EGFR gene mutation testing at the time of patient enrollment in this study.

Oncomine is a testing method that can simultaneously examine 4 driver gene mutations important for the selection of treatment for advanced non-small-cell lung cancer (EGFR exon 19 deletion and EGFR exon 21 L858R mutation, ALK fusion gene, ROS1 fusion gene, BRAF V600E mutation) by next-generation sequencing and is one of the companion diagnostic agents of osimertinib, the trial drug in this study. Regarding its performance in detection of EGFR gene mutations (EGFR exon 19 deletions and EGFR exon 21 L858R mutations), Oncomine has been compared with the COBAS®EGFR mutation detection kit, which is widely used as an extracorporeal diagnostic agent and is also used in this study, reporting high positive and negative concordance rates of 100% and 95.3%, respectively, in a total of 119 cases.

Therefore, we judged that there is no problem in permitting the use of Oncomine as a method to test EGFR gene mutations in this study.

Table 2.1.8. Studies that compared platinum-based combination chemotherapy and EGFR-TKI as treatments for EGFR mutation-positive advanced non-small-cell lung cancer

| Study | Treatments | n | PFS (months)  HR (95% confidence intervals) | | OS (months)  HR (95% confidence intervals) | |
| --- | --- | --- | --- | --- | --- | --- |
| IPASS^12)13)^  (Subgroup) | CBDCA+PTX | 129 | 6.3 | 0.48  (0.36-0.64) | 21.9 | 1.00  (0.76-1.33) |
|  | Gefitinib | 132 | 9.5 |  | 21.6 |  |
| WJTOG3405^14)15)^ | CDDP+DTX | 86 | 6.3 | 0.49  (0.34-0.71) | 38.8 | 1.19  (0.76-1.82) |
|  | Gefitinib | 86 | 9.2 |  | 35.5 |  |
| NEJ002^16)17)^ | CBDCA+PTX | 114 | 5.4 | 0.32  (0.24-0.44) | 26.6 | 0.89  (0.63-1.24) |
|  | Gefitinib | 114 | 10.8 |  | 27.7 |  |
| OPTIMAL^18)19)^ | CBDCA+GEM | 72 | 4.6 | 0.16  (0.10-0.26) | 28.9 | 1.07  (0.79-1.44) |
|  | Erlotinib | 82 | 13.7 |  | 27.7 |  |
| EURTAC^20)^ | Platinum-based chemotherapy | 87 | 5.2 | 0.37  (0.25-0.54) | 19.5 | 1.04  (0.65-1.68) |
|  | Erlotinib | 86 | 9.7 |  | 19.3 |  |
| LUX-Lung 3^21)23)^ | CDDP+PEM | 115 | 6.9 | 0.58  (0.43-0.78) | 28.2 | 0.88 |
|  | Afatinib | 230 | 11.1 |  | 28.2 |  |
| LUX-Lung 6^22)23)^ | CDDP+GEM | 122 | 5.6 | 0.28  (0.20-0.39) | 23.5 | 0.93 |
|  | Afatinib | 242 | 11.0 |  | 23.1 |  |

CBDCA: Carboplatin, PTX: Paclitaxel, CDDP: Cisplatin, DTX: Docetaxel, GEM: Gemcitabine

**➁Reason for restriction of EGFR gene mutations to exon19 partial deletion and exon21 L858R point mutation**

Exon 19 partial deletion and exon 21 L858R point mutation together account for more than 90% of all EGFR gene mutations, and most of the subjects in the phase III trials carried out to the present were patients with lung cancer with these 2 major types of gene mutations. Concerning rare EGFR gene mutations other than exon 19 partial deletion and exon 21 L858R point mutation, there have been many reports that they are less responsive than the 2 major mutations, or irresponsive, to EGFR-TKI (RR ≤60%, median PFS ≤8 months, MST=15-16 months),^4)^ and other drugs are occasionally used in preference to EGFR-TKI. In this study, therefore, patients with non-small-cell lung cancer with either exon 19 partial deletion or exon 21 L858R point mutation, which are susceptible to EGFR-TKI among EGFR mutations, were adopted as subjects, and those with other rare EGFR mutations were excluded from the subjects of this study.

**➂Reason for the inclusion of patients with EGFR exon 20 T790M point mutation in the subjects when osimertinib is used in the protocol treatment <Item added in ver. 2.0>**

EGFR exon 20 T790M point mutation is known as a mechanism of resistance to first/second-generation EGFR-TKI, but it is occasionally observed also in patients previously untreated with EGFR-TKI.^51)-54)^ Since first/second-generation EGFR-TKI was considered ineffective for T790M-positive lung cancer, it was not a target disease before and in ver. 1.1, in which the protocol treatment was gefitinib. However, as described in detail in 2.2.1.3), with the change of the protocol treatment in this study to osimertinib, as osimertinib is reported to be equally effective for T790M-positive and T790M-negative lung cancers, it was decided to also include patients with EGFR exon 20 T790M point mutation in the subjects.^77)78)^

**➃Gene mutations other than EGFR gene mutations**

v-Ki-ras2 Kirsten rat sarcoma viral oncogene homolog (KRAS) is a carcinogenic gene and is reported to be a negative predictive factor of the effect of EGFR-TKI against advanced non-small-cell lung cancer. However, in daily clinical practice, the therapeutic strategy is not often changed simply because being positive for KRAS gene mutations, and it is very rare that both EGFR gene mutation and KRAS gene mutation are positive. For this reason, it was decided not to require KRAS gene testing, which is not always performed in routine clinical practice, and to exclude patients from the subjects only when KRAS gene mutation is known to be positive.

Also, in patients with anaplastic lymphoma kinase (ALK) fusion gene, ALK tyrosine kinase inhibitor (crizotinib) has been highly effective with response rates of 60-80% and progression-free survival time of about 10 months. Since it is very unlikely that EGFR gene mutations and ALK fusion gene occur simultaneously, testing of ALK fusion gene is not required in this study. However, there has been a report of lung cancers with concurrent EGFR gene mutation and ALK fusion gene as very rare cases. For such patients, since there is no standard therapeutic strategy, and EGFR-TKI alone is a treatment option, patients with ALK fusion gene are also included as subjects if they have susceptible mutations of EGFR.

#### 2) Reason for the selection of non-squamous non-small-cell lung cancer as the target disease

In the present drug therapy for advanced non-small-cell lung cancer, generally, squamous and non-squamous cell cancers are distinguished first for its treatment. There are the following 3 reasons for this therapeutic approach. Non-squamous non-small-cell lung cancer is considered appropriate as the target of the present study in which the trial regimens include EGFR-TKI and CDDP+PEM.

➀ PEM tends to be more effective for non-squamous cell carcinoma but less effective for squamous cell carcinoma than other anticancer agents (discussed in detail below).

➁ Carcinogenic gene aberrations that can be treatment targets, such as EGFR gene mutation, which is a predictive factor of the efficacy of EGFR-TKI, are frequently found in adenocarcinoma, which accounts for a majority of non-squamous cell carcinoma (discussed in detail below).

➂ Bevacizumab (BEV), an anti-VEGF monoclonal antibody preparation, is contraindicated for squamous cell carcinoma because of the high risk of hemoptysis as its adverse reaction, and its indication is restricted to non-squamous cell carcinoma. (In a phase II randomized controlled trial to evaluate the usefulness of the addition of BEV to CBDCA+PTX conducted overseas, severe hemoptysis was observed in 6, with 4 deaths due to hemoptysis, of the 66 patients treated with BEV (including 13 with squamous cell carcinoma), and 5 of the 6 patients who showed hemoptysis and all 4 patients who died had squamous cell carcinoma.^35)^)

A phase III trial conducted in Europe that compared CDDP+gemcitabine (GEM) combination therapy and CDDP+PEM combination therapy in chemotherapy-naive advanced non-small-cell lung cancer (1,725 patients) demonstrated non-inferiority of CDDP+PEM to CDDP+GEM regarding OS, which was the primary endpoint (MST: 10.3 months vs. 10.3 months, hazard ratio (HR)= 0.94 (95%CI: 0.84-1.05)), and subgroup analysis showed that CDDP+PEM was superior to CDDP+GEM in non-squamous cell carcinoma (1,000 patients) in OS (MST: 11.8 months vs. 10.4 months, HR=0.81 (95%CI: 0.70-0.94) and that CDDP+PEM was inferior to CDDP+GEM in squamous cell carcinoma (473 patients) (MST: 9.4 months vs. 10.8 months, HR=1.23 (95%CI: 1.00-1.51).^9)^ Moreover, PEM has been confirmed to be more effective against non-squamous cell carcinoma and less effective against squamous cell carcinoma than other drugs by meta-analysis of multiple studies.^36)^

EGFR gene mutations are unlikely to be selected as a target of initial treatment, because they are frequently observed in adenocarcinoma but in only 13% of squamous cell carcinomas, and because the effectiveness of EGFR-TKI is limited even in EGFR mutation-positive cases (RR: 25.0%, median PFS: 1.4 months).^37)38)^ Reports on EGFR mutation-positive adenosquamous carcinoma or carcinoma with polymorphic, sarcomatoid, or sarcomatous elements are fewer than even those on squamous carcinoma, and the response rate and median PFS vary among them from 0-50% and 3.0-5.3 months, respectively. Therefore, it was decided not to include such cancers in the target diseases of this study. Also, advanced lung cancers other than those that recurred after surgery are often definitively diagnosed using minute cell/tissue samples collected transtracheally with forceps or an aspiration needle under bronchoscopy or percutaneously from the body surface with an aspiration needle. Pathological examination of such microsamples may successfully identify the lesion as non-small-cell lung cancer but often fall short of determining its histological type according to the WHO classification. In this study, such non-small-cell lung cancers whose histological types are not identifiable are also included in target diseases if they are positive for EGFR gene mutations.

#### 3) Reasons for the selection of postoperative recurrence as a target disease

Since many of the postoperative recurrences occur as metastases to distant organs, which are usually treated by chemotherapy similarly to stage IV disease, cases of postoperative recurrence are included as subjects in many clinical studies of stage IIIB to IV lung cancer. Therefore, postoperative recurrence is also included in target diseases in this study. However, as mentioned earlier, the prognosis may be better in cases of postoperative recurrence compared with stage IIIB or IV disease untreatable by radical radiation therapy.^26)^ For this reason, in this study, the history of surgery (stage IIIB or IV disease untreatable by radical radiation therapy vs. postoperative recurrence) is adopted as an adjustment factor for allocation.

<Item added in ver. 2.0>

In the stage of protocol preparation, patients with postoperative recurrence who had undergone cisplatin-based combination chemotherapy as postoperative chemotherapy were not considered eligible, because the possibility that the susceptibility to, or safety of, cisplatin+pemetrexed among the protocol treatments differ from those who had not undergone the therapy was not ruled out, and only those who had received postoperative chemotherapy using UFT or S-1 were regarded as eligible. However, in recent clinical trials or clinical studies of initial treatments for advanced or recurrent non-small-cell lung cancer, there is a tendency to accept patients who had recurrence 6-12 months or more after cisplatin-based combination chemotherapy as postoperative chemotherapy. Also, according to the report from a Japanese institution on a retrospective study of 16 cases that underwent cisplatin-based combination chemotherapy again for postoperative recurrence after postoperative cisplatin-based combination chemotherapy, the response rate was 31.2%, median progression-free survival rate was 6.5 months, and median survival time was 28.0 months, which were comparable to the outcomes in cases of progression/recurrence without postoperative cisplatin-based combination chemotherapy, and there was also no safety problem.^79)^

Thus, when a questionnaire survey was made in institutions participating in the JCOG Lung Cancer Group in March 2017, asking whether patients who had undergone postoperative cisplatin-based combination chemotherapy can be regarded as eligible, responses were obtained from a total of 40 institutions consisting of 33 of the 48 institutions participating JCOG and 7 of the 22 institutions participating in WJOG (response rate: 57%). The answers were “Cases of postoperative recurrence 6 months or more after cisplatin-based combination chemotherapy may be enrolled,” from 8 institutions; “Cases of postoperative recurrence 12 months or more after cisplatin-based combination chemotherapy may be enrolled,” from 20 institutions, “Cases of postoperative recurrence after cisplatin-based combination chemotherapy are not considered eligible,” from 10 institutions, and “Cases of postoperative recurrence 3 years/5 years or more after cisplatin-based combination chemotherapy may be enrolled,” from the remaining 2 institutions. About 70% of the institutions supported enrollment of “cases of postoperative recurrence 6 months or more after cisplatin-based combination chemotherapy”, and these results were presented to the group meeting on June 15, 2017 and approved in the Lung Cancer Group.

For these reasons, it was decided to also incorporate cases of postoperative recurrence after cisplatin-based combination chemotherapy following surgery for lung cancer as subjects if the time from the last day of chemotherapy was 12 months or longer.

## Standard treatment for the target disease

### History of the development of treatment to the current standard treatment

#### Standard treatment for advanced non-small-cell lung cancer

Chemotherapy is the standard treatment for patients with advanced non-small-cell lung cancer with a good general condition, and meta-analysis has shown that prolongation of OS (from 4.5 to 6.0 months), 9% decrease in mortality risk (HR=0.77), and an improvement in 1-year survival rate (from 20% to 29%) compared with the best supportive care (BSC).^39)^ Among regimens of chemotherapy, those including a platinum-containing drug is superior to those not including a platinum-containing drug in RR and 1-year survival rate.^40)^ The 2014 edition of the guideline of the Japan Lung Cancer Society also recommended the concomitant use of a platinum-containing drug and a third-generation anticancer drug for PS 0-1 advanced non-small-cell lung cancer. Before the advent of PEM, third-generation anticancer drugs were irinotecan, vinorelbine (VNR), paclitaxel (PTX), DTX, and GEM, and a few phase III clinical trials were carried out to compare their combinations with platinum-containing drugs, but all combinations showed similar effects with none being particularly effective.^7)8)^ As platinum-containing drugs, CDDP and CBDCA (Carboplatin) are used, and meta-analysis that compared them showed that CDDP was superior to CBDCA in RR (30% vs. 24%) and OS for non-squamous cell carcinoma (HR=1.12, 95%CI=1.01-1.23) and by combinations with third-generation anticancer drugs (HR=1.11, 95%CI=1.01-1.21).^41)^

#### Standard treatment for advanced non-squamous non-small-cell lung cancer

Thereafter, as mentioned in 2.1.8. 2), since CDDP+PEM was superior to CDDP+GEM in OS against non-squamous non-small-cell lung cancer in a phase III trial carried out after the advent of PEM,^9)^ CDDP+PEM began to be recommended as a regimen for non-squamous non-small-cell lung cancer. Moreover, in an European phase III trial that examined the usefulness of maintenance therapy using PEM alone in patients who showed SD or better responses to CDDP+PEM (PARAMOUNT study), both PFS (median: 4.1 months vs. 2.8 months, HR=0.62, 95%CI=0.49-0.79) and MST (13.9 months vs. 11.0 months, HR=0.78, 95%CI=0.64-0.96) were better in the PEM maintenance therapy group (359 patients) than in the placebo group (180 patients).^10)11)^ From these results, maintenance therapy using PEM alone is recommended to patients who have shown SD or better response to CDDP+PEM.

At the same time, since the results of a phase III trial (ECOG4599 study) confirmed an additional effect of BEV to the effect of CBDCA+PTX against advanced non-squamous non-small-cell lung cancer (MST=10.3 months by CBDCA+PTX vs. 12.3 months by CBDCA+PTX+BEV, HR=0.79, 95%CI=0.67-0.92),^42)^ CBDCA+PTX/BEV is also considered a current standard treatment. However, in a phase III trial that examined the additional effect of BEV to CDDP+GEM, prolongation of PFS, which was a primary endpoint, was observed, but prolongation of OS was not.^43)44)^ Also, BEV is contraindicated for patients with cavitation, infiltration or adjacency to a large vessel, uncontrollable hypertension, hemoptysis, or active episodes of bleeding because of the risk of adverse events including bleeding, and its indications are limited. According to a single-center retrospective study in Japan, 154 (54.4%) of the 283 patients with advanced non-squamous non-small-cell lung cancer who had previously undergone chemotherapy had indications for BEV.^45)^ For these reasons, the addition of BEV to CDDP-based chemotherapy is not recommended for patients with non-squamous non-small-cell lung cancer.

#### Standard therapy for EGFR-positive non-small-cell lung cancer

As mentioned above, in a few phase III trials that compared platinum-based combination chemotherapy and EGFR-TKI alone as the initial treatment for patients with EGFR-positive advanced non-small-cell lung cancer, EGFR-TKI alone was superior in PFS, which was the primary endpoint, and symptomatic improvement was more rapid and notable.^12)-23)^ Therefore, EGFR-TKI is the first choice from the initial treatment for EGFR-positive advanced non-small-cell lung cancer.

Presently, in Japan, three EGFR-TKIs, i.e., gefitinib, erlotinib, and afatinib, are available, but the standard treatment is considered to be gefitinib. Although there has not been a clinical study that directly compared relative merits of these EGFR-TKIs as a first-line treatment, there was no major difference in PFS in studies comparing them with other chemotherapies (Table 2.1.8.). Also, according to the analysis of the EGFR-positive group in the WJOG5108L study, in which gefitinib was compared with erlotinib as a second-line or subsequent therapy for pulmonary adenocarcinoma regardless of the presence or absence of EGFR gene mutations, no major difference was observed in PFS, OS, or RR.^46)^

Thus, there is no clear difference in efficacy, but gefitinib is adopted as a standard treatment in Japan for the following reasons.

1. Gefitinib has been used in major studies conducted in Japan.
2. The incidences of diarrhea and rash, which are typical adverse events associated with EGFR-TKI, are lower with gefitinib than with erlotinib or afatinib.
3. Gefitinib is less expensive (by standard daily dose: \6,712.7/gefitinib tablet (250 mg), \10,642.60/erlotinib tablet (150 mg), \11,198.50/afatinib tablet (40 mg).
4. Gefitinib is used most widely in daily clinical practice in Japan.

Regarding the results of a phase III trial in Japan, median PFS with gefitinib alone was 10.4 months, 1/2-year PFS was 42.1%/8.1%. As for Grade 3/4 adverse events, diarrhea was observed in 0.9%, rash in 5.3%, liver dysfunction in 26.3%, and drug-induced lung damage in 2.6% of the patients.^16)^

In the United States, as approval of gefitinib was cancelled, erlotinib or afatinib is often used. For this reason, erlotinib or afatinib is used frequently rather than gefitinib in major overseas clinical studies.

<Items added in ver. 2.0>

From the third quarter of 2018, the indication of the third-generation EGFR-TKI osimertinib was expanded from “EGFR-TKI-resistant EGFR T790M mutation-positive inoperable or recurrent non-small-cell lung cancer” to “EGFR mutation-positive inoperable or recurrent non-small-cell lung cancer”, and the standard treatment for initial treatment of EGFR mutation-positive advanced non-small-cell lung cancer is expected to change from gefitinib to the third-generation EGFR-TKI osimertinib. While osimertinib acts on 790M point mutation-positive EGFR, which is a mutation resistant to the first/second-generation EGFR-TKIs, its activity against wild EGFR, which normal cells also have, is weak.

The above change in standard treatment is based on the results of an international collaborative randomized double-blind phase III trial to compare a first-generation EGFR-TKI (gefitinib or erlotinib) and osimertinib in patients with previously untreated EGFR-mutation-positive advanced non-small-cell lung cancer (FLAURA study). In this study, osimertinib was superior to the first-generation EGFR-TKI in PFS, which was the primary endpoint [median PFS: 18.9 months vs. 10.2 months, HR=0.46 (95%CI=0.37-0.57)], and tended to be superior also in OS despite insufficiency in the number of events [median OS not attained by either treatment, HR=0.63 (95%CI=0.45-0.88), 18-month survival rate: 83% vs. 71%].^77)^ Moreover, as shown in Table 2.2.1., the incidences of adverse events characteristic of EGFR-TKI, such as skin disorder (rash) and liver disorder (AST/ALT elevation) and serious adverse events, were also low. The incidences of Grade 3 or severer neutropenia or thrombocytopenia were 1%, which was slightly higher than 0% for gefitinib in WJTOG3405.^14)^

Table 2.2.1. Major adverse events associated with EGFR-TKI

| Clinical study (n) | Skin disorder (%) | | Paronychia (%) | | Diarrhea (%) | | Liver disorder (%) | | Lung disorder (%) | |
| --- | --- | --- | --- | --- | --- | --- | --- | --- | --- | --- |
|  | All | ≥G3 | All | ≥G3 | All | ≥G3 | All | ≥G3 | All | ≥G3 |
| Gefitinib | | | | | | | | | | |
| WJTOG3405^14)^ (n=87) | 85% | 2% | 32% | 1% | 54% | 1% | 70% | 28% | 2% | 1% |
| NEJ002^16)^ (n=114) | 71% | 5% | - | - | 34% | 1% | 55% | 26% | 5% | 3% |
| Erlotinib | | | | | | | | | | |
| OPTIMAL^18)^ (n=83) | 43% | 2% | 4% | 0% | 25% | 1% | 37% | 4% | - | - |
| EUROTAC^20)^ (n=84) | 80% | 13% | - | - | 57% | 5% | 6% | 2% | 1% | 1% |
| JO22903 Phase II^47)^  (n=103) | 83% | 14% | 66% | 1% | 81% | 1% | 33% | 8% | 5% | 2% |
| Afatinib | | | | | | | | | | |
| LUX-Lung3 Japanese^48)^ (n=54) | 98% | 20% | 87% | 24% | 100% | 20% | - | - | 4% | 2% |
| Osimertinib | | | | | | | | | | |
| FLAURA^77)^ (n=65) | 58% | 1% | 35% | <1% | 58% | 2% | (AST)  9% | (AST)  1% | 4% | - |

### Standard treatment regimen in this trial

The current standard treatment for the target disease of this study is gefitinib monotherapy. Whether gefitinib should be discontinued at the time of progression or continued even after progression has been controversial, but, from the results of IMPRESS study described below, it is now considered that gefitinib should be discontinued at the time of progression and switched to second-line treatment.

IMPRESS study was conducted in patients with EGFR mutation-positive lung cancer whose condition became PD after showing response or control of disease for 6 months or longer by gefitinib alone as the first-line treatment to compare the regimens of CDDP+PEM by continuing gefitinib after PD and CDDP+PEM by replacing gefitinib with placebo. PFS, which was the primary endpoint, was 5.4 months in both treatment groups (HR=0.86 (95%CI: 0.65-1.13), p=0.273), and OS was poorer in the group continued to be treated with gefitinib (PFS: 14.8 months vs. 17.2 months, HR=1.62 (95%CI: 1.05-2.52), p=0.029).^49)^ These results indicated denied OS-prolonging effect in continuing gefitinib in platinum-based chemotherapy as the second-line treatment following gefitinib alone. Therefore, if progression is observed by gefitinib alone as the first-line treatment, gefitinib should be discontinued and switched to another regimen. Also, when the therapy is changed, the first choice is platinum-based combination chemotherapy.

There has been no clinical study that examined the pros and cons of chemotherapies other than platinum-based combination chemotherapy as a second-line treatment, and their benefits are unclear.

<Item added in ver. 2.0>

As described in detail in 2.2.1.3), in response to the results of the international collaborative randomized double-blind phase III trial (FLAURA study), the standard treatment for the target disease of this study is expected to be switched to osimertinib monotherapy from the third quarter of 2018.

## Rationale for protocol treatment

### Drugs

#### 1) Gefitinib

Gefitinib is a low-molecular-weight compound that selectively inhibits EGFR tyrosine kinase. It suppresses proliferation of tumor cells and induces apoptosis by binding with the ATP-binding sites competitively against ATP, thus, inhibiting EGFR autophosphorylation and blocking signal transmission. In addition, gefitinib shows an inhibitory activity at a lower concentration against mutant EGFR than against wild-type EGFR. It is administered orally, its bioavailability is about 60%, time until peak blood concentration after oral administration (Tmax) is 3-6 hours, half-life in blood is 48 hours, and daily oral administration for 7-10 days is necessary until equilibration of blood concentration. It is metabolized primarily by the liver, and 86% is excreted in feces, and less than 4% in the urine.

#### 2) Cisplatin (CDDP)

Cisplatin (CDDP) is metal complex ion of the heavy metal platinum. It produces cytocidal action by binding with DNA chains in cancer cells and inhibiting DNA synthesis and subsequent mitosis of tumor cells. It is administered by injection, and its blood concentration after intravenous drip infusion shows a biphasic decline curve with a long β-phase half-life of about 100 hours. It plays a central role in combination chemotherapies against many solid cancers including lung cancer because of demonstration of its synergy with various drugs and mildness of bone marrow toxicity. Its major toxicities include nausea/vomiting, kidney toxicity, and neurotoxicity. Sufficient fluid administration is needed before and after its administration for the prevention of kidney toxicity.

#### 3) Pemetrexed (PEM)

Pemetrexed (PEM) produces antitumor effects by suppressing the activities of multiple enzymes of the folic acid metabolizing system (thymidylate synthetase (TS), dihydrofolate reductase (DHFR), glycinamide ribonucleotide formyltransferase (GARFT)) and inhibiting DNA/RNA synthesis. It is administered by injection, its elimination half-life on daily administration by 10-minute intravenous drip infusion every 21 days is 2.74 hours, and most of the dose is excreted in the urine in the unmetabolized form. It is concomitantly administered with daily oral administration of folic acid and intramuscular injection of vitamin B_12_ every 9 weeks to mitigate adverse reactions.

#### 4) Osimertinib <Item added in ver. 2.0>

Osimertinib is a low-molecular-weight compound with a strong irreversible inhibitory action on susceptible mutant and T790M-mutant EGFR tyrosine kinase but a weak inhibitory action on wild-type EGFR tyrosine kinase. It is administered orally, the time until peak blood concentration after oral administration (Tmax) is about 6 hours, and half-life in blood is 48.6 hours. It is excreted primarily in feces, with 67.8% excreted in feces and 14.2% in the urine.

### Standard treatment regimen in this trial

The trial regimen in this study is first administering gefitinib alone for 8 weeks, inserting 3 courses of cisplatin+pemetrexed combination therapy after a 2-week non-dosing period,* then administering gefitinib alone again.

*In this study, “insertion” is defined as performing a regimen, interrupting it with a different regimen, and resuming the first regimen. Also, “concomitant combination therapy” means simultaneously performing different treatments, “alternating therapy” means administering different regimens alternately, and “consecutive combination therapy” means performing one regimen followed by another.

#### 1) Gefitinib and insertion of chemotherapy

1. **EGFR-TKI therapy and EGFR-TKI-resistant cells**

While about 70-80% of patients with EGFR-mutation-positive lung cancer respond to gefitinib alone, most of them suffer re-progression after a progression-free survival period. A cause of such re-progression is the molecular biological mechanism of acquired resistance to EGFR-TKI, and an involvement of exon 20 T790M point mutation as mentioned above has been confirmed (50%). Regarding the mechanism of the appearance of cells with a mechanism of resistance to EGFR-TKI, there are two possibilities: First, cells with a mechanism of resistance of EGFR-TKI do not exist before treatment but appear secondarily during treatment with EGFR-TKI; second, such cells are mixed in the tumor from before treatment and become apparent with decreases in susceptible cells.

The proportion of EGFR mutation-positive cells in each sample of tumor tissue varies, and there is a report that retrospectively showed a reduction of RR and shortening of OS by gefitinib treatment in tumors heterologously consisting of EGFR mutation-positive cells and wild-type cells compared with those consisting of EGFR mutation-positive cells alone.^50)^ In this report, resected specimens from patients with non-small-cell lung cancer treated with gefitinib for recurrence after surgery (21 patients) were analyzed, and EGFR gene mutations were analyzed by selecting a maximum of 60 sites in each tumor and analyzing EGFR gene mutations by direct sequencing. Of the 21 patients, 6 had areas of not only mutant EGFR cells but also wild-type EGFR cells, and PFS and MST with and without the presence of wild-type EGFR cell areas were 7.5 months vs. 18 months and 16.5 months vs. 27 months, respectively, being inferior in tumors with wild-type EGFR areas.

There are also reports that T790M resistant mutations were observed in 34-80% of patients with EGFR mutation-positive lung cancer even before treatment by the use of highly-sensitive detection methods.^51)-54)^

Thus, there is the possibility that, in such EGFR mutation-positive lung cancer, not only EGFR-TKI-susceptible mutant cancer cells but also wild-type cancer cells and EGFR-TKI-resistant cells, such as T790M mutant, underlie from before treatment with EGFR-TKI. Long-term administration of gefitinib alone to such patients is considered to lead to proliferation of gefitinib-non-responsive cells and progression.

1. **Combined use of EGFR-TKI and chemotherapy**

As observed above, since there is the possibility of the secondary appearance of cells with a mechanism of resistance to EGFR-TKI during treatment with EGFR-TKI and the presence of underlying wild-type cancer cells or EGFR-TKI-resistant cells in EGFR mutation-positive lung cancer from before treatment with EGFR-TKI, combinations of EGFR-TKI with chemotherapies have been developed as a measure to improve the therapeutic outcome. However, there is no information about the appropriate timing of the concomitant use of EGFR-TKI and chemotherapy.

1. **Regimen of inserting chemotherapy during the administration of EGFR-TKI**

By the Norton-Simon hypothesis, the growth rate of tumors decline with enlargement (Gomperzian growth), and the growth rate and susceptibility to chemotherapy are high while tumors are still small. Based on this hypothesis, it is important to perform chemotherapy while cells with a resistance mechanism against EGFR-TKI are small in number. Therefore, the regimen of inserting chemotherapy during gefitinib therapy was devised. This regimen aims to prevent proliferation of gefitinib-unresponsive/resistant cells in an early stage. It also aims to enhance the effect of chemotherapy per se and to prolong PFS/OS by performing chemotherapy while tumor mass is reduced by the effect of gefitinib. Since this regimen of inserting chemotherapy during the administration of EGFR-TKI was shown to be effective and safe by a phase II trial that we conducted based on this hypothesis, we decided to examine the usefulness of the regimen of inserting chemotherapy during EGFR-TKI in this study.


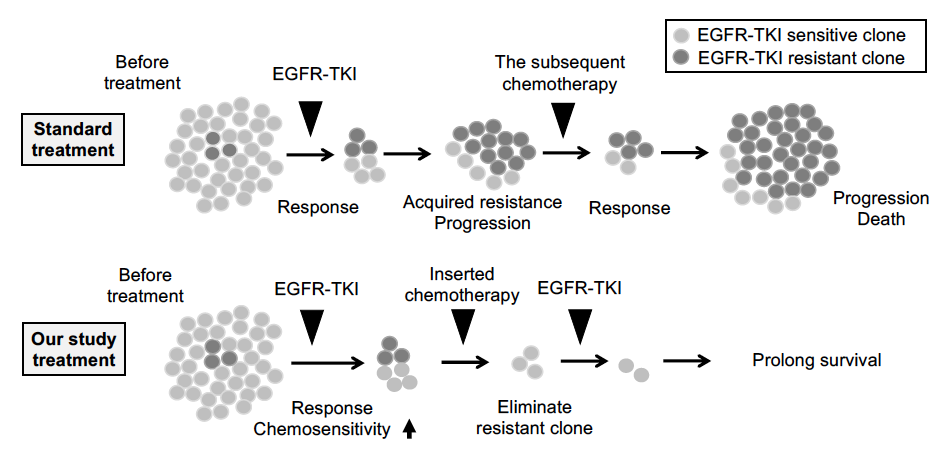


Figure 2.3.2.a Hypothesis about the effect of insertion of chemotherapy

1. **Reasons for not selecting concomitant combination therapy**

In a few previous phase III trials against all non-small-cell lung cancers, not only no additional effect of the simultaneous administration of gefitinib or erlotinib to platinum-based chemotherapy was observed, but also the survival curve was slightly inferior in the combination therapy group, although the difference was not significant.^55)56)^ Also, in basic experiments using wild-type EGFR cancer cells, antagonism was observed by the concomitant combination of gefitinib and chemotherapy with gefitinib arresting the cell cycle in the G1 phase and attenuating the effect of cytocidal anticancer agents or inhibiting cisplatin-induced caspase-independent cell death, and treatment without their simultaneous use was advantageous.^57)-59)^ Thus, there is the possibility that the simultaneous use of EGFR-TKI suppresses cell proliferation and attenuate the effects of cytocidal anticancer agents. For this reason, we inserted chemotherapy after the initial gefitinib therapy with a non-dosing period rather than performing them simultaneously.

1. **Reason for not selecting alternating therapy**

Alternating therapy aims to prevent increases in resistant cells by alternately performing different regimens regardless of whether tumor cells have increased or decreased. However, the insertion therapy used in this study was devised by considering that resistant cells remaining after a sufficient decrease in tumor cells as a result of treatment effect can be eradicated by chemotherapy. The hypotheses of insertion therapy and alternating therapy differ in this respect.

Also, in a phase III trial that compared a standard treatment consisting of platinum-based drug/GEM and a trial treatment using erlotinib as a substitute from day 15 to day 28 during platinum-based drug/GEM therapy against advanced non-small-cell lung cancer (FASTACT-2 study), the median PFS and MST in the EGFR mutation-positive subgroup were 16.8 months and 31.4 months, respectively.^60)^

#### 2) Results of a single-center phase II trial performed at National Cancer Center Hospital^61)^

1. **Trial therapy**


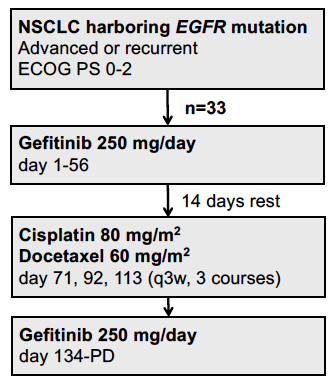


Figure 2.3.2.b Schema

Primary endpoint: 2-year progression-free survival rate

Secondary endpoints: RR, OS, incidence of adverse events

Intended number of registrations: 33 cases, threshold 20%, expected value 40%, one-tailed α=0.05, β=0.2

Criterion for efficacy: The trial therapy is judged to be effective if 2-year progression-free survival is achieved in 11 or more patients.

1. **Results**

Since 2-year progression-free survival was achieved in 12 of the 33 registered patients, the trial therapy was judged to be effective. Particularly, the long-term progression-free survival rate/survival rate were excellent. The 2-year progression-free survival rate was 40.2%. PFS and OS in all 33 patients are shown below.


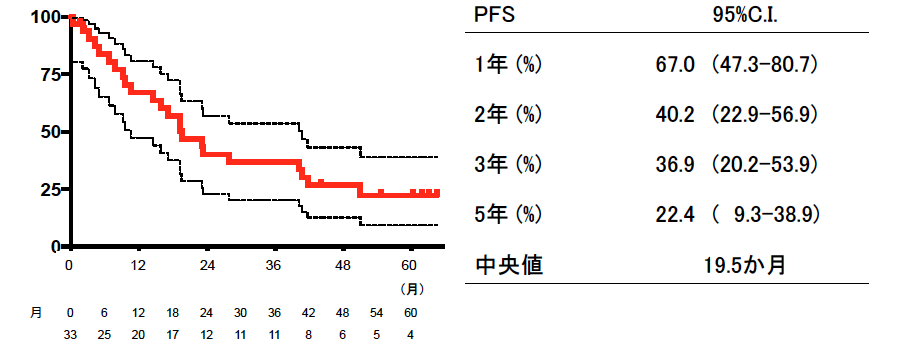

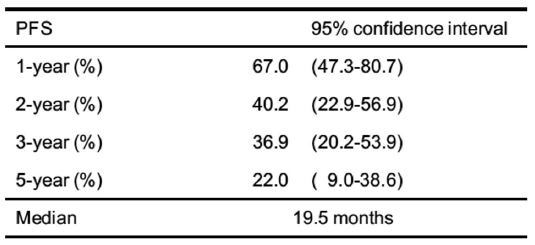


Figure 2.3.2.c PFS


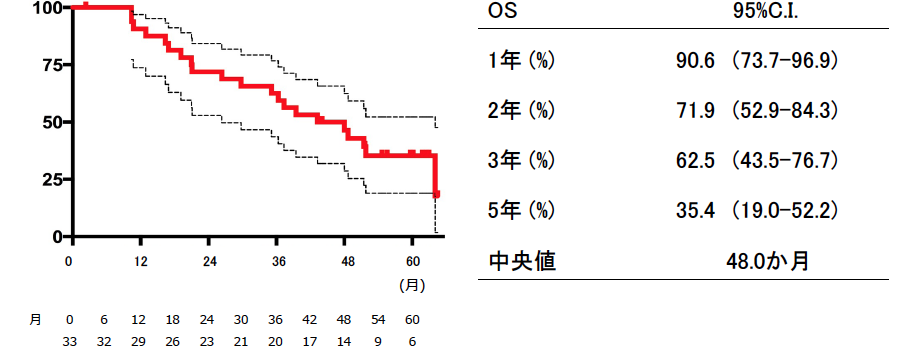

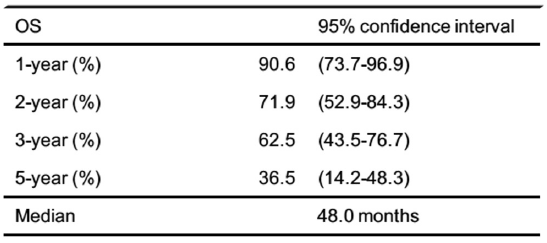


Figure 2.3.2.d OS

1. **Adverse Events**

There were no unexpected serious adverse events or treatment-related deaths, and no intensification of toxicity by the combined use was observed compared with previous reports on gefitinib monotherapy or CDDP+DTX therapy.

Table 2.3.2.a Adverse Events

| NCI-CTCAE 3.0 Grade | Grade 1 | Grade 2 | Grade 3 | Grade 4 |
| --- | --- | --- | --- | --- |
| Anorexia | 10 | 14 | 2 | 0 |
| Serum creatinine increased | 5 | 3 | 0 | 0 |
| Neutrophil count decreased | 4 | 5 | 3 | 14 |
| Anemia | 10 | 4 | 0 | 0 |
| Platelet count decreased | 2 | 1 | 0 | 0 |
| Febrile neutropenia | - | - | 4 | 0 |
| Alopecia | 12 | 6 | - | - |
| Diarrhea | 21 | 4 | 1 | 0 |
| Rash | 15 | 15 | 1 | 0 |
| Elevated AST | 13 | 2 | 4 | 0 |
| Elevated ALT | 11 | 5 | 4 | 0 |
| Pneumonitis | 1 | 0 | 0 | 0 |

#### Reason for the setting of the duration of the initial gefitinib therapy at 8 weeks

Many studies have reported that the median period until PR is reached is 6-8 weeks when EGFR-TKI is used as the first-line treatment for EGFR mutation-positive lung cancer. Also, in one study, the median treatment period required for the tumor mass to become minimum was 5 months.^62)^ On the other hand, there is a report of an in vitro experiment that gefitinib-resistant cells appeared in 2 months at the earliest when an EGFR exon 19 deletion-positive lung cancer cell line was treated continuously with low-dose gefitinib.^63)^ Thus, in consideration of the period appropriate for suppression of the appearance of resistant cells as well as the cytoreductive effect, we concluded that 8 weeks is appropriate. Also, the period of subsequent suspension of administration was made 2 weeks as adopted in many clinical studies/trials.

#### 4) Regimen of inserted platinum-based combination chemotherapy

1. **Selection of the regimen of platinum-based combination chemotherapy in phase II study**

A regimen with excellent cytoreductive and PFS/OS prolonging effects should be used as chemotherapy to be inserted in gefitinib monotherapy. No data that indicate the superiority of any particular chemotherapeutic regimen for EGFR mutation-positive non-small-cell lung cancer, and it is considered practical to select a regimen more effective than other regimens for advanced non-squamous non-small-cell lung cancer. We select platinum-based combination chemotherapy, which is the first-line standard treatment, particularly, CDDP-based combination therapy, which was suggested by meta-analysis^41)^ to be superior to CBDCA when combined with a third-generation anticancer agent against non-squamous cell carcinoma.

In phase II trials, CDDP+DTX was used as the platinum-based combination chemotherapy. In those days, PEM was not approved as a treatment for lung cancer in Japan. Although no difference was observed in clinical studies that compared combinations of a platinum-based drug and a third generation anticancer drug,^7)8)^ we selected CDDP+DTX for the following reasons.

1. CDDP+DTX was shown by a phase III trial conducted in Japan comparing it with CDDP+vindesine (second-generation regimen) to be superior in OS in non-small-cell lung cancer patients (TAXJP301)^64)^ and is a third-generation regimen directly indicated to be superior to the second-generation regimen.
2. CDDP+DTX showed good OS and better RR and QOL compared with CDDP+VNR, although the differences were not significant, in a phase III trial comparing CDDP+DTX and CBDCA+DTX with CDDP+VNR in non-small-cell lung cancer patients (TAX326),^65)^ and is also a regimen with advantages in comparison with other third-generation regimens.
3. Regimens including DTX showed slightly better OS than those including a vinca alkaloid in meta-analysis comparing chemotherapeutic regimens including DTX and those including a vinca alkaloid in non-small-cell lung cancer patients.^66)^
4. **Selection of the regimen of platinum-based combination chemotherapy in this study**

In this study, CDDP+PEM is used instead of CDDP+DTX for the following reasons.

1. Subgroup analysis of a study comparing CDDP+GEM and CDDP+PEM demonstrated that OS of non-squamous non-small-cell lung cancer patients by CDDP+PEM was better than that by CDDP+GEM (MST 11.8 months by CDDP+PEM vs. 10.4 months by CDDP+GEM, HR=0.81 (95%CI: 0.70-0.94)),^9)^ which was considered comparable to that by CDDP+DTX.^7)^
2. In a clinical study that compared DTX monotherapy and PEM monotherapy as a second-line treatment for advanced non-small-cell lung cancer, PEM also tended to be superior to DTX in the non-squamous subgroup.^36)^
3. The incidence of febrile neutropenia as an adverse event was 11% by CDDP+DTX and 4% by CDDP+GEM but was low at 1.3% by CDDP+PEM, and other hematological toxicities were milder by CDDP+PEM.^7)9)^ Pegfilgrastim, which is a long-acting G-SCF preparation approved in Japan in 2014, has the potential to prevent febrile neutropenia, but the guideline for its use has not been established. EGFR mutation-positive non-squamous cell carcinoma, which is the target disease of this study, tends to occur more frequently in younger generations among lung cancers, and whether pegfilgrastim should be administered for the primary prevention of febrile neutropenia to all patients including younger patients with less risk of severe febrile neutropenia is unclear. Therefore, the incidence of febrile neutropenia mentioned above deserves attention.
4. In clinical practice, CDDP+PEM is used frequently, and the use of CDDP+DTX is decreasing. In a multicenter prospective observational study conducted in Japan from 2010 to 2011, the frequency of selection of CDDP+PEM as the first-line platinum-based combination chemotherapy was 22% in 691 patients with advanced non-squamous non-small-cell lung cancer, but CDDP+DTX was selected in only 6% (CBDCA-based chemotherapy was selected for other patients).^67)^

Regarding the cost, one course of therapy administered to a patient with a body surface area of 1.5 m^2^ is about |\97,000 for CDDP+DTX and about \288,000 for CDDP+PEM, which is more expensive. However, as mentioned above, CDDP+PEM is frequently used today in daily practice, because the effect of CDDP+PEM against advanced non-squamous non-small-cell lung cancer is comparable or superior to that of CDDP+DTX, and because the incidence of adverse events, such as febrile neutropenia, is lower by CDDP+PEM. For this reason, CDDP+PEM was decided to be used in this study as the trial regimen. This has also gained consensus in the JCOG Lung Cancer Group and WJOG Respiratory Organ Group.

Table 2.3.2.b Comparison of adverse events between CDDP+GEM and CDDP+PEM^9)^

| Adverse events  CTCAE ver. 2.0 | CDDP+GEM | | CDDP+PEM | |
| --- | --- | --- | --- | --- |
|  | All grade (%) | ≥Grade 3 (%) | All grade (%) | ≥Grade 3 (%) |
| Neutrophil count decreased | 38.4% | 26.7% | 29.0% | 15.1% |
| Anemia | 45.7% | 9.9% | 33.0% | 5.6% |
| Platelet count decreased | 26.6% | 12.7% | 10.1% | 4.1% |
| Febrile neutropenia | - | 3.7% | - | 1.3% |
| Nausea | 53.4% | 3.9% | 56.1% | 7.2% |
| Vomiting | 35.5% | 6.1% | 39.7% | 6.1% |
| Anorexia | 24.2% | 0.7% | 26.6% | 2.4% |
| Malaise | 44.9% | 4.9% | 42.7% | 6.7% |
| Constipation | 19.5% | 0.4% | 21.0% | 0.8% |
| Diarrhea | 12.8% | 1.6% | 12.4% | 1.3% |
| Sensory neuropathy | 12.4% | 0.6% | 8.5% | 0.0% |
| Taste disorder | 8.9% | - | 8.1% | - |
| Serum creatinine increased | 6.9% | 0.5% | 10.1% | 0.8% |
| Alopecia | 21.4% | - | 11.9% | - |
| Rash/exfoliation | 8.0% | 0.5% | 6.6% | 0. %1 |

Also, it was reported that OS can be prolonged by administering PEM alone as maintenance therapy following CDDP+PEM,^10)11)^ and maintenance therapy using PEM following CDDP+PEM has become a common clinical practice. However, there are no data about gefitinib+PEM maintenance therapy against EGFR mutation-positive lung cancer, and maintenance therapy using these drugs is bound to continue for a long time, causing the cost to increase. Therefore, we considered that the effectiveness of gefitinib+PEM maintenance therapy should, ideally, be validated by a separate study. Moreover, in this study evaluating whether insertion of CDDP+PEM in patients responding to EGFR-TKI improves OS or not as a clinical question, there is concern over the possibility that maintenance therapy with PEM modifies OS due to its effect and makes whether insertion of CDDP+PEM is effective or not unclear. For these reasons, maintenance therapy using PEM is not performed in this study.

1. **Number of courses of platinum-based combination chemotherapy**

In studies that compared 3 or 4 courses and 6 courses of platinum-based chemotherapy as the first-line standard chemotherapy for non-small-cell lung cancer,^68)69)^ since OS was comparable, but toxicity was milder, by the first regimen, 3 or 4 courses is considered optimal. Also, as 3 courses of CDDP+DTX were performed in a previous phase II trial, it was decided also to perform 3 courses of CDDP+PEM.

#### 5) Studies that may affect the continuation of this trial or interpretation of the results

1. **NEJ009 study**

The North East Japan Study Group (NEJSG) is conducting a randomized controlled study comparing gefitinib monotherapy and concomitant combination therapy of gefitinib and CBDCA+PEM in patients with EGFR mutation-positive advanced non-squamous non-small-cell lung cancer (NEJ009, UMIN000006340) preceding this study. There are the 3 major differences between NEJ009 study and this study.

1. **Regimen of platinum-based combination chemotherapy**

NEJ009 study: CBDCA+PEM combination therapy

This study: CDDP+PEM combination therapy

As observed above, CBDCA+PEM is not considered the optimal chemotherapy for the target disease.

1. **Maintenance therapy after platinum-based combination chemotherapy**

NEJ009 study: gefitinib+PEM combination therapy

This study: gefitinib monotherapy

Gefitinib+PEM combination therapy has not been validated by itself. OS may be prolonged by gefitinib+PEM alone without platinum-based combination chemotherapy, and even if the trial therapy in NEJ009 study is effective, it cannot be determined whether the combination with platinum-based chemotherapy is effective, gefitinib+PEM is effective, or both are effective. In addition, cost is a problem that demands consideration. By gefitinib monotherapy, also, median PFS is about 11 months, and the period of maintenance therapy is expected to be prolonged, but long-term treatment by gefitinib/PEM is expensive.

1. **Timing of platinum-based combination chemotherapy**

NEJ009 study: concomitant use from the beginning

(As a result of the preceding randomized phase II trial that compared concomitant combination and alternating administration (NEJ005), no clear difference was observed in efficacy or safety between the two regimens, but concomitant combination therapy was adopted because of its convenience for treatment.)

This study: Platinum-based combination therapy (CDDP+PEM) is inserted after 8-week gefitinib monotherapy with a 2-week non-dosing period.

Because of these differences, this study is considered to contribute to the establishment of a better standard treatment regardless of the results of NEJ009 study and is considered significant. Also, globally, few studies to examine the effectiveness of the combination of EGFR-TKI and chemotherapy have been carried out, and the possibility of change of the standard treatment based on the results of NEJ009 alone is considered low. Furthermore, patient registration for NEJ009 study was ended in 2014, and a 3.5-year observation period is scheduled. Since the registration period for this study is 3 years, the results of NEJ009 are not expected to become available during the registration period for this study.

<Item added in ver. 2.0>

Concerning NEJ009 study, PFS, PFS2 (progression-free survival time until the next treatment in the gefitinib monotherapy group, PFS in the concomitant gefitinib+CBDCA+PEM group), and OS, which were the co-primary endpoints evaluated by the gate-keeping method were reported at the 2018 ASCO Annual Meeting.^80)^ Median PFS was 11.2 months vs. 20.9 months (HR=0.493 (95%CI: 0.391-0.625), median PFS2 was 20.7 months vs. 20.9 months (HR=0.966 (95%CI: 0.766-1.220)), and median OS was 38.8 months vs. 52.2 months (HR=0.695 (95%CI: 0.520-0.927)), respectively, in the gefitinib monotherapy and gefitinib+CBDCA+PEM concomitant combination therapy groups. Thus, PFS was better in the gefitinib+CBDCA+PEM concomitant combination therapy group than in the gefitinib monotherapy group, but PFS2 did not differ between the two groups, and OS tended to be better in the gefitinib+CBDCA+PEM concomitant combination therapy group, but the difference was not significant according to the statistical setups determined in advance.

The results of this study warrant the prospect that gefitinib+CBDCA+PEM concomitant combination therapy will become a treatment option against EGFR mutation-positive advanced non-squamous non-small-cell lung cancer. However, because the incidence of serious adverse events (≥Grade 3) is higher by gefitinib+CBDCA+PEM concomitant combination therapy than by gefitinib monotherapy but is lower by osimertinib monotherapy than by first-generation EGFR-TKI monotherapy, osimertinib monotherapy is considered to be safer than gefitinib+CBDCA+PEM concomitant combination therapy. Moreover, as it is likely that FLAURA study will show that osimertinib monotherapy is more effective than gefitinib monotherapy for prolonging OS, osimertinib monotherapy rather than gefitinib+CBDCA+PEM concomitant combination therapy is expected to become the future standard treatment for EGFR mutation-positive advanced non-squamous non-small-cell lung cancer.

1. **LUX-Lung 7**

A randomized controlled phase II trial comparing gefitinib and afatinib in patients with EGFR mutation-positive advanced pulmonary adenocarcinoma (LUX-Lung 7, NCT01466660) is in progress as an international collaborative clinical trial and registration is scheduled to end in December 2016, this randomized phase II trial alone is unlikely to be sufficient to change the standard treatment. However, should afatinib be shown by LUX-lung 7 to be far more effective than gefitinib, change of the standard treatment is possible.

<Item added in ver. 2.0>

PFS, the primary endpoint in LUX-Lung 7, was 10.9 months and 11.0 months in median values in the gefitinib and afatinib arms, respectively (HR=0.73 (95%CI: 0.57-0.95)), being superior for afatinib.^81)^ However, median OS was 24.5 months and 27.9 months in the gefitinib and afatinib arms, respectively (HR=0.86 (95%CI: 0.66-1.12)), showing no difference.^82)^

Based on these trial results, afatinib monotherapy has become an option as the first-line treatment for EGFR mutation-positive advanced non-small-cell lung cancer. However, since the incidence of serious adverse events (≥Grade 3 by CTCAE version 3.0, including diarrhea and skin disorder) was higher for afatinib than for gefitinib, it is not considered to be preferable to osimertinib, which has fewer serious adverse events than first-generation EGFR-TKI.

1. **ARCHER1050 study <Item added in ver. 2.0>**

ARCHER1050 study is an international collaborative randomized controlled phase III trial that compared gefitinib and dacomitinib, a second-generation EGFR-TKI, in patients with EGFR mutation-positive advanced non-small-cell lung cancer. Primary endpoint was PFS, and the dacomitinib arm was shown to be superior with median PFS being 9.2 months in the gefitinib arm vs. 14.7 months in the dacomitinib arm (HR=0.59 (95%CI: 0.47-0.74)).^83)^ In addition, OS was also superior in the dacomitinib arm, with median OS being 26.8 months in the gefitinib arm vs. 34.1 months in the dacomitinib arm (HR=0.76 (95%CI: 0.582-0.993)).^84)^

From the results of this study, dacomitinib monotherapy is expected to become an option as the first-line treatment for EGFR mutation-positive advanced non-small-cell lung cancer. However, since the incidence of serious adverse events associated with dacomitinib (≥Grade 3 by CTCAE version 3.0, including diarrhea and skin disorder) was higher than that with gefitinib, it is not considered to be preferred to osimertinib with fewer serious adverse events than first-generation EGFR-TKI.

1. **JO25567 study and NEJ026 study <Item added in ver. 2.0>**

JO25567 study (JapicCTI-42569) is a randomized phase II trial that compared erlotinib monotherapy and erlotinib+bevasizumab combination therapy in patients with EGFR mutation-positive advanced non-squamous non-small-cell lung cancer. PFS was the primary endpoint, and the median value was reported earlier to have been 9.7 months in the erlotinib monotherapy arm vs. 16.0 months in the erlotinib+bevasizumab arm, being superior in the latter group (HR=0.54 (95%CI: 0.36-0.79).^85)^ Recently, the results of analysis of OS in this study were reported at the 2018 ASCO Annual Meeting, but median OS was 47.4 months in the erlotinib monotherapy arm vs. 47.0 months in the erlotinib+bevasizumab arm (HR=0.81 (95%CI: 0.53-1.23)), showing no difference between the two groups.^86)^

Also, NEJ026 study (UMIN000017069) is randomized phase III trial using the same standard and trial treatments against EGFR mutation-positive advanced non-small-cell lung cancer as in JO25567 study. Its major results were also reported at the 2018 ASCO Annual Meeting. PFS was the primary endpoint, and the median value was 13.3 months in the erlotinib monotherapy arm vs. 16.9 months in the erlotinib+bevasizumab arm (HR=0.605 (95%CI: 0.417-0.877)), being superior in the latter arm, but there was no report about OS.^87)^

From the results of the above two clinical studies, longer PFS is considered to be obtained by erlotinib+bevasizumab combination therapy than by erlotinib monotherapy, but the OS prolonging effects has not been demonstrated. Therefore, erologinib+bevasizumab combination therapy is considered a treatment option for EGFR mutation-positive advanced non-squamous non-small-cell lung cancer, but the standard treatment is considered to remain osimertinib monotherapy.

#### 6) Treatment inserting CDDP+PEM during osimertinib administration <Item added in ver. 2.0>

As described in detail in 2.2.1.3), osimertinib also showed activity against T790M mutant type EGFR, and a longer PFS was found to be obtained in patients with EGFR mutation-positive advanced/recurrent non-small-cell lung cancer as the first-line treatment compared with first-generation EGFR-TKI. However, it is already known that complete cure cannot be achieved by osimertinib alone and that the tumor acquires resistance. Although the mechanism of resistance to osimertinib has not been entirely elucidated, a study of a small number of patients reported an involvement of activation of collateral channels, such as MET or EGFR, amplification and gene mutations, such as PIK3CA and KRAS, as well as the appearance of new resistant mutations such as C797S similarly to resistance to first-/second-generation EGFR-TKI.^88)^ Also, as mentioned in 2.3.2. 1), there is the possibility that resistance is caused by the presence of wild-type EGFR cells in part of EGFR mutation-positive tumors untreated with gefitinib.^50)^ Moreover, osimertinib, which has weaker inhibitory action on wild-type EGFR than gefitinib, is suspected to share the same mechanism of resistance as that to first-/second-generation EGFR-TKIs. Although data concerning the regimen of inserting CDDP+PEM during the administration of osimertinib are not available, the treatment concept of preventing proliferation of cells unresponsive/resistant to EGFR-TKI in an early stage by performing chemotherapy during treatment with EGFR-TKI is considered to remain unchanged if gefitinib is replaced by osimertinib.

### Summary of risk/benefit balance of standard and trial treatments

The benefits of the trial treatment are as below. Median PFS by the trial treatment in a phase II trial was 19.5 months. According to the results of previous clinical trials, median PFS by gefitinib, which is the standard treatment is 9-11 months, and even with subsequent platinum-based chemotherapy, PFS is prolonged by 4-6 months, with a sum of 13-17 months, so the trial treatment is likely to be superior. If PFS and OS in the phase II trial recur in this study, they are considered great benefits.

Next, the risks of the trial treatment include the following. Since the trial treatment is gefitinib monotherapy plus CDDP+PEM, it is a toxic new. Adverse events associated with the combination of gefitinib and CDDP+PEM (See Table 2.3.2.a) are expected to be larger in number than those associated with gefitinib monotherapy. However, in the trial treatment, there are non-dosing periods of 2 weeks between the end of gefitinib monotherapy to the beginning of CDDP+PEM combination therapy and 3 weeks between the beginning of the third course of CDDP+PEM combination therapy and the resumption of gefitinib administration. They are sufficient washout periods used in clinical practice and other clinical trials, and the possibility of the occurrence of serious adverse events caused by insertion of CDDP+PEM is considered low. A reason for this assumption is that the insertion is not considered to cause additional progression of adverse events or the occurrence of serious adverse events, because while adverse events were also observed in the phase II trial, their incidence was similar to that when gefitinib monotherapy and CDDP+DTX are performed separately.

Also, in this trial treatment, the possibility of progression during the 2-week non-dosing period between the 8-week oral administration of gefitinib and subsequent platinum-based chemotherapy can be a risk. However, no progression was observed during the non-dosing period in the phase II trial. Also, the risk of further progression during platinum-based chemotherapy may also be avoided by switching to gefitinib again. Therefore, the risk associated with the change in treatment during the trial regimen is not expected to be high.

Furthermore, the protocol treatment increases the economic burden of patients because of the addition of CDDP+PEM. However, the trial regimen does not require a new drug or a drug not previously used for the treatment of EGFR mutation-positive advanced non-squamous non-small-cell lung cancer, but it is a combination of existing therapies with change in timing. In addition, many patients who show progression during the standard treatment, i.e., gefitinib monotherapy, are expected to undergo CDDP+PEM as a second-line therapy. Therefore, the total cost of the drugs in the trial treatment arm over the entire course of treatment is expected to be comparable to, or lower than, that in the standard treatment arm.

For the above reasons, the trial treatment is considered to be more toxic than the standard treatment, but its cost through the entire course of treatment is not considered high.

<Item added in ver. 2.0>

As described in detail in 2.2.1.3), in FLAURA study, the incidence of adverse events such as rash and AST/ALT elevation or serious adverse events is lower with osimertinib than with first-generation EGFR-TKI including gefitinib. Therefore, adverse events are not considered to increase with change of the protocol treatment from gefitinib to osimertinib.

The risk of the standard treatment or trial treatment associated with change from EGFR-TKI to osimertinib is considered the same as that in the case of gefitinib mentioned above.

#### Cost of drugs by the standard treatment and trial treatment (in the case of protocol treatment with a body surface area of 1.5 m^2^ and a duration of 20 months*）

- 20 months was used for tentative calculation, because the median PFS by the trial treatment was 19.5 months in the phase II trial.

Patients registered before or in ver. 1.1 are administered gefitinib, and those registered in or after ver. 2.0 are administered osimertinib.

**Patients registered before or in Ver. 1.1**

**Standard treatment**

Gefitinib 250 mg/day × 11 months + (cisplatin 112.5 mg + pemetrexed 750 mg) × 4 courses (3 months) + (maintenance therapy with pemetrexed 750 mg) × 4 courses (3 months) + (docetaxel 90 mg) × 4 courses (3 months) =4,968,727.6 yen

**Standard treatment**

Gefitinib 250 mg/day × 20 months =3,759,112.0 yen

**Trial treatment**

Gefitinib 250 mg/day × 2 months + (cisplatin 112.5 mg + pemetrexed 750 mg) × 3 courses (2.25 months) + gefitinib 250 mg/day × 15.25 months =4,235,108.1 yen

- Drug princes (as of May 2015)

Gefitinib 250 mg (Iressa tablets 250) \6,712.7/tablet

Cisplatin 50 mg 100 mL (generic) \5,236

Pemetrexed 500 mg (Alimta injection 500 mg) \185,374

Docetaxel 80 mg (Onetaxotere i.v. infusion 80 mg/4 mL) \59,156

<Item added in ver. 2.0>

**Patients registered in or after ver. 2.0**

**Standard treatment**

Osimertinib 80 mg/day × 20 months =13,402,256.0 yen

**Trial treatment**

Osimertinib 80 mg/day × 2 months + (cisplatin 112.5 mg + pemetrexed 750 mg) × 3 courses (2.25 months) + osimertinib 80 mg/day × 15.25 months =12,552,319.8 yen

- Drug prince (as of July 2018)

Osimertinib 80 mg (tagrisso tablets 80 mg) 23,932.6 yen/tablet

### Second-line treatment

#### 1) Second-line treatment in the standard treatment arm

At the time of progression, standard options are platinum-based combination therapy, such as CDDP+PEM (+maintenance therapy using PEM), if the general condition is satisfactory and monotherapy with a third-generation anticancer agent if the general condition is not expected to tolerate the concomitant use of platinum-based drugs. In routine clinical practice, if only bone metastasis or brain metastasis appear newly, so-called beyond PD, i.e., performing radiation therapy against them while continuing treatment with gefitinib or osimertinib, is also performed. Therefore, we do not restrict second-line treatments including continuation of gefitinib or osimertinib and other EGFR-TKI. Even when the trial is discontinued due to adverse events, second-line treatment is not restricted, because switching to another EGFR-TKI, platinum-based combination therapy, and monotherapy using a third-generation anticancer agent are possible options.

#### 2) Second-line treatment in the trial treatment arm

In the event of progression, DTX monotherapy is the first-choice standard treatment, but there is also no restriction about other treatment options. In case of progression during the initial gefitinib treatment, a standard treatment is platinum-based combination therapy if the general condition is satisfactory and monotherapy with a third-generation anticancer agent if the general condition is not expected to tolerate platinum-combination therapy. There is no restriction on second-line treatment including continuation of gefitinib or osimertinib as beyond PD and other EGFR-TKIs. If the trial is discontinued due to adverse events, there is also no restriction, because switching to other EGFR-TKIs as well as treatments for adverse events can be options.

## Trial Design

### Rationale for the selection of endpoints

This study is a phase III trial, and OS was selected as the true primary endpoint. Therefore, in this study, the superiority of the trial regimen, i.e., administering gefitinib alone for 8 weeks, followed by 3 courses of CDDP+PEM combination therapy, then performing gefitinib monotherapy again, to the standard regimen, i.e., gefitinib monotherapy in terms of OS in patients with EGFR mutation-positive advanced non-squamous non-small-cell lung cancer is examined. In this study, the standard regimen, gefitinib monotherapy, is one treatment, and platinum-based combination chemotherapy is likely to be performed as second-line treatment after progression. In contrast, the trial regimen is a combination of two treatments, i.e. gefitinib and platinum-based combination chemotherapy, the other endpoints, such as PFS, are not considered optimal for the evaluation of the usefulness of the treatment, hence OS was selected as the primary endpoint.

As secondary endpoints, PFS, response rate, incidence of adverse events, incidence of serious adverse events, and frequency of EGFR exon 20 T790M-resistant point mutation in tumors at the time of progression were selected. It is becoming a clinical routine to perform biopsy again at the time of progression to check the presence or absence of exon 20 T790M-resistant point mutation. Also, as next-generation EGFR-TKIs that are also effective against exon 20 T790M-resistant point mutation are expected to be put into clinical use in the future, it is considered important to check the presence or absence of exon 20 T790M-resistant point mutation by performing biopsy again at the time of progression. However, at present, it is not practical to require repeat biopsy at the time of progression. Therefore, repeat biopsy is not required as essential but is recommended as much as possible, and the positive rate of exon 20 T790M-resistant point mutation in repeat biopsy samples at the time of progression is selected as a secondary endpoint. It was stipulated that repeat biopsy at the time of progression be performed during the period from progression to the initiation of the subsequent chemotherapy (except that repeat biopsy is performed during the period until the initiation of chemotherapy other than gefitinib if gefitinib is also continued after the diagnosis of progression) without specifying the site or number of biopsies. For this purpose, EGFR gene mutation may be tested by a method other than the one at the time of registration on condition that it is listed in “4.1. Eligibility criteria (enrollment criteria)”.

<Item added in ver. 2.0>

Until now, in clinical practice, repeat biopsy was performed at the time of progression after initial treatment using first/second-generation EGFR-TKI, such as gefitinib, the presence or absence of EGFR exon 20 T790M-resistant point mutation was examined, and, if positive, osimertinib was the first choice, but the significance of checking T790M mutation at the time of progression was lost as the shift of the standard first-line treatment to osimertinib. Therefore, the frequency of EGFR exon 20 T790M-resistant point mutation in tumor at the time of progression, which is a secondary endpoint, is not calculated in patients administered osimertinib, and it is calculated only in the group administered gefitinib. However, additional study to explore the mechanism of resistance to osimertinib by performing biopsy again at the time of progression is important, and implementation of repeat biopsy at the time of progression as much as possible remains to be recommended.

### Clinical hypothesis and rationale for the number of patients to be registered

The primary research hypothesis of this study is: “The overall survival period of the trial regimen (after administering gefitinib for 8 weeks, performing 3 courses of cisplatin • pemetrexed combination chemotherapy, and administering gefitinib again) arm surpasses that of the standard regimen (gefitinib monotherapy) arm”, and the trial regimen is judged to be more useful if this hypothesis is validated.

The 3-year survival rate in the standard regimen arm is assumed to be 45% from the results of gefitinib monotherapy in NEJ002 study.^16)17)^ Also, from the single-center phase II trial performed at National Cancer Center Hospital, the trial regimen in this study is expected to increase the 3-year survival rate by 10%, and the 3-year survival rate in the trial regimen arm is set at 55% from the results of NEJ002. The primary research hypothesis of this study is that the overall survival period, which is a primary endpoint, is superior in arm B treated by the trial regimen (administering gefitinib for 8 weeks, followed by 3 courses of cisplatin-pemetrexed combination chemotherapy, then administering gefitinib again) compared with arm A treated by the standard regimen (gefitinib monotherapy), and cisplatin-pemetrexed combination therapy is concluded to be more useful if this hypothesis is validated. If the superiority of the trial regimen is not significant, it is concluded that the standard regimen (gefitinib monotherapy) continues to be a useful treatment.

With the assumptions of the level of significance on one-tailed test being α=0.05, power of test being 80%, registration period being 2.5 years, and follow-up period being 3 years, the necessary number of participants is 482 with the two arms combined. By expecting a small number of losses, 250 participants in each arm with a total of 500 participants with the two arms combined was set as the target number of registrations (See “12.2. Target number of registrations, registration period, follow-up period”). However, the registration period was set at 3 years in consideration of the time needed to complete the procedures at the institutional review boards.

<Item added in ver. 2.0>

As described in detail in “2.2.2. Standard treatment regimen” and “2.3.2. 6) Regimen of inserting CDDP+PEM therapy during the administration of osimertinib”, the standard regimen in this study was changed to osimertinib monotherapy, and the trial regimen to insertion of cisplatin+pemetrexed during the administration of osimertinib. Therefore, the clinical hypothesis is changed to “The overall survival period in the trial regimen (after administering gefitinib or osimertinib for 8 weeks, performing 3 courses of cisplatin-pemetrexed combination therapy, then administering gefitinib or osimertinib again) arm is superior to that in the standard regimen (gefitinib or osimertinib monotherapy) arm”. Acquired resistance is also known to develop with osimertinib, and its mechanism is estimated to an extent to be the same as that of resistance to first/second-generation EGFR-TKI such as gefitinib. Therefore, the concept of treatment, i.e., preventing proliferation of EGFR-TKI-unresponsive or resistant cells in an early stage by performing chemotherapy during treatment with EGFR-TKI, is considered to remain unchanged even with change of EGFR-TKI used in the study from gefitinib to osimertinib.

However, the greatest problem with the substitution of gefitinib for osimertinib in this revision is handling treatment with different drugs as a single treatment while both drugs belong to the same family of drugs (EGFR-TKI), and if there is drug interaction between the drug and therapeutic effect, i.e., if the results concerning the overall survival period differ between the gefitinib cohort and osimertinib cohort, there is the possibility that a valid conclusion is not obtained by the study as a whole.

Should the results be that there is a difference in the overall survival period in one cohort (more likely to occur in the gefitinib cohort with a larger number of registrations) but that there is no difference in the other cohort (more likely to occur in the osimertinib cohort), the conclusion, “Insertion of cisplatin+pemetrexed combination therapy is useful” cannot be applied to both cohorts (particularly to the cohort of osimertinib, which will become the standard treatment in the future). Also, should a clinically significant difference be observed in one cohort, and sample size of this cohort is not sufficient to confirm the statistical significance of the difference, leading to failure in obtaining a confirmatory conclusion. Therefore, by the present revision of changing gefitinib to osimertinib, this study is considered to become a significant research only if there is no interaction between the drug and therapeutic effect.

However, we consider that there is no interaction between the drug and therapeutic effect for the following reasons. It has been known that exon 20 T790M point mutation is involved in about 50% of the molecular biological mechanisms of acquired resistance to gefitinib, and the prognosis of tumors with resistant gene including this mutation has been expected to be improved by insertion of cisplatin+pemetrexed combination therapy. Osimertinib is also known to act on exon 20 T790M point mutation, and, if the drug is changed to osimertinib, there is the possibility that it mitigates resistance due to exon 20 T790M point mutation reduces the effect of cisplatin+pemetrexed combination therapy. However, there are also the remaining about 50% of the mechanisms and unknown mechanisms of resistance, and the effect of cisplatin+pemetrexed combination therapy is not considered to be nullified. Therefore, from a medical viewpoint, quantitative interaction (some difference in therapeutic effect) may occur between the cohorts, the possibility of the occurrence of qualitative interaction (difference or reversal of therapeutic effect) is considered small.

Moreover, statistically, the possibility of the occurrence of qualitative interactions due to the smallness of the number of participants is considered small. Specifically, the predictive probability of the test of primary hypothesis in all registered patients at the time of primary analysis becoming significant and the hazard ratio in the osimertinib cohort surpassing 1 is calculated to be 9.3%, and the predictive probability calculated in the gefitinib cohort becomes nearly 0%.

Although there is a sufficient probability of the occurrence of quantitative interactions, if the interactions remain quantitative (OS is superior in the trial regimen arm in both cohorts), it is considered possible to extrapolate the results in all participants to both cohorts if the results of primary analysis are significant. On the other hand, if a large difference is observed in either cohort, but the difference is not significant in all participants, the conclusion of the study will be interpreted as negative.

In addition, as mentioned in the addition 12.2.2. to the protocol, efforts were made to enhance the scientific validity as much as possible also in the osimertinib cohort after revision by calculating the number of events in the osimertinib cohort necessary to make the probability of the judgment that the results are consistent ≥70% based on observation of a therapeutic effect at least 50% or higher of the therapeutic effect of the entire study and setting the target number of registrations at 174 to meet the calculated number of events, i.e., 36.

Of course, to be exact, it is the optimal choice to discontinue this study in this stage and perform a new study in the osimertinib cohort alone. However, in the present circumstances in which the standard treatment for non-small-cell lung cancer is replaced one after another with the development of novel drugs, we aim to find an answer by a study involving 500 participants as initially planned concerning the significance of insertion of cisplatin+pemetrexed, which is a clinical question of this study. Also, as we wish to use the data of the gefitinib cohort followed up to the present, the setup of the study was determined as mentioned above in consideration of the feasibility and after discussion in the JCOG lung cancer group.

The specific number of registrations after the change in the study design was discussed in team meetings (September and December 2017 and March 2018) within the JCOG lung cancer group since the results of FLAURA study were presented at ESMO in 2017. Below are the decisions made. (However, since the time of expansion of indications of osimertinib was unknown at the time of the team meetings, attention to some inconsistency from the number of registrations decided in ver. 2.0.)

- As of March 2018, the time of expansion of indications of osimertinib was expected to be January 2019, the target number of registrations was set on the assumption that half a year is needed for the procedure of IRB from protocol revision and that registration is suspended during this period. The pace of registration was estimated from that until January 2018 to be 10 patients/month. Also, since the 18-month survival rate in FLAURA study was 83% in the osimertinib arm, the 3-year survival rate in arm A of the osimertinib cohort of this study was assumed to be 68.9%. The hazard ratio was expected to be 0.749 similarly to the gefitinib cohort (3-year survival rate in arm B: 75.6%).
- Based on this setup, the time of completion of registration of 500 participants as planned at designing of the study is tentatively calculated to be August 2020, and the number of events at the time of primary analysis to be performed after 3-year follow-up as initially planned (August 2023) to be 302, with the prospect of achieving the planned number of events (297).
- However, multiple competitive studies concerning osimertinib combination therapies and new drugs are expected to be planed and implemented in the subject group of this study. It is certainly important to accumulate a sufficient number of events, but it is also important to report major results of this study before the standard treatment is substituted. As a result of comparison and evaluation of multiple patterns of setting of the number of registration in JCOG lung cancer group, it was agreed within the group to advance the time of major analysis to about February 2022 (by keeping the target number of registrations at 500) despite sacrifice of some loss of the power of test.

As mentioned above, the target number of registration is kept at 500 even with change of the base drug to osimertinib. Since the time of expansion of the indication of osimertinib, time needed for protocol revision, and time until approval by IRB cannot be accurately estimated, some misestimation of the actual number of events or power of test is inevitable. However, if registration of the osimertinib cohort can be started in October 2018, registration of 500 participants (consisting of 326 patients in the gefitinib cohort and 174 patients in the osimertinib cohort) is expected to be completed by the end of March 2020. Should primary analysis be performed in February 2022, a total of 256 events (consisting of 214 and 42 events in the gefitinib and osimertinib cohorts, respectively) are expected to be accumulated, and the power of test of all registered patients at the 5% level of significance on one-tailed test is expected to be 74.8%. Should the beginning of registration of the osimertinib cohort is delayed a few months from October 2018, the follow-up period of the osimertinib cohort is shortened by a few months compared with the case in which registration can be started in October 2018. This delay may be accompanied by a slight decrease in the number of events, but its effect on the power of test is very small.

In this study, as mentioned above, cases in which the effectiveness of the trial regimen varies according to the base drug (i.e., there is interaction) are excluded, and all registered patients are defined as the main analysis set. If there are interactions, the effectiveness of the trial regimen is evaluated in each drug cohort (gefitinib cohort/osimertinib cohort). Discussion about the setting of the number of registrations in this case is presented in detail in <Item added in ver. 2.0> of “12.2. Target number of registrations, registration period, follow-up period”.

### Collaborative trial by JCOG and WJOG

This study is carried out as a collaboration of JCOG and WJOG. Registration, data collection, and preparation of periodic reports are made at their respective data centers, but, in interim analysis, major analysis, and final analysis, information collected at each data center are integrated at the JCOG data center and analyzed as one trial. This study is defined exclusively as a single trial, and it is not continued in one group alone should its discontinuation become necessary in either group. Some of the institutions belonging to the JCOG lung cancer group also belong to the WJOG respiratory organ group, and they participate in this study as JCOG institutions.

<Item added in ver. 2.0>

After the present revision, there will be no new registrations from WJOG. This is a measure taken as WJOG respiratory organ group meeting judged, despite wishes of many WJOG institutions to continue registering patients also in the osimertinib cohort, that disadvantages of the continuation of new registrations are greater, because the cost associated with the present revision (including the preparation of a registration system and a database) is expected reach a large sum.

Originally, this study was designed as a collaboration of JCOG and WJOG partly for promoting patient registration, but WJOG will stop new registrations in the osimertinib cohort due to the above compelling circumstances. Since about 60% of the target number of registrations has already been reached, and since it is preferred to use the data obtained to the present from patients in the gefitinib cohort registered also by WJOG, registration in the osimertinib cohort will be continued in JCOG alone in consideration of feasibility. Since the protocol treatment and subsequent follow-up are planned to be performed as before in the gefitinib cohort registered before ver.1.1 at the institutions that belong to WJOG, there will be no change in the systems for reporting of adverse events, management of COI, monitoring, and inspection or the roles of WJOG Study Coordinator and Principal Investigator.

### Prospects of patient registration

No previous study in the same patients has been conducted by the JCOG lung cancer group. There are about 4,000 new patients with stage IV non-small-cell lung cancer annually at all institutions belonging to the JCOG lung cancer group. If 30% of them are assumed to be positive for EGFR gene mutation and fulfill the eligibility criteria, the number of eligible patients is estimated to be about 1,200 annually. Even if the percentage of consenters is assumed to be 50%, registration of about 600 cases annually is considered possible. Since cases of postoperative recurrence as well as stage IV cases can be registered, registration of about 150 patients annually is considered to be highly likely even in consideration of possible competition with other trials. From JCOG1210/WJOG7813L “Phase III randomized controlled trial of docetaxel monotherapy and carboplatin/pemetrexed combination therapy followed by pemetrexed maintenance therapy in older patients with advanced non-squamous non-small-cell lung cancer”, which precedes this study, the number of patient registrations including WJOG is expected to be 1.6 times higher, being about 240 annually.

<Item added in ver. 2.0>

As mentioned in 2.4.3., there will be no new registrations from WJOG after the present revision. Furthermore, some institutions belonging to JCOG may also stop registration of new patients from the osimertinib cohort. However, after the switch of the standard treatment to osimertinib, it is expected that patient registration in “multiple other clinical trials with the same subjects as this study” will stop and registration in this study will be accelerated. Together, we expect the number of eligible patients to remain at a previous level and specifically be about 10 patients per month similarly to the pace before the revision.

### Rationale for the setting of the allocation adjustment factors

Random allocation is made by minimization to adjust ➀ facility, ➁ clinical stage (stage IIIB and IV vs. postoperative recurrence), ➂ sex (male vs. female), and ➃ EGFR gene mutation (exon19 partial deletion vs. exon21 L858R point mutation) to avoid large bias in them.

#### 1) Institution

Inter-center variation is widely known to exist in the background of registered patients, treatment, efficacy evaluation, and safety evaluation, and adjustment for the institution is a standard of the JCOG.

#### 2) Clinical stage (stage IIIB and IV vs. postoperative recurrence)

Clinical stage is a prognostic factor of non-small-cell lung cancer. According to retrospective analysis by National Cancer Center Hospital, MST was 13.3 months in stage IV patients who underwent chemotherapy but was 21.3 months, i.e., a better prognosis, in those who had postoperative recurrence.^27)^ Also, in a phase III trial of gefitinib and CDDP+DTX in patients with EGFR mutation-positive advanced or postoperative recurrent non-small-cell lung cancer carried out in Japan, MST was 27.5 and 38.8 months, respectively, in those with advanced disease (stage III untreatable by radiation therapy and stage IV) (101 patients) but was more favorable, being 42.7 and 47.6 months, respectively, in those with postoperative recurrence (71 patients).^15)^ In addition, in a retrospective study in 496 patients with EGFR mutation-positive lung cancer conducted in the United States, MST was 30 months in stage IV patients (366 patients) but was 32 months in those with postoperative recurrence (130 patients) (HR=1.36, 95%CI: 1.05-1.76, p=0.019).^28)^ Therefore, stage IIIB and IV vs. postoperative recurrence is made an allocation adjustment factor.

#### 3) Sex (male vs. female)

Sex has long been known as a prognostic factor of advanced non-small-cell lung cancer, and females are often reported to have a better prognosis than males. According to multivariate analysis in a retrospective study involving 3,455 Japanese patients with advanced non-small-cell lung cancer, sex was a prognostic factor along with PS, age, stage, and smoking history (prognosis was better for females with HR=0.747, 95%CI: 0.677-0.825, p<0.0001).^29)^ When the target is restricted to EGFR mutation-positive lung cancer, no difference in prognosis was observed according to sex in the above retrospective analysis in the United States, but the PFS-prolonging effect of EGFR-TKI was higher in females than in males according to the above meta-analysis integrating 7 phase III trials comparing EGFR-TKI and platinum-based combination chemotherapy (HR against chemotherapy in females=0.33, HR against chemotherapy in males=0.45, P_interaction_=0.02).^26)^ In this study, sex is made an allocation adjustment factor in consideration of previous reports and results of meta-analyses about all non-small-cell lung cancers.

#### 4) EGFR gene mutation (exon19 partial deletion vs. exon21 L858R point mutation)

Of the EGFR gene mutations, prognosis may be more favorable with exon 19 partial deletion, which is a major mutation, than with exon 21 L858L point mutation. Concerning comparison between exon 19 partial deletion and exon 21 L858R point mutation, there are not only reports of clinical trials using gefitinib against advanced lung adenocarcinoma that no difference was observed between them in efficacy or prognosis^14)16)^ but also a report concerning treatment using gefitinib or erlotinib that RR, PFS, and OS were better in lung cancer with exon 19 partial deletion than in lung cancer with exon 21 L858R point mutation.^25)^ This study, which retrospectively compared 36 patients with lung cancer with EGFR exon 19 partial deletion or with exon 21 L858R point mutation treated with gefitinib or erlotinib, showed that the outcome was better in the group with exon 19 partial deletion than in the group with exon 21 L858R point mutation with RR of 73% vs. 50%, PFS of 24 months vs. 10 months, and OS of 38 months vs. 17 months, respectively. Also, according to the analysis integrating LUX-Lung3 (307 patients) and LUX-Lung6 (324 patients) comparing afatinib and platinum-based combination chemotherapy in patients with EGFR-mutation-positive advanced lung adenocarcinoma (631 patients), OS was better in the afatinib arm (236 patients) than in the combination chemotherapy arm (119 patients) (31.7 months vs. 20.7 months, HR=0.59, 95%CI: 0.45-0.77, p=0.0001) in the exon 19 partial deletion group (355 patients) but showed no difference between the afatinib arm (183 patients) and chemotherapy arm (93 patients) (22.1 months vs. 26.9 months, HR=1.25, 95%CI: 0.92-1.71, p=0.1600) in the exon 21 L858R point mutation group.^23)^ Moreover, according to the meta-analysis integrating 7 phase III trials that compared EGFR-TKI and platinum-based combination chemotherapy as a first-line treatment, the PFS-prolonging effect of EGFR-TKI was better in lung cancer with exon 19 partial deletion than in lung cancer with exon 21 L858R point mutation (HR against chemotherapy in lung cancer with exon 19 partial deletion=0.24, HR against chemotherapy in lung cancer with exon 21 L858R point mutation=0.48, P_interaction_<0.001).^26)^

For the above reasons, exon 19 partial deletion vs. exon 21 L858R point mutation is made an allocation adjustment factor. There has been no report of coexistence of exon 19 partial deletion and exon 21 L858R point mutation.

## Summary of advantages and disadvantages expected from the participation in the trial

### Expected advantages

If better OS/PFS is obtained in the trial regimen arm than in the standard regimen arm as mentioned above, it would be a major benefit.

All drugs used in this trial are approved to be used for the treatment of the target of this trial under coverage of national health insurance. Also, as the cost of medical services including the cost of drugs used for the patients participating in the trial during the trial period is paid by the patients themselves, there is no special economic benefit given to the patients by their participation in the trial compared with usual clinical practice.

### Expected risks and disadvantages

The risks mentioned in “2.3.3. Summary of risk/benefit balance of standard and trial treatments” are the risks/disadvantages expected in this study. Since the regimen in the trial treatment arm consists of gefitinib monotherapy with the addition of CDDP-PEM, the types of adverse events that may occur during the protocol treatment increase accordingly.

To minimize the risks of adverse events and disadvantages, matters including “4. Patient selection criteria”, “6.3. Treatment modification criteria”, and “6.4. Concomitant treatment and supportive care” are carefully evaluated within the group. Also, in JCOG clinical trials, periodic monitoring 2 times a year is mandated after the beginning of a trial, and whether the occurrence of adverse events is in the expected range is monitored by the data center and Data and Safety Monitoring Committee, and, in case of the occurrence of serious or unexpected adverse events, a system to carefully evaluate and inspect them according to the JCTN Guideline for Reporting of Adverse Events, JCOG Guideline for the Handling of Clinical Safety Information, and various related rules and to take necessary measures is established.

## Significance of this trial

If, in this study, the superiority in terms of OS of the trial regimen, i.e., administering gefitinib alone for 8 weeks, followed by 3 courses of CDDP+PEM combination therapy after a 2-week no-dosing period, then administering gefitinib alone, compared with the standard regimen, i.e., gefitinib monotherapy, is validated in patients with EGFR mutation-positive advanced non-squamous non-small-cell lung cancer, the trial regimen becomes a new standard treatment for EGFR mutation-positive advanced non-squamous non-small-cell lung cancer. This leads to improvements in the prognosis of a large population accounting for 30-50% of advanced non-small-cell cancers in Japanese. In addition, as mentioned above, the trial regimen does not use a new drug or a drug that has not been used for patients with EGFR mutation-positive advanced non-squamous non-small-cell lung cancer but is a combination of existing treatments and change in timing of their use. Therefore, the trial regimen does not increase the economic burden of patients throughout the course of treatment and improves the cost-effectiveness of treatment.

However, if the study fails to validate the superiority of the trial regimen, conventional gefitinib monotherapy remains the standard treatment, and the correctness of the existing therapeutic strategy performing platinum-based combination chemotherapy before and after, rather than simultaneously with, gefitinib monotherapy is confirmed.

<Item added in ver. 2.0>

As described in detail in “2.2.2. Standard treatment regimen” and “2.3.2. 6) Regimen of inserting CDDP+PEM therapy during the administration of osimertinib”, the standard and trial treatments in this study have been changed to osimertinib monotherapy and cisplatin+permetrexed inserted during the administration of osimertinib, respectively. For this reason, if the superiority in terms of OS of the trial treatment of administering gefitinib or osimertinib, followed by 3 courses of CDDP+PEM combination therapy after a 2-week no-dosing period, then administering gefitinib again to the standard treatment of administering gefitinib or osimertinib alone is validated by this study, the trial treatment becomes a new standard treatment for EGFR mutation-positive advanced non-squamous non-small-cell lung cancer.

## Associated studies (including sample analysis studies)

Protocols are being evaluated with plans to perform associated studies using tumor and blood samples.

<Item added in ver. 2.0>

JCOG1404A1 (biomarker study to analyze the mechanism of acquisition of resistance to EGFR tyrosine kinase inhibitors and to evaluate the usefulness of liquid biopsy) is underway as an associated study, but the protocol of JCOG1404A1 is scheduled to be revised with the protocol revision of this trial.

## JCOG biobank collaborating with BioBank Japan (BBJ) (JCOG-BBJ biobank)

Since studies associated with this trial using tumor and blood samples are planned to be performed, this trial does not participate in banking of blood samples (DNA/plasma) in the JCOG-BBJ biobank based on the common protocol of all JCOG trials.

# Criteria and definitions used in this study

Clinical staging is according to the TNM classification by the UICC, 7th edition. Pathological classification is based on the WHO Classification of Lung Cancer, Seventh Edition, 1999.

## Clinical stage classification （UICC-TNM 7^th^ edition）

**T- Primary Tumor**

| TX | Primary tumour cannot be assessed, or tumour proven by the presence of malignant cells in sputum or bronchial washings but not visualized by imaging or bronchoscopy |
| --- | --- |
| T0 | No evidence of primary tumour |
| Tis | Carcinoma *in situ* |
| T1 | Tumour < 3 cm in greatest dimension, surrounded by lung or visceral pleura, without bronchoscopic evidence of invasion more proximal than the lobar bronchus (i.e., not in the main bronchus) |
| T1a | Tumour < 2 cm in greatest dimension |
| T1b | Tumour > 2 cm but < 3 cm in greatest dimension |
| T2 | Tumour > 3 cm but < 7 cm or tumour with any of the following features (T2 tumours with these features are classified T2a if < 5 cm): |
|  | Involves main bronchus, > 2 cm distal to the carina |
|  | Invades visceral pleura |
|  | Associated with atelectasis or obstructive pneumonitis that extends to the hilar region but does not involve the entire lung |
| T2a | Tumour > 3 cm but < 5 cm in greatest dimension |
| T2b | Tumour > 5 cm but < 7 cm in greatest dimension |
| T3 | Tumour > 7 cm or one that directly invades any of the following: |
|  | Chest wall (including superior sulcus tumours), diaphragm, phrenic nerve, mediastinal pleura, parietal pericardium |
|  | Tumour in the main bronchus < 2 cm distal to the carina but without involvement of the carina |
|  | Associated atelectasis or obstructive pneumonitis of the entire lung |
|  | Separate tumour nodule(s) in the same lobe |
| T4 | Tumour of any size that invades any of the following: |
|  | Mediastinum, heart, great vessels, trachea, recurrent laryngeal nerve, esophagus, vertebral body, carina |

**N- Lymph nodees**

| NX | | Regional lymph nodes cannot be assessed | |
| --- | --- | --- | --- |
| N0 | | No regional lymph node metastasis | |
| N1 | | Metastasis in ipsilateral peribronchial and/or ipsilateral hilar lymph nodes and intrapulmonary nodes, including involvement by direct extension | |
| N2 | | Metastasis in ipsilateral mediastinal and/or subcarinal lymph node(s) | |
| N3 | | Metastasis in contralateral mediastinal, contralateral hilar, ipsilateral or contralateral scalene, or supraclavicular lymph node(s) | |
|  |  | |

**M- Metastasis**

| MX | Distant metastasis cannot be assessed |
| --- | --- |
| M0 | No distant metastasis |
| M1 | Distant metastasis |
| M1a | Separate tumour nodule(s) in a contralateral lobe |
|  | tumour with pleural nodules or malignant pleural/ pericardial effusion |
| M1b | Distant metastasis |

表3.1. Clinical stage classification （The subject of this study is the shaded area）

| Cryptogenic | TX | N0 | M0 |
| --- | --- | --- | --- |
| Satge 0 | Tis | N0 | M0 |
| Stage IA | T1a or T1b | N0 | M0 |
| Stage IB | T2a | N0 | M0 |
| Stage IIA | T1a or T1b | N1 | M0 |
|  | T2a | N1 | M0 |
|  | T2b | N0 | M0 |
| Stage IIB | T2b | N1 | M0 |
|  | T3 | N0 | M0 |
| Stage IIIA | T1a or T1b | N2 | M0 |
|  | T2a or T2b | N2 | M0 |
|  | T3 | N1 | M0 |
|  | T3 | N2 | M0 |
|  | T4 | N0 | M0 |
|  | T4 | N1 | M0 |
| Stage IIIB | AnyT | N3 | M0 |
|  | T4 | N2 | M0 |
| Stage IV | AnyT | AnyN | M1a or M1b |

## Pathological classification

The subject of this study is the shaded area.

1. Squamous cell carcinoma
2. Small cell carcinoma

Combined small cell carcinoma

1. Adenocarcinoma
2. Large cell carcinoma

Large cell neuroendocrine carcinoma

Combined large cell neuroendocrine carcinoma

Basaloid carcinoma

Lymphoepithelioma-like carcinoma

Clear cell carcinoma

Large cell carcinoma with rhabdoid phenotype

1. Adenosquomous carcinoma
2. Carcinoma with pleomorphic、 sarcomatoid or sarcomas elements
3. Carcinoid tumors
4. Carcinomas of salivary-gland type
5. Unclassified carcinoma

# Patients Selection Criteria

For inclusion in the study, patients must fulfill all of the following eligibility criteria and they are excluded if they meet any of the following exclusion criteria.

Eligibility criteria （inclusion criteria）

1) Histologically or cytologically confirmed non-squamous non-small cell lung cancer (either adenocarcinoma, large cell carcinoma (excluding large cell neuroendocrine carcinoma), or non-small cell lung cancer without identifiable histologic type) (see 3.2. Tissue Classification).

2) Radical non-radiotherapy stage IIIB stage /IV, or postoperative recurrence.

3) Gene mutations in tissue or cell samples meet all of the following.

① There is a exon 19 partial deletion or exon 21 L858R point mutation of EGFR genetic ^※^

② EGFR gene ^※^ has no exon 20 T790M point mutation (but exon 20 T790M point mutation positive for EGFR gene ^※^ is acceptable if osimertinib is used).

③ If KRAS gene is identified prior to enrollment, there is no KRAS gene mutation (but identification of KRAS gene mutation is not mandatory)

※ EGFR mutation testing should be done in one of the following ways (but not in Liquid biopsy):

① Real-time PCR assay using COBAS®EGFR mutation detection kit.

② Real-time PCR method using therascreen® EGFR mutation detection kit.

③ PNA-LNA clamp method

④ PCR Invader method

⑤ Cycleave method

⑥ PCR-RFLP method

⑦ Loop-Hybrid method

⑧ Oncommine Dx Target Test Multiple CDx System

4) Age at enrollment date is 20 years or older and 74 years or younger.

5) Performance status (PS) is 0 or 1 according to ECOG criteria (PS must be listed in the medical record).

6) With or without measurable disease.

7) No symptomatic brain metastases, meningeal carcinomatosis, or spinal metastases requiring radiotherapy or surgery.

8) Neither superior vena cava syndrome nor pericardial effusion, pleural effusion, or ascites are Grade 3 or above.
Pleural effusion may be registered if there is no pleural effusion ≧ Grade 3 14 d after drainage followed by pleurodesis.

9) No history of surgery with organ resection within 28 days before enrollment. However, patients who undergo surgery for cytology or biopsy or open thoracotomy will be eligible if they are 14 days or more after surgery.

10) No palliative radiotherapy for lung cancer metastases within 14 days prior to enrollment (eligible if palliative radiotherapy had been completed before 15 days prior to enrollment).

11) Systemic chemotherapy or definitive thoracic radiotherapy, including treatment for other cancer types, has not been performed (however, postoperative chemotherapy with UFT or S-1 after lung cancer surgery can be registered if the withdrawal period until the date of enrollment is at least 4 weeks. Combination chemotherapy with cisplatin after lung cancer is available for at least 48 weeks (336 days) from the date of last chemotherapy treatment (relapse on the same day of the week after 48 weeks of last treatment can be registered), and surgery/hormonal therapy for other cancer types will be eligible).

12) The most recent test value within 14 days before enrollment (the same day of the week two weeks before enrollment is acceptable) meets all of the following.

① Neutrophil count ≧1500 per mm^3^

② Platelet count≧10×10^4^ / mm^3^

③ Total Bilirubin≦1.5 mg/dL

④ AST(GOT)≦100 U/L

⑤ ALT(GPT)≦100 U/L

⑥ Serum creatinine≦1.2 mg/dL

⑦ Creatinine clearance≧60 mL/min

If the estimated value is less than 60 mL/min, it is eligible if it is confirmed that the observed value is more than 60 mL/min by the 24-hour urine collection method.

Cockcroft-Gault formula

Male:Ccr={(140-age)×body weight (kg)}/{72×serum creatinine level (mg/dL)}

Women: Ccr=0.85×{(140-age)×body weight (kg)}/{72 × serum creatinine level (mg/dL)}

⑧ Indoor air SpO_2_≧92%
However, if SpO_2_<92%, it is eligible if it satisfies the indoor airflow PaO_2_≧60 Torr.

13) Chest CT showed no evidence of interstitial pneumonitis or pulmonary fibrosis.

14) Written informed consent to participate in the study has been obtained from the patient.

## Exclusion criteria

1. Active overlapping cancers (simultaneous overlapping/multiple cancers and iatrogenic overlapping/multiple cancers with a disease-free interval of 5 years or less, but not including stage I prostate cancer and completely resected pathologic stage I prostate cancer with a disease-free interval of less than 5 years). However, even if the disease-free period is less than 5 years, prostate cancer of clinical stage I and a history of cancer of the following pathological stages that has been completely resected are not included in active overlapping/multiple cancers).Gastric cancer "adenocarcinoma (general type)": stage 0-I; colon cancer (adenocarcinoma): stage 0-I; rectum cancer (adenocarcinoma): stage 0-I; esophagus cancer (squamous cell carcinoma, adenosquamous cell carcinoma, basaloid cell carcinoma): stage 0; breast cancer (noninvasive ductal carcinoma of breast, noninvasive lobular carcinoma): stage 0; breast cancer (invasive ductal carcinoma of breast, invasive lobular carcinoma, Paget's disease): stage 0-IIA, Uterine cancer (endometrial adenocarcinoma, mucinous adenocarcinoma): stage I, prostate cancer (adenocarcinoma): stage I-II, cervical cancer (squamous cell carcinoma): stage 0, thyroid cancer (papillary cancer, follicular cancer): stage I, II, III, renal cancer (clear cell carcinoma, chromophobe cancer): stage I
2. Patients have an infectious disease requiring systemic treatment.
3. Patient has a fever of 38°C or higher at the time of enrollment.
4. Women who are pregnant, possibly pregnant, within 28 days postpartum, or lactating.
5. Has psychosis or psychiatric symptoms that would make participation in the study difficult.
6. Receiving continuous systemic administration (oral or intravenous) of steroids or other immunosuppressive agents.
7. Patients have concomitant diabetes mellitus that is poorly controlled despite appropriate therapy (patients are eligible if they are considered well controlled despite continuous insulin use).
8. Patients with poorly controlled hypertension.
9. Complicated unstable angina (angina with onset or worsening attacks within the last 3 weeks) or history of myocardial infarction within 6 months.
10. Serum HBs antigen positive.

# Registration and randomization

## Procedure of registration

### JCOG participating institutions

Ensuring that the patient meets all eligibility criteria and does not meet any of the exclusion criteria, and enrolling the subject from JCOG Web Entry System. Web enrollment requires a JCOG Web System personal account/password. If unknown, contact JCOG Data Center.

Patient enrollment JCOG Web Entry System

URL:https://secure.jcog.jp/dc/

(Web enrollment can be registered for 24 hours)

Patient enrollment and JCOG Web Entry System inquiries

JCOG data center

TEL:03-3542-3373

Weekdays 9-17 o'clock (do not accept holidays, Saturdays, Sundays, and the beginning of the year)

E-mail: JCOGdata@ml.jcog.jp

Contact for patient selection criteria

Study coordinators

Shintaro Kanda

Shinshu Cancer Center, Kanda-shinsu University Hospital, Faculty of Medicine

TEL:0263-37-2554 (inner line 91426)

FAX:0263-37-3302

E-mail: skanda@shinshu-u.ac.jp

Ninho Seiji

Dokkyo Medical University in Internal Medicine (Respiratory and Allergic Diseases).

TEL:0282-86-1111

FAX:0282-86-7780

E-mail: siniho@dokkyomed.ac.jp

### WJOG participating institutions

<Additional items in ver. 2.0>

No new cases are registered from WJCOG participating centre since ver. 2.0.

Electric Data Capturing (EDC) for enrollment

Enrollment is acceptable for 24 hours except during maintenance. Enrollment is performed according to the following procedures.

1) Written informed consent will be obtained from the patient to confirm eligibility for the study.

2) Physicians or collaborators will access Web registry in the following URL of the study via the Internet.

3) The required information will be entered according to the instructions of the registration system, and the eligibility criteria and exclusion criteria will be confirmed and registered.

4) As a result of registration, the reference dose of each drug calculated from the case number and body surface area is obtained.

Web Registry URL: https://edmsweb23.e-trial.co.jp/wjog_edc/

In the event of a EDC trouble, contact the e-trial help desk.

TEL: 0120-972-172

Accession time: Month to gold, 9:00 to 17:00 (except for holidays and the end of the year, 12/29-1/4)

Enquiries related to enrollment other than EDC system problems

WJOG data center

TEL:06-6633-7400

FAX:06-6633-7405

E-mail: datacenter@wjog.jp

Accession time: Month to gold, 9 to 17 o'clock (except for holidays and 12/29 to 1/3 in the end of the year)

Contact for patient selection criteria

Study coordinator

Takayasu Kurata

Department of Respiratory Oncology, Kansai Medical University Hospital

TEL:072-804-0101

FAX:072-804-0131

E-mail: kuratat@hirakata.kmu.ac.jp

### Precautions for patient registration

#### Common matters to be noted for web registration

1. Registration after initiation of protocol treatment is unacceptable without exception.
2. Once registered, patients will not be retracted (retracted from the database) unless there is withdrawal of consent, including refusal to use the data for research. For duplicate registration, the information at the initial registration (registration number, allocated arm) are used in all cases.
3. When misregistration or duplicate registration is found, contact Data Center immediately.
4. Body surface area and drug dose calculations are institutional responsibilities, and the body surface area and drug dose displayed on Web Entry System at registration are only for double-checking with the physician’s calculation. Calculation and checking must always be done also at the institution. When the body surface area calculation formula adopted in the hospital information system of the institution differs from calculation formula adopted by JCOG/WJOG (Dubois formula: Body surface area (m^2^) = Body weight (kg) ^0.425^ x Height (cm) ^0.725^ 84 ÷ 10,000), there can be a difference in the dose by the hospital information system of the institution and the dose by the calculation formula adopted by JCOG. In that case, which dosage should be used is decided by the Site Investigator/Representative.
5. The JCOG data center issues serial registration numbers from 0001 to the patients registered in JCOG, and the WJOG data center issues serial registration numbers from 10001 to the patients registered in WJOG.

#### Web registration procedure for JCOG

1. Registration is performed by accessing the URL in ‘5.1. Procedure of registration’.
2. Eligibility checks are performed on the screen of Registration Form, so it is not necessary to send a Registration Form to Data Center by mail or fax.
3. If input data are insufficient, registration is not accepted until all are met.
4. The registration number is issued after the confirmation of eligibility on the registration screen, then the registration is completed.

#### Web registration procedure for WJOG

No patients are newly registered from institutions participating in WJCOG in and after ver. 2.0.

1. A web registration system using electric data capturing (EDC) is used in this study.
2. Each participating institution must appoint a person responsible for the management of EDC.
3. WJOG issues ID of the institution manager and its password for logging in to EDC to the person responsible for the management of EDC of the institution.
4. The person responsible for the management of EDC issues researcher IDs and passwords to physicians and cooperators who use EDC at his/her institution.
5. Issued researcher IDs and passwords are managed by the person responsible for the management of EDC at each institution.

## Random allocation and adjustment factors

At the time of registration, allocation to the treatment arm is randomly made by the data center.

Random allocation is made by the minimization method using ➀ institution, ➁ clinical stage (stage IIIB and IV vs. postoperative recurrence), ➂ sex (male vs. female), and ➃ EGFR mutation (exon19 partial deletion vs. exon21 L858R point mutation) as adjustment factors to avoid large bias in them. Details of the randomization procedure are not disclosed to the researchers of the participating institutions. Also, if there are both exon 19 partial deletion and exon 21 L858R point mutation as EGFR gene mutations, the patient is registered as a case of “exon 19 partial deletion”.

## Procedures for accrual completion

No patient is newly registered from WJCOG institutions in and after ver. 2.0.

If completion of patient accrual is expected in this study (if less than 10 patients remain to be registered), JCOG Data Center will inform Study Coordinator and distribute emails to the Group mailing list to inform the registration status.

Study Coordinator contacted by Data Center on the date of accrual completion will inform the participating institutions of the prospect of accrual completion and precautions for the remaining registrations (after planned sample size is reached, the physicians at the participating institutions will not make the informed consent to new patients).

Data Center closes the registration on the JCOG Web Entry System of the study at the specified date after achieving planned accrual (17 o'clock on Friday of the week following the day on which the planned sample size is achieved, in principle).

# Treatment Plan and Treatment Modification Criteria

Unless patient safety is threatened, treatment and treatment modifications are done in compliance with the specifications in this chapter.

If it is considered that the protocol specification may cause medically dangerous situation of the patient, treatment modifications should be made according to the medical judgment of the sub-investigator. Such protocol deviation is considered to be "clinically relevant deviation" if considered medically appropriate (see 14.1.3. Protocol deviation/violation). Deviations that occur with intentions other than safety, such as increasing efficacy, are not considered clinically relevant deviations.

## Protocol treatment

Protocol treatment is initiated within 14 days of patient registration.

If treatment initiation occurs after 15 days from registration for any reason, the reason should be documented on the Treatment Form. If it is determined that treatment cannot be initiated, describe the details in the Off-treatment Form as protocol treatment termination.

When laboratory parameters worsen and eligibility criteria are no longer met before the start of treatment after registration, the sub-investigator is allowed to decide whether initiate or terminate protocol treatment at their own discretion.

"6.3. Treatment modification criteria" are not applicable at the start of the first course.

#### Drugs used

- Gefitinib (used only in patients registered before or in ver. 1.1)
- Cisplatin
- Pemetrexed
- Folic acid
- Vitamin B_12_
- Entecavir, tenofovir disoproxil fumarate, tenofovir alafenamide fumarate

The use of generic drugs is not restricted.

<Item added in ver. 2.0>

- Osimertinib (used only in patients registered in and after ver. 2.0)

### Arm A

#### Gefitinib (used only in patients registered before and in ver. 1.1)

Gefitinib is orally administered once daily at 250 mg (treatment is continued unless “6.2.2. Protocol treatment termination criteria” are met).

- If hypoacidity continues, there is the possibility of a decrease in blood drug concentration and attenuation of action. Since anacidity is reportedly common among Japanese elderly people, postprandial administration is preferred.
- Since drug-induced lung disease is likely to occur early after initiation of the administration, sufficient observation for the appearance of serious adverse reactions, such as drug-induced lung disease, is necessary at least for 4 weeks after the beginning of the administration by hospitalization or under a similar condition.*

* The definition in this study is “a condition in which screening for adverse events by physical examination and chest X-ray examination can be performed at least once every 2 weeks, and, if symptoms that suggest drug-induced lung disease, such as fever, cough, and dyspnea, appear in the patient, the diagnosis by physical examination or chest X-ray examination is possible within one day or two.”

| Drug | Dosage | Dosing route | Days of administration |
| --- | --- | --- | --- |
| Gefitinib | 250 mg/day | Oral | Daily from Day 1 |

#### Osimertinib (used only in patients registered in and after ver. 2.0)

Osimertinib is orally administered once daily at 80 mg (treatment is continued unless “6.2.2. Protocol treatment termination criteria” are met).

- The package insert of osimertinib warns, “Sufficient observation for the appearance of serious adverse reactions, such as interstitial lung disease, is necessary by hospitalization or under a similar condition of management, particularly, early after initiation of the treatment.” Also, according to the interim report of a survey after the use of osimertinib administered to patients with EGFR-TKI-resistant EGFR T790M mutation-positive inoperable or recurrent non-small-cell lung cancer, the median period until the appearance of interstitial lung disease from the beginning of the treatment was 54 days with a standard deviation of 41 days. In consideration of these reports, treatment is conducted in this study under a condition in which screening for adverse events by means of physical examination and chest X-ray examination can be performed at least once every 2 weeks until 10 weeks after the beginning of protocol treatment, and, if symptoms that suggest drug-induced lung disease, such as fever, cough, and dyspnea, appear in the patient, the diagnosis by means of physical examination and chest X-ray examination can be made within one day or two.

| Drug | Dosage | Dosing route | Days of administration |
| --- | --- | --- | --- |
| Osimertinib | 80 mg/day | Oral | Daily from Day 1 |

### Arm B

Gefitinib is administered to patients registered before and in Ver. 1.1, and osimertinib is administered to those registered in and after ver. 2.0.

Gefitinib is orally administered once daily at 250 mg/day from day 1 to day 56, then, after a 2-week no-dosing period, 3 courses of cisplatin+pemetrexed combination therapy each with a duration of 3 weeks is performed.

Thereafter, gefitinib is orally administered once daily at 250 mg (treatment is continued unless “6.2.2. Protocol treatment termination criteria” are met).

<Item added in ver. 2.0>

Osimertinib is orally administered once daily at 80 mg/day from day 1 to day 56, and, after a 2-week no-dosing period, 3 courses of cisplatin+pemetrexed combination therapy each with a duration of 3 weeks is performed.

Thereafter, osimertinib is orally administered once daily at 80 mg/day (treatment is continued unless “6.2.2. Protocol treatment termination criteria” are met).

#### First-line gefitinib therapy or first-line osimertinib therapy

**First-line gefitinib therapy (used only in patients registered before and in ver. 1.1)**

Gefitinib is orally administered once daily at 250 mg/day from day 1 to day 56. The treatment must be ended on day 56 even if the gefitinib administration is interrupted or missed.

- If hypoacidity continues, there is the possibility of a decrease in blood drug concentration and attenuation of its action. Since anacidity is reportedly common in Japanese elderly people, postprandial administration is preferred.
- Since pneumonitis is likely to occur early after the beginning of the administration, sufficient observation for the appearance of serious adverse reactions such as pneumonitis by hospitalization or under a similar condition* is necessary at least for 4 weeks after the beginning of the administration.

* The definition in this study is “a condition in which screening for adverse events by physical examination and chest X-ray examination can be performed at least once every 2 weeks, and, if symptoms that suggest drug-induced lung disease such as fever, cough, and dyspnea appear in the patient, the diagnosis by physical examination or chest X-ray examination is possible within one day or two.”

| Drug | Dosage | Dosing route | Days of administration (usually) |
| --- | --- | --- | --- |
| Gefitinib | 250 mg/day | Oral | Daily from day 1 to day 56 |

**First-line osimertinib therapy (used only in patients registered in and after ver. 2.0)**

Osimertinib is orally administered once daily at 80 mg/day from day 1 to day 56. The treatment must be ended on day 56 even if the osimertinib administration is interrupted or missed.

- The package insert of osimertinib warns, “Sufficient observation for the appearance of serious adverse reactions such as interstitial lung disease is necessary by hospitalization or under a similar condition of management, particularly, early after initiation of the treatment.” Also, according to the interim report of a survey after the use of osimertinib administered to patients with EGFR-TKI-resistant EGFR T790M mutation-positive inoperable or recurrent non-small-cell lung cancer, the median period until the appearance of interstitial lung disease from the beginning of the treatment was 54 days with a standard deviation of 41 days. In consideration of these reports, treatment is conducted in this study under a condition in which screening for adverse events by means of physical examination and chest X-ray examination can be performed at least once every 2 weeks until 10 weeks after the beginning of protocol treatment, and, if symptoms that suggest drug-induced lung disease, such as fever, cough, and dyspnea appear in the patient, the diagnosis by means of physical examination and chest X-ray examination can be made within one day or two.

| Drug | Dosage | Dosing route | Days of administration (usually) |
| --- | --- | --- | --- |
| Osimertinib | 80 mg/day | Oral | Daily from day 1 to day 56 |

#### Cisplatin+pemetrexed combination therapy (CDDP+PEM combination therapy)

Three courses of this therapy, each with a duration of 3 weeks, are repeated, starting on day 15 (usually on day 71) by defining the day after the last day of gefitinib or osimertinib administration* (day 1-56) as day 1.

- Gefitinib is administered to patients registered before and in ver. 1.1, and osimertinib is administered to those registered in and after ver. 2.0.

| Drug | Dose | Dosing route | Days of administration (usually) |
| --- | --- | --- | --- |
| Cisplatin | 75 mg/m^2^ | i.v. infusion | day 71, 92, 113 |
| Pemetrexed | 500 mg/m^2^ | i.v. infusion | day 71, 92, 113 |
| Folic acid | 0.5 mg | oral | Daily from 7 or more days before the scheduled beginning of pemetrexed administration  From the last day of pemetrexed administration to day 22 |
| Vitamin B_12_ | 1 mg | i.m. injection | Administered 7 or more days before the scheduled beginning of pemetrexed administration |

- The doses of cisplatin and pemetrexed are determined by rounding down the doses calculated from the body surface area below 1 milligram. The doses are calculated using the body weight measured within 14 days before the beginning of the first course of cisplatin+pemetrexed therapy.
- The doses are not corrected if the body weight change after the beginning of treatment from the body weight used for the dose calculation of the first course is within ±10% but determined again by recalculating the body surface area if body weight change of greater than ±10% is observed. Also, if further body weight change greater than ±10% compared with the body weight at the time of recalculation is observed, the doses are determined again by recalculating the body surface area.
- Pemetrexed and cisplatin are administered in this order.
- Although methods concerning cisplatin administration recommended by the package insert including fluid replacement are listed below, adjustment of the volume of fluid replacement by the judgment of each institution is permitted.

1. Fluid replacement of 1,000-2,000 mL is performed before cisplatin administration.
2. At the time of cisplatin administration, cisplatin is mixed with 500-1,000 mL of physiological saline or glucose/saline mixture depending on the dose and administered by i.v. infusion over 60-120 minutes. The dosing mixture is shielded from the light if the administration is protracted.
3. After cisplatin administration, fluid replacement of 1,000-2,000 mL is performed to ensure sufficient diuresis.
4. Attention is paid to securing the urine volume, and diuretics, such as mannitol, are used as necessary.

- Pemetrexed is diluted with 100 mL of physiological saline and administered by i.v. infusion over 10 minutes.
- The administration of NK1 receptor antagonist (aprepitant), 5-HT_3_ receptor antagonist, or corticosteroid (e.g., dexamethasone) is recommended for the prevention of nausea/vomiting.

#### Gefitinib or osimertinib monotherapy following CDDP+PEM combination therapy (gefitinib or osimertinib after CDDP+PEM)

**Gefitinib after CDDP+PEM (used only in patients registered before and in ver. 1.1)**

Gefitinib is orally administered once daily at 250 mg/day from day 22 (usually day 134) by defining the last day of CDDP+PEM combination therapy as day 1 (treatment is continued unless “6.2.2. Protocol treatment termination criteria” are met).

- If hypoacidity continues, there is the possibility of a decrease in blood drug concentration and attenuation of action. Since anacidity is reportedly common among Japanese elderly people, postprandial administration is preferred.

| Drug | Dose | Dosing route | Days of administration (usually) |
| --- | --- | --- | --- |
| Gefitinib | 250 mg/day | Oral | Daily from day 134 |

**Osimertinib after CDDP+PEM (used only in patients registered in and after ver. 2.0)**

Osimertinib is orally administered once daily at 80 mg/day from day 22 (usually day 134) by defining the last day of CDDP+PEM combination therapy as day 1 (treatment is continued unless “6.2.2. Protocol treatment termination criteria” are met).

| Drug | Dose | Dosing route | Days of administration (usually) |
| --- | --- | --- | --- |
| Osimertinib | 80 mg/day | Oral | Daily from day 134 |

## Protocol Treatment Termination/Completion Criteria

### Definition of protocol treatment completion

Since the protocol treatment is continued in both Arm A and Arm B until the protocol termination criteria are met, no definition of completion of the protocol treatment is set.

### Criteria for termination of protocol treatment

Protocol treatment is terminated in any of the following cases.

As protocol treatment, gefitinib is administered to patients registered before and in ver. 1.1, and osimertinib is administered to those registered in and after ver. 2.0.

1. Judgment of protocol treatment as ineffective

- In case of progression of the primary disease after the beginning of treatment

* In Arm B, in case of progression during the no-dosing period after the first-line gefitinib or osimertinib therapy, the treatment is advanced to cisplatin+pemetrexed combination therapy if the criteria for initiation of cisplatin+pemetrexed therapy (See 6.3.2.) are fulfilled without termination of the protocol treatment.

Note) Whether the treatment is “ineffective” or not, which determines the continuation or discontinuation of treatment, is decided by comprehensive clinical judgment. The overall effect evaluated by imaging examinations (CR, PR, SD, PD) is used strictly as a reference. In reality, the overall effect can be rated as PD even when the tumor is reduced in size or as PR even when the tumor has enlarged. There are cases in which termination of protocol treatment is appropriate based on the judgment that the treatment is ineffective when the overall effect is PR, cases in which the treatment is judged to be effective even when the overall effect is PD, or cases in which the continuation of treatment is appropriate for the prevention of flare after termination or control of proliferation until the beginning of the next treatment. However, all treatments performed after the judgment of the overall effect as PD are regarded as second-line treatments even if their contents are the same as those of the protocol treatment.

1. Cases in which protocol treatment cannot be continued due to adverse events
2. Grade 4 non-hematological toxicity* is observed (with the exception of the following adverse events)

(*Non-hematological toxicity: Adverse events other than anemia, bone marrow hypocellular, lymphocyte count decreased, neutrophil count decreased, white blood cell decreased, platelet count decreased, and CD4 lymphocyte decreased in CTCAE v4.0-JCOG)

However, the following adverse events are excluded.

Hyperglycemia, hypernatremia, hyponatremia, hyperkalemia, hypokalemia hypercalcemia, hypocalcemia

1. The administration of gefitinib or osimertinib cannot be resumed by day 22 with the day of termination of its administration defined as day 1.
2. A further reduction of the dose of gefitinib or osimertinib becomes necessary after reducing its dose to Level-1.
3. “Termination criteria for gefitinib or osimertinib” are met 3 times after reducing the dose of gefitinib or osimertinib to Level-1.
4. The first course of CDDP+PEM combination therapy cannot be initiated by day 22 with the day of planned initiation of the first course defined as day 1.
5. The second (or third) course of CDDP+PEM combination therapy cannot be initiated by day 22 with the day of planned initiation of the second (or third) course of CDDP+PEM combination therapy defined as day 1, and “6.3.1.2) Criteria for initiation of gefitinib administration” or “6.3.1.5) Criteria for initiation of osimertinib administration” are not met at day 22.
6. The administration of gefitinib or osimertinib cannot be initiated by day 22 after CDDP+PEM therapy with the day of planned initiation of gefitinib or osimertinib administration after CDDP+PEM as day 1.
7. Clear pneumonitis has appeared on chest CT examination (≥Grade 1 except radiation pneumonitis, ≥Grade 2 radiation pneumonitis*).

- If palliative radiation therapy of the chest is performed before initiation of the protocol treatment with the occurrence of associated radiation pneumonitis, treatment can be continued if radiation pneumonitis is Grade 1 but terminated if it is ≥Grade 2.

1. Provisions of termination of protocol treatment in Treatment modification criteria (6.3.) are met.
2. The sub-investigator has judged that termination of protocol treatment is necessary due to adverse events regardless of the Treatment modification criteria.
3. If the patient offers termination of protocol treatment for reasons not denied to be associated with the adverse event
   - This category should be used if an association with an adverse event cannot be ruled out.
4. When the patient offers termination of protocol treatment because of reasons judged not to be association with adverse events
   - Rejection by the patient after registration and before initiation of protocol treatment
   - When the association with adverse events can be excluded first such as change of residence of the patient or his/her family during protocol treatment.
5. Death during protocol treatment
   - Death before deciding to terminate protocol treatment for other reasons
6. In addition, progressions before the start of protocol treatment after registration (protocol treatment could not be initiated due to rapid progression), protocol violations were identified, and ineligibility was determined due to changes in pathological diagnosis after registration and treatment was changed.
7. If no progression is observed for 5 years or longer after initiation of treatment, protocol treatment can be terminated with consent by the patient.

The date of termination of protocol treatment shall be the date of death in case of 6.2.2.5) and the date on which the investigator/Subinvestigator judged termination of protocol treatment in other cases.

## Treatment modification criteria

The following terms shall be used for the treatment modification.

Termination: Discontinuation of a part of or all of the treatment without restarting.

Delay: Delay the start of the course or administration of treatment from the planned date.

Suspending: Temporary interruptions or withdrawals that may be resumed if conditions are met.

Categories of infection (CTCAEv4.0-JCOG) used in this study are as follows

**Infection:** CTCAEv4.0-JCOG infections and infestations

Bronchial infection; pulmonary infection; upper respiratory tract infection; mediastinal infection; pleural infection; catheter-related infection; biliary tract infection; gallbladder infection; bladder infection; kidney infection; urinary tract infection; peritoneal infection

### Treatment modification criteria: gefitinib or osimertinib monotherapy* (applied to both Arms A and B)

* Gefitinib is administered to patients registered before and in ver. 1.1, and osimertinib is administered to those registered in and after ver. 2.0.

#### 1) Gefitinib dose level (used only in patients registered before and in ver. 1.1)

| Drug | Dose level | Dosage |
| --- | --- | --- |
| Gefitinib | Level 0 | 250 mg/day once daily |
|  | Level -1 | 250 mg/day once every 2 days |

#### 2) Gefitinib initiation criteria (including restarting after suspension) (used only in patients registered before and in ver. 1.1)

- Gefitinib administration is started by confirming that all of the “Gefitinib initiation criteria” below on the day of administration or the day before.
- The initiation is postponed on a day-to-day basis if even one of the “Gefitinib initiation criteria” is not met.
- Arm B only: If gefitinib administration cannot be initiated by day 22 after CDDP+PEM by defining the scheduled day of the initiation of gefitinib administration after CDDP+PEM as day 1, protocol treatment is terminated. (protocol treatment is not terminated if gefitinib administration can be initiated on day 22 after CDDP+PEM).
- Arm B alone: If the dose level of the first-line gefitinib therapy is reduced to Level -1, gefitinib administration is initiated after CDDP+PEM at Level -1 (the dose is not increased again).
- “Gefitinib initiation criteria” are not applied at the time of the beginning of protocol treatment.

Table 6.3.1.a Gefitinib initiation criteria (applied to both Arms A and B)

| Item | Grade |
| --- | --- |
| 1. PS | 0-2 |
| 1. No fever (axillary temperature) | Grade 0 (<38°C) |
| 1. Neutrophil count (rod+segmented karyocyte) | ≤Grade 2 (≥1,000 /mm^3^) |
| 1. Platelet count | ≥10×10^4^/mm^3^ |
| 1. Total bilirubin | ≤2.0 mg/dL |
| 1. AST | ≤100 U/L |
| 1. ALT | ≤100 U/L |
| 1. SpO_2_ (room air) | ≥92%* |
| 1. Pruritus, acneiform rash, dry skin | ≤Grade 2* |
| 1. Paronychia | ≤Grade 2* |
| 1. Oral mucositis | ≤Grade 2* |
| 1. Diarrhea | ≤Grade 1 |

* Even if SpO_2_ is <92%, ➇ is considered to be met if PaO_2_ is ≥60 Torr.

* Gefitinib administration is not initiated if its continuation is judged to be difficult due to even Grade 2 pain, itching, or cosmetic problem.

#### 3) Suspension/dose reduction/termination of gefitinib (used only in patients registered before and in ver. 1.1)

- Oral gefitinib administration is suspended if even one of the “Gefitinib suspension criteria” is met during the administration period.
- Gefitinib administration is restarted at the same dose level if “6.3.1.2) Gefitinib initiation criteria” are all met by day 22 with the day of its suspension defined as day 1.
- Protocol treatment is terminated if even one of the “6.3.1.2) Gefitinib initiation criteria” is not met by day 22 with the day of its suspension defined as day 1.
- If “Gefitinib suspension criteria” are met twice due to the same adverse event, the dose of gefitinib is reduced by one level (regardless of the duration of the adverse events or the interval between the two events). Even if two or more criteria are met, the dose is reduced by only one level. Also, adverse events that occur during first-line gefitinib therapy are not counted as adverse events during the subsequent gefitinib therapy after CDDP+PEM.

Example 1: If gefitinib administration is suspended due to the occurrence of Grade 3 diarrhea on day 45 and restarted on day 48, and Grade 3 diarrhea occurs again on day 150, the dose of gefitinib is reduced to Level -1, because they are the same adverse event and meet “Gefitinib suspension criteria” twice.

Example 2: Gefitinib administration is suspended because of the occurrence of Grade 3 pruritis on day 20 and restarted on day 28, but Grade 3 diarrhea occurs on day 29, the dose of gefitinib is not reduced.

Example 3: Gefitinib administration is suspended due to Grade 3 acneiform rash on day 15 during first-line gefitinib therapy and restarted on day 22. If, thereafter, the treatment is advanced to gefitinib administration after CDDP+PEM, and Grade 3 acneiform rash occurs on day 149, the dose of gefitinib is not reduced (because adverse events that occur during first-line gefitinib treatment in Arm B are not counted as adverse events during gefitinib therapy after CDDP+PEM).

- If further reduction of the dose of gefitinib becomes necessary after reducing it to Level -1, protocol treatment is terminated. Protocol treatment is also terminated if “Gefitinib suspension criteria” are met 3 times after dose reduction to Level -1.

Example 1: Gefitinib administration is suspended due to Grade 3 AST elevation on day 48 and restarted on day 51, but the dose is reduced to Level -1 due to the occurrence of Grade 3 AST elevation on day 55. Thereafter, the administration is restarted on day 58. Then, on day 145, gefitinib administration is suspended due to the occurrence of Grade 3 diarrhea, restarted on day 148, but Grade 3 diarrhea occurs again on day 150. In such a case, protocol treatment is terminated since further dose reduction is necessary after the dose is reduced to Level -1.

Example 2: Gefitinib administration is suspended on day 21 due to the occurrence of Grade 3 ALT elevation and restarted on day 37, but the dose of gefitinib is reduced to Level -1 due to the occurrence of Grade 3 ALT elevation on day 41. Thereafter, the administration is restarted on day 58, suspended on day 149 due to Grade 3 diarrhea, restarted but suspended on day 160 due to Grade 3 pruritus, and restarted but suspended on day 180 due to Grade 3 AST elevation. In such a case, protocol treatment is terminated, because “Gefitinib suspension criteria” are met 3 times after dose reduction to Level -1.

- Arm B alone: If gefitinib administration cannot be initiated by day 22 with the scheduled day of the initiation of gefitinib administration after CDDP+PEM as day 1, protocol treatment is terminated (protocol treatment is not terminated if gefitinib can be administered on day 22).
- Protocol treatment is terminated if clear pneumonitis appears on chest CT (≥Grade 1 for conditions other than radiation pneumonitis, ≥Grade 2 for radiation pneumonitis*).
- If radiation pneumonitis occurs due to palliative radiation therapy of the chest performed before protocol treatment, treatment may be continued if it is Grade 1 but is terminated if it is ≥Grade 2.

Table 6.3.1.b Gefitinib suspension criteria (applied to both Arms A and B)

| Item | Grade |
| --- | --- |
| 1. Pruritus, acneiform rash, dry skin | Grade 3 or intolerable* Grade 2 |
| 1. Paronychia | Grade 3 or intolerable* Grade 2 |
| 1. Oral mucositis | Grade 3 or intolerable* Grade 2 |
| 1. Diarrhea | Grade 3 or intolerable* Grade 2 |
| 1. Elevated AST | >150 U/L |
| 1. Elevated ALT | >150 U/L |

* “Intolerable” means cases in which pain, itching, or cosmetic problem makes continuation of gefitinib administration difficult.

#### 4) Osimertinib dose level (used only in patients registered in and after ver. 2.0)

| Drug | Dosage level | Dosage |
| --- | --- | --- |
| Osimertinib | Level 0 | 80 mg/day once daily |
|  | Level -1 | 40 mg/day once daily |

#### 5) Osimertinib initiation criteria (including restarting after suspension) (used only in patients registered in and after ver. 2.0)

- Osimertinib administration is initiated after conforming the fulfillment of all of the following “osimertinib initiation criteria” on the day of administration or the day before.
- If any of the “osimertinib initiation criteria” is not met, the initiation is postponed on a day-to-day basis.
- Arm B alone: If the osimertinib administration after CDDP+PEM cannot be initiated by day 22 with the scheduled day of the initiation of osimertinib administration after CDDP+PEM defined as day 1, protocol treatment is terminated (protocol treatment is not terminated if osimertinib administration after CDDP+PEM can be initiated on day 22).
- Arm B alone: If the dose of first-line osimertinib administration is reduced to Level -1, osimertinib administration after CDDP+PEM is initiated at Level -1 (not increased again).
- “Osimertinib initiation criteria” are not applied at the time of initiation of protocol treatment.

Table 6.3.1.c Osimertinib initiation criteria (applied to both Arms A and B)

| Item | Grade |
| --- | --- |
| 1. PS | 0-2 |
| 1. No fever (axillary temperature) | Grade 0 (<38°C) |
| 1. Neutrophil count (rod+segmented karyocyte) | ≤Grade 2 (≥1,000 /mm^3)^ |
| 1. Platelet count | ≥10×10^4^ /mm^3^ |
| 1. Total bilirubin | ≤2.0 mg/dL |
| 1. AST | ≤100 U/L |
| 1. ALT | ≤100 U/L |
| 1. SpO_2_ (room air) | ≥92%*^1^ |
| 1. Pruritus, acneiform rash, dry skin | ≤Grade 2*^2^ |
| 1. Paronychia | ≤Grade 2*^2^ |
| 1. Oral mucositis | ≤Grade 2*^2^ |
| 1. Diarrhea | ≤Grade 1 |
| 1. Prolonged corrected QT interval*^3^ | ≤Grade 1 |
| 1. Thromboembolism*^3^ | ≤Grade 2 |

*^1^ Even if SpO_2_ is <92%, ➇ is considered to be met by fulfillment of PaO_2_ ≥60 Torr.

*^2^ Osimertinib administration is not initiated if the continuation of osimertinib administration is judged to be difficult due to even Grade 2 pain, itching, or cosmetic problem.

*^3^ Evaluation item added with change to osimertinib

#### 6) Suspension/dose reduction/termination of osimertinib (used only in patients registered in and after ver. 2.0)

- Oral osimertinib administration is suspended if any of the following “Osimertinib suspension criteria” is met during the period of osimertinib administration.
- Osimertinib administration is restarted at the same dose level if all of “6.3.1.5) Osimertinib initiation criteria” are met by day 22 with the day of suspension of osimertinib administration defined as day 1.
- Protocol treatment is terminated if any of “6.3.1.5) Osimertinib initiation criteria” is not met by day 22 with the day of suspension of osimertinib administration defined as day 1.
- If “Osimertinib suspension criteria” are met twice due to the same adverse event, the dose of osimertinib is reduced by 1 level (regardless of the duration of the adverse events or the interval between the two events). However, even if 2 or more criteria are met, the dose is reduced by only 1 level. Also, adverse events that occur during the first-line osimertinib therapy are not counted as adverse events during the subsequent osimertinib therapy after CDDP+PEM.

Example 1: If osimertinib administration is suspended due to the occurrence of Grade 3 diarrhea on day 45, restarted on day 48, and Grade 3 diarrhea occurs again on day 150, the dose of osimertinib is reduced to Level -1, because “Osimertinib suspension criteria” are met twice due to the same adverse event.

Example 2: If osimertinib administration is suspended on day 20 due to the occurrence of Grade 3 pruritus, restarted on day 28, but Grade 3 diarrhea occurs on day 29, the dose of osimertinib is not reduced.

Example 3: If osimertinib administration is suspended on day 15 during first-line osimertinib therapy due to Grade 3 acneiform rash, restarted on day 22, the treatment is subsequently advanced to osimertinib therapy after CDDP+PEM, and Grade 3 acneiform rash occurs on day 149, the dose of osimertinib is not reduced (because adverse events that occur in Arm B during first-line osimertinib therapy are not counted as adverse events during osimertinib therapy after CDDP+PEM).

- Protocol treatment is terminated if, after the dose of osimertinib is reduced to Level -1, further reduction of osimertinib becomes necessary. Protocol treatment is also terminated if “Osimertinib suspension criteria” are met 3 times after reducing the dose of osimertinib to Level -1.

Example 1: Osimertinib administration is suspended on day 48 due to the occurrence of Grade 3 AST elevation, restarted on day 51, but the dose of osimertinib is reduced to Level -1 on day 55 due to the occurrence of Grade 3 AST elevation. The administration is restarted on day 58 but suspended on day 145 due to the occurrence of Grade 3 diarrhea, restarted on day 148, but Grade 3 diarrhea occurred on day 150. In such a case, protocol treatment is terminated, because further dose reduction is necessary after reducing the dose to Level -1.

Example 2: Osimertinib administration is suspended on day 21 due to the occurrence of Grade 3 ALT elevation, restarted on day 37, but the dose of osimertinib is reduced to Level -1 on day 41 due to the occurrence of Grade 3 ALT elevation. Thereafter, the administration is restarted on day 58, suspended on day 149 due to the occurrence of Grade 3 diarrhea, restarted but suspended on day 160 due to Grade 3 pruritus, restarted again, but Grade 3 AST elevation occurs again on day 180. In such a case, protocol treatment is terminated, because “osimertinib suspension criteria” are met 3 times after the dose is reduced to Level -1.

- Arm B alone: If osimertinib administration cannot be initiated by day 22 with the scheduled day of the initiation of osimertinib administration after CDDP+PEM defined as day 1, protocol treatment is terminated (protocol treatment is not terminated if the administration can be administered on day 22).
- Protocol treatment is terminated if clear pneumonitis appears on chest CT (≥Grade 1 for conditions other than radiation pneumonitis, ≥Grade 2 for radiation pneumonitis*).
- If radiation pneumonitis occurs due to palliative radiation therapy of the chest performed before protocol treatment, treatment may be continued if the condition is Grade 1 but is terminated if it is ≥Grade 2.

Table 6.3.1.d Osimertinib suspension criteria (applied to both Arms A and B)

| Item | Grade |
| --- | --- |
| 1. Pruritus, acneiform rash, dry skin | Grade 3 or intolerable*^1^ Grade 2 |
| 1. Paronychia | Grade 3 or intolerable*^1^ Grade 2 |
| 1. Oral mucositis | Grade 3 or intolerable*^1^ Grade 2 |
| 1. Diarrhea | Grade 3 or intolerable*^1^ Grade 2 |
| 1. Elevated AST | >150 U/L |
| 1. Elevated ALT | >150 U/L |
| 1. Neutrophil count (rod+segmented karyocyte)*^2^ | ≥Grade 3 (<1,000 /mm^3^) |
| 1. Platelet count*^2^ | ≥Grade 3 (<5.0×10^4^ /mm^3^) |
| 1. Prolonged corrected QT interval*^2^ | ≥Grade 3 |
| 1. Thromboembolism*^2^ | ≥Grade 3 |

*^1^ “Intolerable” means cases in which the continuation of osimertinib administration is judged to be difficult for reasons such as pain, itching, and cosmetic problem.

*^2^ Evaluation item added with change to osimertinib

### Treatment modification criteria: Cisplatin+pemetrexed (CDDP+PEM) combination therapy (Arm B alone)

#### Cisplatin+pemetrexed dose level

| Drug | Dose level | Dosage and Administration |
| --- | --- | --- |
| Cisplatin | Level 0 (full dose) | 75 mg/m^2^ day 1, intravenous drip infusion |
|  | Level -1 | 60 mg/m^2^ day 1, intravenous drip infusion |
|  | Level -2 | 50 mg/m^2^ day 1, intravenous drip infusion |
| Pemetrexed | Level 0 (full dose) | 500 mg/m^2^ day 1, intravenous drip infusion |
|  | Level -1 | 400 mg/m^2^ day 1, intravenous drip infusion |
|  | Level -2 | 350 mg/m^2^ day 1, intravenous drip infusion |

#### 2) Cisplatin+pemetrexed initiation criteria

- The first course is initiated by confirming that all the following “Cisplatin/pemetrexed initiation criteria” are met on the day of the initiation of the course or the day before. The second and subsequent courses are initiated by confirming the fulfillment of all the following “Cisplatin/pemetrexed initiation criteria” except ➈ on the day of the initiation of the course or the day before.
- The initiation is postponed on a day-to-day basis if even one of the “Cisplatin+pemetrexed initiation criteria” is not met.
- The doses are calculated using **the body weight** measured **within 14 days before the initiation of the first course of cisplatin+pemetrexed**. For the second and subsequent courses, the body weight is measured within 14 days before the initiation of the course, and if change of more than ±10% compared with the body weight at the initiation of the first course is observed, the body surface area is calculated again, and the dose is calculated again. If change of more than ±10% compared with the body weight at the time of re-calculation is observed thereafter, the body surface area is calculated again, and the dose is redetermined.
- If the first course of cisplatin+pemetrexed cannot be initiated by day 22 with the scheduled day of its initiation defined as day 1, protocol treatment is terminated (protocol treatment is not terminated if cisplatin+pemetrexed can be administered on day 22).
- If the second (or third) course of cisplatin+pemetrexed cannot be initiated by day 22 with the scheduled day of its initiation defined as day 1, and if “6.3.1. 2) Gefitinib initiation criteria” are met on day 22, gefitinib administration is restarted on day 22 (protocol treatment is not considered to be terminated).
- If the second (or third) course of cisplatin+pemetrexed cannot be initiated by day 22 with the scheduled day of its initiation defined as day 1, and if “6.3.1. 2) Gefitinib initiation criteria” are not met on day 22, protocol treatment is terminated (protocol treatment is not terminated if gefitinib can be administered on day 22).

Table 6.3.2.a Cisplatin+pemetrexed initiation criteria (Arm B)

| Item | Grade |
| --- | --- |
| 1. PS | 0-1 |
| 1. No fever (axillary temperature) | Grade 0 (<38°C) |
| 1. Neutrophil count (rod + segmented karyocyte) | ≤Grade 1 (≥1,500 /mm^3)^ |
| 1. Platelet count | ≥10×10^4^ /mm^3^ |
| 1. Total bilirubin | ≤2.0 mg/dL |
| 1. AST | ≤100 U/L |
| 1. ALT | ≤100 U/L |
| 1. Serum creatinine | ≤1.5 mg/dL |
| 1. Creatinine clearance | ≥60 mL/min*^1^ |
| 1. SpO_2_ (room air) | ≥92%*^2^ |

*^1^ If the estimated value is <60 mL/min, and if the measured value in 24-hour pooled urine is confirmed to be ≥60 mL/min, ➈Creatinine clearance is considered to be fulfilled.

Cockcroft-Gault formula

Male: Ccr = {(140 – age) × body weight (kg)} / {72 × serum creatinine (mg/dL)}

Female: Ccr = 0.85 × {(140 – age) × body weight (kg)} / {72 × serum creatinine (mg/dL)}

*^2^ If SpO_2_ is <92%, ➉ is considered to be fulfilled if PaO_2_ is ≥60 Torr.

#### 3) Cisplatin+pemetrexed dose reduction/termination criteria

- If any of the toxicities listed in the following “Cisplatin+pemetrexed dose reduction/termination criteria” in the previous course of cisplatin+pemetrexed combination therapy, the doses of cisplatin and pemetrexed in the next course are reduced by 1 level according to the table. However, the dose of each drug is reduced by one level even if the toxicities correspond to two or more items.
- The dose is not increased again even if the following toxicities disappear after dose reduction.

Table 6.3.2.b Cisplatin+pemetrexed dose reduction/termination criteria (Arm B alone)

| Item | Grade | Cisplatin | Pemetrexed |
| --- | --- | --- | --- |
| 1. Creatinine increased | 1.5< creatinine ≤2.0 mg/dL | Reduce the level by 1 | No change |
|  | >2.0 mg/dL | **Terminate CDDP+PEM** | |
| 1. Neutrophil count decreased | Grade 4 (<500/mm^3)^ | Reduce the level by 1 | Reduce the level by 1 |
| 1. Febrile neutropenia*^1^ | ≥Grade 3 | Reduce the level by 1 | Reduce the level by 1 |
| 1. Platelet count decreased | Grade 4 (<2.5×10^4^/mm^3)^ | Reduce the level by 1 | Reduce the level by 1 |
| 1. Non-hematological toxicities other than the above*^2^ | Grade 3 | Reduce the level by 1 | Reduce the level by 1 |

*^1^ Fever of ≥38°C observed even once or sustained for more than 1 hour with a neutrophil count of <1,000/mm^3^

*^2^ Adverse events whose relationships with anorexia, nausea/vomiting, hypernatremia, constipation, hyperglycemia, or cisplatin+pemetrexed is “unlikely” or “not related” are excluded.

### Consultation on treatment modification

If there are any questions about treatment modification, contact "16.6. Study Coordinator".

Study Coordinator Contact:

**JCOG**

**Shintaro Kanda**

First Department of Internal Medicine

Shinshu University Hospital

3-1-1 Asahi, Matsumoto Nagano 390-8621

Tel: +81-263-37-2554 (ex. 91426)

Fax: +81-263-37-3302

E-mail: skanda@shinshu-u.ac.jp

**Seiji Niho**

Department of Pulmonary Medicine and Clinical Immunology

Dokkyo University Hospital

880 Kitakobayashi, Mibu, Shimotsugagun, Tochigi 321-0293

Tel: +81-282-86-1111

Fax: +81-282-86-7780

E-mail: siniho@dokkyomed.ac.jp

**WJOG**

**Takayasu Kurata**

Department of Respiratory Oncology

Kansai Medical University

2-3-1 Shinmachi Hirakata, 573-1191

Tel: +81-72-804-0101

Fax: +81-72-804-0131

E-mail: kuratat@hirakata.kmu.ac.jp

## Concomitant treatment and supportive care

### Required concomitant treatment and supportive care

#### Laboratory Test and Supportive Care for HBsAg-Negative and HBc Antibody-Positive and/or HBs Antibody-Positive Cases

HBV-DNA quantitative analysis should be performed at least once prior to initiation of chemotherapy. HBV-DNA assays are performed by real-time PCRs.

#### i) HBV-DNA ≥20 IU/mL (1.3 log IU/mL) prior to initiation of chemotherapy

It has been clarified that HBV-DNA replicates persist at low levels in the livers and peripheral blood mononuclear cells when HBc or HBs are positive, even if they are HBs-Ag negative. It has been reported that reactivation of HBV and development of severe hepatitis are caused by the use of potent immunosuppressive agents even in such patients with previous infections.

If HBV-DNA ≥20 IU/mL (1.3 log IU/mL), the risk of HBV reactivation is judged to be as high as in HBs-Ag positive cases, and prophylactic administration of nucleic acid analogues (entecavir, tenofovir disoproxil fumarate, tenofovir alafenamide fumarate) is administered. The following laboratory tests and supportive care are performed in accordance with the "Guideline for the Treatment of Hepatitis B, 2nd edition (Japanese Society of Hepatology)" with reference to the following for examination, dosage and dosing regimen of supportive therapy, and monitoring before the start of chemotherapy.

However, these are not applicable if the HBs antibody alone is positive and the HBV vaccination history is obvious.

➀ Dosing schedule for supportive care (nucleic acid analogues prophylaxis)

- **Drugs used**
- **Entecavir (Bristol-Myers: Baraclude Tablets 0.5 mg)**
- **Tenofovir disoproxil fumarate (GlaxoSmithKline: Tenozet Tablets 300 mg)**
- **Tenofovir alafenamide fumarate (Giliado: Vemuridi Tablets 25 mg)**

The following dosage regimen should be followed, starting at least 1 week before the start of chemotherapy (as soon as possible), and continuing for at least 12 months after the end of chemotherapy. After 12 months of completion of chemotherapy, nucleic acid analogues may be discontinued if the patient meets the conditions* for discontinuing nucleic acid analogues. However, if the administration of a nucleic acid analogue is discontinued, consultation with a hepatologist is always obtained, and the administration is discontinued only if the hepatologist deems it appropriate. Bearing in mind that reactivation may occur after discontinuation of nucleic acid analogues treatment, HBV-DNA quantitative analysis should be continued at intervals specified in “➁ Monitoring” In addition, if HBV-DNA level is 20 IU/mL (1.3 log IU/mL) or more after discontinuation of nucleic acid analogues therapy, nucleic acid analogues therapy should be resumed immediately.

| *1 Requirements for discontinuation of nucleic acid analogues (entecavir, tenofovir disoproxil fumarate, tenofovir alafenamide fumarate): all of the following   1. The treatment has been continued for at least 12 months after the completion of immunosuppression or chemotherapy. 2. HBV-DNA quantitative analysis is persistently negative 3. HBsAg and HB core-related antigens are persistently negative 4. Normalized ALT (except for causes of ALT abnormalities other than HBV)   Adapted from Guidelines for the Treatment of Hepatitis B, the 3rd edition  (Japanese Society of Hepatology) |
| --- |

##### **Entecavir**

- **Dosage regimen:** Take this medicine on an empty stomach (2 hours after meals and more than 2 hours before the next meal).
- **Dosage:**

| Creatinine clearance (mL/min). | Dosage |
| --- | --- |
| 50 or more | 0.5 mg once daily |
| ≥30, <50 | 0.5 mg once every 2 days |
| ≥10, <30 | 0.5 mg once every 3 days |
| <10 | 0.5 mg once every 7 days |

- **Adverse drug reactions (incidence of all grades): nucleoside analog-naïve patients**

Diarrhea (6.0%), nausea (4.5%), constipation (3.7%), upper abdominal pain (3.0%), malaise (1.5%), nasopharyngitis (3.0%), muscle stiffness (2.2%), headache (14.2%), dizziness (3.0%), rash (incidence unknown), alopecia (incidence unknown), laboratory tests: elevated AST (GOT) (3.7%), elevated ALT (GPT) (3.7%), increased blood bilirubin (6.0%), blood amylase increased (10.4%), lipase increased (10.4%), blood glucose increased (6.0%), blood lactate increased (23.1%), BUN increased (6.7%), urine occult blood positive (4.5%), white blood cells urine positive (3.0%), white blood cell count decreased (8.2%), eosinophil count increased (0.7%),

**[significant adverse reactions (incidence unknown)]** Hepatitis worsened after completion of treatment, anaphylactoid symptoms, lactic acidosis, severe hepatomegaly due to fatty liver

Tenofovir disoproxil fumarate

- **Dosage and administration:** 300 mg is orally administered once daily.
- **Dosage**:

| Creatinine clearance (mL/min) | Dosage |
| --- | --- |
| ≥50 | 300 mg once daily |
| ≥30, <50 | 300 mg once every 2 days |
| ≥10, <30 | 300 mg once every 3 to 4 days |
| Hemodialysis | 300 mg once every 7 days^note)^  Or 300 mg after completion of cumulative approximately 12 hours of dialysis  NOTE) After hemodialysis was performed. The pharmacokinetics in patients with creatinine clearance <10 mL/min and not on hemodialysis have not been investigated. |

- **Dosing Precautions:**

In the long-term administration of tenofovir disoproxil fumarate, attention should be paid to renal dysfunction, hypophosphatemia (including Fanconi syndrome), and decrease in bone mineral density. It is recommended that renal function and serum phosphorus should be measured regularly during tenofovir disoproxil fumarate administration.

- **Adverse reactions (incidence of all grades):**

Seven patients (4.9%) had abnormal liver function tests (AST, ALT and γ-GTP increased), 4 patients (2.8%) had increased creatinine, 3 patients (2.1%) each had increased amylase, increased lipase and nausea, 2 patients (1.4%) each had abdominal pain.

**[Significant adverse reactions (incidence unknown)]** renal dysfunction, renal failure, acute renal failure, proximal renal tubular dysfunction, severe renal dysfunction such as Fanconi syndrome, acute renal tubular necrosis, renal diabetes insipidus or nephritis, severe hepatomegaly due to lactic acidosis and fatty deposition (steatohepatitis), pancreatitis

**Tenofovir alafenamide fumarate**

- **Dosage and administration:** 25 mg is orally administered once daily.
- **Dosage:**

| Creatinine clearance (mL/min). | Dosage |
| --- | --- |
| ≥15 | 25 mg once daily |
| <15 | Consider discontinuation |

- **Dosing Precautions:**

In the long-term administration of tenofovir alafenamide fumarate, attention should be paid to renal dysfunction, hypophosphatemia (including Fanconi syndrome), and decrease in bone density. It is recommended that renal function and serum phosphorus should be measured periodically during tenofovir alafenamide fumarate administration.

- **Adverse reactions (incidence of all grades):**

Nausea and abdominal distension, headache, fatigue (≥1%), dyspepsia and diarrhea, flatus, upper abdominal pain, constipation, ALT increased, arthralgia, dizziness, insomnia, pruritus, rash (≥0.5% to <1%).

**[Significant adverse reactions (incidence unknown)]** renal dysfunction, renal failure, acute renal failure, proximal renal tubular dysfunction, severe renal impairment such as Fanconi syndrome, acute renal tubular necrosis, renal diabetes insipidus or nephritis, severe hepatomegaly due to lactic acidosis and fatty deposits (fatty liver)

➁ Monitoring: Quantitative analysis of HBV-DNA (during and after administration of nucleic acid analogues)

**During nucleic acid analogue (entecavir, tenofovir) administration:**

They are monitored every 4 weeks by both HBV-DNA quantitative analysis and liver function (ASTs, ALTs). However, if HBV-DNA level is less than 20 IU/mL (1.3 log IU/mL) during administration of nucleic acid analogues, it is acceptable to perform tests every 4 to 12 weeks.

**After discontinuation of nucleic acid analogue administration:**

Bearing in mind that reactivation may occur even after discontinuation of administration of a nucleic acid analogues, the patient should be consulted with a hepatologist, and the patient should be monitored for HBV-DNA determination and hepatic function (AST/ALT) every 4 weeks for at least 1 year after discontinuation of administration of a nucleic acid analogues. If HBV-DNA level is 20 IU/mL (1.3 log IU/mL) or more after discontinuation of nucleic acid analogues therapy, the nucleic acid analogues therapy should be resumed immediately.

#### ii) HBV-DNA <20 IU/mL (1.3 log IU/mL) prior to initiation of chemotherapy

HBV-DNA quantitative analysis and hepatic function (AST, ALT) will be monitored, and nucleic acid analogues (entecavir, tenofovir disoproxil fumarate, tenofovir alafenamide fumarate) will be started when ≥20 IU/mL (1.3 log IU/mL) is achieved.

The Guidelines for the Treatment of Hepatitis B, the 3rd edition (Japanese Society of Hepatology) recommends monitoring with HBV-DNA quantitative analysis or high-sensitivity HBsAg during and after chemotherapy, depending on the risks of revitalization.

➀ **Monitoring: HBV-DNA quantitative analysis (Consider monitoring intervals based on risk)**

HBV-DNA quantitative analysis should be performed every 4-12 weeks from the start of chemotherapy until at least 12 months after the end of chemotherapy.

If HBV-DNA level is more than 20 IU/mL (1.3 log IU/mL), administration of nucleic acid analogues should be started immediately in accordance with the Guidelines for the Treatment of Hepatitis B, the 3rd edition (Japanese Society of Hepatology). If high-sensitivity HBsAg monitoring is positive for <1 IU/mL (low positive), nucleic acid analogues should be administered after additional HBV DNA determinations of ≥20 IU/mL (1.3 log IU/mL). It is advisable to consult a hepatologist at a time prior to initiation of NAs.

➁ **Supportive care in reactivation**

Nucleic acid analogues should be administered according to the supportive care described in 6.4.1. i) HBV-DNA of 20 IU/mL (1.3 log IU/mL) or more prior to initiation of chemotherapy. Once administration of nucleic acid analogues is started, nucleic acid analogues should be discontinued only if appropriate by the hepatologist.

### Recommended/not recommended concomitant treatment/supportive care

The following concomitant treatment and supportive care are recommended. Even if it is not carried out, it is not regarded as protocol deviation.

#### 1) Addressing fever during neutropenia

➀ Assessment at onset of febrile neutropenia (FN)

1. If the neutrophil count is less than 500/mm^3^, or less than 1,000/mm^3^ and is predicted to decrease to less than 500/mm^3^ in less than 48 hours, and if the axillary temperature is 37.5°C (oral 38°C) or higher, then immediately assess the severity risks and start the antivirus treatment as appropriate.
2. Severity risk assessment is performed with reference to Multinational Association for Supportive Care in Cancer (MASCC) scoring system*^1^.
3. For initial evaluation, complete blood cell count including differential and platelet count, renal function (BUN, creatinine), electrolytes, liver function (transaminases, total bilirubin, and alkaline phosphatase) tests, two or more sets of venous blood cultures before initiation of antimicrobials, one set of cultures from the catheter lumen and one set from a peripheral vein if a central venous catheter is in place, culture of suspected infected areas, and plain chest x-ray if respiratory symptoms or signs are present.
4. When febrile neutropenia (FN) develops in a patient with a central venous catheter, blood cultures from the catheter and peripheral blood are performed, and catheter-related infections are considered if there is a time difference of more than 120 minutes in the positivity of both. If appropriate antimicrobial therapy does not improve after more than 72 hours, catheter should be removed. For infections caused by Staphylococcus aureus, Pseudomonas aeruginosa, Bacillus, fungi, and acid-fast bacilli, the catheter should be removed and appropriate antimicrobial therapy based on culture results should be performed.

➁ Antibiotic use

1. In high-risk patients, β-lactams with anti-Pseudomonas aeruginosa activity are administered intravenously as a single agent. However, other antimicrobials (aminoglycosides, fluoroquinolones, and/or vancomycin) may be added to a single agent in the initial regimen in patients with unstable or complicated conditions or when drug-resistant organisms are strongly suspected. Low-risk patients may be treated with antibiotics orally or intravenously, hospitalized, or with adequate evaluation, if appropriate, as outpatients.
2. The antimicrobial agent should be reassessed 3-4 days after initiation, and antimicrobial agents should be continued or changed. As a rule, antimicrobials should be continued until the neutrophil count is at least 500/mm^3^.
3. Empiric antifungal therapy is recommended in high-risk patients who do not respond to 4-7 days of broad-spectrum antibiotics.
4. Fluoroquinolone prophylaxis is recommended in high-risk patients with an expected neutrophil count ≤ 100 /mm^3^ lasting >7 days.

➂ Therapeutic administration of G-CSF

Therapeutic administration of G-CSF during the development of FNs is referred to Section 6.4.6.3) Therapeutic Administration of G-CSF

*1 Multinational Association for Supportive Care in Cancer (MASCC) scoring system.

(Adapted in part from the Practice Guideline for Febrile Neutropenia (FN) [Japanese Society of Medical Oncology].*2)

| Item | Score |
| --- | --- |
| Clinical manifestations (select one of the followings)  * No symptoms  * Mild symptoms  * Moderate symptoms | 5  5  3 |
| No decrease in blood pressure | 5 |
| No chronic obstructive pulmonary disease | 4 |
| Solid tumors, or hematopoietic tumors without a history of fungal infection | 4 |
| No dehydration symptoms | 3 |
| Patients with fever during outpatient management | 3 |
| Age <60 | 2 |

The total score is up to 26 points. Twenty-one points or more are considered low risk and 20 points or less are considered high risk.

*2 Since patients aged 20 years or older are subjects in this study, we deleted "Not applicable to patients younger than 16 years old" from the original edition of the Practice Guideline for Febrile Neutropenia (FN) (Japanese Society of Medical Oncology).

#### 2) Inoculation of influenza vaccine and pneumococcal vaccine

There is no clear evidence about the usefulness of preventive vaccination or timing of inoculation before the beginning of, or during, chemotherapy or before surgery. However, since infection is known to be severe in immunocompromised patients, the recommendation grade of vaccination against influenza is A, and that of vaccination against pneumococcal vaccination is B by the “Practical Guideline of Febrile Neutropenia (FN)” of the Japanese Society of Medical Oncology (JSMO Guidelines), following guidelines including the IDSA Guidelines. Therefore, if it is judged that vaccination is expected to be beneficial and that its safety can be ensured, vaccination should be evaluated by considering the timing of protocol treatment and vaccination and referencing the JSMO Guidelines.

#### 3) Precautions on the day of cisplatin administration

On the day of cisplatin administration, aminoglycoside antibiotics, vancomycin, or non-steroidal anti-inflammatory drugs must not be administered or must be administered carefully if they are administered.

#### 4) Prevention of rash caused by pemetrexed

To prevent rash due to pemetrexed, corticosteroid (such as dexamethasone) should be orally administered on the day before, on the day, and on the day after the administration of pemetrexed.

#### 5) Skin disorder

1. Xeroderma (dry skin): A moisturizing agent (e.g., heparin analog, petrolatum, urea preparation) and, if necessary, corticosteroid should be applied externally.
2. Acneiform rash: Corticosteroid is applied externally and, if necessary, minomycin is orally administered depending on the site and severity of rash.
3. Paronychia: Washing, protection with gauze, cooling, and taping are performed. If necessary, moisturizing agent or corticosteroid is applied externally, and minomycin is orally administered. In some situations, a dermatologist should be consulted, and liquid nitrogen cryotherapy and onicoplasty (partial matrix excision) should be considered.

If severe itching is caused by ➀ or ➁, antihistamine or antiallergic agent should be orally administered in combination. A dermatologist should be consulted as necessary.

#### 6) Diarrhea

Oral administration of an antidiarrheal agent, such as loperamide, and fluid replacement should be performed depending on the patient’s condition.

#### 7) Lung disorder

Oxygen and corticosteroid administration appropriate for the severity of hypoxemia should be performed. If pneumonitis due to infection cannot be excluded, antibiotics are used concomitantly.

#### 8) Nausea and vomiting

Regarding nausea and vomiting, antiemetics are positively administered according to Clinical Practice Guidelines for Antiemesis in Oncology^70^, and fluid and electrolyte repletion are performed when oral intake is severely reduced.

#### 9) Anorexia

If oral intake drops markedly, fluid and electrolyte supplements should be given as needed. Especially, in the cases with diabetes mellitus, the abnormality of blood sugar level and electrolyte is noticed.

#### 10) Anemia, thrombocytopenia

If anemia (hemoglobin <8.0 g/dL) or thrombocytopenia (platelet count <20,000/mm^3^) is observed, transfusions should be performed as appropriate at the discretion of the Investigator/Subinvestigator.

**11) Shock symptoms, anaphylaxis-like reactions**

In administering cisplatin and pemetrexed, the patient should be observed sufficiently, and, if related signs or symptoms are observed, appropriate measures, such as termination of the administration, should be taken.

### Concomitant/supportive therapies that need precautions

#### 1) Non-steroidal anti-inflammatory drugs (NSAIDs)

In concomitantly using NSAIDs, since there is the possibility that it increases the blood pemetrexed concentration and exacerbates adverse reactions, it is desirable to avoid their use for 5 days from 2 days before to 2 days after the day of pemetrexed administration. When NSAIDs with a long half-life (e.g., Relifen®, Naixan®, Feldene®) are used, it is desirable to avoid their administration for 8 days from 5 days before to 2 days after the day of pemetrexed administration. The use of external preparations (except suppositories) is permitted. If their concomitant use is unavoidable, the patient’s condition should be carefully observed by means such as frequent laboratory testing.

#### 2) Aminoglycoside antibiotics, vancomycin

Since aminoglycoside antibiotics and vancomycin may exacerbate nephrotoxicity of cisplatin and auditory nerve disorder, it is desirable to avoid their concomitant use.

### Acceptable concomitant therapy and supportive therapy

The concomitant use of bone-modifying agents, such as denosumab and bisphosphonates, drugs for complications, and drugs for palliation of symptoms, such as morphine, is permitted on condition that they do not interact with the anticancer agent used.

### Unacceptable concomitant treatment and supportive care

Radiation therapy for the primary disease (primary and metastatic lesions), surgery, anticancer chemotherapy other than protocol treatment, hormone therapy other than steroid, and immunotherapy are not performed during protocol treatment.

### Granulocyte colony-stimulating factor: G-CSF

* The use of G-CSF biogenerics (biosimilars) is allowed in this study.

#### 1) G-CSF primary precautionary administration*

* Primary prophylaxis: G-CSF should be administered from the first course of anticancer chemotherapy without checking for neutropenia or fever for the prevention of febrile neutropenia.

Primary prophylactic administration of G-CSF is not recommended in accordance with the "Guidelines for Appropriate Use of G-CSF, 2013 edition" and "Febrile Neutropenia (FN) Practice Guidelines by JSMO".

#### 2) Secondary prophylactic administration of G-CSF*

* Secondary prophylaxis: If febrile neutropenia has occurred, or postponement of the dosing schedule has been necessary due to prolonged neutropenia, in the previous course of anticancer chemotherapy, G-CSF should be administered prophylactically in the next course.

In Arm A, if FN occurs during the administration of gefitinib or osimertinib, the advisability of G-CSF administration for secondary prophylaxis remains an open question, since the evidence concerning whether G-CSF should be administered for secondary prophylaxis is insufficient. Therefore, whether G-CSF should be used for secondary prophylaxis or not is not stipulated in this study.

In Arm B, if febrile neutropenia has occurred in the previous course, the dose of the anticancer drug is reduced or dosing schedule is changed according to “6.3.2. 3) Cisplatin/pemetrexed dose reduction/termination criteria”. Therefore, the administration of G-CSF for secondary prophylaxis in the next and subsequent courses is not recommended.

#### 3) Therapeutic administration of G-CSF

Therapeutic administration of G-CSF should be performed according to the approved dosage and administration shown in the table below.

| Time of initiation | • When neutrophil count are below 1,000/mm^3^ and fever (38°C or higher as a general rule) occurs  • When neutrophil count <500/mm^3^ were observed |
| --- | --- |
| Dosage  Dosing regimen | • Filgrastim: 50 μg/m^2^ subcutaneously once daily or 100 μg/m^2^ intravenously once daily  • Naltograstim: 1 μg/kg subcutaneously once daily or 2 μg/kg IV once daily  • Renograstim: 2 μg/kg subcutaneously once daily or 5 μg/kg IV once daily |
| Timing of discontinuation | • If neutrophil count reach a nadir level of at least 5,000/mm^3^ after the elapse, discontinue administration.  • If the neutrophil count recover to ≥2,000/mm^3^, if there are no symptoms suspicious of infection, and if the patient's safety is determined to be ensured, discontinue or reduce the dose of the drug. |

## Post-study Treatment

Second-line treatment after termination of protocol treatment is not stipulated. As second-line treatment, CDDP+PEM combination therapy is permitted in Arm A, and PEM monotherapy is permitted in Arm B. Also, in both arms, if the administration of gefitinib or osimertinib is continued after confirmation of progression while performing other treatments, such as radiation therapy, or if gefitinib or osimertinib is administered again after other treatments, they are considered second-line treatments but are also permitted.

If either of the treatment arms is concluded to be superior by main analysis or interim analysis, the results of the trial are explained to the patients registered in the study as necessary, and the treatment considered optimal is provided in consideration of the clinical course of each patient.

Also, if protocol treatment termination criteria are met, but “continuation of protocol treatment” is clinically judged to be appropriate, Study Coordinator should be consulted through Site Investigator or Site Coordinator without making decisions at the Subinvestigator level, in principle (except when there is no time). The decision of either “termination of protocol treatment→initiation of second-line treatment” or “continuation of protocol treatment by deviating from the protocol” is made by the agreement of Study Coordinator and Site Investigator/Site Coordinator. The contents of consultation with Study Coordinator and the process of decision making must be recorded in detail in the free-text field of the treatment completion report form or CRF of the patient. If cases of “continuation of protocol treatment by deviating from the protocol” occur frequently, Study Coordinator must evaluate revision of the protocol treatment termination criteria in group conferences and by the use of the mailing list, because the protocol treatment termination criteria may be clinically inappropriate.

# Expected Adverse Events

## Expected Adverse Reactions

Expected adverse reactions in this study are as follows:

### Expected Adverse Drug Reactions

Adverse reactions expected by the protocol treatment and the drugs used in the examinations/tests specified in the protocol can be found in the most recent edition of the drug package insert.

Drug package insert can be obtained from the Search Page of the website of the Pharmaceuticals and Medical Devices Agency.

Prescription Drug Information Search Page http://www.pmda.go.jp/PmdaSearch/iyakuSearch/

### Adverse reactions expected in both arms

#### 1) Patients administered gefitinib (used only in patients registered before and in ver. 1.1)

Concerning adverse reaction to gefitinib monotherapy, findings in the gefitinib arm (87 patients) in the phase III trial of gefitinib monotherapy vs. platinum-based combination chemotherapy carried out in Japan serve as reference.^14)^

Table 7.1.2.a Adverse reactions to gefitinib in phase III trial against platinum-based chemotherapy

| Adverse events (CTCAE version 3.0) | Any grade (%) | ≥Grade 3 (%) |
| --- | --- | --- |
| Hematotoxicity |  |  |
| White blood cell count decreased | 14.9% | 0% |
| Neutrophil count decreased | 8.0% | 0% |
| Anemia | 37.9% | 0% |
| Platelet count decreased | 13.8% | 0% |
| Non-hematological toxicities |  |  |
| Nausea | 17.2% | 1.1% |
| Diarrhea | 54.0% | 1.1% |
| Constipation | 16.1% | 2.2% |
| Rash | 85.1% | 0% |
| Elevated AST | 70.1% | 16.1% |
| Elevated ALT | 70.1% | 27.6% |
| Pneumonitis | 2.2% | 1.1% |

#### 2) Patients administered osimertinib (used only in patients registered in and after ver. 2.0)

Concerning adverse reactions to osimertinib monotherapy, findings in FLAURA study (279 patients) serve as reference.^77)^

Table 7.1.2.b Adverse reaction to osimertinib in FLAURA study

| Adverse events (CTCAE version 3.0) | Any grade (%) | ≥Grade 3 (%) |
| --- | --- | --- |
| Hyponatremia |  |  |
| Lymphocyte count decreased | No data | 1% |
| Neutrophil count decreased | No data | 1% |
| Anemia | 12% | 1% |
| Platelet count decreased | No data | 1% |
| Non-hematological toxicities |  |  |
| Malaise | 14% | 1% |
| Fever | 10% | 0% |
| Anorexia | 20% | 3% |
| Nausea | 14% | 0% |
| Diarrhea | 58% | 2% |
| Vomiting | 11% | 0% |
| Constipation | 15% | 0% |
| Stomatitis | 29% | <1% |
| Dry skin | 36% | <1% |
| Rash | 58% | 1% |
| Paronychia | 35% | <1% |
| Headache | 12% | <1% |
| Elevated AST | 9% | 1% |
| Elevated ALT | 6% | <1% |
| Pneumonitis | 4% | 1% |
| Prolonged QT interval | 10% | 2% |

### Adverse reactions expected in arm B

In addition to the above adverse reactions to gefitinib or osimertinib monotherapy, adverse reactions to CDDP+PEM are expected to appear in arm B. Concerning adverse reactions to CDDP+PEM, findings in phase II trial in 50 Japanese patients with advanced non-squamous non-small-cell lung cancer serve as reference.^71)^

Table 7.1.3. Adverse reactions to CDDP+PEM in phase II trial in patients with advanced non-squamous non-small-cell lung cancer

| Adverse events (CTCAE version 3.0) | Any grade (%) | ≥Grade 3 (%) |
| --- | --- | --- |
| Hyponatraemia |  |  |
| White blood cell count decreased | 80% | 2% |
| Neutrophil count decreased | 66% | 16% |
| Anemia | 90% | 16% |
| Platelet count decreased | 28% | 4% |
| Non-hematological toxicities |  |  |
| Nausea | 80% | 0% |
| Vomiting | 10% | 0% |
| Diarrhea | 14% | 0% |
| Constipation | 70% | 0% |
| Rash | 30% | 0% |
| Serum creatinine increased | 30% | 0% |
| Elevated AST | 42% | 0% |
| Elevated ALT | 42% | 0% |
| Infection | 7% | 7% |
| Pneumonitis | 2% | 2% |

## Evaluation of adverse events/reactions

The Common Terminology Criteria for Adverse Events v4.0 Japanese JCOG Version (CTCAE v4.0-JCOG) is used to assess adverse events/reactions. For CTCAE v4.0-JCOG, AE terms for which grade is defined by the institutional standard values of laboratory test, 'JCOG Common Standard Value Ranges' are used instead of the institutional standard values at each participating institution. For more information on 'JCOG Common Standard Value Ranges', see JCOG website (http://www.jcog.jp/doctor/tool/kijun.html).

### Grading of adverse events

In grading of adverse events, each adverse event is graded to the nearest definitions among Grade 0 to 4 (nearest match).

In addition, grading should be decided to the clinical need for specific procedures that have been described in grade definitions. For example, when patients have increased pleural effusion, but refuse oxygen inhalation or chest drainage indications. In such cases, grading is based on the medical judgment of what should be done rather than on whether the treatment was actually done.

For treatment-related deaths, original NCI-CTCAE requires that the adverse event resulted in treatment-related deaths is graded as "Grade 5." However, because the outcome of the serious adverse event is reported in the Adverse Event Reporting and is discussed in detail, the Site Investigator's judgment of Grade 5 is likely to be changed finally. Additionally, because the factors other than the adverse event have a significant effect on whether or not the serious adverse event leads to death to the same extent, and it is not appropriate to compare %Grade 4 and %Grade 5 separately between treatment arms, then it is not meaningful to distinguish Grade 5 from Grade 4. Therefore, in this study, Grade 5 is not used when the CRFs are filled out, but record/report serious adverse event as Grade 4.

A speculation of the causal relationship between adverse events and death in cases of treatment-related deaths should be included in the Off-treatment Form and/or 'Situation at Death' field in the Follow-up Form, and the Expedited ADR Reporting should be made (the decision is made to be Grade 5 or not in a post-hoc review including the review of the Expedited ADR Reporting). For the adverse event items specified in the '8.3. Examination and evaluation during treatment' and '8.4. Tests and evaluation items 53 or more weeks after the initiation of protocol treatment', grade and the date of first onset of grade should be entered into the relevant CRFs (Treatment Form). If the other Grade 3 or greater adverse events or Grade 3/2/1 adverse events which require hospitalization for at least 24 hours or prolongation of hospitalization are observed (see 10.1.1.3), the AE term, grade and the date of first onset of grade should be entered in the free-text column of the Treatment Form.

Grade entered in the CRFs should always be recorded in the medical records. The records will be confirmed during site visit audit.

### Determination of the causal relationship between adverse events and treatment

When assessing the causal relationship between adverse events and treatment, it should be classified into 5 categories of "definite, probable, possible, unlikely, unrelated". Each of "definite", "probable", "possible" is defined as "causally related" and "unlikely" and "unrelated" is defined as "not causally related"(see Table 7.4.2).

If the observed adverse events meet the criteria for the expedited reporting specified in '10.1. Serious adverse event and the expedited reporting', the Site Investigator must report those adverse events to the Study Coordinator according to '10.2. Site Investigator/Representative's responsibility and the procedures of expedited reporting'.

Table 7.2.2. Criteria for causal relationships between adverse events and treatment

|  | Judgment | Vision of judgment |
| --- | --- | --- |
| Causal | Definite | The AE is clearly related to the intervention  Adverse event is considered to be apparently caused/aggravated by the protocol treatment, and it is considered unlikely to be due to disease progression or other factors (comorbidity, other drugs/treatments, accidental complications) |
|  | Probable | The AE is likely related to the intervention  Adverse event is considered to be unlikely caused/aggravated by disease progression or other factors (comorbidity, other drugs/treatments, accidental complications), and it is considered to be likely due to protocol treatment |
|  | Possible | The AE may be related to the intervention  Adverse event is considered to be more plausibly caused by protocol treatment, and unlikely to be due to disease progression or other factors (comorbidity, other drugs/treatments, accidental complications). |
| Not causal | Unlikely | The AE is doubtfully related to the intervention  Adverse event is considered to be more plausibly due to disease progression or other factors (comorbidity, other drugs/treatments, accidental complications) rather than that it was caused by the protocol treatment. |
|  | Unrelated | The AE is clearly NOT related to the intervention  Adverse event is considered to be apparently caused/aggravated by disease progression or other factors (comorbidity, other drugs/treatments, accidental complications), and it is considered not to be due to protocol treatment |

# Examination, evaluation and evaluation schedule

## Examination and evaluation before registration

### Examination and evaluation before registration (time is disregarded if they are before registration)

1. HBs antigen, HBs antibody*, HBc antibody*, HCV antibody

* If HBc-Ab positive and/or HBs-Ab are positive, HBV-DNA is measured (See, 6.4.1)

1. Histological examination of the primary, recurrent, or metastatic lesions

- Histopathological type
- Presence or absence of exon 19 partial deletion or exon 21 L858R point mutation in EGFR gene*
- Presence or absence of exon 20 T790M point mutation in EGFR gene*
- Presence or absence of KRAS mutation (identification of KRAS mutation is not essential)
- Presence or absence of ALK fusion gene (identification of ALK fusion gene is not essential)

*EGFR mutations are examined by one of the following methods.

1. Real-time PCR using COBAS®EGFR mutation detection kit
2. Real-time PCR using therascreen® EGFR mutation detection kit
3. PNA-LNA clamp method
4. PCR Invader method
5. Cycleave method
6. PCR-RFLP method
7. Loop-Hybrid method
8. Oncomine Dx Target Test multi CDx system

### Examinations performed within 28 days before registration

1. Contrast-enhanced brain MRI or contrast-enhanced brain CT (slice thickness: ≤5 mm): If a contrast agent cannot be used due to a history of allergy to contrast agent, bronchial asthma, and kidney dysfunction, plain MRI or plain CT is permitted.
2. Contrast-enhanced CT of the chest and contrast-enhanced CT of the abdomen (slice thickness: ≤5 mm): If a contrast agent cannot be used due to a history of allergy to contrast agent, bronchial asthma, and kidney dysfunction, plain CT is permitted.
3. Bone scintigraphy or FDG-PET
4. 12-lead, resting electrocardiography

### Examination and evaluation within 14 days before registration

1. General condition: PS (ECOG), body weight
2. Peripheral blood count: white blood cell count, neutrophil count (ANC: rod+segmented karyocyte), hemoglobin, platelet count
3. Blood chemistry: albumin, total bilirubin, AST (GOT), ALT (GPT), ALP, LDH, creatinine, calcium, sodium, potassium, CRP
4. Creatinine clearance (estimated value by Cockcroft-Gault formula. If the estimated value is <60 mL/min, measure the actual value)
5. Chest X-P (frontal view)
6. Cutaneous oxygen saturation: SpO_2_ (If SpO_2_ is <92%, measure PaO_2_)

## Definitions of evaluation periods

#### 1) Observation period

Until day 358 (week 52) by defining the day of the initiation of protocol treatment (day of the initiation of gefitinib administration) as day 1.

In both arms, if protocol treatment is terminated before the end of the observation period, the time until the day of termination of protocol treatment is defined as the observation period.

#### 2) Patient follow-up period

The period from the end of the observation period to death or the last follow-up examination in this study.

The patient follow-up period includes the following 2 periods (the term “patient follow-up period” is used to distinguish it from the usual “follow-up period”.)

1) From the end of the observation period to the termination of protocol treatment

2) From the termination of protocol treatment to the day of death or the last follow-up in this study

## Examination and evaluation during treatment

The following safety examination and evaluation are minimal in frequency: Performing examinations more frequently at the discretion of the treating physician is not prohibited.

However, the examination for efficacy evaluation should be performed at specified frequencies, unless progression is suspected, because dense frequency may lead to bias in the efficacy evaluation.

### Safety evaluation items in Arm A (mentioned in CTCAE v4.0-JCOG)

The examinations and observations listed in 1)-6) are performed at the following frequency by defining the day of the initiation of protocol treatment (day of the initiation of gefitinib or osimertinib administration) as day 1. The permitted range of deviation of the time of evaluation is ±1 week (e.g., between day 50 and day 64 if day 57 is the day of evaluation).

From day 1 to day 70 day 15, day 29, day 43, day 57 (No examination needed on day 70*)

From day 71 to day 133 day 71, day 92, day 113 (No examination needed on day 133*)

From day 134 to day 358 (52 weeks) day 134 (20 weeks), day 190 (28 weeks), day 246 (36 weeks),

day 302 (44 weeks), day 358 (52 weeks),

* Since the permitted range is ±1 week, the examination scheduled on day 71 may be performed on day 70 (the same applies also to day 134). Subsequent deviations of the time of evaluation are permitted if they are within the permitted range.

If protocol treatment is terminated, the examinations and observations listed in 1)-6) are performed in the same periods as above until 30 days after the termination or the day of the initiation of second-line treatment, whichever the earlier (permitted range of deviation: ±1 week). No evaluation is needed thereafter.

1. PS, body weight
2. Peripheral blood count: white blood cell count, neutrophil count (ANC: rod+segmented karyocyte) hemoglobin, platelet count
3. Biochemical tests: albumin, total bilirubin, AST (GOT), ALT (GPT), ALP, LDH, creatinine, Na, K, calcium*, CRP

* If the serum albumin level is <4.0 g/dL, calculate the corrected calcium level.

Corrected calcium level (mg/dL)=Serum calcium level (mg/dL) + [4-albumin level (g/dL)] × 0.8

1. Chest X-P (frontal view)
2. Cutaneous oxygen saturation: SpO_2_ (if SpO_2_ is <92%, measure PaO_2_)
3. Subjective and objective findings (described according to CTCAE v4.0-JCOG)

- Blood and lymphatic system disorders: Febrile neutropenia
- General disorders and administration site conditions: fever, fatigue
- Skin and subcutaneous tissue disorders: pruritus, acneiform rash, dry skin, paronychia, alopecia
- Gastrointestinal disorders: constipation, diarrhea, nausea, vomiting, oral mucositis
- Metabolism and nutrition disorders: anorexia
- Nervous system disorders: peripheral motor neuropathy
- Respiratory, thoracic and mediastinal disorders: pneumonitis
- Infections and infestations: bronchial, pulmonary, upper respiratory tract

<Item added in ver. 2.0>

1. 12-lead, resting electrocardiography (only when the patient is registered in and after ver. 2.0 and administered osimertinib)

Since prolonged corrected QT interval is observed on ECG as an adverse reaction to osimertinib at a certain frequency, 12-lead resting ECG is performed at the following frequency by defining the day of the initiation of protocol treatment (day of the initiation of osimertinib administration) as day 1 (permitted range of deviation: ±1 week).

From day 1 to day 70 day 29, day 57

From day 71 to day 133 day 71

From day 134 to day 358 (52 weeks) day 134 (20 weeks), day 190 (28 weeks), day 246 (36 weeks), day 302 (44 weeks), day 358 (52 weeks)

### Safety evaluation items in Arm B (mentioned in CTCAE v4.0-JCOG)

#### 1) Safety evaluation items during first-line gefitinib or osimertinib administration

The examinations and observations listed in 1)-6) are performed every 14 days (2 weeks) between day 1 and day 70 (day 15, day 29, day 43, day 57 (no examination is needed on day 70*)) by defining the day of the initiation of protocol treatment (day of the initiation of gefitinib or osimertinib administration) as day 1. The permitted range of deviation of the time of evaluation is ±1 week (e.g., between day 50 and day 64 if day 57 is the scheduled day of evaluation).

If protocol treatment is terminated, the examinations and observations listed in 1)-6) are performed until 30 days after the termination or the day of the initiation of second-line treatment, whichever is the earlier (permitted range of deviation: ±1 week). No evaluation is needed thereafter.

1. PS, body weight
2. Peripheral blood count: white blood cell count, neutrophil count (ANC: rod+segmented karyocyte) hemoglobin, platelet count
3. Biochemical tests: albumin, total bilirubin, AST (GOT), ALT (GPT), ALP, LDH, creatinine, Na, K, calcium*, CRP

* If the serum albumin level is <4.0 g/dL, calculate the corrected calcium level.

Corrected calcium level (mg/dL)=Serum calcium level (mg/dL) + [4-albmin level (g/dL)] × 0.8

1. Chest X-P (frontal view)
2. Cutaneous oxygen saturation: SpO_2_ (if SpO_2_ is <92%, measure PaO_2_)
3. Subjective and objective findings (described according to CTCAE v4.0-JCOG)

- Blood and lymphatic system disorders: febrile neutropenia
- General disorders and administration site conditions: fever, fatigue
- Skin and subcutaneous tissue disorders: pruritus, acneiform rash, dry skin, paronychia, alopecia
- Gastrointestinal disorders: constipation, diarrhea, nausea, vomiting, oral mucositis
- Metabolism and nutrition disorders: anorexia
- Nervous system disorders: peripheral motor neuropathy
- Respiratory, thoracic and mediastinal disorders: pneumonitis
- Infections and infestations: bronchial, pulmonary, upper respiratory tract

<Item added in ver. 2.0>

1. 12-lead, resting electrocardiography (only when the patient is registered in and after ver. 2.0 and administered osimertinib)

Since prolonged corrected QT interval of ECG is observed at a certain frequency as an adverse reaction to osimertinib, 12-lead resting ECG is performed in patients administered osimertinib on day 29 and day 57 by defining the day of the initiation of protocol treatment (day of the initiation of osimertinib administration) as day 1 (permitted range of deviation: ±1 week).

#### 2) Safety evaluation items during cisplatin+pemetrexed administration

The examinations and observations listed in 1)-6) are performed on the day of administration of cisplatin+pemetrexed or the day before in each course.

If protocol treatment is terminated, the same evaluations are performed until 30 days after the termination or the day of the initiation of second-line treatment, whichever is the earlier (permitted range of deviation: ±1 week). No evaluation is needed thereafter.

1. PS, body weight (measurement within 14 days before the initiation of each course is permitted for the body weight only)
2. Peripheral blood count: white blood cell count, neutrophil count (ANC: rod+segmented karyocyte) hemoglobin, platelet count
3. Biochemical tests: albumin, total bilirubin, AST (GOT), ALT (GPT), ALP, LDH, creatinine, Na, K, CRP
4. Chest X-P (frontal view)
5. Cutaneous oxygen saturation: SpO_2_ (if SpO_2_ is <92%, measure PaO_2_)
6. Subjective and objective findings (described according to CTCAE v4.0-JCOG)

- Blood and lymphatic system disorders: febrile neutropenia
- General disorders and administration site conditions: fever, fatigue
- Skin and subcutaneous tissue disorders: pruritus, acneiform rash, dry skin, paronychia, alopecia
- Gastrointestinal disorders: constipation, diarrhea, nausea, vomiting, oral mucositis
- Metabolism and nutrition disorders: anorexia
- Nervous system disorders: peripheral motor neuropathy
- Respiratory, thoracic and mediastinal disorders: pneumonitis
- Infections and infestations: bronchial, pulmonary, upper respiratory tract

<Item added in ver. 2.0>

1. 12-lead, resting electrocardiography (only when the patient is registered in and after ver. 2.0 and administered osimertinib)

Since prolonged corrected QT interval of ECG is observed at a certain frequency as an adverse reaction to osimertinib, 12-lead resting ECG is performed in patients administered osimertinib on day 71 by defining the day of the initiation of protocol treatment (day of the initiation of osimertinib administration) as day 1 (permitted range of deviation: ±1 week).

#### 3) Safety evaluation items during gefitinib administration after CDDP+PEM

The following examinations and observations are performed after day 22 on day 134, day 190, day 246, day 302, and day 358 by defining the day of the initiation of 3 courses of cisplatin+pemetrexed therapy (after day 134 if the day of the initiation of protocol treatment is defined as day 1) (permitted range of deviation: ±1 week).

If the treatment is advanced to gefitinib monotherapy without performing 3 courses of CDDP+PEM combination therapy or if protocol treatment is terminated, for reasons other than progression of the primary disease, the same evaluations are performed until 30 days after the termination or the day of the initiation of second-line treatment, whichever is the earlier (permitted range of deviation: ±1 week). No evaluation is needed thereafter.

1. PS, body weight
2. Peripheral blood count: white blood cell count, neutrophil count (ANC: rod + segmented karyocyte) hemoglobin, platelet count
3. Biochemical tests: albumin, total bilirubin, AST (GOT), ALT (GPT), ALP, LDH, creatinine, Na, K, calcium*, CRP

* If the serum albumin level is <4.0 g/dL, calculate the corrected calcium level.

Corrected calcium level (mg/dL)=Serum calcium level (mg/dL) + [4-albumin level (g/dL)] × 0.8

1. Chest X-P (frontal view)
2. Cutaneous oxygen saturation: SpO_2_ (If SpO_2_ is <92%, measure PaO_2_)
3. Subjective and objective findings (described according to CTCAE v4.0-JCOG)

- Blood and lymphatic system disorders: febrile neutropenia
- General disorders and administration site conditions: fever, fatigue, edematous limbs
- Skin and subcutaneous tissue disorders: pruritus, acneiform rash, dry skin, paronychia, alopecia
- Gastrointestinal disorders: constipation, diarrhea, nausea, vomiting, oral mucositis
- Metabolism and nutrition disorders: anorexia
- Nervous system disorders: peripheral motor neuropathy
- Respiratory, thoracic and mediastinal disorders: pneumonitis
- Infections and infestations: bronchial, pulmonary, upper respiratory tract

<Item added in ver. 2.0>

1. 12-lead, resting electrocardiography (only when the patient is registered in and after ver. 2.0 and administered osimertinib)

Since prolonged corrected QT interval of ECG is observed at a certain frequency as an adverse reaction to osimertinib, 12-lead resting ECG is performed in patients administered osimertinib on day 134, day 190, day 246, day 302, and day 358 by defining the day of the initiation of protocol treatment (day of the initiation of osimertinib administration) as day 1 (permitted range of deviation: ±1 week).

### Safety examination and evaluation to be performed as necessary

1. When dyspnea is observed

- Arterial blood gases: PaO_2_
- Chest X-P (frontal view)

1. If an arrhythmia is observed

- 12-lead, resting electrocardiography

### Efficacy evaluation items

The following examinations are performed by the same methods as baseline evaluation on day 57, day 134, day 190, day 246, day 302, and day 358 by defining the day of the initiation of protocol treatment as day 1, and the cytoreductive effect is evaluated according to “11.1. Efficacy evaluation”. The permitted range of deviation of the time of examination is ±1 week (e.g., between day 50 and day 64 if day 57 is the scheduled day of examination).

1. Contrast-enhanced CT of the chest (slice thickness: ≤5 mm)
2. Contrast-enhanced CT of the upper abdomen (slice thickness: ≤5 mm)
3. Concerning non-target lesions among the lesions out of the scanning range of chest/abdominal CT, such as those of the brain and bone, examinations, such as head CT, MRI, bone scintigraphy, or FDG-PET, are performed, if necessary, when symptoms appear or new lesions are suspected, and regular examinations according to the above schedule are not required.

### Presence or absence of EGFR exon 20 T790M point mutation (at the time of progression only)

After a judgment of progression of the primary disease has been made, re-biopsy is performed from the lesion (regardless of whether it is in or out of the lung) as much as possible (but examination by liquid biopsy is unacceptable).

However, re-biopsy is not essential.

1. Time of re-biopsy at the time of progression

At the time of progression, re-biopsy should be performed from the time of progression to the initiation of next chemotherapy.

However, if gefitinib administration is continued even after the judgment of progression, re-biopsy should be performed before the initiation of chemotherapy other than gefitinib.

1. The site or number of biopsies is not specified.
2. EGFR mutation testing at the time of progression

The presence or absence of EGFR exon 20 T790M point mutation is checked.

The method of testing may not be the same as that at the time of registration if it is mentioned in “4.1. Eligibility criteria (enrollment criteria)”.

1. Data concerning whether re-biopsy has been performed, site of re-biopsy, and methods of EGFR mutation testing are collected. If biopsy is performed multiple times, and if ➀ there have been positive biopsies, the data of the first biopsy that indicated positivity for EGFR exon 20 T790M point mutation are used, or if ➁ all biopsies have been negative for EGFR exon 20 T790M point mutation, the data of the first biopsy are used.

<Item added in ver. 2.0>

With the change of the standard first-line treatment to osimertinib, to which T790M mutant EGFR also responds, checking for T790M mutation at the time of progression loses significance. Therefore, in patients administered osimertinib, the percentage of patients positive for EGFR exon 20 T790M-resistant point mutation in the tumor at the time of progression, which was a secondary endpoint, is not calculated, and it is calculated only in the population administered gefitinib. However, associated research to explore the mechanism of resistance to osimertinib by performing re-biopsy at the time of progression is important, and re-biopsy continues to be recommended.

## Items of examinations and evaluations 53 or more weeks after the initiation of protocol treatment

The following evaluations are performed 53 or more weeks after the initiation of protocol treatment until a judgment of progression is made. In patients in whom protocol treatment has been terminated for reasons other than progression of the primary disease, 1)-3) are evaluated according to the following schedule regardless of whether second-line treatment is performed or not (evaluation of 4) is unnecessary in such cases).

Evaluation Items

1. Contrast-enhanced CT of the chest (slice thickness: ≤5 mm)
2. Contrast-enhanced CT of the upper abdomen (slice thickness: ≤5 mm)
3. Examinations, such as head CT, MRI, bone scintigraphy, and FDG-PET, are performed when necessary for lesions located out of the scanning range of chest/abdominal CT, such as the brain and bone, when symptoms appear or new lesions are suspected, and regular examinations according to the above schedule are not required.
4. Subjective and objective findings (mentioned in CTCAE v4.0-JCOG)

Evaluation interval (permitted range of deviation: ±1 week)

53 weeks-2 years (104 weeks) after the initiation of protocol treatment: Every 12 weeks

2 years or more (105 weeks or more) after the initiation of protocol treatment: Every 24 weeks

## Study calendar

Arm A

| Course | Before treatment | Gefitinib/osimertinib | | | | | | | | | Time of discontinuation | After discontinuation |
| --- | --- | --- | --- | --- | --- | --- | --- | --- | --- | --- | --- | --- |
| Week |  | 1-2 | 3-4 | 5-6 | 6-8 | 9-10 | 11-13 | 14-16 | 17-19 | Ongoing |  |  |
| General condition |  |  |  |  |  |  |  |  |  |  |  |  |
| Physical findings | ○^14^ | ○ | ○ | ○ | ○ | ○ | ○ | ○ | ○ | ◎^安全^ | ○ | ◎^安全^ |
| Height | ○^14^ |  |  |  |  |  |  |  |  |  |  |  |
| Body weight | ○^14^ | ○ | ○ | ○ | ○ | ○ | ○ | ○ | ○ | ◎^安全^ | ○ | ◎^安全^ |
| PS | ○^14^ | ○ | ○ | ○ | ○ | ○ | ○ | ○ | ○ | ◎^安全^ | ○ | ◎^安全^ |
| Laboratory tests |  |  |  |  |  |  |  |  |  |  |  |  |
| CBC | ○^14^ | ○ | ○ | ○ | ○ | ○ | ○ | ○ | ○ | ◎^安全^ | ○ | ◎^安全^ |
| Biochemistry | ○^14^ | ○ | ○ | ○ | ○ | ○ | ○ | ○ | ○ | ◎^安全^ | ○ | ◎^安全^ |
| CCr | ○^14^ |  |  |  |  |  |  |  |  |  |  |  |
| HBs antigen; HBs antibody; HBc antibody; HCV antibody; HBV-DNA^*^ | ○^前^ |  |  |  |  |  |  |  |  |  |  |  |
| SpO_2_ | ○^14^ | ○ | ○ | ○ | ○ | ○ | ○ | ○ | ○ | ◎^安全^ | ○ | ◎^安全^ |
| 12-lead ECG | ○^28^ |  | ○^オ^ |  | ○^オ^ |  | ○^オ^ |  |  | ◎^安全オ^ |  | ◎^安全オ^ |
|  |  |  |  |  |  |  |  |  |  |  |  |  |

○:○ Implementation, ^前^: Implementation before registration (can be done at any time prior to registration)

○^14^: Execute within 14 days prior to registration, ○^28^: Execute within 28 days prior to registration

○^オ^: Enrolled after ver. 2.0 and performed when using osimertinib as protocol treatment

◎^安全オ^: Enrolled after ver. 2.0 and performed when using osimertinib as protocol treatment
From 19 to 52 weeks after protocol treatment initiation; day 57, day 134, day 190, day 246, day 302, day 358:

◎^安全^:day 57, day 134, day 190, day 246, day 302, day 358 from 19 to 52 weeks after initiation of protocol treatment:

◎^有効^:day 57, day 134, day 190, day 246, day 302, day 358 from 19 to 52 weeks after initiation of protocol treatment:

From 53 weeks to 2 years after initiation of protocol treatment every 12 weeks (acceptable ± 1 week).

After 2 years of protocol treatment every 24 weeks (acceptable ± 1 week)

In addition, even if the protocol treatment is discontinued for reasons other than exacerbation, the above until exacerbation or death is confirmed.

Perform the test according to the schedule.

●:Performed whenever possible after the diagnosis of exacerbation of the pathogen

△:Head CT/MRI and bone scintigraphy (which can be substituted for PET) should be performed as appropriate when symptoms appear or new lesions are suspected, and regular examination according to the timeline is not mandatory

□:Submission

*: HBV-DNA is measured if the patient is HBs antibody-positive and/or HBc antibody-positive.

※Follow-up will be conducted up to 3 years after enrollment in the study, and will be reported after 3 years after enrollment for individual patients according to the cut-off date.

Arm B

| Course | Before treatment | Gefitinib/  Osimertinib | | | | Washout | CDDP+PEM | | | Gefitinib/osimertinib | Time of discontinuation | After discontinuation |
| --- | --- | --- | --- | --- | --- | --- | --- | --- | --- | --- | --- | --- |
| Week |  | 1-2 | 3-4 | 5-6 | 6-8 | 9-10 | 11-13 | 14-16 | 17-19 | Ongoing |  |  |
| General condition |  |  |  |  |  |  |  |  |  |  |  |  |
| Physical findings | ○^14^ | ○ | ○ | ○ | ○ | ○ | ○ | ○ | ○ | ◎^安全^ | ○ | ◎^安全^ |
| Height | ○^14^ |  |  |  |  |  |  |  |  |  |  |  |
| Body weight | ○^14^ | ○ | ○ | ○ | ○ | ○ | ○ | ○ | ○ | ◎^安全^ | ○ | ◎^安全^ |
| PS | ○^14^ | ○ | ○ | ○ | ○ | ○ | ○ | ○ | ○ | ◎^安全^ | ○ | ◎^安全^ |
| Laboratory tests |  |  |  |  |  |  |  |  |  |  |  |  |
| CBC | ○^14^ | ○ | ○ | ○ | ○ | ○ | ○ | ○ | ○ | ◎^安全^ | ○ | ◎^安全^ |
| Biochemistry | ○^14^ | ○ | ○ | ○ | ○ | ○ | ○ | ○ | ○ | ◎^安全^ | ○ | ◎^安全^ |
| CCr | ○^14^ |  |  |  |  |  |  |  |  |  |  |  |
| HBs antigen; HBs antibody; HBc antibody; HCV antibody; HBV-DNA^*^ | ○^前^ |  |  |  |  |  |  |  |  |  |  |  |
| SpO_2_ | ○ | ○ | ○ | ○ | ○ | ○ | ○ | ○ | ○ | ◎^安全^ | ○ | ◎^安全^ |
| 12-lead ECG | ○^28^ |  | ○^オ^ |  | ○^オ^ |  | ○^オ^ |  |  | ◎^安全オ^ |  | ◎^安全オ^ |
|  |  |  |  |  |  |  |  |  |  |  |  |  |

○:○ Implementation, ^前^: Implementation before registration (can be done at any time prior to registration)

○^14^: Execute within 14 days prior to registration, ○^28^: Execute within 28 days prior to registration

○^オ^: Enrolled after ver. 2.0 and performed when using osimertinib as protocol treatment

◎^安全オ^: Enrolled after ver. 2.0 and performed when using osimertinib as protocol treatment
From 19 to 52 weeks after protocol treatment initiation; day 57, day 134, day 190, day 246, day 302, day 358:

◎^安全^:day 57, day 134, day 190, day 246, day 302, day 358 from 19 to 52 weeks after initiation of protocol treatment:

◎^有効^:day 57, day 134, day 190, day 246, day 302, day 358 from 19 to 52 weeks after initiation of protocol treatment:

From 53 weeks to 2 years after initiation of protocol treatment every 12 weeks (acceptable ± 1 week).

After 2 years of protocol treatment every 24 weeks (acceptable ± 1 week)

In addition, even if the protocol treatment is discontinued for reasons other than exacerbation, the above until exacerbation or death is confirmed.

Perform the test according to the schedule.

●:Performed whenever possible after the diagnosis of exacerbation of the pathogen

△:Head CT/MRI and bone scintigraphy (which can be substituted for PET) should be performed as appropriate when symptoms appear or new lesions are suspected, and regular examination according to the timeline is not mandatory

□:Submission

*: HBV-DNA is measured if the patient is HBc antibody-positive and/or HBs antibody-positive.

# Data Collection

## Case Report Form (CRF）, Electronic Case Report Form (eCRF）

### CRF type and submission deadline

Record forms (Case Report Form: CRF) used in this study and time limits for submission are as follows:

1) Pretreatment report (blue) - Within 2 weeks of enrollment

2) Course records (yellow) Group A - Every 8 weeks/within 6 weeks of discontinuation

Course records (yellow) Group B (gefitinib) - every 8 weeks/within 6 weeks of discontinuation

Elapsed records (yellow) Group B (CDDP+PEM) - Within 6 weeks after completion/discontinuation of 3 courses

3) Tumor response report (green) - Within 2 weeks after each response evaluation

4) Treatment discontinuation report (red). - Within 2 weeks after protocol treatment discontinuation/completion

5) Follow-up (white and EDC) - Within the time limit notified by the data center

**[JCOG]**

・ CRF use eCRF.

・ "1) Pretreatment Report to 4) Treatment Discontinuation Report" will be displayed on JCOG Web Entry System after enrollment.

・ "5) Follow-up" will be informed by the data center through the group mailing list of the timing of follow-up.

・ Treatment of "ineligible cases" (see 14.1.2.) should be submitted according to the deadline for submitting CRF until the ineligibility is established. Submit pre-treatment reports, end-of-treatment reports, and follow-up forms even in ineligible patients. After ineligibility has been determined, submissions of CRF such as treatment course and response assessment are not required.

**<eCRF Precautions>**

- Data-entry and response to queries is done via JCOG Web Entry System.
- The timing of the query is notified by the data center through the group mailing list.
- For details on the input method, refer to the separate input manual.
- In case of incorrect answers during entry, enter details in the facility remarks column. At a later date, the study office review or groups may review and modify the data. The data correction is notified by e-mail from the data center at the timing of the inquiry, so check it.
- To avoid the risk of patient personal information leakage, the patient registration number should be used and the medical record number of the institution should not be used when contacting the data center.
- The timing of "5) follow-up" will be notified through the group mailing list by the data center.
- Details of EDC system are described in the following table.

| System name | E-DMS Online |
| --- | --- |
| System Development Corporation | EP Techno Co., Ltd. |
| Input method | Data input via Web |
| Input terminal | Personal computer of participating institutions |
| Supported browsers | Recommendations: Google Chrome; Internet Explorer |
| Method for identifying entry personnel | Personal IDs and passwords |
| Form of communication encryption | SSL (RSA key length of 2048 bits) |

**[WCOG]**

- The electronic case report system (EDC) is used to electronically record case reports. Details of the system are described in the table below.

| System name | E-DMS Online |
| --- | --- |
| System Development Corporation | EP Techno Co., Ltd. |
| Input method | Data input via Web |
| Input terminal | Personal computer of participating institutions |
| Supported browsers | Recommendations: Google Chrome; Internet Explorer |
| Method for identifying entry personnel | Personal IDs and passwords |
| Form of communication encryption | SSL (RSA key length of 2048 bits) |

### Storage of CRF

- Data entered into eCRF will be stored semi-permanently in the data center.
- EDC (Electronic Data Capturing) does not require CRF to be printed on paper and stored at the site because CRF content can be checked on EDC screen even at the site.

### CRFの送付方法

**[JCOG]**

- Emailing of files digitized by scanners is not allowed.
- All CRF will be sent to the data center by mail or handover. FAX transmission is not performed.
- To avoid the risk of patient personal information leakage, the patient registration number should be used and the medical record number of the institution should not be used when contacting the data center for CRF shipment requests, etc.

**【WJOG】**

- The site representative physicians and physicians in charge of the study will enter and transmit information on enrolled cases to EDC as a case report form in accordance with the provisions of the study protocol. The method is prescribed in a separate guide.

### Correction of the contents of CRFs

If lacking of necessary data items or inadequate categorization in the CRFs are identified after the start of the study, the CRF shall be revised in agreement with the Director of JCOG Data Center and Study Coordinator, unless the data collected are not exceeded the extent specified in '8. Examination and Evaluation' and the CRF revision is judged not to increase the medical and economic burden on registered patients.

# Reporting of Adverse Events

**[JCOG participating institution]**

In the event of "serious adverse events" or "unexpected adverse events", the Site Investigator should report to Study Coordinator/Principal Investigator in accordance with this chapter based on JCTN-Adverse Event Reporting Guidelines and JCOG Clinical Safety Information Handling Guidelines.

The most recent version of the report is available on the JCOG website (http://www.jcog.jp/doctor/todo/researcher/harmfulness.html). Use the most recent version of the report.

Serious adverse events occurring after the initiation of protocol treatment (after the date of registration if death) by the date of final follow-up are the subjects.

Reporting to the Ministry of Health, Labour and Welfare on side effects based on the "Law Concerning the Assurance of Quality, Effectiveness and Safety of Pharmaceuticals, Medical Devices" (Destination: FAX: 0120-395-390; E-mail: anzensei-hokoku@pmda.go.jp)^1)^, reporting of serious adverse event according to the "Ethical Guidelines for Medical and Health Research Involving Human Subjects" (Notification No.1 of the Ministry of Education, Culture, Sports, Science and Technology and the Ministry of Health, Labour and Welfare in 2017) ^2)^ to the administrator of medical institution, reporting of unexpected serious adverse events from the administrator of medical institution to the Minister of Health, Labour and Welfare, and communications regarding side effects from medical institution to pharmaceutical company should be appropriately conducted under the responsibility of the Site Investigator in accordance with the provisions of each participating institution.

1) http://www.pmda.go.jp/safety/reports/hcp/pmd-act/0003.html

2) http://www.mhlw.go.jp/stf/seisakunitsuite/bunya/hokabunya/kenkyujigyou/i-kenkyu/index.html

**[WJOG participating institution]** (**take the following measures concerning patients registered before and in ver. 1.1)**

In accordance with JCTN-Adverse Event Reporting Guidelines and WJOG Adverse Event Reporting Rules, reportable adverse events that have occurred are reported to WJOG Data Center. Request the report form from WJOG Data Center. WJOG members can download it from the website of WJOG.

Reporting to the Ministry of Health, Labour and Welfare on side effects based on the "Law Concerning the Assurance of Quality, Effectiveness and Safety of Pharmaceuticals, Medical Devices" (Destination: FAX: 0120-395-390; E-mail: anzensei-hokoku@pmda.go.jp)^1)^, reporting of serious adverse event according to the "Ethical Guidelines for Medical and Health Research Involving Human Subjects" (Notification No.1 of the Ministry of Education, Culture, Sports, Science and Technology and the Ministry of Health, Labour and Welfare in 2017) ^2)^ to the administrator of medical institution, reporting of unexpected serious adverse events from the administrator of medical institution to the Minister of Health, Labour and Welfare, and communications regarding side effects from medical institution to pharmaceutical company should be appropriately conducted under the responsibility of the Site Investigator in accordance with the provisions of each participating institution.

1) http://www.pmda.go.jp/safety/reports/hcp/pmd-act/0003.html

2) http://www.mhlw.go.jp/stf/seisakunitsuite/bunya/hokabunya/kenkyujigyou/i-kenkyu/index.html

## Serious Adverse Events and subjects of Expedited Reporting

Serious adverse events are defined as any of the following:

1. Death
2. Adverse events that may lead to death
3. Adverse events requiring hospitalization or prolongation of hospital stay for treatment.
4. Disability
5. Adverse events that may lead to disability
6. Serious adverse events according to 1) to 5)
7. Congenital disease or abnormality in later generations

Of these, those that fall under any of the following categories shall be regarded as serious adverse events for which Expedited Reporting is mandatory

**1) Death**

➀ All deaths that occur after registration and before the start of protocol treatment

➁ All deaths (with or without causality to protocol treatment) that occur during protocol treatment or within 30 days of the last treatment day.

➂ Death that occur after 31 days from the last treatment date that are causally related to protocol treatment (definite, probable, possible)

**2) Adverse events that may lead to death**

➀ Grade 4 adverse events that occur during protocol treatment or within 30 days of the last treatment day (excluding events in Table 10.1)

➁ Grade 4 adverse events that occur after 31 days from the last treatment date (excluding events in Table 10.1) that are causally related to the protocol treatment (definite, probable, possible)

**3) Unexpected*^2^ adverse events requiring hospitalization or prolongation of hospital stay^*1^ for treatment**

➀ Grade 3/2/1 adverse events that occur during or within 30 days of protocol treatment and requiring at least 24 hours of hospitalization or prolongation of hospital stay*^1^ to treat the adverse event (excluding the event in Table 10.1)

➁ Grade 3/2/1 adverse events that occur 31 days after the last treatment day and requires 24-hour or longer hospitalization or prolongation of hospital stay*^1^ for treatment and causally related to protocol treatment (definite, probable, possible) (excluding the events in Table 10.1)

*^1^ "Hospitalization or prolongation of hospital stay" refers only to those for which hospitalization of at least 24 hours or prolongation of hospital stay is medically required for the treatment of an adverse event. The followings are not subjects for reporting:

- Hospitalization or prolongation of hospital stay performed for follow-up of adverse event that has disappeared or improved
- Hospitalization or prolongation of hospital stay for reducing patient burden, e.g. patients from distant areas.
- Hospitalization or prolongation of hospital stay for other medically unnecessary situation

*^2^ “Unexpected” refers to those not listed in “7. Expected Adverse Events”.

**4) Disability, 5) Adverse events that may lead to disability, which is unexpected**

Permanent or marked disability/dysfunction (excluding myelodysplastic syndromes, secondary cancers) or possible medical situation

**6) Serious adverse events similar to 1) to 5), which is unexpected**

**7) Congenital disorders or abnormalities in later generations, which is unexpected**

Table 10.1. Adverse events excluded from the subjects of Expedited Reporting

| SOC* (CTCAE ver4.0) | AE term |
| --- | --- |
| Blood and lymphatic system disorders | Anemia, bone marrow hypocellular |
| Gastrointestinal disorder | Constipation |
| General disorders and administration site conditions | Fever |
| Laboratory tests | Alkaline phosphatase increased, CD4 lymphocytes decreased, cholesterol high, CPK increased, GGT increased, lipase increased, lymphocyte count decreased, neutrophil count decreased, platelet count decreased, serum amylase increased, white blood cell decreased |
| Metabolism and nutrition disorders | Obesity, anorexia, hyperuricemia, hypoalbuminemia, hypertriglyceridemia, hypoglycemia, hypokalemia, hypomagnesemia, hyponatremia |
| Renal and urinary disorders | Chronic kidney disease |
| Respiratory, thoracic and mediastinal disorders | Sleep apnea |
| Skin and subcutaneous tissue disorders | Hypohidrosis |

* SOC: System Organ Class

## Site Investigator/Representative's responsibility and the procedures of expedited reporting

Site Investigator of JCOG follows 10.2.1. Site Representative of WJOG follows 10.2.2.

### JCOG participating institution: Expedited Reporting

In the event of a serious adverse event specified in 10.1. for which expedited reporting is mandatory, the Subinvestigator must promptly inform the Site Investigator. If the Site Investigator cannot be contacted, the Site Coordinator or Subinvestigator must take over the responsibility of the Site Investigator. The investigator must report adverse events according to the following procedures. Attention should be paid not to include the patient's name and medical record number when sent. Serious adverse events that occur after the initiation of protocol treatment (after the date of registration if death) by the date of final follow-up are subjects of Expedited Reporting.

#### 1) Adverse events that may lead to death or death specified in 10.1 1) and 2)

#### Primary reporting:

The Subinvestigator who is aware of the occurrence of adverse events will promptly notify the Site Investigator. The Site Investigator who receives the notice should fill out “JCOG Adverse Event Report Form” as far as possible and contact Study Coordinator via e-mail, fax, or telephone within 72 hours of knowledge of the occurrence of the adverse event.

#### Secondary reporting:

The Site Investigator should add detailed information on adverse events to “JCOG Adverse Event Report Form”, and send them to the Study Coordinator via e-mail, fax, postal mail, or hand delivery within 7 days of knowledge of the occurrence of the adverse event. If necessary, attach copies of laboratory data, images, and autopsy report.

#### 2) Unexpected adverse events that require hospitalization or prolongation of hospital stay for treatment (10.1. 3)) or other unexpected adverse events judged to be medically important conditions (10.1. 4)-7))

The Subinvestigator who is aware of the occurrence of adverse events will promptly notify the Site Investigator. The Site Investigator who receives notice must fill out “JCOG Adverse Event Report Form” within 10 days of knowledge of the occurrence of an adverse event and send them to the Study Coordinator via e-mail. If necessary, attach copies of laboratory data, images, and autopsy report.

#### 3) Additional reporting

If new information is obtained after conducting the above reporting, the Site Investigator must add information to “JCOG Adverse Event Report Form (for institution)”, and report it as needed.

Table 10.2.1. Summary of adverse events which are subjects for Expedited Reporting and the deadline of reporting to Study Coordinator

| Causal relationship | The patient was hospitalized due to  Grade 1-3 AE.  Other medically important conditions* | | Grade 4 | | Death | |
| --- | --- | --- | --- | --- | --- | --- |
|  | Expected | Unexpected** | Expected | Unexpected | Expected | Unexpected |
| Present | No need of reporting | Primary reporting: within 10 days  Additional reporting: as needed | Primary reporting: within 72 hours  Secondary reporting: within 7 days  Additional reporting: as needed | | | |
| None | <Only on-treatment or within 30 days of last protocol treatment day> | | | | | |
|  | No need of reporting | Primary reporting: within 10 days  Additional reporting: as needed | Primary reporting: within 72 hours  Secondary reporting: within 7 days  Additional reporting: as needed | | | |

* 4) Disability, 5) Adverse events that may lead to disability, and 6) Adverse events that are serious similar to 1) to 5) specified in 10.1., 7) Congenital disorders or abnormalities in later generations.

** “Unexpected” refers to those not listed in “7. Expected Adverse Events”

### WJOG participating institution: Expedited Reporting

In the event of an adverse event specified in 10.1. for which expedited reporting is mandatory, the Site Investigator must promptly inform WJOG Data Center.

#### 1) Death or Grade 4 adverse events

#### Primary reporting:

The Site Investigator should fill out “WJOG Adverse Event Report Form” as far as possible and contact WJOD Data Center via fax within 72 hours of knowledge of the occurrence of the adverse event.

#### Secondary reporting:

The Site Investigator should add detailed information on adverse events to “WJOG Adverse Event Report Form”, and send them to WJOD Data Center via fax within 7 days of knowledge of the occurrence of the adverse event. If necessary, attach copies of laboratory data, images, and autopsy report.

#### 2) 10.1.3) Adverse events of ≤Grade 3 or other adverse events judged to be medically important conditions

The Site Investigator should fill out detailed information on adverse events to “WJOG Adverse Event Report Form”, and report them to WJOD Data Center within 10 days of knowledge of the occurrence of the adverse event. If necessary, attach copies of laboratory data, images, and autopsy report.

#### 3) Additional reporting

If new information is obtained after making the above report, it is reported as needed.

Summary of adverse events which are subjects for Expedited Reporting and the deadline of reporting.

| Causal relationship | Grade 1/2/3 | | | | Grade 4 | | Death | | Other medically important conditions |
| --- | --- | --- | --- | --- | --- | --- | --- | --- | --- |
|  | Expected | | Unexpected | | Expected | Unexpected | Expected | Unexpected |  |
|  | Hospitalized | Without hospitalization | Hospitalized | Without hospitalization |  |  |  |  |  |
| Present | No need of reporting | No need of reporting | No need of reporting | Primary reporting: within 10 days  Additional reporting: as needed | Primary reporting: within 72 hours  Secondary reporting: within 7 days  Additional reporting: as needed | | | | Primary reporting: within 10 days  Additional reporting: as needed |
| None | No need of reporting | No need of reporting | No need of reporting | <Only on-treatment^*^ or within 30 days of last protocol treatment day> | | | | | |
|  |  |  |  | Primary reporting: within 10 days  Additional reporting: as needed | Primary reporting: within 72 hours  Secondary reporting: within 7 days  Additional reporting: as needed | | | | Primary reporting: within 10 days  Additional reporting: as needed |

* However, deaths after registration but before the initiation of protocol treatment are also included as matters of emergency reporting, because evaluation of eligibility at the time of registration may be necessary.

### Report to the administrator of the medical institution

JCOG participating institution: In the event of an adverse event that is subjects of an urgent report, the Site Investigator shall report to the head of the relevant medical institution as a serious adverse event in the “Ethical Guidelines for Medical Research with Human Subjects” in accordance with the provisions of the relevant medical institution.

WJOG participating institution: The Site Investigator shall report to the head of the relevant medical institution in accordance with the provisions of the relevant medical institution.

### Reporting to other destinations

#### Reporting of safety information on pharmaceuticals, medical devices, regenerative medicine product:

In accordance with Article 68-10, Paragraph 2 of the Law Concerning the Assurance of Quality, Efficacy, and Safety of Pharmaceuticals and Medical Devices, the investigator should appropriately report to the MHLW in accordance with the regulations of each medical institution.

## Responsibilities of Principal Investigator/Study Coordinator

### Determination of necessity of suspension of registration and emergency notification to institutions

JCOG Coordinator reported from Site Investigator and WJOG Coordinator reported from WJOG Director of Data Center must report and consult with Coordinator of the other group, JCOG/WJOG Principal Investigators, and JCOG/WJOG Group Chairs, determine the urgency, importance, and impact of the report, and, if necessary, take measures such as suspending registration (notifying the Data Centers and all participating institutions) and urgently communicating information to participating institutions. Data Center and institutions may be contacted by telephone depending on the degree of urgency, but they should also be promptly informed by document (e-mail).

### Reporting to Data and Safety Monitoring Committee

#### [JCOG participating institution]

#### 1) Reporting from Study Coordinator to Data and Safety Monitoring Committee Office

If the adverse event reported from the institution is considered to be an adverse event specified in 10.2.1.1) 2), Study Coordinator should consult with Principal Investigator and Group Chair, and then contact Data and Safety Monitoring Committee Office by e-mail within 72 hours of knowledge of the occurrence of the adverse event.

In doing so, to the extent feasible, Study Coordinator should send "JCOG Adverse Event Report Form (for institution)" sent from the institution and attach "JCOG Adverse Event Report (for Study Coordinator)" with Study Coordinator's view (including judgments of causality and expectation, and judgments of continuation/discontinuation of the study)". For the expected adverse events of 10.2.1. 1) and 2), include a discussion not only of the individual patient's course but also of whether the frequency of appearance is within the expected range.

#### 2) Additional reporting

After receipt of secondary or additional reports from the institution, Study Coordinator/Principal Investigator should add additional information from the primary report and their views to "JCOG Adverse Event Report (for Study Coordinator)" and promptly contact Data and Safety Monitoring Committee Office via e-mail. If the report was sent to Institutional Review Board in the primary reporting, the secondary reporting and additional reporting must be made in the same manner.

#### [WJOG participating institution]

All adverse events reported from WJOG institutions must be checked by WJOG Director of Data Center and, if judged to be necessary, discussed among the persons involved. If judged to be necessary, WJOG Data and Safety Monitoring Committee Chairperson must consult Data and Safety Monitoring Committee for evaluation of whether the trial should be continued due to the reported event primarily through the approval procedure and notify the result to WJOG Principal Investigator, WJOG Study Coordinator, and WJOG Data Center.

Also, WJOG Study Coordinator (or WJOG Director of Data Center acting as a surrogate) must report the result of the discussion among the persons involved to JCOG Data and Safety Monitoring Committee in written form (e-mail permitted).

### Notification to researchers in participating institution

#### [JCOG]

When reported to Data and Safety Monitoring Committee Office, Study Coordinator/Principal Investigator should inform (e-mail) the Site Investigators of all participating institutions of the confirmation form issued by Data and Safety Monitoring Committee Office, “JCOG Adverse Event Report (for Study Coordinator)” and “JCOG Adverse Event Report (for institution)”. In addition, Study Coordinator/Principal Investigator can notify the Site Investigators of participating institutions without waiting for Data and Safety Monitoring Committee Office's confirmation form if there are details to be urgently disseminated.

Even if not reported to Data and Safety Monitoring Committee Office, Study Coordinator/Principal Investigator must inform the Site Investigator of the reporting institution of the decision of Study Coordinator/Principal Investigator in written form (e-mail permitted).

#### [WJOG]

WJOG Data Center notifies the results of the conference of the persons involved to Site Representatives of all WJOG group participating institutions and JCOG Study Coordinator. If necessary, WJOG Study Coordinator (or WJOG Director of Data Center acting as a surrogate) informs JCOG Study Coordinator, JCOG/WJOG Principal Investigators, and JCOG/WJOG Group Chairs of the contents of the advice and discuss subsequent measures to take.

Also, WJOG Study Coordinator/Principal Investigator notifies Site Investigators of all WJOG participating institutions of the contents of the evaluation/recommendation by JCOG Data and Safety Monitoring Committee about the adverse events at JCOG participating institutions reported through JCOG Study Coordinator in written form (e-mail permitted).

### Assessment of Adverse Events in Periodic Monitoring

During Periodic Monitoring, the Principal Investigator/Study Coordinator of JCOG and WJOG should carefully review the adverse events in the Monitoring Reports issued by the Data Center and ensure that there is no omission of reports from the participating institutions. It should also be confirmed that all reported adverse events are listed in the Monitoring Reports. The presence or absence of omitted reports should be indicated in the column of the results of Group review on the Periodic Monitoring Report.

### Procedure for making important decisions such as termination of trial

If an important decision, such as termination of trial, is necessary, consultations among JCOG and WJOG Study Coordinators, Principal Investigators, Group Chairs are held, and the final decision is made by the agreement of the 6 parties.

This trial is defined as a single study jointly conducted by JCOG and WJOG, and, if the trial is terminated in one group, it is not continued in the other group. If termination of the trial is recommended by JCOG Data and Safety Monitoring Committee, the decision of whether the trial should be altogether terminated is made by a conference of the above 6 parties.

## Responsibilities of the Site Investigator/Representative of participating institution (including reporting institution)

Also, if the adverse event in question is a subject of expedited reporting, the Site Investigator/Site Representative must report it as a “serious adverse event” defined by “Ethical Guidelines for Medical and Health Research Involving Human Subjects” to the administrator of his/her own institution according to its internal rules.

## Responsibility of the administrator of the institution where the adverse event occurred

If an unexpected serious adverse event that is considered to be related to the protocol treatment (definite, probable, possible) occurs during the course of this study, the administrator of the institution where the adverse event occurred must report to the Minister of Health, Labour and Welfare in accordance with the provisions of the “Ethical Guidelines for Medical and Health Research Involving Human Subjects” and publicize the results of the review by the Institutional Review Board.

## Evaluation by Data and Safety Monitoring Committee

#### [JCOG]

Data and Safety Monitoring Committee Office checks the content of adverse event reports reported by Study Coordinator/Principal Investigator according to the procedures described in 10.3.2, reviews the causality and expectation, and, if there is any doubt, asks Principal Investigator/Study Coordinator to reconsider them. If there is no doubt, the Secretary-General of Data and Safety Monitoring Committee Office issues a confirmation form and adverse event report is not reviewed by Data and Safety Monitoring Committee.

If there is a discrepancy between the opinions of Principal Investigator/Study Coordinator and the Data and Safety Monitoring Committee Office, and it is determined that the appropriateness of the institutional response to adverse events or the appropriateness of continuation of the study needs to be discussed, the Secretary-General of Data and Safety Monitoring Committee Office reports it to the Data and Safety Monitoring Committee and asks the final judgment to the Chair of Data and Safety Monitoring Committee (which may be reviewed in a meeting or by document review). In such cases, a notice of the review results is issued.

The information used in the review (e.g., adverse event reports) will be stored semi-permanently at the Data and Safety Monitoring Committee Office in the JCOG Operations Office.

#### [WJOG]

WJOG Data and Safety Monitoring Committee must check the contents of the report according to “WJOG Rules for Reporting of Adverse Events” and, if necessary, make recommendations about future responses including whether registration should be continued or protocol revision is necessary to WJOG Principal Investigator, WJOG Study Coordinator, and WJOG Data Center in written form.

# Response Evaluation and Endpoint Definition

## Response evaluation

Tumor response evaluation is performed according to the following instructions in compliance with the Japanese JCOG version of "New response evaluation criteria in solid tumors: Revised RECIST guideline (version 1.1)"^72)^. The original article of RECIST version 1.0 stipulated in the Introduction that "use in the context of decisions regarding continuation of therapy is not the primary focus of this document." and a similar description is seen also in RECIST version 1.1 as follows:

"Many oncologists in their daily clinical practice follow their patients’ malignant disease by means of repeated imaging studies and make decisions about continued therapy on the basis of both objective and symptomatic criteria. It is not intended that these RECIST guidelines play a role in that decision making, except if determined appropriate by the treating oncologist."

Therefore, the "overall response" as determined by response evaluation in accordance with the RECIST guidelines should be used to determine whether “a drug or treatment regimen shows promising study results worthy for continuing research and development of them”. In other words, the judgment of whether or not to continue treatment in individual patients should not be based on CR/PR/SD/PD of the overall response, but rather on the clinical judgment, which is based on a comprehensive consideration of symptoms, physical findings, and various laboratory data as well as imaging findings.

Therefore, there are cases where it is clinically appropriate to continue the protocol treatment even when it is judged as "PD (Progressive Disease)" as the overall response by response evaluation based on the imaging diagnosis. However, although whether or not to continue protocol treatment should be decided by clinical judgment regardless of response evaluation in those cases, the event date of progression-free survival should be the date on which the overall response is judged to be PD. This is due to three reasons: (i) the decision whether or not to continue protocol treatment for each arm may differ; (ii) the RECIST guidelines are the criteria which intend to standardize progression-free survival as well as response proportion (response rate); and (iii) the standard definition in the US Cooperative Groups is that the event date of progression-free survival is defined as the date on which the overall response is judged to be PD in any situation.

On the other hand, if the patient does not meet the criteria for "PD" according to response evaluation criteria based on imaging, but the treating physician judges "clinical progression" based on clinical and comprehensive judgment not based on imaging, protocol treatment should be terminated according to "6.2.2. Protocol treatment termination criteria". If the patient is judged as "clinical progression", the event date of progression-free survival should be the date on which judged as "clinical progression", even if not judged as "PD" by response evaluation. This is because imaging after a patient is judged to have "clinical progression" is often not done on schedule, so if "clinical progression" is not an event for progression-free survival, progression-free survival is likely to be overestimated. It should be noted that treating "clinical progression" as "censoring" in progression-free survival is also statistically incorrect (informative censoring) because it would handle the patients at increased risk of progression or death as censored cases.

In the original article of RECIST v1.1, it is described that "unequivocal progression" in the PD criteria for non-target lesions is "an overall level of substantial worsening in non-target disease such that the overall tumor burden has increased sufficiently to merit discontinuation of therapy," and therefore the judgment of PD in non-target lesions includes "judgment of whether or not to continue treatment in individual patients". It is confusing. It should be noted that this "unequivocal progression" is only a criterion for "PD in non-target lesions."

The relationships among 'PD', 'clinical progression', 'progression', and the events of progression-free survivals are as shown in Figure 11.1. in JCOG.


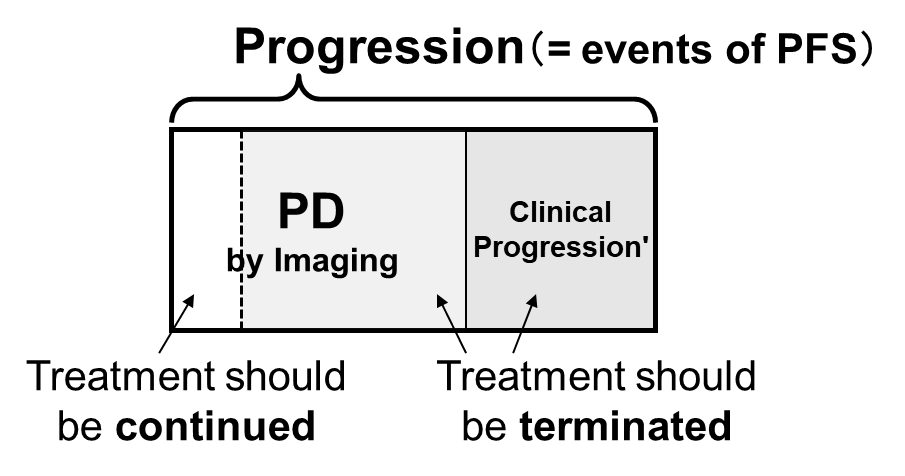


Figure 11.1. Relationship among Progression, PD by Imaging, and Clinical Progression.

### Baseline Evaluation

According to "8.1. Baseline examination and evaluation before registration", tumorous lesions at baseline are identified and categorized into "measurable lesions" and "non-measurable lesions" by chest CT (slice thickness ≤5 mm), regional MRI (slice thickness ≤5 mm), and CT or MRI including regional lymph node sites before registration.

Measurement of tumor diameter is carried out on the axial plane of MRI, and the measurement on 3-dimensional reconstruction image of CT and sagittal or coronal plane of MRI are not used. Baseline evaluations are done using the most recent imaging within 28 days prior to registration. If the other imaging studies are performed after registration and before initiation of protocol treatment, the most recent imaging studies should be used.

### Definition of measurable lesions

A measurable lesion is defined as a lesion that meets any of the following.

- 1. A lesion that fulfills any of the following and does not involve a lymph node (non-nodal lesion)

➀ Having a maximum diameter of ≥10 mm on CT with a slice thickness of ≤5 mm (MRI is acceptable for the brain)

➁ Osteolytic bone metastasis having soft tissue components that fulfills ➀ or ➁

➂ Cystic metastasis that fulfills ➀ or ➁ with no other measurable non-cystic lesion

- 1. Malignant lymph nodes ≥15 mm in short axis on CT with slice thickness ≤5 mm

(Lymph nodes with ≥10 to < 15 mm in short axis are considered non-target lesions, and those with < 10 mm in short axis are not considered as malignant lymph nodes.)

All lesions other than those listed above are considered non-measurable lesions.

Note that the following lesions are all regarded as non-measurable lesions regardless of the examination method or size.

- Bone lesions (other than osteolytic lesions with measurable soft tissue components)
- Cystic lesions (except 1)-➂ above)
- Lesions with a history of topical treatment such as radiation therapy
- Pial/meningeal lesions
- Ascites, pleural effusion, pericardial effusion
- Skin or lung lymphangiosis
- Abdominal mass or enlargement of an abdominal organ that is palpable but not measurable by imaging examinations

### Selection of target lesions and baseline documentation

Up to a maximum of five measurable lesions at baseline (a maximum of to two lesions per organ*), in descending order of diameter (longest diameter for non-nodal lesions and short axis diameter for nodal lesions) should be identified as target lesions. Target lesions should be selected on the basis of their size (lesions with the longest diameter), representative of all involved organs, but in addition should be those that lend themselves to reproducible repeated measurements (avoiding lesions that are difficult to measure even if they are large in diameter).

For selected target lesions, the region (code), examination methods, date of examination, longest diameter of non-nodal lesions, short axis diameter of nodal lesions, and the sum of the diameters of all target lesions (hereinafter referred to as the sum of diameters) are reported from head to tail in “Pre-treatment Evaluation Form”.

*How to count “organs”

1) Bilateral organs (e.g., lungs, kidneys) are counted as one with the right and left combined

2) All lymph nodes are regarded as one organ regardless of the region

### Baseline documentation of non-target lesions

All other lesions which are not selected as target lesions should be identified as non-target lesions regardless of whether or not they are measurable, and those site (code), examination methods, and date of examination are reported in “Pre-treatment Evaluation Form”. Multiple non-target lesions in the same organ may be recorded as a single lesion (e.g., multiple enlarged pelvic lymph nodes, multiple liver metastases).

### Determining objective tumor response

Target lesions and non-target lesions are evaluated by the same methods as those at the time of registration according to "8.2 Examination and evaluation during treatment" on day 57, day 134, day 190, day 246, day 302, and day 358 by defining the day of initiation of protocol treatment as day 1, and the diameters of target lesions and the disappearance or progression of non-target lesions are recorded in “Response Evaluation Form”.

### Response evaluation criteria for target lesions

**• Complete Response (CR):**

Disappearance of all target lesions. Any pathological lymph nodes (whether target or non-target) must have reduction in short axis to<10 mm. When lymph nodes are included as target lesions, the ‘sum’ of lesions may not be zero even if complete response criteria are met.

**• Partial Response (PR):**

At least a 30% decrease in the sum of diameters of target lesions, taking as reference the baseline sum of diameters.

**• Progressive Disease(PD):**

At least a 20% increase in the sum of diameters of target lesions, taking as reference the smallest sum on study (this includes the baseline sum if that is the smallest on study). In addition to the relative increase of 20%, the sum must also demonstrate an absolute increase of at least 5 mm.

**• Stable Disease (SD):**

Neither sufficient shrinkage to qualify for PR nor sufficient increase to qualify for PD, taking as reference the smallest sum of diameters while on study.

**• Not all Evaluated (NE):**

If some of examinations could not be performed for any reason or if neither CR, PR, PD, or SD could be determined.

Pre-treatment sum of diameters - sum of diameters at the time of study

%Decrease of sum of diameters = ------------------------------------------------------------------------------------ x 100%

Pre-treatment sum of diameters

Sum of diameters at examination - minimum sum of diameters

%Increase of sum of diameters = ------------------------------------------------------------------------------------ x 100%

Minimum sum of diameters

* The diameters of the target lesions should be measured, and their actual measurements should be recorded whenever measurable (e.g., <5 mm). However, if the diameter of a target lesion is judged to be “too small to measure”, it is recorded as 0 mm if the tumor is judged not to remain and as 5 mm if it is judged to remain regardless of the slice thickness of CT.

* If %Decrease meets the criteria of PD and %Increase meets the criteria of PR simultaneously, the tumor response should be PD.

* If a lesion fragments during treatment, the diameter of each fragment is added to the sum of diameters.

* If multiple lesions coalesce and their borders become indistinguishable during treatment, the sum of the coalesced lesions is added to the sum of diameters. If lesions are in contact with each other but their borders remain distinguishable, the diameter of each lesion is added to the sum of diameters.

### Response evaluation criteria for non-target lesions

**• Complete Response (CR):**

Disappearance of all non-nodal non-target lesions and decrease of the short axis diameter of all nodal non-target lesions to <10 mm.

**• Non-CR/Non-PD:**

Persistence of one or more non-target lesion(s) (including persistence of nodal non-target lesions with a short axis diameter of ≥10 mm)

**• Progressive Disease (PD);**

Unequivocal progression of existing non-target lesions (including recurrences).

When the patient also has measurable disease. In this setting, to achieve ‘unequivocal progression’ on the basis of the non-target lesions, there must be an overall level of substantial worsening in non-target lesions such that, even in presence of SD or PR in target lesions, the overall tumor burden has increased sufficiently to merit discontinuation of therapy. If the response of the target lesions is SD or PR, the increase in tumor burden of the non-target lesions to a much greater extent than the decrease in tumor burden in the target lesions is considered as ‘unequivocal progression’ and if not, response of the non-target lesions should be Non-CR/Non-PD.

Having only non-measurable lesions: An ‘unequivocal progression’ is defined as an increase in non-target lesions that is judged to clearly exceed the tumor burden corresponding to a 20% increase in diameter and a 73% increase in tumor volume.

**• Not all Evaluated (NE):**

If some examinations could not be performed for any reason or if neither CR, Non-CR/Non-PD, nor PD could be determined.

### New lesions

If a lesion that was not present at baseline was present after initiation of treatment, it should be considered as "new lesion".

However, a "new lesion" must not be a change in the image due to a difference in the imaging methods or a change in the imaging modality from the examination at the baseline evaluation, or a change in the imaging findings thought to representing something other than the tumor. For example, a cystic lesion arising within a lesion due to necrosis of a liver metastatic lesion is not considered a new lesion. New lesions are defined as new lesions by examination of sites that were not mandatory at baseline (evaluation before registration).

If a lesion disappears and later reappears, measurements should be continued. However, the response at the time the lesion reappears depends on the status of the other lesions. If the lesions reappear after CR, it is judged as PD at the time of reappearance. On the other hand, if the overall response is PR or SD, the once disappeared lesion reappears, the diameter of the lesion should be added to the sum of diameters of the remaining lesions to evaluate response. That is, in the condition where many lesions remain, even if a lesion reappears after an disappearance in imaging only, it is not considered to be PD, and it is considered to be PD when the sum of the diameters of all target lesions meets the criteria for PD. This is due to the realization that most lesions do not truly 'disappear' and are not only depicted due to the limitation of resolution of the imaging modalities used.

If a new lesion is suspected but not definitive, it should not be a new lesion, and imaging studies should be repeated at a clinically relevant time interval. When a new lesion is confirmed by repeated imaging, it is judged as the new lesion at the date of imaging at which the new lesion is confirmed.

If a FDG-PET positive lesion (with an FDG uptake exceeding two times that of the surrounding tissue in the attenuation correction image) appears in an area that was FDG-PET negative at baseline, it is regarded as a new lesion.

If a FDG-PET positive lesion appears after the initiation of treatment without baseline FDG-PET, the lesion is regarded as a new lesion when a lesion not observed at baseline is confirmed by CT or MRI in the FDG-PET positive area.

### Overall Response

The overall response is determined by combining the response of the target lesions, the response of the non-target lesions, and the presence or absence of new lesions according to Table 11.1.9.a below on day 57, day 134, day 190, day 246, day 302, and day 358 by defining the day of the initiation of treatment in Arm A and the day of initiation of first-line gefitinib or osimertinib administration in Arm B as day 1. The overall response without non-target lesions at baseline is determined by combining the response of the target lesions and the presence or absence of new lesions according to Table 11.1.9. The permitted range of deviation of the time of examination is ±1 week (e.g., day 49-day 63 if the scheduled day of examination is day 56).

Table 11.1.9. Overall response at each time of evaluation: when there are target lesions (regardless of whether there are non-target lesions).

| Target lesions | Non-target lesions | New lesions | Overall response |
| --- | --- | --- | --- |
| CR | CR | No | CR |
| CR | Non-CR/non-PD | No | PR |
| CR | Not evaluated | No | PR |
| PR | Non-PD or not all evaluated | No | PR |
| SD | Non-PD or not all evaluated | No | SD |
| Not all evaluated | Non-PD | No | NE |
| PD (clear progression) | Not questioned | Yes or No | PD |
| Not questioned | PD | Yes or No | PD |
| Not questioned | Not questioned | Yes | PD |

### Best Overall Response

Responses are rated in the descending order of CR>PR>SD>PD>NE, and the best of the overall responses observed throughout the course is regarded as the best overall response.

However, for the best overall response to be SD, the overall response 6 weeks or more after the initiation of treatment must be SD.

If any response evaluation by imaging cannot be performed due to early symptomatic deterioration or death before the first response evaluation. In addition, if any response evaluation by imaging cannot be performed by the early termination of the protocol treatment due to toxicity or patient refusal before the first response evaluation, the best overall response should be NE.

## Definitions of analyses sets

The analysis sets used in periodic central monitoring, interim analysis, and final analysis are defined as follows: The flow diagram below shows the analysis sets.

### All registered patients

Among the patients enrolled according to "5.1. Registration procedures", the group excluding duplicate registration and mis-registration is regarded as all registered patients.

### All eligible patients

The group excluding "ineligible cases (post hoc ineligibles, de facto ineligibles, violation of registration)" determined by group review from all registered patients is regarded as all eligible patients. "Ineligible cases" as judged by the Subinvestigator or Site Coordinator or Site Investigator alone are included in all eligible patients.

### All treated patients

Among all registered patients, all patients who received some or all of the protocol treatments are regarded as "all treated patients".

Data Center is allowed to determine "non-treated patients" who received no protocol treatment, and to determine whether exclude them from the safety analysis under the consent by the Study Coordinator. Whether ineligible patient is included in the analysis sets or not is determined after reviewing the ineligibility by the Study Coordinator in consultation with JCOG Data Center and WJOG Data Center.

### Patients who have undergone CDDP+PEM combination therapy (Arm B alone)

Among all treated patients in Arm B, all patients who have undergone part or all of CDDP+PEM combination therapy are regarded as “patients who have undergone CDDP+PEM combination therapy”

The identification of patient who have not undergone CDDP+PEM combination therapy as “patients untreated by CDDP+PEM combination therapy” and whether they are excluded from safety analysis may be determined by Data Center with approval of Study Coordinator. Also, whether ineligible cases are included in analysis sets is determined by Study Coordinator after evaluation of the contents of ineligibility and consultation by with JCOG Data Center and WJOG Data Center.

### Patients administered gefitinib or osimertinib* after CDDP+PEM (Arm B alone)

* Gefitinib is administered to patients registered before and in ver. 1.1, and osimertinib is administered to those registered in and after ver. 2.0

All patients who have been administered gefitinib or osimertinib even once after CDDP+PEM combination therapy are regarded as “patients administered gefitinib or osimertinib after CDDP+PEM”.

The identification of “patients not administered gefitinib or osimertinib after CDDP+PEM”, who have not been administered gefitinib or osimertinib even once after CDDP+PEM, and whether they are excluded from safety analysis may be determined by Data Center with approval by Study Coordinator. Also, whether ineligible cases are included in analysis sets is determined by Study Coordinator after evaluation of the contents of ineligibility and consultation with JCOG Data Center and WJOG Data Center.

## Definition of endpoints

| Endpoints | Event (whichever is the earlier) | | Day of censoring |
| --- | --- | --- | --- |
| Overall survival (OS) | Death due to all causes | - | Last day of confirmation of survival |
| Progression-free survival (PFS) | Death due to all causes | Progression/  recurrence | Last day of confirmation of no clinical progression |

### Overall survival

The duration from the date of registration to the date of death from any cause.

- Survivors are censored at the date of final survival confirmation (survival confirmation by telephone contact is also permitted, but the fact that survival confirmation was performed should be recorded in the medical record).
- Patients lost to follow-up are censored at the last date of survival confirmation before lost to follow-up.

### Progression-free survival (PFS)

The duration from the date of registration to the date of confirmation of progression or the date of death from any cause, whichever is the earlier.

- “Progression” includes both PD (progression of disease) based on imaging findings defined in “11.1.9. Overall response” and progression of the primary disease not confirmed by imaging examinations (clinical progression). If the judgment of progression is made based on imaging findings, the date of the examination is regarded as the date of progression, and in the case of clinical progression, the date of clinical judgment is regarded as the date of progression. If the condition is judged to be PD according to the efficacy evaluation criteria as in the case of marked decrease in tumor diameter but is clinically judged to be “clearly not progression”, it is judged to be progression by giving priority to PD based on the efficacy evaluation criteria (in such a case, whether protocol treatment should be continued is determined primarily by the clinical judgment). Also, even if the condition is not PD according to the efficacy evaluation criteria but is judged clinically to be clear progression, it is judged to be progression by giving priority to the clinical judgment.
- Survivors in whom the condition is not judged to be progression are censored on the last day of the absence of clinical progression (last day of confirmation of progression-free survival) (confirmation of the absence of progression by imaging examination or laboratory testing is not required, and clinical confirmation of no progression by means, such as outpatient examination, suffices. Reporting by telephone alone is not accepted. If information about progression or no progression is obtained at a medical institution to which the patient has been transferred or referred, the diagnostic information form indicating the grounds for the diagnosis is received and filed. In this case, also, reporting by telephone alone is not accepted.)
- If another treatment is performed as a second-line treatment in patients in whom chemotherapy has been terminated due to reasons, such as toxicity and rejection by the patient, events and censoring are handled similarly, i.e., the patients are not censored at the time of termination of treatment or the date of initiation of second-line treatment.
- If progression is diagnosed by imaging examinations, the “date of examination” of the imaging examination on which the “definitive diagnosis” is obtained later rather than the date of examination that indicated “suspicion by imaging examination” is regarded as an event. If progression is judged clinically without imaging examinations, the date of the judgment of progression is regarded as an event.
- If the definitive diagnosis of recurrence or new lesion has been made pathologically by biopsy, the date of clinical diagnosis is regarded as an event when the recurrence or new lesion can be diagnosed clinically, and the date of biopsy is regarded as an event when the recurrence is diagnosed pathologically by biopsy rather than clinically.
- In the case of the occurrence of secondary cancer (heterochronic double cancer), it is not regarded as an event, or the patient is not censored, and the duration until observation of another event is regarded as the progression-free survival period.

### Response proportion (Response rate)

The percentage of patients in whom “11.1.10 Best overall response” within 52 weeks after the initiation of protocol treatment is CR or PR among the eligible patients with measurable lesions is regarded as the response proportion. The eligible patients without a measurable lesion are not included in either the numerator or denominator.

### Proportion of patients with adverse events (adverse reactions)

Using all treated patients as denominators, the frequency of the worst grade during all courses (by arm) by CTCAE v4.0-JCOG for each of the following adverse events (toxicities) that have occurred within 52 weeks after the initiation of protocol treatment is calculated:

Laboratory tests: leukopenia, neutrophil count decreased, platelet count decreased, weight loss, blood bilirubin increased, aspartate aminotransferase increased (AST or GOT), alanine aminotransferase increased (ALT or GPT), hyponatremia, hypokalemia, hyperkalemia, creatinine increased

General disorders and administration site conditions: fever, fatigue

Skin and subcutaneous tissue disorders: pruritus, acneiform rash, dry skin, paronychia, alopecia

Gastrointestinal disorders: diarrhea, constipation, nausea, vomiting, oral mucositis

Metabolism and nutrition disorders: anorexia, hypoalbuminemia

Infections and infestations: bronchial infection, pulmonary infection, upper respiratory tract infection

Blood and Lymphoid Disorders: anemia, febrile neutropenia

Nervous system disorders: peripheral sensory neuropathy

Respiratory, thoracic and mediastinal disorders: pneumonitis

<Item added in ver. 2.0>

(only in patients registered in and after ver. 2.0 and administered osimertinib)

Thromboembolism, prolonged corrected QT interval of ECG

In the other adverse event (toxicity) than the above, the proportion of occurrence are not calculated unless a large number of specific adverse events are observed, since only Grade 3 or higher non-hematological toxicity* is reported in the Treatment Form.

* Non-hematological toxicity refers to adverse events other than those listed below in CTCAE v4.0-JCOG.

anemia, bone marrow hypocellular, lymphocyte count decreased, neutrophil count decreased, white blood cell decreased, platelet count decreased, CD4 lymphocyte decreased.

### Proportion of patients with serious adverse events (adverse reactions)

#### 1) Proportion of Grade 4 non-hematological toxicity

The proportion of patients in whom at least one Grade 4 non-hematological toxicity* that has occurred within 52 weeks after the initiation of protocol treatment and is judged to be related (definite, probable, or possible) to protocol treatment among the adverse events mentioned in the free-text field of CRF in addition to the items mentioned in 11.3.4. among all treated patients.

* Non-hematological toxicity refers to adverse events other than those listed below in CTCAE v4.0-JCOG.

anemia, bone marrow hypocellular, lymphocyte count decreased, neutrophil count decreased, white blood cell decreased, platelet count decreased, CD4 lymphocyte decreased.

#### 2) Proportion of early death

Proportion of all deaths during the protocol treatment or within 30 days from the last day of protocol treatment among all treated patients irrespective of the relationship of the cause of death with the protocol treatment.

#### 3) Proportion of treatment-related death (TRD incidence)

Proportion of all deaths judged as causally related (definite, probable, or possible) to the protocol treatment among all treated patients

### Proportion of patients with EGFR exon 20 T790M point mutation in the tumor during progression (only in patients registered before and in ver. 1.1 and administered gefitinib)

Proportion of those with EGFR exon 20 T790M point mutation among those who underwent re-biopsy during progression in a prescribed period in all treated patients. The prescribed period means the period from progression to the initiation of the next chemotherapy or, if gefitinib administration is continued after the judgment of progression, the period until the initiation of chemotherapy other than gefitinib.

<Item added in ver. 2.0>

With the change of standard first-line treatment to osimertinib, to which EGFR with T790M mutation also responds, the significance of checking for T790M mutation during progression is lost. Therefore, the proportion of patients positive for EGFR exon 20 T790M-resisant point mutation in the tumor during progression, which was one of the secondary endpoints, is calculated only in patients administered gefitinib and not in patients administered osimertinib.

# Statistical Considerations

## Main analysis and evaluation criteria

In this trial, main analysis is the final analysis.

The primary objective of analysis in this study is to examine whether the overall survival period, which is the primary endpoint, is better in Arm B that receives study treatment (8-week gefitinib administration, followed by 3 courses of cisplatin/pemetrexed combination therapy, and, then again, by gefitinib administration) than in Arm A that receives the standard treatment (gefitinib monotherapy).

If the results in the study treatment group is significantly better than those in the standard treatment group, the study treatment (8-week gefitinib administration, followed by 3 courses of cisplatin/pemetrexed combination therapy, and, then again, by gefitinib administration) is concluded to be more useful. If the results are not significantly better, gefitinib monotherapy is concluded to remain a useful treatment.

In this study, one-tailed test is performed, because, if the study treatment is inferior to the standard treatment, whether the difference is statistically significant is not a matter of interest (the conclusion that “gefitinib monotherapy, which is the standard treatment, remains a useful treatment” is not affected by whether the difference is significant). The level of significance of the entire study is set at 5% one-tailed. The level of significance used for validation of the primary hypothesis and corresponding confidence coefficient are those based on adjustment for multiplicity associated with interim analysis. For objectives other than validation of the primary hypothesis, the 95% confidence interval is calculated for summarization.

The testing of the nil hypothesis in primary analysis that the overall survival period is equal in the two groups is performed in all registered patients by stratified log-rank test with stratification by allocation adjustment factors other than the institution (surgical history [stage IIIB-IV vs. postoperative recurrence], sex [male vs. female], EGFR mutation [exon19 partial deletion vs. exon21 L858R point mutation]). However, if stratified log-rank test is not expected to be performed appropriately using 3 factors, e.g., when the number of subjects/events in each stratum is small, the handling of allocation adjustment factors is determined in the statistical plan prepared without information concerning comparison among groups before confirmatory analysis involving comparison among groups. Also, analysis in all eligible cases is also performed as sensitivity analysis.

Regarding the overall survival period, which is the primary endpoint, the cumulative survival curve, median survival time, and annual survival rate are estimated using the Kaplan-Meier method, the confidence interval of the median survival time is calculated using the Brookmeyer and Crowley method, and the confidence interval of the annual survival rate is calculated using the Greenwood formula. As the estimated value of the therapeutic effect, the hazard ratio of the therapeutic effect between the groups and its confidence interval are calculated by the stratified Cox proportional hazards model using the same factors as the primary analysis. If necessary, Cox regression is performed with adjustment for biased background factors in addition to the allocation adjustment factors.

The results of the primary analysis are compiled by JCOG Data Center as “Report of Primary Analysis” and submitted to JCOG/WJOG Study Coordinators, JCOG/WJOG Principal Investigators, JCOG/WJOG Group Chairs, JCOG Study Coordinators, JCOG Data and Safety Monitoring Committee, WJOG (permanent) board of directors, JCOG Study Chair, Chairperson of WJOG board of directors with approval by JCOG/WJOG Directors of Data Center.

<Item added in ver. 2.0>

Since the base drug is changed to osimertinib, the drug [gefitinib, osimertinib] is added to the stratification factors in the stratified log-rank test of the primary analysis.

## Target number of registrations, registration period, follow-up period

If a superiority trial design is adopted to examine whether the 3-year survival rate in Arm B is 10% higher than that in Arm A, which is assumed to be 45% (HR=0.749) based on the background shown in “2.4.2. Clinical hypothesis and rationale for the number of patients to be registered”, the number of subjects necessary for analysis calculated using the Schoenfeld & Richter method^73)^ by setting the registration period at 2.5 years, follow-up period at 3 years, α at 5% (one-tailed), and detection power at 80% is 241 in 1 group and 482 with the two groups combined (necessary number of events: 297). If the 3-year survival rate in the standard treatment arm deviates from the assumption, the number of subjects necessary for analysis (necessary number of events) changes to those shown in Table 12.2.1 below.

Table 12.2.1. Numbers of subjects necessary for analysis corresponding to the 3-year survival rates of the two groups and power of test

|  | Power of test | | |
| --- | --- | --- | --- |
| 3-year survival rate | 75% | 80% | 85% |
| 35% vs. 45.6% | 362 (258) | 416 (296) | 482 (345) |
| 45% vs. 55% | 418 (258) | 482 (297) | 560 (345) |
| 55% vs. 63.9% | 502 (259) | 578 (298) | 672 (346) |

* Necessary numbers of events are shown in ( ).

Based on these assumptions, the following setup was adopted in expectation of a few losses to follow up.

Target number of patients to be registered: 250 in each group, 500 with the two groups combined

Registration period: 3 years, follow-up period: 3 years after the end of registration

If there is a large deviation from the assumption, e.g., the outcome is found by periodic monitoring to be clearly better than the assumption, resetting of the sample size is considered. In this event, clinically significant differences are also reevaluated, and re-designing of the trial is made blindly before implementation of analysis.

<Item added in ver. 2.0>

Since the base drug is changed to osimertinib, the setup was modified as follows.

Target number of patients to be registered: 250 in each group, 500 with the two groups combined

Expected registration period: 5 years, follow-up period: 2 years after the end of registration (primary analysis is scheduled in February 2022)

The target number of patient registrations, registration period, and follow-up period of each drug cohort (gefitinib cohort, osimertinib cohort) are shown below. Since the numbers are based on tentative calculations on the assumptions that 10 cases are registered per month and that registration of the osimertinib cohort begins in October 2018, they change if the assumptions prove to be incorrect. In considerations of situations in which minor changes and revisions cause arrest of registration, the registration period is set at 5 years (calculated to be 4.3 years), and the follow-up period at 2 years (calculated to be 1.9 years) after the end of registration.

[Gefitinib cohort]

Target number of registrations: 163 in each arm, 326 with the two arms combined

Registration period: 2.8 years, follow-up period: 3.3 years after the end of registration (if the primary analysis is performed in February 2022)

[Osimertinib cohort]

Target number of registrations: 87 in each group, 174 with the two arms combined

Registration period: 1.5 years, follow-up period: 1.9 years after the end of registration (if the primary analysis is performed in February 2022)

The expected numbers of events if registration of the osimertinib cohort is started in October 2018 are shown in 2.4.2. As supplemental data, the number of events in each drug cohort at each time of analysis was calculated (Table 12.2.2 below). Since the primary analysis of this study is scheduled in February 2022, and the necessary number of events is revised downwardly from the initial 297 to 257 (215 in the gefitinib cohort, 42 in the osimertinib cohort).

Table 12.2.2. Numbers of events in each drug cohort (level of significance in one-tailed tests 5%: no adjustment for the multiplicity of test is made)

| Number of events (power) | Time of analysis | | |
| --- | --- | --- | --- |
|  | Feb. 2022 | Feb. 2023 | Feb. 2024 |
| All registered patients (n=500) | 257 (74.9%) | 294 (79.8%) | 324 (83.1%) |
| Gefitinib cohort (n=326) | 215 (68.1%) | 238 (72.1%) | 256 (74.8%) |
| Osimertinib cohort (n=174) | 42 (23.9%) | 56 (28.7%) | 68 (32.5%) |

Table 12.2.2 indicates that sufficient power of test cannot be maintained in the osimertinib cohort alone. In this study, the results of analysis/evaluation criteria for all registered patients are also applied to the osimertinib cohort if the hazard ratios estimated separately in all registered patients and the osimertinib cohort are consistent. If the number of events in all registered patients is assumed to be 257, and the hazard ratio observed in each drug cohort is assumed to be 0.749 (i.e., the hazard ratio in all registered patients is also 0.749), the minimum number of events in the osimertinib cohort necessary to make the probability that the estimated hazard ratios are consistent between the osimertinib cohort and all registered patients (calculated by Method 1 of Question 6 in the MHLW guideline “Basic concept concerning international collaborative trials”^89)^ with π substituted for 0.5. This is equivalent to the judgment that there is consistency if a therapeutic effect at least half that in the entire trial is observed in the subgroup.) ≥70% is calculated to be 36.^90)^ In the primary analysis scheduled to be performed in February 2022, the number of events reaches this value in the osimertinib cohort alone.

As mentioned in 2.4.2, if interaction is observed between the drug (gefitinib/osimertinib) and treatment (Arm A/Arm B), evaluation of the superiority of Arm B to Arm A in each drug cohort is inevitable, but qualitative interaction is unlikely to be caused by a small number of subjects. As shown in Table 12.2.2, 42 events are expected to be collected in the osimertinib cohort in the primary analysis scheduled to be implemented in February 2022, and, if the hazard ratio observed in each drug cohort is assumed to be 0.749 (i.e., the hazard ratio in all registered patients is also 0.749), the probability that the test of the primary hypothesis in all registered patients becomes statistically significant at the time of primary analysis and that the hazard ratio in the osimertinib cohort surpasses 1 is calculated to be 9.3% (the same probability calculated in the gefitinib cohort is 0.0%). No clear evaluation criteria are set for the occurrence of quantitative interaction, and a conclusion is made in consideration of the standard treatment system and the status of development at the time of analysis.

## Interim analysis and early termination of trial

### Objective and time of interim analysis

Two interim analyses are performed during the study to evaluate whether the primary objective of this study has been achieved. The first interim analysis is performed during the registration period to evaluate whether the continuation of registration is appropriate, and the second interim analysis is performed approximately 1 year after the end of registration to evaluate whether the follow-up should be continued for the scheduled period. In both analyses, the study is terminated if the primary objective of the study is judged to have been achieved, and the results are promptly made public as a conference presentation and a paper.

The first interim analysis is conducted using the data of the first periodic monitoring, in which inquiries are made after about 200 registrations have been obtained (about 1 year after the beginning of registration if it progresses as planned). When the prospect of the time of attaining about 200 registrations becomes clear, JCOG Data Center notifies it to JCOG/WJOG Study Coordinators. JCOG Data Center and WJOG Data Center make preparations for the interim analysis in cooperation with JCOG/WJOG Study Coordinators by prompting the submission of record forms and making inquiries about unclear entries in record forms and check the possibility of combining data prior to the interim analysis.

The second interim analysis is performed simultaneously with periodic monitoring at an appropriate timing about 1 year after the end of registration by consultation among JCOG Data Center, WJOG Data Center, and JCOG/WJOG Study Coordinators.

Registration is not interrupted during the first interim analysis, in principle.

If the study progresses as scheduled, the numbers of events at the times of the first and second interim analyses performed under the conditions shown in 12.2 are expected to be 21-46 and 198 on the assumption that they are performed 1-1.5 years after the beginning of registration and 1 year after the end of registration, respectively.

### Methods for interim analyses

Interim analyses are performed by JCOG Data Center.

To maintain the α error of the entire study at 5% on one-tailed tests, multiplicity of the tests in the interim and final analyses is adjusted using the Lan & DeMets α-spending function, and the statistical significance of the difference in the survival time between the arms is examined. The O’Brien & Fleming spending function is used as the α-spending function.

Regarding details of interim analyses, the statistical staff of the JCOG Data Center Lung Cancer Group prepares the analysis plan by the time of interim analysis. The actual interim analysis is performed, and the interim analysis report is prepared, by statistical staff members not assigned to the Lung Cancer Group.

If the survival time in Arm B is superior to that in Arm A, and the p value of stratified log-rank test is below the level specified by the above method, the difference is judged to be statistically significant, and the trial is terminated, in principle. If the survival curve of Arm B is below that of Arm A, the appropriateness of termination of the trial is evaluated comprehensively rather than by statistical tests.

The following values are calculated as information useful for the judgment of whether the trial should be terminated as ineffective.

- Predictive probability concerning the primary endpoint (probability calculated based on estimation of the distribution of hazard ratios obtained at the end of the study by the method of Spiegelhalter et al.^75)^)
  - Predictive probability that statistically significant superiority of Arm B to Arm A is demonstrated concerning the primary endpoint at the time of final analysis
  - Predictive probability that the point estimate of the hazard ratio in Arm B relative to Arm A is greater than 1.0 at the time of final analysis
- Conditioned power concerning the primary endpoint (power of test calculated by the method of Halperin et al.^76)^)
  - Conditioned power calculated by giving the results of interim analysis on the assumption that the hazard ratio between Arm B and Arm A remains consistent with the nil hypothesis (HR=1.0) after interim analysis
  - Conditioned power calculated by giving the results of interim analysis on the assumption that the hazard ratio between Arm B and Arm A remains the same as that at the planning of the trial (HR=0.749) after interim analysis

### Reporting and review of the results of interim analyses

The results of interim analyses are compiled as the interim analysis report, submitted by JCOG Data Center to JCOG Data and Safety Monitoring Committee, and reviewed concerning the appropriateness of continuation of the trial and publication of the results. JCOG Data and Safety Monitoring Committee evaluates the appropriateness of continuation of the trial by a conference and, according to the results of the review, recommends whether the trial should be continued and whether the results should be publicized to JCOG/WJOG Principal Investigators or JCOG/WJOG Group Chairs.

However, of the JCOG Data and Safety Monitoring Committee members, those belonging to the Lung cancer group do not participate in the review. Also, JCOG/WJOG Principal Investigators, JCOG/WJOG Study Coordinators, researchers of the participating institutions, JCOG/WJOG Group Chairs, and JCOG Lung cancer group Coordinator of this study are not informed of the results of interim analyses of this study until the end of the final follow-up unless Data and Safety Monitoring Committee recommends the termination of this study based on the results of interim analyses.

If JCOG Data and Safety Monitoring Committee recommends the termination or modification of all or part of the trial based on the review of the interim analysis report, JCOG/WJOG Principal Investigators and JCOG/WJOG Group Chairs evaluate the contents of the recommendation and make a decision about the termination or partial modification of the trial.

In the event of termination or partial modification of the trial, JCOG/WJOG Principal Investigators and JCOG /WJOG Group Chairs submit a written “request for study termination” or “request for protocol revision” to JCOG Data and Safety Monitoring Committee in their joint names. JCOG/WJOG Principal Investigators can terminate or partially modify the study with approval by JCOG Data and Safety Monitoring Committee.

JCOG/WJOG Principal Investigators and JCOG/WJOG Group Chairs may file an objection concerning the contents of the recommendation of JCOG Data and Safety Monitoring Committee but must eventually abide by the order of JCOG Study Chair if they fail to reach an agreement with JCOG Data and Safety Monitoring Committee.

If the study is terminated, the subsequent follow-up period is 3 years from the last registration.

If the study is terminated according to the results of an interim analysis, the interim analysis is regarded as the primary analysis of the study. JCOG Data Center supplements incomplete data and performs analyses necessary for publication of the results primarily of that interim analysis in cooperation with JCOG/WJOG Principal Investigators and JCOG/WJOG Study Coordinators, promptly prepares the “primary analysis report”, and submits it to JCOG Lung Cancer Group, WJOG Respiratory Organ Group, JCOG Data and Safety Monitoring Committee, WJOG board of directors, JCOG Study Chair, and Chairperson of WJOG board of directors with approval by JCOG/WJOG Directors of Data Center.

## Analysis of secondary endpoints

Secondary endpoints are analyzed for evaluation to supplement the results of primary analysis of the study. Since analysis of secondary endpoints is exploratory, adjustment for multiplicity is not performed. Comparison between arms is performed, if necessary, but it should be noted that the results of inter-arm comparison not being significant does not mean that there is no difference between the arms.

### Analysis of secondary endpoints concerning the safety

Of the secondary endpoints, the proportion of patients with adverse events and proportion of patients with serious adverse events are related to the safety, and they are items of periodic monitoring, in principle (“14.1. Periodic monitoring”).

It is hoped that the proportion of patients with adverse events and the proportion of patients with serious adverse events are not markedly higher in the trial treatment arm than in the standard treatment arm. Concerning the proportion of patients with adverse events, the frequencies of the occurrence of individual adverse events are tallied up, and the proportion of Grade 3 or severer adverse events is calculated. Concerning adverse events other than laboratory test results, the proportion of Grade 2 or severer adverse events is also calculated. Concerning Grade 4 non-hematological toxicities, early death, and treatment-related death, which are serious adverse events, the registration numbers and details of the conditions are reported in the periodic monitoring report. Also, the proportions of patients with Grade 4 non-hematological toxicities, early death, and treatment-related death are calculated at the time of interim analyses, primary analysis, and final analysis. The interval estimation of the proportions is performed using accurate confidence intervals based on binomial distribution. None of these endpoints are evaluated based on statistical testing, but inter-arm comparisons are performed as necessary by tests using Fisher’s exact test.

### Analysis of secondary endpoints concerning the efficacy

Of the secondary endpoints, the response rate, progression-free survival time, and proportion of patients with EGFR exon20 T790M point mutation in the tumor during progression are related to the efficacy, but the response rate and progression-free survival time are analyzed only in interim analyses and final analysis. However, as interpretation of the response rate is not considered possible at the times of interim analyses, tallying is made to check whether data have been collected appropriately, and the results are not presented in interim analysis reports. The proportion of patients with EGFR exon20 T790M point mutation in the tumor during progression is analyzed only in the final analysis.

Since progression-free survival is defined as a surrogate endpoint for overall survival, it is hoped that it is longer in the trial treatment arm than in the standard treatment arm. It is hoped that the response rate is not lower, and the proportion of patients with EGFR exon20 T790M point mutation in the tumor during progression is lower, in the trial treatment arm than in the standard treatment arm. The progression-free survival and response rate are analyzed in all registered patients, but comparisons in all eligible patients after exclusion of ineligible cases determined by evaluation in the group are also performed as sensitivity analysis.

Variables, such as the progression-free survival curve, median progression-free survival time, and annual progression-free survival rate, are estimated using the Kaplan-Meier method, the confidence interval of the median progression-free survival rate is calculated using the Brookmeyer and Crowley method, and the confidence interval of the annual progression-free survival rate is calculated using the Greenwood formula. Log-rank test is used for inter-arm comparisons. As estimates of the therapeutic effect, the hazard ratio of the therapeutic effect between the arms and its confidence interval are calculated using the Cox proportional hazards model. If necessary, Cox regression analysis is performed with adjustment for biased background factors in addition to the allocation adjustment factors.

Inter-arm comparisons of the response rate and proportion of patients with EGFR exon20 T790M point mutation in the tumor during progression are made using Fisher’s exact test, and interval estimation is made using accurate confidence intervals based on binomial distribution.

## Final analysis

After the end of the follow-up period, all endpoints are analyzed after fixation of data by the final review. In this study, primary analysis is the final analysis (See 12.1).

At other times, inter-arm comparison of the primary endpoint or inter-arm comparison of the secondary endpoints concerning the efficacy is not performed except when they are mentioned in the protocol or permission by JCOG Data and Safety Monitoring Committee is obtained.

The results of the final analysis are compiled by JCOG Data Center as “Report of final analysis (report of primary analysis)” and submitted with approval by JCOG/WJOG Directors of Data Center to JCOG/WJOG Study Coordinators, JCOG/WJOG Principal Investigators, JCOG/WJOG Group Chairs, JCOG Lung Cancer Group Coordinator, JCOG Data and Safety Monitoring Committee, WJOG board of directors, JCOG Study Chair, and Chairperson of WJOG board of directors (contents of 12.1 presented again).

The JCOG/WJOG Principal Investigators/Study Coordinators review the contents of Report of final analysis, prepare “Review Report” summarizing the conclusion of the entire study, problems, interpretation and discussion of the results, and future policy primarily from clinical viewpoints, and, with approval by JCOG/WJOG Group Chairs and JCOG/WJOG Directors of Data Center, submit it to JCOG Data and Safety Monitoring Committee, WJOG board of directors, JCOG Study Chair, and Chairperson of WJOG board of directors.

In JCOG, approval of Review Report by Data and Safety Monitoring Committee is considered to mark the “end of study administration”. In WJOG, approval by the board of directors is considered to mark the “end of study administration”.

## Exploratory analyses

To evaluate interaction between the therapeutic effect and subsets, exploratory subgroup analyses are performed concerning the following factors. Since these analyses are not performed by securing sufficient power of test or with adjustment for multiplicity, the results of each subgroup analysis is interpreted only as exploratory results.

Factors expected to be used for subgroup analyses

- Surgical history (stage IIIB-IV/postoperative recurrence)
- Sex (male/female)
- EGFR mutations (exon 19 partial deletion/exon 21 L858R point mutation)
- PS (0/1)
- Histological type (adenocarcinoma/others)
- Smoking history (Yes/No)
- Age (≤64 years/≥65 years)

<Item added in ver. 2.0>

Since the base drug is changed to osimertinib, subgroup analysis concerning “drug (gefitinib/osimertinib)” is also performed.

## Early termination of the study

In this study, early termination of the study may occur in the following cases:

1) Early termination by interim analysis

2) Early termination due to adverse events

3) Early termination due to poor accrual

4) Early termination due to other reasons

### Early termination by interim analysis

In this study, based on the criteria described in "12.3. Interim Analysis," recommendations for early termination of the study may be issued at the interim analysis review by the Data and Safety Monitoring Committee. If the Data and Safety Monitoring Committee provides recommendations for early termination of the study, JCOG/WJOG Principal Investigators and JCOG/WJOG Group Chairs will review the recommendations and decide whether to terminate the study early.

### Early termination due to adverse events

It is hoped that the frequencies of pneumonitis and treatment-related death in each treatment arm of this study do not exceed 6% and 3%, respectively, on the basis of the following observations.

- Generally, the permissible range of frequency of treatment-related death in chemotherapy for advanced non-small cell lung cancer is considered 3-5%.
- Results of phase III clinical trials of gefitinib monotherapy vs. platinum-based combination chemotherapy conducted in Japan

WJTOG3405 study: Of the 87 patients in the gefitinib arm, pneumonitis (all grades) was observed in 2 (2.3%), and treatment-related death was observed in 1 (1.1%)

NEJ002 study: Of the 114 patients in the gefitinib arm, pneumonitis (all grades) was observed in 6 (5.3%), and treatment-related death was observed in 1 (0.9%).

- In phase III clinical trials of CDDP+PEM vs. CDDP+GEM performed in Western countries, treatment-related death was observed in 9 (1.0%) of the 837 patients in the CDDP+PEM arm.

If pneumonitis is observed in 15 patients, or treatment-related death is observed in 8 patients, in each treatment arm during the study, registration should be suspended immediately, and Data and Safety Monitoring Committee should be consulted about whether the study should be terminated, because it is clear that the point estimate of the incidences of pneumonitis and treatment-related death will be ≥6% and ≥3%, respectively. While the numbers of patients with pneumonitis and those of treatment-related deaths remain ≤14 and ≤7, respectively, in each treatment arm, each patient is reported to Data and Safety Monitoring Committee for judgment about the handling, but registration is continued until the result of the review is obtained, in principle.

<Item added in ver. 2.0>

In FLAURA study, pneumonia due to osimertinib and treatment-related death were observed in 4% and 2% of the patients, respectively. Therefore, if pneumonitis is observed in 4 patients, or treatment-related death is observed in 2 patients in each treatment arm of the osimertinib cohort, registration is suspended immediately, and Data and Safety Monitoring Committee should be consulted about whether the study should be terminated.

In all patients including the gefitinib cohort, registration is suspended immediately, and Data and Safety Monitoring Committee should be consulted about whether the study should be terminated if pneumonitis is observed in 15 patients, or treatment-related death is observed in 8 patients, in each treatment arm, as has been practiced.

<Item added in ver. 2.2.0>

According to the results of subgroup analysis in Japanese participants of FLAURA study, pneumonitis was observed in 8 (12.3%) of the 65 patients treated with osimertinib, but the condition was mild (Grade 1-2) in 7 (10.8%), severe (≥Grade 3) in only 1 (1.5%), and Grade 5 in none. However, according to the final report of the post-marketing surveillance of TAGRISSO® tablets (osimertinib) using patients previously treated with first/second generation EGFR-TKI and positive for EGFR T790M as primary participants, 245 (6.8%) of the 3,578 patients analyzed developed pneumonitis, which was Grade 1-2 in 141 (3.9%), ≥Grade 3 in 104 (2.9%), and Grade 5 in 29 (0.8%). From these results, osimertinib is considered to more frequently cause pneumonitis but less frequently cause severe pneumonitis or death than first/second generation EGFR-TKI including gefitinib. Thus, considering it appropriate to establish criteria for early termination of the study due to pneumonitis for each cohort rather than for all cohorts, we reset them.

Since the incidence of severe pneumonitis in the gefitinib cohort was 2.7% in NEJ002 trial, it is decided in this study to immediately suspend registration and consult Data and Safety Monitoring Committee about the appropriateness of study termination if Grade 3 or severer pneumonitis is observed in 4 of 151 patients in each treatment arm.

In the osimertinib cohort (planned to consist of 192 patients with the two arms combined), it is decided to immediately suspend registration and consult Data and Safety Monitoring Committee about the appropriateness of study termination if 4 patients with Grade 3 or severer pneumonitis or 2 patients with treatment-related death are observed in each treatment arm.

Regarding treatment-related deaths in the entire study, it is decided to immediately suspend registration and consult Data and Safety Monitoring Committee about whether the study should be terminated if 8 treatment-related deaths are observed in each treatment arm as has been practiced.

### Early termination due to poor accrual

If the patient accrual pace is significantly worse than that at the planning, early termination of the study may be recommended by the Data and Safety Monitoring Committee. If early termination recommendations are issued by the Data and Safety Monitoring Committee due to poor accrual, the JCOG/WJOG Principal Investigators and the JCOG/WJOG Group Chairs will review the recommendations and decide whether to terminate the study early.

### Early termination due to other reasons

If it is judged difficult to continue the study for other reasons for 12.7.1.–12.7.3., the JCOG/WJOG Principal Investigators will submit the “Request for Early Termination of the Study” to Data and Safety Monitoring Committee. If the Data and Safety Monitoring Committee recommends early termination of the study based on the submitted document, the procedure for early termination of the study will be progressed.

## Procedures after early termination of the Study

If the JCOG/WJOG Principal Investigators accepts the recommendations made by the Data and Safety Monitoring Committee based on Section 12.7, he/she will promptly submit a notification to the Data and Safety Monitoring Committee that early termination of the study will be performed.

The JCOG/WJOG Principal Investigators promptly informs the Site Investigator of the decision to terminate the study early in writing, and the Site Investigator who has received a report of early termination of the study will report in writing that the study was prematurely terminated to the administrator of the institution without delay.

If the study is terminated early, JCOG Data Center will promptly initiate the development of the primary analysis report or final analysis report. Subsequent follow-up will be 3 years from last enrollment. However, in case of early termination due to poor accrual, subsequent follow-up is omitted, in principle.

# Ethical Considerations

## Human subject protection

All researchers involved in this study will conduct this trial in accordance with "Helsinki Declaration" ^2)^ of the (^1^) and "Ethical Guidelines for Medical and Health Research Involving Human Subjects" (No.1 of Notice of the Ministry of Education, Culture, Sports, Science and Technology and the Ministry of Health, Labour and Welfare, 2017).

1) http://dl.med.or.jp/dl-med/wma/helsinki2013j.pdf

2) http://www.mhlw.go.jp/stf/seisakunitsuite/bunya/hokabunya/kenkyujigyou/i-kenkyu/index.html

The "institution" in this protocol correspond to the "research institutions and collaborating institutions" in the above guidelines. In addition, the term "institutional approval" in this protocol refers to the availability of approval documents issued by the Administrator of the participating institution based on the results reviewed by the Institutional Review Board (IRB)*.

※ Regardless of whether or not the IRB is established by the institution itself.

## Informed consent

### Explanation to the patient

Prior to patient registration, the investigator or subinvestigator will provide the patient with written informed consent form approved by Certified Review Board and explain the following details verbally.

#### Descriptions

1. Disease names, Stages, and expected prognosis (Helsinki 6) (Regulation 46 1②, 2).
2. That this study is a clinical trial and is conducted by JCOG and WJOG (Helsinki 5, 6, 10, 12, 21, 22, 31) (rules 46 1①, ②, 17)
   Name of Certified Review Board and contact information for receiving complaints and inquiries to the committee
3. Design and rationale of the study (Helsinki 22, 31, 33) (Regulation 46 3, 4①:
4. Protocol treatment content (Helsinki 16, 18, 22, 33) (Regulation 46 1③, 4)
5. Effects expected by protocol treatment (Helsinki 16, 17, 18) (Regulation 46 4)
6. Expected adverse events, complications, and sequelae and how to deal with them (Helsinki 15, 16, 17, and 18) (Regulation 46 4 and 14).
   Explanation of the extent and frequency of expected adverse events, including complications, sequelae, and treatment-related deaths, and how to deal with them when they occur. In addition to these explanations, obtain the most recent version of the drug package insert and deliver it to patients (PMDA Prescription Pharmaceutical Information Search http ://www.pmda.go. jp/PmdaSearch/iyakuSearch/)
7. Post-study treatment after end of protocol treatment should also be performed appropriately (Helsinki 18, 22, 34) (Regulation 46 18①②)
8. Cost burden and compensations (Helsinki 15, 22) (Regulation 46 14 16①②)
   Explanation of the cost of treatment, compensation that can be received in the event of a health hazard (equivalent to measures taken in general practice, etc.)
9. Alternative treatment (Helsinki 37) (Regulation 46 15)
   Explanation of treatments that can be received if not participating in this study
10. Anticipated benefits and possible disadvantages (Helsinki 11, 16, 17, 18) (Rule 46 4②③)
    Explanation of anticipated benefits and possible disadvantages by participating in this study
11. Direct access to the medical records (Helsinki 23) (Regulation 46 18④:
    Explanations on acceptance of site visit audits, such as "direct access to medical records etc. by healthcare professionals at other medical institutions for quality control with permission from the administrator of the participating institution."
12. Refusal of consent and withdrawal of consent (Helsinki 8, 9, 10, 14, 25, 26, 27, 28, 29) (Rule 46 5, 6, 7)
    Refusal to consent prior to participation in the study is free, and withdrawal after having given consent is free, thereby not causing undue medical disadvantage.
13. Protecting human rights (Helsinki 7, 9, 24) (Rule 46 8③ 10)
    Every effort should be made to ensure that personal information, such as names, is kept confidential.
14. Conflicts of Interest (Helsinki 22, 23, 36) (Rule 46 12, 18③④)
15. Secondary use of data (Helsinki 34) (Regulation 46 8)
    The possibility of secondary use of data obtained from this study in Japan and overseas (ancillary studies, meta-analyses, etc.) only when approved by either committee in JCOG
16. Method of disclosure of information on the study (Rule 46 8)
    The study is registered and published in jRCT^※^. In addition, the results of clinical studies should also be published in jRCT (∗ Databases (Japan Registry of Clinical Trials) https://jrct.niph.go.jp/ prepared by the MHLW as stipulated in Paragraph 1 in Article 24 of Clinical Trials Act Enforcement Regulations)
17. Freedom of questions (Helsinki 8, 9, 24) (Regulation 46 1, 2, 9, 13, 18⑤)

Explanation that investigators, written contact information for consultations on study details, on the Principal Investigator and the Study Coordinator, and freely asking questions about study and treatment

1. Sample collection for ancillary studies (including translational researchs and biobanking) (Helsinki 32) (Regulation 46 11).

### Consent

Explain the study, give sufficient time to think, confirm that the patient understood the study well, and ask for participation in the study. If the patient agrees to participate in the study, the written informed consent form in the appendix will be used to obtain the patient's own signature. The site investigator or the subinvestigator confirms that the study consent form contains the name of the physician who provided the explanation and the date of explanation, the name of the patient who gave informed consent, and the date of informed consent.

In addition, when it is not possible to read the documents due to visual impairment, etc., but the details can be understood by verbal explanation, or the documents can not be signed due to limb disorders, etc., but the documents can be read and understood, signatures may be obtained from the proxy author under the consent by the patient. However, the signature of the proxy author should be based on this study's consent, and should be described as "Signing by the proxy author" and "Relationship with the patient" so that the person can be found to be the proxy author.

### Response to inquiries, consultations, etc. after consent

In principle, the investigator or subinvestigator of the relevant patient's participating institution responds to any consultation related to the study by the patient or his/her family after registration. If it is unclear how to respond, respond in consultation with Principal Investigator, Study Coordinator, the Group Secretariat, Group Chair, JCOG Data Center/Operations Office, etc. in accordance with the content of the consultation.

### Withdrawal of consent

The procedures for discontinuation of the patient's follow-up request and removal of patient data will be specified separately in the procedural manual, and the completion of each task will be reported to Study Chair and Study Coordinator.

1. Patient refusal: Refusal to continue subsequent protocol treatment (follow-up continues).
2. Withdrawal of consent: Withdrawal of consent to participate in the study and termination of all subsequent treatment and follow-up in accordance with the study protocol. Research use of data prior to withdrawal of consent is permitted.
3. Full withdrawal of consent: Withdrawal of consent to participate in the study and unavailability of all data from the time of patient registration, including information at registration.

In addition, some medical institutions may request that a "withdrawal of consent" form be prepared as a written document. However, in the event of withdrawal of consent, written expressions of willingness are required to increase the psychological barriers to withdrawal of consent (i.e., it is difficult to withdraw consent), and it is considered unwanted from the viewpoint of protecting human subjects. Therefore, in JCOG, written expressions of willingness are not mandatory for withdrawal of consent, verbal withdrawal of consent is valid, and the "withdrawal of consent" form is not prepared. If "withdrawal of consent" form is required by the participating institution, it should be prepared by the institution.

## Protection of personal Information and patient identification

JCOG recognizes that information on privacy, such as personal information and medical information, should be protected and handled carefully, based on the principle of respecting the personality of individuals, and has formulated JCOG Privacy Policy, and will take all possible measures to protect privacy. For more information, see JCOG website (http://www.JCOG.jp/).

### Policies, legislation, and norms followed by JCOG

In conducting JCOG study, JCOG follows, in principle, JCOG Privacy Policy as well as the following laws and norms depending on the content of the research. If other laws, norms, and policies are applicable, they should be followed.

- Clinical Trials Act (Law No. 16, 2017).
- Act on the Protection of Personal Information (Law No. 57, 2003, Final Amendment: Law No. 65, September 9, 2015).
- Helsinki Declaration (Translation by the Japan Medical Association)
- Ethical Guidelines for Medical and Health Research Involving Human Subjects (No.1 of Notice of the Ministry of Education, Culture, Sports, Science and Technology and the Ministry of Health, Labour and Welfare, 2017).

### Use of personal information objective, items to be used, and methods of use

#### 1) Objective of use

In accordance with the basic philosophy "Providing the best treatment to more patients," JCOG uses personal information, etc. of patients for objective of "Identifying patients and conducting surveys not only during treatment but also for a long period after end of treatment in order to obtain the correct results of clinical studies, and appropriately managing the acquired information."

#### 2) Items to be used

Information to identify individuals who will be used by JCOG as minimally require for identification and inquiry of patients is as follows.

Medical record number, date of birth, initials, and registration number

In other words, information that can identify individuals other than those listed above, such as the patient's name, is not informed to Data Center by participating institutions, and if they are falsely informed, they should be destroyed without using the recording medium or stored after performing appropriate processing, such as masking, which is incapable of reading.

#### 3) Method of use

Personal information of patients used by JCOG will be collected by entering the CRFs etc. by researchers at participating institutions and submitting them to Data Center either by JCOG Web Entry System, mailing, or handover as a rule. However, telephone calls will be used only for patient registration where prompt contact is necessary.

In addition, in order to confirm the accuracy of the collected information, inquiries regarding various types of CRFs, including personal information, between Data Center and researchers at medical institutions are limited to either JCOG Web Entry System, mailing, or handover. Only the more anonymous registration number should be used when interacting with e-mail inquiries, and medical record numbers and initials should not be used.

### Preparation of records for provision of samples and information, etc. at participating institutions

For JCOG studies, the methods used to prepare records related to the provision of samples and data shall be in accordance with the Guidance for Ethical Guidelines for Medical and Health Research Involving Human Subjects (partially revised on March 8, 2017) (5 in 8(1)) (see Tables 13.3.3).

Table 13.3.3. Records and provide chapters related to the provision of samples and information at participating institutions (adapted from the Guidance Edition, p65)

| Matters to be recorded | Providing institution  Participating institutions | Recipient institution  JCOG Data Center | Protocol  Relevant chapter |
| --- | --- | --- | --- |
| Recorded Matters A (must be stated) | | |  |
| ○ Name of the recipient institution | → On behalf of the recipient | Study protocol | Chapter 16 |
| ○ Name of site investigator of recipient institution | → On behalf of the recipient | Study protocol | Chapter 16 |
| ○ Name of providing institution, etc. |  | Study protocol | Chapter 16 |
| ○ Name of site investigator of the providing institution, etc. |  | Study protocol | Chapter 16 |
| ○ Sample and Information Items | → On behalf of the recipient | Study protocol | Chapters 8 and 9,  Chapters 10 and 11 |
| ○ Log of obtaining samples and information |  | Study protocol | Chapter 13 |

### Storage of Samples and Information

Samples and information of patients enrolled in the study should be retained in accordance with the procedures specified by each medical institution. It is recommended that the storage time limit for samples and information on this study at participating institutions and records on the provision of samples and information be maintained for as long as possible after the final analysis report is submitted up to 5 years or the publication date of any article related to this study up to 3 years, whichever comes later. After the storage period, samples and information related to this study should be discarded after being anonymized.

The storage date of the data collected in JCOG Data Center will be semi-permanent in view of the possibility of long-term follow-up and secondary use of data. In addition, as a record of the provision of specimens/information, the protocol/model informed consent form will be retained semi-permanently in JCOG Data Center.

### Anonymization and control of response tables

JCOG studies do not collect information that clearly identifies individuals by itself, such as patient names, and use registration numbers and medical record numbers to identify individuals (anonymized). Correspondence tables (not always in the form of Tables) of information and registration numbers that can clearly identify individuals, such as patient names, are positioned to be present at each participating medical institution and should be appropriately managed according to the policies of participating medical institutions to ensure the identification of enrolled patients.

### Secondary use of data

Data from this study may be used secondarily (e.g., meta-analysis) in the country or abroad only if approved by JCOG or WJOG relevant committees (e.g., Protocol Review Committee). However, when providing data to external organization (e.g., meta-analysis), it is not possible to identify individuals.

Secondary use of data should be made available on JCOG website to ensure that patients are able to refuse use of the data.

### Safety management responsibility system

The privacy protection control manager and privacy protection manager shall be established, and various safety control measures shall be taken to minimize the risk of leakage of information when using personal information.

### Response to disclosure of patient information, etc.

When patients ask to disclose privacy, information held by JCOG and WJOG, the responders shall in principle be the researchers (Site Investigator, Site Coordinator, and physician-in-charge) at the institution of the patient.

### General Inquiries and Complaints Received

General inquiries and complaints regarding privacy policies will be received by either postal, e-mail, or FAX.

Inquiry contact: [JCOG]

JCOG Data Center Privacy Protection Manager

Postal Destinations: 5-1-1, Tsukiji, Chuo-ku, Tokyo 104-0045, Japan

Clinical Research Support Office, National Cancer Center Hospital

E-mail: JCOG_privacy@ml.jcog.jp

FAX: 03-3542-3374

[WJOG]

WJOG Data Center

Postal Destinations:1-5-7-304, Motomachi, Naniwa-ku, Osaka City, Osaka 556-0016, Japan

E-mail: datacenter@wjog.jp

FAX: 06-6633-7405

## Compliance with the protocol

## Researchers participating in this study will comply with this protocol unless it compromises patient safety and human rights.

## Approval of the Ethics Review Committee of the medical institution

### Approval at the initiation of the study

When participating in this study, this protocol and informed consent form must be approved by each medical institution.

When approval is obtained, the site coordinator of each medical institution will send copies of the approval document to each group's data center. Original approval documents will be stored by the site coordinator, and copies will be stored by the data center.

The informed consent form can be used at each medical institution with modifications to the extent that they do not deviate from the requirements for clinical trials with the approval of the medical institution in question, but no changes in the protocol are allowed at each medical institution. A protocol common to all medical institutions will be used. Site coordinator should consult Study Coordinator if they request a modification of the protocol text.

### Annual update of each medical institution's approval

The presence or absence of annual updates to the approval of each medical institution for this protocol and informed consent form shall be in accordance with the regulations of each participating institution. As JCOG and WJOG, it is not required to submit annual renewal approvals of medical institutions.

## Protocol revision/amendment

### Categories of protocol modifications

In the event of a protocol change, the Protocol revision Application must be submitted to JCOG Data and Safety Monitoring Committee and approved prior to the issue of the change (activation). If it is considered necessary to change the protocol in this study, JCOG Study Coordinator /WJOG Study Coordinator will discuss the content of revision, develop a draft revision based on a consensus between the two groups, and request JCOG Data and Safety Monitoring Committee for review. After approval of revision at JCOG Data and Safety Monitoring Committee, approval of revision at WJOG Respiratory Group Meeting will be obtained, and revision will be enacted.

In JCOG and WJOG, changes in the protocol after approval of the Protocol Review Committee will be handled in two different ways: amendment and revision, but JCOG Data and Safety Monitoring Committee Office will distinguish between amendment and revision, so all applications will be submitted as 'revision'. We also distinguish the addition of supplementary explanations that do not fall under a change in protocol content as 'memorandum'. Definitions and handling are as follows.

1) Amendment

Partial protocol changes which meet one or more of the followings: i) Potential to increase the risk of patients enrolled in the study, ii) Having substantial effects on primary endpoint of the study, iii) having essential effects on the study's implementation structure. Data and Safety Monitoring Committee and institutional approvals are required.

The amended version of the protocol and informed consent form version numbers are shown as in 2.0.0, 3.0.0, and 4.0.0….

Approval of both Group Chair and the director of both data centers is required prior to submission to　JCOG Data and Safety Monitoring Committee.

When classified as "amendment" by the Secretary-General of Data and Safety Monitoring Committee, the change will be reviewed by Data and Safety Monitoring Committee. When approved through Data and Safety Monitoring Committee review, the date of approval of Data and Safety Monitoring Committee and the date of entry into force are listed on the protocol cover page. The date of entry into force will be set at 2 months after the date of approval by Data and Safety Monitoring Committee.

In the case of during accrual, patient registration should not be suspended until the entry into force, as a rule, and approval of each participating institution should be obtained for the contents of the amendment. When approval is obtained, the site coordinator at each site will send copies of the approval documents for each institution to the Data Center. If the approval of the institution is obtained before the date of entry into force, the institution shall continue to enroll patients beyond the date of entry into force. On the other hand, if the approval of the institution cannot be obtained by the entry into force date, the institution will be suspended for the registration of the patient at the entry date and resume the registration once a copy of the approval document is received at the Data Center.

Treatment and assessment of enrolled patients will be performed according to the pre-change version protocol until entry into force. Protocol deviations to enhance patient safety during treatment will be permitted if pre-change protocols threaten patient safety, such as inadequate treatment modification criteria. If protocol deviations occur, they should be listed in the monitoring report.

2) Revision

Protocol changes which meet all of the followings: i) does not have an increased risk for patients enrolled in the study; ii) does not have a substantial effect on primary endpoint of the study; iii) does not have an inherent effect on the system in which the study is conducted. Changes will not be reviewed by Data and Safety Monitoring Committee but will need to be approved by each institution. Whether the type of review at each institution is a routine review or an accelerated review is left to the discretion of each institution. In principle, suspension of patient registration is not performed at the case of "revision".

The revised version of the protocol and informed consent form version numbers are shown as in 1.1.0, 1.2.0, and 1.3.0….

Approval by both Group Chair and the director of both Data Center is mandatory prior to submission to the Data and Safety Monitoring Committee.

When classified as "Revision" by the Secretary-General of Data and Safety Monitoring Committee, the Secretary-General of Data and Safety Monitoring Committee will issue a verification form. Also, the date of approval by the Data Center Director and the date of entry are listed on the protocol cover page.

Since the entry into force, the study shall be conducted in accordance with the revised details approved as a rule, even before approval by the institution. Consult with Study Coordinator and the Data Center if revisions cannot be enacted until approved by the institution because of the institution's circumstances. When approval is obtained at each institution, it is not necessary to send copies of the approval documents from each institution to the Data Center, but the original approval documents will be retained by the site coordinator because they will be checked during the site visit audit.

3) Memorandum

Supplementary description of the protocol distributed from Study Chair/Study Coordinator to study personnel in objective, such as reduction of interpretive variation in sentences, and special precautions, rather than change of protocol content.

Any form is used.Approval by both Group Chair and both Director of the Data Center is needed prior to distribution.　Reporting to Data and Safety Monitoring Committee before distribution or immediately after distribution

is required.

It is not necessary to include in on the cover page of the protocol.

### Institutional approval at the time of protocol amendment/revision

If this protocol or informed consent form for patients is amended with JCOG Data and Safety Monitoring　Committee approval and WJOG Respiratory Group meeting approval during the study, the amended protocol and informed consent form must be approved by each participating institution. During patient accrual, the patient registration will be suspended until approval is obtained from each medical institution, and the registration will be resumed sequentially from the institution where approval has been obtained. If amendment is approved, the site coordinator at each institution will send copies of the site's approval document to each data center. Original approval documents will be stored by the site coordinator, and copies will be stored by the data center.

If the content change is revised (not amended), approval by each institution is also required. Whether the type of review is a routine review, or an accelerated review is left to the discretion of each institution.

If approval for the amendment is obtained, the site coordinator of each institution will send copies of　the approval documents of each group's data center. Original approval documents will be stored by the site coordinator, and copies will be stored by each data center.

## Control of Conflicts of Interest (COIs) of Persons Involved in JCOG Studies

### Control of Conflicts of Interest (COIs) of Persons Involved in JCOG Studies

COIs for researchers involved in JCOG studies and for those supporting JCOG studies are managed as follows: 1) COIs for those involved in JCOG studies in clinical practice at participating institutions, such as site investigators and site coordinators, shall be in accordance with the regulations of participating institutions.

2) COIs of those involved in JCOG studies, such as Study Chair and Study Coordinator, Group Chair and the Group Secretariat, are managed by JCOG COI Committee. COIs for other JCOG members such as the members of Data and Safety Monitoring Committee and JCOG Data Center/Operations Office involved in individual JCOG studies should be managed as well.

### Control of Conflicts of Interest (COIs) of Persons Involved in WJOG Studies

COIs of researchers involved in the study of WJOG and those supporting WJOG studies should be managed as follows.

1) COIs for those involved in WJOG studies in clinical practice at participating institutions, such as site investigators and site coordinators, shall be in accordance with the regulations of participating institutions.

2) COIs of persons involved in WJOG studies, such as Study Chair and Study Coordinator, Group Chair and the Group Secretariat, are managed by WJOG Ethics Committee. In addition, the COIs of members of WJOG Data and Safety Monitoring Committee and other members of WJOG Data Center/Secretariat involved in individual WJOG studies will be managed as well.

### 本試験に中心的な役割を持つ者のCOIについて

[JCOG]

In the event that the Principal Investigator, the Research Office, the Group Representative, or the Group Secretariat has a COI that exceeds a certain amount as specified in the JCOG Conflict of Interest Policy, the relevant COI will be disclosed on the JCOG website and updated approximately once a year. The COI of the above four parties at the start of this study are as follows.

There are no conflicts of interest related to this study that should be disclosed.

[WJOG]

**【WJOG】**

In the event that the principal investigator, research secretariat, group representatives, etc. have a COI above a certain amount as defined by the WJOG Ethics Committee, the relevant COI will be disclosed on the website and updated approximately once a year. The COI of the above four parties at the start of this study are as follows.

There are no conflicts of interest related to this study that should be disclosed.

## Compensation

Health hazards caused by participating in this clinical trial will be provided as insurance medical care with appropriate treatment according to the condition as well as usual medical care. At that time, the out　of-pocket payments for medical costs will be paid by patients. In addition, financial compensation, such as payments and various treatments, is not provided.

## Intellectual Property

The findings, data, and intellectual property rights obtained from this trial are attributed to Study Chair, Study Coordinator, Group Chair, National Cancer Center, and NPO-West Japan Oncology Group. Specific treatment and allocation shall be determined after due consultation between the parties hereto. Individuals belong to the intellectual property related to Study Chair, Study Coordinator, and Group Chair or to the affiliated medical institution in accordance with the rules of the affiliated medical institution.

## Disclosure of information on this study

Summary, progress, and main results of this study will be disseminated on JCOG website (www. jcog.jp) and UMIN-CTR (www. umin.ac. jp/ctr /).

# Monitoring and audit

## Periodic monitoring

In principle, periodic monitoring is performed twice a year for objective of ensuring that the study is conducted safely and in accordance with the protocol, and that data are accurately collected.

Monitoring is central monitoring conducted by the Data Center based on CRF input data collected to the Data Center, and site visit monitoring, including source document verification, will not be conducted.

JCOG Data Center and WJOG Data Center will simultaneously generate periodic monitoring reports twice a year based on the data collected in each. Periodic monitoring reports created at individual data centers will be interchanged and reviewed through JCOG Study Coordinator/WJOG Study Coordinator.

Periodic monitoring reports prepared by JCOG Data Center will be submitted to JCOG Study Coordinator, WJOG Study Coordinator, JCOG Study Chair, JCOG Group Chair, JCOG Data and Safety Monitoring Committee, and JCOG Chair and reviewed in accordance with JCOG monitoring regulations.

Periodic monitoring reports generated by WJOG Data Center will be submitted to WJOG Study Coordinator, JCOG Study Coordinator, WJOG Study Chair, and WJOG Group Chair and reviewed in accordance with WJOG monitoring regulations.

Since objective of periodic monitoring is to provide feedback on issues and increase the scientific and ethical nature of the study and not to identify issues related to the study or institution, both Study Coordinator, both Study Chair, both Group Chair, and Site Investigators will strive to improve issues identified in the periodic monitoring report.

### Monitoring items

1) Accrual status: number of enrollment-cumulative/monthly, by arm/by site

2) Eligibility: Ineligible/Potentially ineligible patients: by arm/by site

3) During protocol treatment/end of treatment: Reason for treatment termination/completion: by arm/by site

4) Pretreatment background factors: by arm

5) Serious Adverse Events: by arm/by site

6) Adverse Reactions/Adverse Events: by arm

7) Protocol Deviation: by arm/by site

8) Overall survival, relapse-free survival: all registered patients

9) Other issues related to study progress and safety.

### Eligibility (Eligible/Ineligible)

For all enrolled patients, eligibility will be classified according to the following definitions as: When monitoring, data centers shall list potentially ineligible cases in the "Evaluation of Eligibility" column of the monitoring report. After CRF review by Study Coordinator, either 1), 2), 9), or 99) is finally confirmed with Group Chair approval prior to primary analysis.

Only 1) eligible shall be "eligible case", 2) post hoc ineligible,9) de facto ineligible and 99) violation of registration shall be "ineligible case".

1) Eligible

All information generated prior to registration meets all of the Patients Selection Criteria according to the methods and criteria specified in the study protocol.

2) Post hoc ineligible

The information generated after registration does not meet either Patients Selection Criteria, or the information generated prior to registration does not meet either Patients Selection Criteria by methods or criteria other than those specified in the protocol.

Examples)

i) In the study for Stage II-III, bone scintigraphy performed immediately after registration revealed bone metastases, and the patient was diagnosed as Stage IV. The protocol treatment was terminated. The protocol treatment was terminated.

ii) In the study for early gastric cancer, bloody stools are seen after registration, and colonoscopy revealed advanced colorectal cancer (synchronous double cancer). Colectomy was performed after termination of the protocol treatment.

iii) In the study for gastric cancer (adenocarcinoma), the institution's pathological diagnosis was changed to malignant lymphoma after registration.

9) De facto ineligible

Information generated prior to registration according to protocol-specified methods (performed in all cases) and criteria does not meet either Patients Selection Criteria. This includes cases where it is determined after registration that the information that occurred before registration had been incorrect.

Example: When the supervising physician reviews the CT, images performed before registration as specified, there is obvious liver metastasis (if it is a mistake by the attending physician and it is considered that there is no future).

99) Violation of registration

Deliberately (falsely) enroll while knowing that Patients Selection Criteria is not met. Corresponds to a misconduct and treats it as a serious problem.

### Protocol deviations/violations

Protocol deviations are defined as those in which treatment, such as drug administration, radiotherapy, or surgical resection, as well as laboratory tests and evaluation of toxicity and efficacy, etc. was not performed according to the protocols.

In monitoring, deviations that exceed a certain acceptable range limit for each study decided by both Data Center and Study Chair/Study Coordinator prior to or after the initiation of the study are listed in the monitoring report as "possible deviation" and are classified into one of the following categories after consideration by both Study Coordinator and both study groups Both Study Coordinator should communicate closely with respect to the acceptable range of deviations and use the same acceptable range as possible.

1) Violation

Any deviation from the protocol that is clinically inappropriate and caused by the treating physician/institution and that meets two or more following criteria shall be classified as a violation.

i) Have a substantial impact on the assessment of study endpoints.

ii) Intentional or systematic

iii) Dangerous or remarkable deviation

For "violations", in principle, the content of each violation should be described in a paper when publishing.

2) Deviation

Deviations that do not fall into 1) violation or 3) acceptable deviation.

If same kind of deviations are frequent, they should preferably be included in the publication of the article. They are classified as either of the following at the time of monitoring report review:

i) Deviations...-Undesirable and to be sublober.

ii) Deviations (unavoidable) - things that are not proactively reducing.

iii) Deviations (clinically relevant) - Those in which the decision of the treating

physician/institution are positively affirmed.

3) Acceptable deviation

Deviations from protocols within acceptable range agreed by the entire JCOG and the entire WJOG, or both Study Chair/Study Coordinator and both Data Center, pre- or post-study initiation, on a trial-by-trial basis. Deviations within the pre-specified acceptable ranges are not included in the monitoring report.

## Site visit audits

[JCOG]

In JCOG, site visit audits are conducted in accordance with JCTN-Audit Guidelines (http://jctn.jp/guideline.html)) for objective of improving the scientific and ethical quality of studies. Site visit audits are conducted by researchers (auditors) within JCOG designated by the Audit Committee who will visit the participating sites in the study and follow JCOG audit policy (http://www.jcog.jp/basic/policy/index.html) set out by the Audit Committee, including checking the approval documents of the medical institution, checking the informed consent documents, and verifying the CRF entry data with the medical records (direct access to source documents).

In addition, the audit results of each institution are reported to the site investigators, the administrator of the institution, Study Coordinator, Study Chair, Group Chair, the Director of JCOG Data Center, the Director of JCOG Operations Office, and JCOG Chair as well as the results of the review of the audit report by JCOG Audit Committee. Reports are also made to the researchers in the group and JCOG Executive Committee as appropriate. If they are published to the others, the name of the institution should be masked.

[WJOG]

In WJOG, site visit audits are conducted by a separate WJOG Institutional Review Committee. The findings will be reported in writing to the site investigators, the administrators of the institution and WJOG President by the chair of the institutional review committee.

# Special Instructions

No central pathology diagnosis or extra-institutional determination of tumor response will be performed.

## JCOG BioBank Japan (BBJ) Biorepository

This study will participate in the banking of blood samples (DNA/plasma) in JCOG BBJ Biorepository based on a common protocol for all JCOG studies (hereafter referred to as common banking).

## Multiple Study Enrollment

The study will include an ancillary biomarker study in which tumor samples and plasma will be analyzed using digital PCR and next-generation sequencers.

In order to get a rough idea of the number of patients who may participate in the ancillary studies, physicians may ask patients at the time of enrollment whether or not they are willing to participate in the ancillary studies.

<Additions in ver. 2.0>

Ancillary study JCOG1404A1 (Biomarker study to analyze the mechanism of resistance acquisition to EGFR tyrosine kinase inhibitors and to investigate the usefulness of liquid biopsy) is currently being conducted, and JCOG1404A1 will be revised according to the revised protocol of this study.

# Organization

Changes to this chapter are considered to be revision rather than amendment.

Although revision review of Data and Safety Monitoring Committee is not required, it must be approved by each Group Chair. In the event of a change, each Study Chair/Study Coordinator will promptly inform all participating institutions, JCOG Data Center, and WJOG Data Center of the changes in writing.

## Main study fund (funding source) of this study

- National Cancer Center Research and Development Fund 2020-J-3, Principal Investigator: Yuichiro Ohe

"Scientific research on multi-institutional trials to establish new standard treatment of solid tumors in adults."

- National Cancer Center Research and Development Fund 29-A-3, Principal Investigator: Yuichiro Ohe

"Scientific research on multi-institutional trials to establish new standard treatment of solid tumors in adults."

- National Cancer Center Research and Development Fund 29-A-3, Principal Investigator: Kensei Tobinai

"Scientific research on multi-institutional trials to establish new standard treatment of solid tumors in adults."

- Japan Agency for Medical Research and Development Entrusted R&D Expenditure Innovative Cancer Medicine Practical Research Project　20ck0106492h0002

“A phase III study comparing gefitinib or osimertinib and inserted cisplatin and pemetrexed with gefitinib or osimertinib as a first-line treatment for patients with advanced non-squamous non-small-cell lung cancer harboring *EGFR* activating mutation （JCOG1404/WJOG8214L:AGAIN)”

- Japan Agency for Medical Research and Development Entrusted R&D Expenditure Innovative Cancer Medicine Practical Research Project　19ck0106492h0001“A phase III study comparing gefitinib or osimertinib and inserted cisplatin and pemetrexed with gefitinib or osimertinib as a first-line treatment for patients with advanced non-squamous non-small-cell lung cancer harboring *EGFR* activating mutation （JCOG1404/WJOG8214L:AGAIN)”
- Japan Agency for Medical Research and Development Entrusted R&D Expenditure Innovative Cancer Medicine Practical Research Project　18ck0106221h0003

“A phase III study comparing gefitinib or osimertinib and inserted cisplatin and pemetrexed with gefitinib or osimertinib as a first-line treatment for patients with advanced non-squamous non-small-cell lung cancer harboring *EGFR* activating mutation （JCOG1404/WJOG8214L:AGAIN)”

- Japan Agency for Medical Research and Development Entrusted R&D Expenditure Innovative Cancer Medicine Practical Research Project　17ck0106221h0002

“A phase III study comparing gefitinib and inserted cisplatin and pemetrexed with gefitinib as a first-line treatment for patients with advanced non-squamous non-small-cell lung cancer harboring *EGFR* activating mutation （JCOG1404/WJOG8214L:AGAIN)”

- Japan Agency for Medical Research and Development Entrusted R&D Expenditure Innovative Cancer Medicine Practical Research Project　16ck0106221h0001

“A phase III study comparing gefitinib and inserted cisplatin and pemetrexed with gefitinib as a first-line treatment for patients with advanced non-squamous non-small-cell lung cancer harboring *EGFR* activating mutation （JCOG1404/WJOG8214L:AGAIN)”

## JCOG （Japan Clinical Oncology Group）

JCOG is a multi-institutional clinical research group consisting of research teams funded by public research grants mainly on National Cancer Center Research and Development Fund and Japan Agency for Medical Research and Development research costs that receive direct support for research by the Clinical Research Support Office of the National Cancer Center Hospital in accordance with JCOG Policy (<http://www.jcog.jp/>).

This study is conducted using JCOG research organizations and in accordance with the regulations set out by JCOG Executive Committee.

## WJOG （West Japan Oncology Group）

WJOG (West Japan Oncology Group) is a specified non-profit organization that is mainly based on multi-institutional research on cancer. This study will be conducted using WJOG study organization and in accordance with the regulations set by WJOG Council. (<http://www.wjog.jp/>)

## JCOG Chair

Yuichiro Ohe, National Cancer Center Hospital

## WJOG President

Kazuhiko Nakagawa, Department of Medical Oncology, Kindai University Faculty of Medicine

## Study Group and Group Chair

### JCOG Lung Cancer Internal Medicine Group Chair

Yuichiro Ohe

Department of Respiratory Medicine, Central Hospital, National Cancer Research Center

〒104-0045 Tsukiji 5-1-1, Chyuo-ku, Tokyo

TEL: +81-3-3542-2511

FAX: +81-3-3545-5370

E-mail: [yohe@ncc.go.jp](mailto:yohe@ncc.go.jp)

Group coordinator: Hidehito Horinouchi

Department of Respiratory Medicine, Central Hospital, National Cancer Research Center

〒104-0045 Tsukiji 5-1-1, Chyuo-ku, Tokyo

TEL: +81-3-3542-2511

FAX: +81-3-3545-5370

E-mail：hhorinou@ncc.go.jp

### WJOG Respiratory Group Chair (Internal Medicine)

Nobuyuki Yamamoto

Department of Respiratory Medicine and Medical Oncology, Wakayama Medical University

811-1, Kimiidera, Wakayama, Wakayama 641-8509

<TEL:073-447-2300>

FAX:073-446-2877

E-mail: nbyamamo@wakayama-med.ac.jp

## Study Chair

### JCOG Study Chair

Yuichiro Ohe

Department of Respiratory Medicine, Central Hospital, National Cancer Research Center

〒104-0045 Tsukiji 5-1-1, Chyuo-ku, Tokyo

TEL: +81-3-3542-2511

FAX: +81-3-3545-5370

E-mail: [yohe@ncc.go.jp](mailto:yohe@ncc.go.jp)

### WJOG Study Chair

Nobuyuki Yamamoto

Department of Respiratory Medicine and Medical Oncology, Wakayama Medical University

811-1, Kimiidera, Wakayama, Wakayama 641-8509

<TEL:073-447-2300>

FAX:073-446-2877

E-mail: nbyamamo@wakayama-med.ac.jp

## Study Coordinator

### JCOG Study Coordinator

Shintaro Kanda

Shinshu Cancer Center, Shinshu University Hospital

〒390-8621 3-1-1 Asahi, Matsumoto City, Nagano Prefecture

TEL: +81-263-37-2554

FAX: +81-263-37-3302

E-mail: [skanda@shinshu-u.ac.jp](mailto:skanda@shinshu-u.ac.jp)

Seiji Niho

Department of Pulmonary Medicine and Clinical Immunology, Dokkyo Medical University

〒321-0293 880 Oaza Kitakobayashi, Mibu-cho, Shimotsuga-gun, Tochigi Prefecture

TEL: +81-282-86-1111

FAX: +81-282-86-7780

E-mail: [siniho@dokkyomed.ac.jp](mailto:siniho@dokkyomed.ac.jp)

### WJOG Study Coordinator

Takayasu Kurata

Department of Thoracic Oncology, Kansai Medical University Hospital

〒573-1191 2-3-1 Hirakata Municipality, Osaka

TEL: 072-804-0101

FAX: 072-804-0131

E-mail: kuratat@hirakata.kmu.ac.jp

## Participating Institutions

### JCOG participating institutions

The participating institutions scheduled to participate in this study are listed below. Institutions marked with △ are those that have transitioned to cooperating medical institutions.

|  | Institution | Department | Principal investigator | Estimated annual registrations |
| --- | --- | --- | --- | --- |
| ○ | National Hospital Organization Asahikawa Medical Center | Respiratory Medicine | Fujita Yuka | 2 |
| ○ | National Hospital Organization Hokkaido Cancer Center | Respiratory Medicine | Satoshi Oizumi | 2 |
| ○ | Hokkaido University Hospital | Internal Medicine I | Hajime Asahina | 2 |
| ○ | Iwate Medical University | Respiratory, Allergy and Collagen Diseases | Makoto Maemondo | ＊ |
| ○ | Miyagi Cancer Center | Respiratory Medicine | Tatsuro Fukuhara | 2 |
| ○ | Sendai Kosei Hospital | Respiratory Medicine | Shunichi Sugawara | ＊ |
| △ | Yamagata Central Hospital | Internal Medicine | Toshihiko Hino | 3 |
| ○ | Tochigi Cancer Center | Respiratory Medicine | Takashi Kasai | 3 |
| ○ | Gunma Cancer Center | Respiratory Medicine | Koichi Minato | 2 |
| ○ | Saitama Cancer Center | Respiratory Medicine | Hiroshi Sakai | 2 |
| ○ | National Cancer Center Hospital East | Internal Medicine | Koichi Goto | 4 |
| ○ | National Cancer Center Central Hospital | Respiratory Medicine | Uichiro Ohe | 6 |
| ○ | Cancer and Infectious Disease Center Tokyo Metropolitan Komagome Hospital | Respiratory Medicine | Yukio Hosomi | 4 |
| ○ | National Center for Global Health and Medicine Hospital | Respiratory Medicine | Yuichiro takeda | 2 |
| ○ | Showa University Hospital | Medical Oncology | Atsushi Horiike | ＊ |
| ○ | Cancer Institute Hospital | Respiratory Medicine | Noriko yanagitani | 4 |
| ○ | Toranomon Hospital | Respiratory Center Internal Medicine | Hisashi Takaya | ＊ |
| ○ | Juntendo University Hospital | Respiratory Medicine | Kazuhisa Takahashi | 2 |
| ○ | Nippon Medical School Hospital | Department of Chemotherapy / Respiratory Medicine | Kaoru Kubota | 2 |
| ○ | Teikyo University School of Medicine | Medical Oncology | Nobuhiko Seki | 2 |
| ○ | Kanagawa Cancer Center | Respiratory Medicine | Terufumi Kato | 4 |
| ○ | Yokohama City Municipal Hospital | Respiratory Medicine | Hiroaki Okamoto | 4 |
| ○ | Kitasato University School of Medicine | Respiratory Medicine | Katsuhiko Naraki | 2 |
| ○ | Niigata Cancer Center Niigata Hospital | Internal Medicine | Hiroshi Tanaka | 4 |
| ○ | Shinshu University School of Medicine | Shinshu Cancer Center / Respiratory Medicine | Tomonobu Koizumi | ＊ |
| ○ | Gifu City Hospital | Respiratory Medicine | Toshiyuki Sawa | 4 |
| ○ | Shizuoka Cancer Center | Respiratory Medicine | Toshiaki Takahashi | 4 |
| ○ | Aichi Cancer Center | Respiratory Medicine | Toyoaki Hida | 4 |
| ○ | National Hospital Organization Nagoya Medical Center | Respiratory Medicine・Medical Oncology | Masahide Oki | 4 |
| ○ | Nagoya University School of Medicine | Respiratory Medicine | Masahiro Morise | 2 |
| ○ | Fujita Medical College | Respiratory Medicine | Kazuyoshi Imaizumi | 4 |
| ○ | Osaka City University Hospital | Respiratory Medicine | Hiroyasu Kaneda | ＊ |
| ○ | Kinki University Hospital | Medical Oncology | Kazuhiko Nakagawa | 4 |
| ○ | Osaka International Cancer Center | Respiratory Medicine | Toru Kumagai | 4 |
| ○ | Osaka Habikino Medical Center | Medical Oncology | Tomonori Hirashima | 6 |
| ○ | National Hospital Organization Kinki Central Respiratory Center | Internal Medicine | Shinji Atagi | 4 |
| ○ | Osaka Acute & Comprehensive Medical Center | Respiratory Medicine | Kiyonobu Ueno | ＊ |
| ○ | Osaka City General Medical Center | Medical Oncology | Haruko Daga | 4 |
| ○ | Kansai Medical University Hospital | Pulmonary Oncology | Takayasu Kurata | 4 |
| △ | Kobe City Medical Center Chuo Municipal Hospital | Medical Oncology | Hisateru Yasui | ＊ |
| ○ | Hyogo Medical University | Respiratory Medicine | Takashi Kijima | ＊ |
| △ | Hyogo Cancer Center | Respiratory Medicine | Miyako Satouchi | 2 |
| ○ | Itami Municipal Hospital | Respiratory Medicine | Satoshi Hara | ＊ |
| ○ | Wakayama Medical University | nternal Medicine III | Nobuyuki Yamamoto | 4 |
| ○ | Kurashiki Central Hospital | Respiratory Medicine | Toshihide Yokoyama | 4 |
| △ | Okayama University Hospital | Respiratory and Allergy Medicine | Katsuyuki Hotta | 4 |
| ○ | Okayama Red Cross Hospital | Respiratory Medicine | Akihiro Bessho | ＊ |
| ○ | Hiroshima University Hospital | Respiratory Medicine | Noboru Hattori | ＊ |
| ○ | National Hospital Organization Yamaguchi-Ube Medical Center | Respiratory Medicine | Kamei Haruhito | 4 |
| ○ | Tokushima University Hospital | Respiratory and Collagen Diseases | Yasuhiko Nishioka | ＊ |
| ○ | National Hospital Organization Shikoku Cancer Center | Respiratory Medicine | Kozuki Toshiyuki | 4 |
| ○ | National Hospital Organization Kyushu Cancer Center | Pulmonary Oncology | Ryo Toyosawa | 4 |
| ○ | Kurume University School of Medicine | Respiratory Disease Center | Tomoaki Hoshino | ＊ |
| ○ | Kyushu University Hospital | Respiratory Medicine | Isamu Okamoto | 4 |
| ○ | Nagasaki University Hospital | Respiratory Medicine (Second Department of Internal Medicine) | Hiroyuki Yamaguchi | ＊ |
| ○ | Kumamoto University Hospital | Respiratory Medicine | Takuro Sakagami | ＊ |

Total 140 （at the start of the study）

### WJOG participating institutions

In order to participate in WJOG clinical trials, a institution must be belong to WJOG and is selected according to rules established by the WJOG Board of Directors, but there is no limit to the number of institutions.

|  | Institution | Department | Principal investigator | Coordinator | Estimated annual registrations |
| --- | --- | --- | --- | --- | --- |
| ○ | Ishikawa Prefectural Central Hospital | Respiratory Medicine | Koichi Nishi | Koichi Nishi | 4 |
|  |  | Respiratory Surgery | Yoshio Tsunezuka | Yoshio Tsunezuka | 20 |
| ○ | Kanazawa University Hospital | Respiratory Surgery | Toshiro Kasahara | Takashi Sone | 2 |
| ○ | Hiroshima University Hospital | Respiratory Medicine | Morihito Okada | Yoshihiro Miyata | 3～4 |
| ○ | Kishiwada Municipal Hospital | Medical Oncology | Tomohiro Ozaki | Junko Tanizaki | 7 |
| ○ | Izumi City General Medical Center | Medical Oncology | Hiroshi Tsukuda | Kaoru matsui  Ichiro Kawai  Asuka Tsutani | 3 |
| ○ | Chiba Cancer Center | Respiratory Medicine | Masato Shingyoji | Hironori Ashinuma | 2 |
| ○ | Osaka Medical College Hospital | Respiratory Medicine・Pulmonary Oncology | Yasuhito Fujisaka | Yosuke Tamura  Kenjiro Tsuruoka | 3 |
| ○ | Kansai Electric Power Hospital | Medical Oncology | Kazuhiro Yanagihara | Kazuhiro Yanagihara | 2 |
| ○ | Iizuka Hospital | Respiratory Disease Center | Noriyuki Ebi | Noriyuki Ebi | 3 |
| ○ | Kinki University Nara Hospital | Medical Oncology | Yusaku Akashi | Yusaku Akashi | 4 |
| ○ | Osaka Saiseikai Nakatsu Hospital | Respiratory Medicine | Yoshinori Hasegawa | Yoshinori Hasegawa | 3 |
| ○ | Kyoto Katsura Hospital | Respiratory Center Internal Medicine | Naoshi Nishimura | Noriko Fujita | 4 |
| ○ | Okazaki Municipal Hospital | Respiratory　Medicine / Medical Oncology | Motoyasu Okuno | Motoyasu Okuno | 1 |
|  | Oita Prefectural Hospital | Pulmonary Oncology | Ryotaro Morinaga | Yasushi Hisamatsu | 3 |
|  | Osaka Toneyama Medical Center | Pulmonary Oncology | Masahide Mori | Yukihiro Yano | 3 |
|  | Kumamoto Regional Medical Center | Respiratory Medicine | Kosuke kashiwabara | Kosuke kashiwabara | 5 |
|  | St. Marianna University Hospital | Respiratory Medicine | Masamichi Mineshita | Naoki Furuya | 3 |

## JCOG Protocol Review Committee

This protocol was approved by JCOG Protocol Review Committee prior to submit to IRB-review at participating institutions. (See Web site http://www.jcog.jp/basic/org/committee/protocol.html for membership composition and affiliation.)

Contact: Protocol Review Committee Office

JCOG Operations Office/Clinical Research Support Office, National Cancer Center Hospital

〒104-0045 5-1-1, Tsukiji, Chuo-ku, Tokyo

TEL: 03-3542-2511 (ext. 2302)

FAX:03-3542-7006

E-mail: jcogoffice@ml.jcog.jp

## Data and Safety Monitoring Committee

### JCOG Data and Safety Monitoring Committee

During study period, this study is monitored by Data and Safety Monitoring Committee (adverse event reports, interim analysis reviews, monitoring report reviews, protocol revision reviews, etc.).

(See the website http://www.jcog.jp/basic/org/committee/jury.html for the composition of the members. The members of the study groups that conduct this study do not participate directly in the review of the study.)

Contact: JCOG Data and Safety Monitoring Committee Office

Clinical Research Support Office, National Cancer Center Hospital/JCOG Operations Office

5-1-1, Tsukiji, Chuo-ku, Tokyo 104-0045

TEL: 03-3542-2511 (ext. 2403)

FAX:03-3542-7006

E-mail: jcogoffice@ml.jcog.jp

### WJOG Data and Safety Monitoring Committee

During study period this study is monitored by WJOG Data and Safety Monitoring Committee.

(The composition is as of Aug 2018.)

Chairman: Shinzo Kudo, Osaka Social Medical Center Affiliated Hospital

Committee Members: Kouichi Takayama, Kyoto Prefectural University of Medicine Hospital

Kensei Yamaguchi, Cancer Institute Hospital

Kenjiro Aogi, Shikoku Cancer Center

## Audit Committee

### JCOG Audit Committee

Site-visit audits by Audit Committee will be conducted during study period.

(See Website http://www.jcog.jp/basic/org/committee/audit.html for membership)

Contact: JCOG Audit Committee Office

Clinical Research Support Office, National Cancer Center Hospital/ JCOG Operations Office

5-1-1, Tsukiji, Chuo-ku, Tokyo 104-0045

TEL: 03-3542-2511 (ext. 2403)

FAX:03-3542-7006

E-mail: jcogoffice@ml.jcog.jp

### WJOG Audit Committee

Site-visit audits by Audit Committee will be conducted during study period.

Chairman: Hiroto Tada

Contact： wjog@wjog.jp

## Conflict of Interest

### JCOG Conflict of Interest Committee

JCOG investigators involved in the study will be governed by the JCOG Conflict of Interest Committee d uring the study period. (For the composition of the committee members, please refer to the website: http://www.jcog.jp/basic/org/committee/coi.html)

Contact: JCOG Conflict of Interest Committee

Clinical Research Support Office, National Cancer Center Hospital/ JCOG Operations Office

5-1-1, Tsukiji, Chuo-ku, Tokyo 104-0045

TEL: 03-3542-2511 (ext. 2403)

FAX:03-3542-7006

E-mail: jcogoffice@ml.jcog.jp

### WJOG Ethics Committee

WJOG investigators involved in the study will be governed by the WJOG Ethics Committee d uring the study period.

(The composition is as of May 2020.)

Chairman: Masahiro Fukuoka, Izumi City General Medical Center

Vice Chair: Hiroto Tada, Suita Tokushukai Hospital

Committee Member: Hirohiko Ikeda, Oebashi Law Office

Mitsunori Hamamoto, Cancer Living with Cancer Association

Masahiro Tanaka, Takarazuka City Hospital

Fumio Imamura, Osaka International Cancer Center

Takao Tamura, Kinki University Nara Hospital

Kaoru Matsui, Izumi City General Medical Center

Mitsushi Matsumoto, Hyogo Cancer Center

## JCOG Data Center/Operations Office

JCOG Data Center

Haruhiko Fukuda, Director of Data Center

Clinical Research Support Office, National Cancer Center Hospital

5-1-1, Tsukiji, Chuo-ku, Tokyo 104-0045

TEL:03-3542-3373

FAX:03-3542-3374

E-mail: jcogdata@ml.jcog.jp

JCOG Operations Office

Kenichi Nakamura, Director of Operations Office

Clinical Research Support Office, National Cancer Center Hospital

5-1-1, Tsukiji, Chuo-ku, Tokyo 104-0045

<TEL:03-3547-1002>

FAX:03-3547-1002

E-mail: jcogoffice@ml.jcog.jp

Official website http://www.jcog.jp/

Study group personnel

JCOG Data Center

Statistical Section Taro Shibata

Data Management Section Tomoko Kazato / Tetsuharu Miyata

JCOG Operations Office

Study Coordinating Section Tomoko Kataoka / Hideki Masai

## WJOG Data Center

Shinichiro Nakamura, Director of Data Center

Room 304 of Namba Plaza Building, 1-5-7, Motomachi, Naniwa-ku, Osaka 556-0016

<TEL:06-6633-7400>

FAX:06-6633-7405

E-mail: datacenter@wjog.jp

## Protocol development

Protocol development

Shintaro Kanda

Department of Respiratory Medicine, Central Hospital, National Cancer Research Center

Protocol development support

JCOG Data Center

Statistical Section Taro Shibata / Shogo Nomura

Data Management Section Harumi Kaba

JCOG Operations Office

Protocol development support Tomoki Mizutani / Aya Kimura

Informed concent document Aya Kimura

# Publication of the study results and completion of the study

## Papers and conference presentations

Primary publications will be published in English journals.

No publication other than primary analysis, final analysis and interim analysis with publication purpose specified in the protocol will be performed unless previously approved by Data and Safety Monitoring Committee.

However, paper publication including review article and conference presentation of introduction of the study which does not include the analytical results of the endpoint of the study, are allowed when JCOG Group Chair, WJOG Group Chair, JCOG Data Center Director and WJOG Data Center Director agree to them.

In principle, the authors of the primary publication of the study results shall be the first JCOG Study Coordinator, followed by WJOG Study Coordinator, JCOG Study Chair, WJOG Study Chair, and statistician at each data center (one person each in charge at the time of the publication analysis). The following shall be based on the order of selecting site investigators or site coordinators by institution in order of increasing enrollment, and finally, WJOG Group Chair and JCOG Group Chair, in accordance with the restrictions provided in the article's posting regulations. Ultimately, however, it is decided to discuss and decide between both Study Coordinator, both Study Chair, and both Group Chair according to the enrollment status of each institution.

All co-authors will review the article content prior to posting and only those who agree on the content of the publication. If there is no agreement on the content, Study Chair may not include the researcher in the co-author after Group Chair approval.

Since conference presentations may occur more than once, presentations will be made on a rounds of time from both Study Coordinator, both Study Chair, site investigators, or site coordinators of the institutions with many enrollments. The presenter will be determined by Study Chair with the consent of Group Chair. However, Study Coordinator is responsible for preparing the presentation and presenting the presentation, and in principle, the communication with the Data Center is made by Study Coordinator.

Publishers other than Study Coordinator cannot receive aggregated and analyzed data directly from the Data Center without the approval of Study Coordinator and Data Center Director. Exploratory analyses for conference presentations and paper publication will be conducted after coordinating opinions between JCOG Study Coordinator/Study Chair and WJOG Study Coordinator/Study Chair.

## Primary Endpoint Report and Clinical Summary Report

The procedures are specified from the preparation of the primary endpoint report and clinical summary report. If primary analysis is the final analysis, the primary endpoint report will not be prepared and the clinical summary report will be prepared.

Based on primary analysis report, Study Chair/Study Coordinator will prepare a "Summary of the primary endpoint report" including the results of the analysis of the study endpoints and their interpretations, and submit it to the Data Center for review within 6 months as a general rule. The prepared primary endpoint report will be approved by Group Chair and the Director of the Data Center and submitted to Data and Safety Monitoring Committee and JCOG Chair with primary analysis report.

Summary of the primary endpoint report will be published on JCOG website (http://www.JCOG.jp/).

If primary analysis is the final analysis, the primary endpoint report will not be prepared, and the following clinical summary report will be prepared.

On the basis of the final analysis report, Study Chair/Study Coordinator will prepare a "Summary of the primary endpoint report", including background information on subjects of this study (age, sex, etc.), study design and study progress, analysis results on each endpoint, conclusions of the entire study, interpretation and discussion of the results, etc., and submit it to the Data Center within 6 months as a rule from the issued date the final analysis report.

Summary of the primary endpoint report will be submitted to Data and Safety Monitoring Committee and JCOG Chair with final analysis reports approved by Group Chair and the Director of the Data Center.

Summary of the clinical summary report is available on JCOG website (<http://www.JCOG.jp/>).

## Completion of the study

On the date that the final analysis report is submitted by Data Center to both Study Coordinator, both Study Chair, both Group Chair, both Group Secretary, both Data and Safety Monitoring Committee, JCOG Chair and WJOG President, the study is completed.

Study Chair/Study Coordinator receiving the final analysis report will report the completion of the study with summary of the outcome to the investigators at the participating institution. The timing of distribution of the final analysis report to participating institutions will be determined by Study Chair/Study Coordinator for both groups, taking into account the timing of publication of the primary results, and the final analysis report will be distributed to investigators at participating institutions by Study Chair/Study Coordinator for both groups themselves or through both groups of data centers.

The site investigator who received a report of completion of the study will report the completion of the study and summary of the results to the administrator of the medical institution without delay. Summary may be reported using the Clinical Summary Report prepared by Study Chair/Study Coordinator after the final analysis.

In institutions that did not enroll patients, the date of completion of accrual may be used as the date of completion of the study at the institution.

In JCOG, submit of the clinical summary report (see 17.2) to Data and Safety Monitoring Committee is considered "completion of study control".

# References

1. 部位別がんの統計情報 http://ganjoho.jp/public/statistics/pub/statistics04.html
2. 全国がん罹患モニタリング集計 http://ganjoho.jp/professional/statistics/monita.html
3. Mitsudomi T, et al: Biological and clinical implications of EGFR mutations in lung cancer. Int J Clin Oncol 11: 190-198, 2006
4. Chong CR, et al: The quest to overcome resistance to EGFR-targeted therapies in cancer. Nat Med 19: 1389-1400, 2013
5. Kohno T, et al: KIF5B-RET fusions in lung adenocarcinoma. Nat Med 18: 375-377, 2012
6. Sawabata N, et al: Japanese lung cancer registry study of 11,663 surgical cases in 2004: demographic and prognosis changes over decade. J Thorac Oncol 6: 1229-1235, 2011
7. Schiller JH, et al: Comparison of four chemotherapy regimens for advanced non-small cell lung cancer. N Engl J Med 346: 92- 98, 2002
8. Ohe Y, et al: Randomized phase III study of cisplatin plus irinotecan versus carboplatin plus paclitaxel, cisplatin plus gemcitabine, and cisplatin plus vinorelbine for advanced non-small-cell lung cancer: Four-Arm Cooperative Study in Japan. Ann Oncol 18:317-323, 2007
9. Scagliotti GV, et al: Phase III study comparing cisplatin plus gemcitabine with cisplatin plus pemetrexed in chemotherapy-naive patients with advanced-stage non-small-cell lung cancer. J Clin Oncol 26:3543-3551, 2008
10. Paz-Ares LG, et al: Maintenance therapy with pemetrexed plus best supportive care versus placebo plus best supportive care after induction therapy with pemetrexed plus cisplatin for advanced non-squamous non-small-cell lung cancer (PARAMOUNT): a double-blind, phase 3, randomised controlled trial. Lancet Oncol 13: 247-255, 2012
11. Paz-Ares LG, et al: PARAMOUNT: Final overall survival results of the phase III study of maintenance pemetrexed versus placebo immediately after induction treatment with pemetrexed plus cisplatin for advanced nonsquamous non-small-cell lung cancer. J Clin Oncol 10: 2895-2902, 2013
12. Mok TS, et al: Gefitinib or carboplatin-paclitaxel in pulmonary adenocarcinoma. N Engl J Med 361:947-957, 2009
13. Fukuoka M, et al: Biomarker analyses and final overall survival results from a phase III, randomized, open-label, first-line study of gefitinib versus carboplatin/paclitaxel in clinically selected patients with advanced non-small-cell lung cancer in Asia (IPASS). J Clin Oncol 29:2866-2874, 2011
14. Mitsudomi T, et al: Gefitinib versus cisplatin plus docetaxel in patients with non-small-cell lung cancer harbouring mutations of the epidermal growth factor receptor (WJTOG3405): an open label, randomised phase 3 trial. Lancet Oncol 11:121-128, 2010
15. Mitsudomi T, et al: Updated overall survival results of WJTOG 3405, a randomized phase III trial comparing gefitinib (G) with cisplatin plus docetaxel (CD) as the first-line treatment for patients with non-small cell lung cancer harboring mutations of the epidermal growth factor receptor (EGFR). J Clin Oncol 30: 2012 (suppl; abstr 7521)
16. Maemondo M, et al: Gefitinib or chemotherapy for non-small-cell lung cancer with mutated EGFR. N Engl J Med 362:2380-2388, 2010
17. Inoue A, et al: Updated overall survival results from a randomized phase III trial comparing gefitinib with carboplatin-paclitaxel for chemo-naive non-small cell lung cancer with sensitive EGFR gene mutations (NEJ002). Ann Oncol 24:54-59, 2013
18. Zhou C, et al: Erlotinib versus chemotherapy as first-line treatment for patients with advanced EGFR mutation-positive non-small-cell lung cancer (OPTIMAL, CTONG-0802): a multicentre, open-label, randomised, phase 3 study. Lancet Oncol 12:735-742, 2011
19. Zhou C, et al: Overall survival (OS) results from OPTIMAL (CTONG0802), a phase III trial of erlotinib (E) versus carboplatin plus gemcitabine (GC) as first-line treatment for Chinese patients with EGFR mutation-positive advanced non-small cell lung cancer (NSCLC). J Clin Oncol 2012; 30: 2012 (supple; abstr 7520)
20. Rosell R, et al: Erlotinib versus standard chemotherapy as first-line treatment for European patients with advanced EGFR mutation-positive non-small-cell lung cancer (EURTAC): a multicentre, open-label, randomised phase 3 trial. Lancet Oncol 13:239-246, 2012
21. Sequist LV, et al: Phase III study of afatinib or cisplatin plus pemetrexed in patients with metastatic lung adenocarcinoma with EGFR mutations. J Clin Oncol 31: 3327-3334, 2013
22. Wu YL, et al. Afatinib versus cisplatin plus gemcitabine for first-line treatment of Asian patients with advanced non-small-cell lung cancer harbouring EGFR mutations (LUX-Lung 6): an open-label, randomised phase 3 trial. Lancet Oncol 15: 213-222, 2014
23. Yang JC,　et al: Afatinib versus cisplatin-based chemotherapy for EGFR mutation-positive lung adenocarcinoma (LUX-Lung 3 and LUX-Lung 6): analysis of overall survival data from two randomised, phase 3 trials. Lancet Oncol 16: 141-151,2015
24. Takano T, et al: EGFR mutations predict survival benefit from gefitinib in patients with advanced lung adenocarcinoma: a historical comparison of patients treated before and after gefitinib approval in Japan. J Clin Oncol 26: 5589-5595, 2008
25. Jackman DM, et al: Exon 19 deletion mutations of epidermal growth factor receptor are associated with prolonged survival in non-small cell lung cancer patients treated with gefitinib or erlotinib. Clin Cancer Res 12: 3908-3914, 2006
26. Lee CK，et al: Impact of Specific Epidermal Growth Factor Receptor (EGFR) Mutations and Clinical Characteristics on Outcomes After Treatment With EGFR Tyrosine Kinase Inhibitors Versus Chemotherapy in EGFR-Mutant Lung Cancer: A Meta-Analysis. J Clin Oncol 10; 33 (17): 1958-1965，2015
27. Sekine I, et al: Comparative chemotherapeutic efficacy in non-small cell lung cancer patients with postoperative recurrence and stage IV disease. J Thorac Oncol 4; 518-521, 2009
28） Yu HA， et al: Differences in the survival of patients with recurrent versus de novo metastatic *KRAS*-mutant and *EGFR*-mutant lung adenocarcinomas. Cancer 121 (12): 2078-2082， 2015
29） Kawaguchi T, et al: Gender, histology, and time of diagnosis are important factors for prognosis: analysis of 1499 never-smokers with advanced non-small cell lung cancer in Japan. J Thorac Oncol 5: 1011-1017, 2010
30） Kobayashi S, et al: EGFR mutation and resistance of non-small-cell lung cancer to gefitinib. N Engl J Med 352: 786-792, 2005
31） Engelman JA, et al: MET amplification leads to gefitinib resistance in lung cancer by activation ERBB3 signaling. Science 316: 1039-1043, 2007
32） Sequist LV, et al: Genotypic and histological evolution of lung cancers acquiring resistance to EGFR inhibitors. Sci Trans Med 3: Article ID 75ra26, 2011
33） Ohashi K,　et al: Epidermal growth factor receptor tyrosine kinase inhibitor-resistant disease. J Clin Oncol 31: 1070-1080,2013
34） Goto K, et al: An evaluation study of EGFR mutation tests utilized for non-small-cell lung cancer in the diagnostic setting. 23: 2914-2919, 2012
35） Johnson DH,　et al: Randomized phase II trial comparing bevacizumab plus carboplatin and paclitaxel with carboplatin and paclitaxel alone in previously untreated locally advanced or metastatic non-small-cell lung cancer. J Clin Oncol 22: 2184-2191,2004
36） Scagliotti G, et al: Treatment-by-histology interaction analyses in three phase III trials show superiority of pemetrexed in nonsquamous non-small cell lung cancer. J Thorac Oncol 6: 64-70, 2011
37） Hata A, et al: How sensitive are epidermal growth factor receptor-tyrosine kinase inhibitors for squamous cell carcinoma of the lung harboring EGFR gene-sensitive mutations? J Thorac Oncol 8: 89-95, 2013
38） Shukuya T,　et al: Efficacy of gefitinib for non-adenocarcinoma non-small-cell lung cancer patients harboring epidermal growth factor receptor mutations: a pooled analysis of published reports. Cancer Sci 105: 1032-1037, 2011
39） Chemotherapy in non-small cell lung cancer: a meta-analysis using updated data on individual patients from 52 randomised clinical trials. Non-small Cell Lung Cancer Collaborative Group. BMJ 311:899-909, 1995
40） D’Addario G, et al: Platinum-based versus non-platinum-based chemotherapy in advanced non-small-cell lung cancer: a meta-analysis of the published literature. J Clin Oncol 23: 2926-2936,2005
41） Ardizzoni A, et al: Cisplatin- versus carboplatin-based chemotherapy in first-line treatment of advanced non-small-cell lung cancer: an individual patient data meta-analysis. J Natl Cancer Inst 99: 847-857, 2007
42） Sandler A, et al: Paclitaxel-carboplatin alone or with bevacizumab for non-small-cell lung cancer. N Engl J Med 355: 2542-2550, 2006
43） Reck M, et al: Phase III trial of cisplatin plus gemcitabine with either placebo or bevacizumab as first-line therapy for nonsquamous non-small-cell lung cancer: AVAiL. J Clin Oncol 27: 1227-1234, 2009
44） Reck M, et al: Overall survival with cisplatin-gemcitabine and bevacizumab or placebo as first-line therapy for nonsquamous non-small-cell lung cancer: results from a randomised phase III trial (AVAiL). Ann Oncol 21: 1804-1809, 2010.
45） Takagi Y, et al: Eligibility for bevacizumab as an independent prognostic factor for patients with advanced non-squamous non-small cell lung cancer: a retrospective cohort study. PLoS One 8, e59700, 2013
46） Katakami N, et al: Randomized phase III study comparing gefitinib (G) with erlotinib (E) in patients (pts) with previously treated advanced lung adenocarcinoma (LA): WJOG 5108L. J Clin Oncol 32: 2014 (supple; abstr 8041)
47） Goto K, et al: A prospective, phase II, open-label study (JO22903) of first-line erlotinib in Japanese patients with epidermal growth factor receptor (EGFR) mutation-positive advanced non-small-cell lung cancer (NSCLC). Lung Cancer 82: 109-114,2013
48） Yamamoto N,　et al: LUX-LUNG 3: Afatinib versus cisplatin and pemetrexed in Japanese patients with adenocarcinoma of the lung harboring an EGFR mutation. Ann Oncol 23 (suppl 11): xi6-xi8,2012
49） Mok TSK, et al: Gefitinib/chemotherapy vs chemotherapy in epidermal growth factor receptor (EGFR) mutation-positive non-small-cell lung cancer (NSCLC) after progression on first-line gefitinib: the phase III, randomized IMPRESS study. Ann Oncol 25 (supple 5): v1-v41, 2014
50） Taniguchi K, et al: Intratumor heterogeneity of epidermal growth factor receptor mutations in lung cancer and its correlation to the response to gefitinib. Cancer Sci 99: 929-935, 2008
51） Maheswaran S, et al: Detection of mutations in EGFR in circulating lung-cancer cells. N Engl J Med 24: 366-377, 2008
52） Rosell R, et al: Pretreatment EGFR T790M mutation and BRCA1 mRNA expression in erlotinib-treated advanced non-small-cell lung cancer patients with EGFR mutations. Clin Cancer Res 17: 1160-1168, 2011
53） Su KY, et al. Pretreatment epidermal growth factor receptor (EGFR) T790M mutation predicts shorter EGFR tyrosine kinase inhibitor response duration in patients with non-small-cell lung cancer. J Clin Oncol 30: 433-440, 2012
54） Fujita Y, et al: Highly sensitive detection of EGFR T790M mutation using colony hybridization predicts favorable prognosis of patients with lung cancer harboring activating EGFR mutation. J Thorac Oncol 7: 1640-1644, 2012
55） Giaccone G, et al: A phase III clinical trial of Gefitinib, an EGFR inhibitor, in combination with gemcitabine and cisplatin in advanced non-small-cell lung cancer (INTACT 1). J Clin Oncol 22: 777-784, 2004
56） Herbst RS, et al: Gefitinib in combination with paclitaxel and carboplatin in chemotherapy-naïve patients with advanced non-small-cell lung cancer: results from a phase III clinical trial (INTACT 2). J Clin Oncol 22: 785-794, 2004
57） Kimura T, et al: Apoptotic effects of the docetaxel OSI-774 combination in nonsmall cell lung carcinoma (NSCLC) cells. Proc Am Soc Clin Oncol. 23: 2004, (supple; abstr 7143)
58） Piperdi B, et al: Schedule-dependent interaction between epidermal growth factor inhibitors (EGFRI) and G2/M blocking chemotherapeutic agents (G2/MB) on human NSCLC cell lines in vitro. Proc Am Soc Clin Oncol. 23: 2004, (supple; abstr 7028)
59） Yamaguchi H, et al. Caspase-independent cell death is involved in the negative effect of EGF receptor inhibitors on cisplatin in non-small cell lung cancer cells. Clin Cancer Res 19: 845-854, 2013
60） Wu YL, et al: Intercalated combination of chemotherapy and erlotinib for patients with advanced stage non-small-cell lung cancer (FASTACT-2): a randomised, double-blind trial. Lancet Oncol 14: 777-786, 2013
61） Kanda S, et al: Cytotoxic chemotherapy may overcome the development of acquired resistance to epidermal growth factor receptor tyrosine kinase inhibitors (EGFR-TKIs) therapy. Lung Cancer. 89: 287-93,2015
62） Nishino M, et al: Tumor volume decrease at 8 weeks is associated with longer survival in EGFR-mutant advanced non-small-cell lung cancer patients treated with EGFR TKI. J Thorac Oncol 8: 1059-1068, 2013
63） Ogino A, et al: Emergence of epidermal growth factor receptor T790M mutation during chronic exposure to gefitinib in a non small cell lung cancer cell line. Cancer Res 15: 7807-7814, 2007
64） Kubota K, et al: Phase III randomized trial of docetaxel plus cisplatin versus vindesine plus cisplatin in patients with stage IV non-small-cell lung cancer: the Japanese Taxotere Lung Cancer Study Group. J Clin Oncol 22: 254-261, 2004
65） Fossella F, et al: Randomized, multinational, phase III study of docetaxel plus platinum combinations versus vinorelbine plus cisplatin for advanced non-small-cell lung cancer: the TAX 326 study group. J Clin Oncol 21: 3016-3024, 2003
66） Douillard JY, et al: Comparison of docetaxel- and vinca alkaloid-based chemotherapy in the first-line treatment of advanced non-small cell lung cancer: a meta-analysis of seven randomized clinical trials. J Thorac Oncol 2: 936-946, 2007
67） Yoh K, et al: Prospective observational cohort study of second-line chemotherapy administration after first-line platinum-based chemotherapy for patients with advanced NSCLC in Japan (SAPPHIRE study). J Clin Oncol 31: 2013 (supple; abstr 8058)
68） Von Plessen C, et al: Palliative chemotherapy beyond three courses conveys no survival or consistent quality-of-life benefits in advanced non-small-cell lung cancer. Br J Cancer 95: 966-973, 2006
69） Park JO, et al: Phase III trial of two versus four additional cycles in patients who are nonprogressive after two cycles of platinum-based chemotherapy in non small-cell lung cancer. J Clin Oncol 25: 5233-5239, 2007
70） 制吐薬適正使用ガイドラインver. 1.2 日本癌治療学会編. http://jsco-cpg.jp/item/29/index.html
71） Kawano Y, et al: Pemetrexed and cisplatin for advanced non-squamous non-small cell lung cancer in Japanese patients: phase II study. Anticancer Res 33: 3327-3333, 2013
72） Eisenhauer EA, et al: New response evaluation criteria in solid tumors: Revised RECIST guideline （version 1.1）. Eur J Cancer 45: 228-247, 2009
73） Schoenfeld DA, et al: Nomograms for calculating the number of patients needed for a clinical trial with survival as an endpoint. Biometrics 38: 163-170, 1982
74） Lan KKG, et al: Discrete sequential boundaries for clinical trials. Biometrika 70: 659-663, 1983
75） Spiegelhalter DJ, et al: Applying Bayesian ideas in drug development and clinical trials. Statistics in Medicine 12:1501-1511, 1983
76） Halperin M, et al: An aid to data monitoring in long-term clinical trials. Controlled Clinical Trials 3:311-323,1982
77） Soria JC,et al: Osimertinib in Untreated EGFR-Mutated Advanced Non-Small-Cell Lung Cancer. N Engl J Med 378:113-125,2018
78） Zhang B,et al: Coexistence of sensitive and resistant epidermal growth factor receptor (EGFR) mutations in pretreatment non-small cell lung cancer (NSCLC) patients: First or third generation tyrosine kinase inhibitors (TKIs)? Lung Cancer 117:27-31,2018
79） Imai H, et al: Efficacy and safety of platinum combination chemotherapy re-challenge for relapsed patients with non-small-cell lung cancer after postoperative adjuvant chemotherapy of cisplatin plus vinorelbine. [Chemotherapy.](https://www.ncbi.nlm.nih.gov/pubmed/24480845) 59: 307-13, 2013.
80） Nakamura A, et al: Phase III study comparing gefitinib monotherapy (G) to combination therapy with gefitinib, carboplatin, and pemetrexed (GCP) for untreated patients (pts) with advanced non-small cell lung cancer (NSCLC) with EGFR mutations (NEJ009). J Clin Oncol 36,2018 (suppl; abstr 9005)
81） Park K, et al: Afatinib versus gefitinib as first-line treatment of patients with EGFR mutation-positive non-small-cell lung cancer (LUX-Lung 7): a phase 2B, open-label, randomised controlled trial. Lancet Oncol 17: 577-89,2016
82） Paz-Ares L, et al: Afatinib versus gefitinib in patients with EGFR mutation-positive advanced non-small-cell lung cancer: overall survival data from the phase IIb LUX-Lung 7 trial. Ann Oncol. 28: 270-277,2017
83） Wu YL, et al: Dacomitinib versus gefitinib as first-line treatment for patients with EGFR-mutation-positive non-small-cell lung cancer (ARCHER 1050): a randomised, open-label, phase 3 trial. Lancet Oncol. 18: 1454-1466,2017.
84） Mok TS, et al: Improvement in Overall Survival in a Randomized Study That Compared Dacomitinib With Gefitinib in Patients With Advanced Non-Small-Cell Lung Cancer and EGFR-Activating Mutations. J Clin Oncol. 2018 Jun 4:JCO2018787994.
85） Seto T,et al: Erlotinib alone or with bevacizumab as first-line therapy in patients with advanced non-squamous non-small-cell lung cancer harbouring EGFR mutations (JO25567): an open-label, randomised, multicentre, phase 2 study. Lancet Oncol 15: 1236-44,2014
86） Yamamoto N,et al: Erlotinib plus bevacizumab (EB) versus erlotinib alone (E) as first-line treatment for advanced EGFR mutation–positive non-squamous non–small-cell lung cancer (NSCLC): Survival follow-up results of JO25567. J Clin Oncol 36,2018 (suppl; abstr 9007)
87） Furuya N,et al: Phase III study comparing bevacizumab plus erlotinib to erlotinib in patients with untreated NSCLC harboring activating EGFR mutations: NEJ026. J Clin Oncol 36,2018 (suppl; abstr 9006)
88） Ramalingam SS,et al: Osimertinib As First-Line Treatment of EGFR Mutation-Positive Advanced Non-Small-Cell Lung Cancer. J Clin Oncol 36:841-849,2018
89） 厚生労働省 国際共同治験に関する基本的考え方について. <https://www.pmda.go.jp/files/000157000.pdf>
90） Quan, H,et al. Sample size considerations for Japanese patients in a multi‐regional trial based on MHLW guidance. Pharmaceutical Statistics, 9; 100-112,2012

# 19.  Appendix

- Explanation/ Informed consent form
- Body surface area table
- Toxicity Criteria (CTCAE v4.0-JCOG)
- CRF samples
